# Genomics-assisted characterization of a breeding collection of *Apios americana*, an edible tuberous legume

**DOI:** 10.1038/srep34908

**Published:** 2016-10-10

**Authors:** Vikas Belamkar, Andrew D. Farmer, Nathan T. Weeks, Scott R. Kalberer, William J. Blackmon, Steven B. Cannon

**Affiliations:** 1 Interdepartmental Genetics, Iowa State University, Ames, IA 50011, USA; 2 Department of Agronomy, Iowa State University, Ames, IA 50011, USA; 3 National Center for Genome Resources, Santa Fe, NM 87505, USA; 4 United States Department of Agriculture–Agricultural Research Service, Corn Insects and Crop Genetics Research Unit, Ames, IA 50011, USA; 5 Department of Horticulture, Louisiana Agricultural Experiment Station, Louisiana State University Agricultural Center, Baton Rouge, LA 70803, USA

## Abstract

For species with potential as new crops, rapid improvement may be facilitated by new genomic methods. Apios (*Apios americana* Medik.), once a staple food source of Native American Indians, produces protein-rich tubers, tolerates a wide range of soils, and symbiotically fixes nitrogen. We report the first high-quality *de novo* transcriptome assembly, an expression atlas, and a set of 58,154 SNP and 39,609 gene expression markers (GEMs) for characterization of a breeding collection. Both SNPs and GEMs identify six genotypic clusters in the collection. Transcripts mapped to the *Phaseolus vulgaris* genome–another phaseoloid legume with the same chromosome number–provide provisional genetic locations for 46,852 SNPs. Linkage disequilibrium decays within 10 kb (based on the provisional genetic locations), consistent with outcrossing reproduction. SNPs and GEMs identify more than 21 marker-trait associations for at least 11 traits. This study demonstrates a holistic approach for mining plant collections to accelerate crop improvement.

Many un- or semi-domesticated edible plants have valuable characteristics not found in existing widely cultivated crops. Such underutilized plants include those adapted to extreme or unusual climatic conditions and soil types; they may also possess resistance against a range of biotic and abiotic stresses. However, the domestication of any new species remains a formidable challenge.

Happily, there are recent examples of rapid progress and success for a few underexploited crops. Quinoa (*Chenopodium quinoa*) breeding has rapidly progressed in the last 4 to 5 years, along with its recent wide acceptance as a food on the world market^1^,^2^,^3^. Perennial grains are being explored as a means to reduce soil degradation and water contamination^4^. DeHaan’s research at The Land Institute to domesticate perennial intermediate wheat grass (Kernza™; *Thinopyrum intermedium*) has been quite successful, with yields increasing by ~77% following two rounds of phenotypic selection^5^. These examples show that rapid improvement of underused crops is feasible.

Apios (*Apios americana* Medik.) is a candidate crop that merits further investigation. Apios is a perennial legume that produces a podded fruit aboveground (as do most other legume crops), which is edible but with small seeds and variable fruit set. More importantly for agricultural use, starchy tubers develop from swellings on stolons (modified subterranean stems) as observed in the potato^6^. Apios tubers have a relatively high protein content for a tuber (11 to 14% on dry defatted basis), long shelf life when stored at ~4 °C (>1 yr), high amounts of novel isoflavones with suspected health benefits, and low levels of reducing sugars^6^. Apios tubers are pleasant to taste, easy to cook and are rich in carbohydrates, proteins, iron and dietary fiber^7^. Recently, a novel isoflavone (genistein-7-O-gentiobioside) has been identified in the tubers. It is deglycosylated to produce the isoflavone genistein^8^. In northern Japan, where Apios is grown as a food crop, apios powder (dried powdered tuber) is used in cookies, donuts, dumplings and bread because of the tuber’s nutritional benefits^7^.

Apios is native to the temperate central and eastern regions of North America, and can thrive in varied growth conditions found in a range of habitats. It grows along the banks of creeks, rivers, and lakes, but can also be grown on well-drained farmland. The versatility of Apios is enhanced by its ability to form root nodules containing symbiotic rhizobia that fix atmospheric nitrogen^9^. Both diploid (2n = 2x = 22) and triploid (2n = 3x = 33)^10^ populations exist in the wild^11^. Triploids are sterile and propagate only asexually via tubers, whereas diploids appear to be generally fertile and may propagate either clonally by tubers or sexually via seeds^11^. The flowers have a complex structure and pollination is achieved when visiting insects set off an explosive tripping mechanism^11^,^12^. Bruneau and Anderson^11^ observed low fruit set in many diploid plants; they attributed these results to partial self-incompatibility and suggested an outcrossing mode of sexual reproduction in Apios^11^.

Historically, Apios was a staple tuber crop for many North American Indian cultures who kept it in a semi-cultivated state near their habitations, and it was well known as an emergency food to early European colonists^13^. Although Apios continued to be consumed by indigenous peoples and wild plant foragers in the centuries that followed, it has remained largely obscure and failed to achieve mainstream acceptance. Apios was reevaluated by Blackmon and Reynolds from 1985–1994 with the objectives of introducing it to cultivation and developing superior cultivars^14^,^15^. They collected and characterized wild germplasm, facilitated open pollinations, selected progeny with desirable agronomic traits, and then repeated the cycles of pollinations and selection (Fig. 1)^14^,^15^. Their breeding effort has lead to improved genotypes that are high yielding, with a number of additional favorable characteristics (e.g., reduced stolon lengths and tuber-to-tuber spacing). In the 21^st^ century we have restarted the research program of Blackmon and Reynolds, recently completing a rigorous phenotypic evaluation of genotypes originating from their breeding program^6^. We have reported these results showing how the various genotypes perform across multiple years and in differing locations and growing conditions^6^.

In the present study, we have generated extensive genomic resources using RNA-Seq and combined them with the previously generated phenotypic data. The goals of this study are as follows: (1) Building a *de novo* reference transcriptome assembly; (2) Developing a gene expression catalog; (3) Identifying SNPs, gene expression markers (GEMs), and genotyping the collection using RNA-Seq; (4) Investigating heterozygosity, pedigree and population structure; (5) Examining linkage disequilibrium; (6) Identifying marker-trait associations using SNPs and gene expression markers; and (7) Combining phenotypic and genetic data to assist in making parental selections for subsequent cultivar development. The results obtained in this study will have broader implications for other plants with limited genomic resources, as well as for staple crops that can be further mined for crop diversity and cultivar development.

## Results

### Development and evaluation of an Apios breeding collection

**Figure 1:**
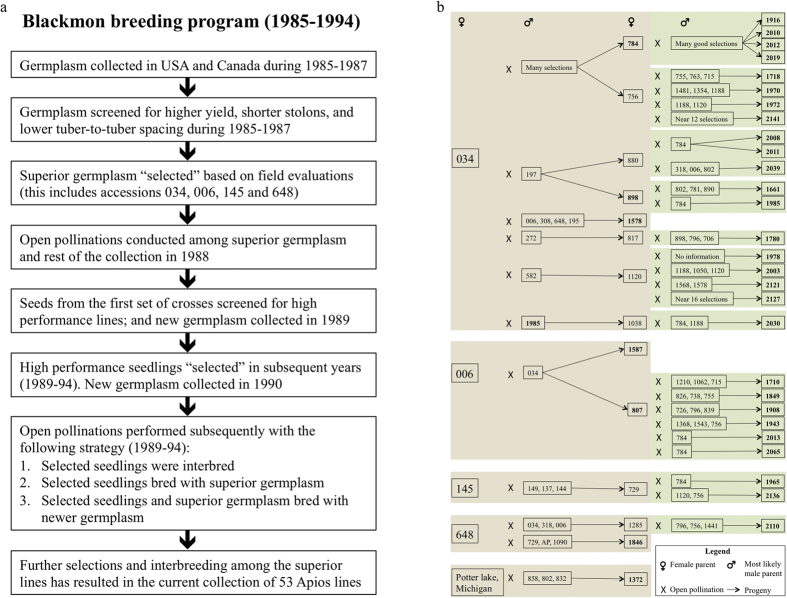
Breeding strategy and pedigree of the *Apios americana* collection. (**a**) Breeding strategy utilized by Dr. Blackmon and Mr. Reynolds during 1985–1994 at Louisiana State University Agricultural Experiment Station in Baton Rouge, LA, for developing elite genotypes used in this study. This information is based on an interpretation of the field books of Dr. Blackmon and Mr. Reynolds. (**b**) Partial maternal lineage information that was traced from the field books for 35 of the 53 genotypes. The genotypes that produced seeds were recorded as maternal parents, and the genotypes that were growing close to the maternal parent were recorded as likely paternal parents. Hence, there are usually multiple paternal parents listed for a genotype. The genotypes in bold are the ones that exist in the collection used in this study.

The plant material used in this study derives from Blackmon and Reynolds’s breeding program from 1985–1994 (Fig. 1). Field books of the breeding program described collection of germplasm, pollinations performed, and phenotypic selections of ~20,000 plants (Fig. 1a). Of those selections, 53 genotypes remained as of 2010–of which 52 were carried forward for use in the present study. Partial pedigree information was traceable for 35 of the 53 (Fig. 1b). “Good-performing” genotypes were repeatedly favored during breeding and this resulted in primary founder genotypes. For instance, genotype “034” contributed to 22 of the 52, and “006” to 8 of the 52 (Fig. 1b). Genotype 034 performed consistently under different growing conditions, and therefore was selected for release in the late 1980s. All of the genotypes were derived through pollinations, and thus are likely to be diploids. Twenty-five of the 53 genotypes tested using flow cytometry had an average genome size of 1644 ± 34 Mb, whereas wild accessions collected from Iowa, New York, and Quebec in Canada had an average genome size of 2380 ± 28 Mb (Supplementary Table 1). The genome size of the genotypes in the Blackmon and Reynolds collection is approximately two-thirds the average genome size of the wild accessions. This result suggests that our particular wild accessions are triploids, whereas the Blackmon and Reynolds genotypes are diploids. These genome size estimates are comparable to the results previously published^16^. Recent field evaluation of the 53 genotypes from the Blackmon and Reynolds collection found high amounts of phenotypic diversity for 18 of the 20 traits measured^6^. The REML-based least square (LS) means generated for each trait from that phenotyping study^6^ are used in this work.

### *De novo* transcriptome assembly, annotation and expression catalog

A transcriptome assembly for *A. americana* was built by sequencing 14 above- and belowground samples, from six tissues, from genotype 2127 (Supplementary Data 1). Sequencing of the 14 samples on Illumina GAIIx/HiSeq generated ~210 million reads, which were then assembled into 96,560 transcripts, and 48,615 components (generally corresponding with genes) (Table 1). The length of the transcripts ranged from 201 to 15,850 bp, with an average of 1,173 bp, and N50 of 1,863 bp (Supplementary Fig. 1)–statistics on par with other *de novo* transcriptome assemblies published to date^17^,^18^,^19^,^20^,^21^. This transcriptome assembly project is deposited at DDBJ/EMBL/GenBank under the accession GEHN00000000. The version described in this article is the first version, GEHN01000000. More than 14% of the transcripts in the assembly are nearly full-length as judged by alignment coverage with soybean and common bean peptides (at >80% coverage, 14,905 (15.4%) for soybean and 14,084 (14.6%) for common bean). The percentage of transcripts matching other legumes with genome sequences and annotations, and Arabidopsis, ranged from 61.1% to 67.5%, and the percentage of peptides from legume species and Arabidopsis that matched to an Apios transcript (allowing only one match per Apios transcript) ranged from 32.4% to 66% (Supplementary Fig. 2; Supplementary Data 2). The percentage of Apios transcripts matching *Phaseolus vulgaris* proteins is 66.5%, while the percentage of *P. vulgaris* proteins matching Apios transcripts is 66% (Supplementary Fig. 2). This nearly one-to-one match between two-thirds of the Apios transcripts and common bean peptides suggests relative completeness of the Apios transcriptome assembly. Extraction of the putative coding regions from the transcripts generated 23,691 unique peptides (Supplementary Data 3). Greater than 70% of these peptides were assigned a function using Swiss-Prot and Pfam databases, and >57% assignments were made using eggNOG and Gene Ontology (Table 1; Supplementary Data 4).

**Figure 2:**
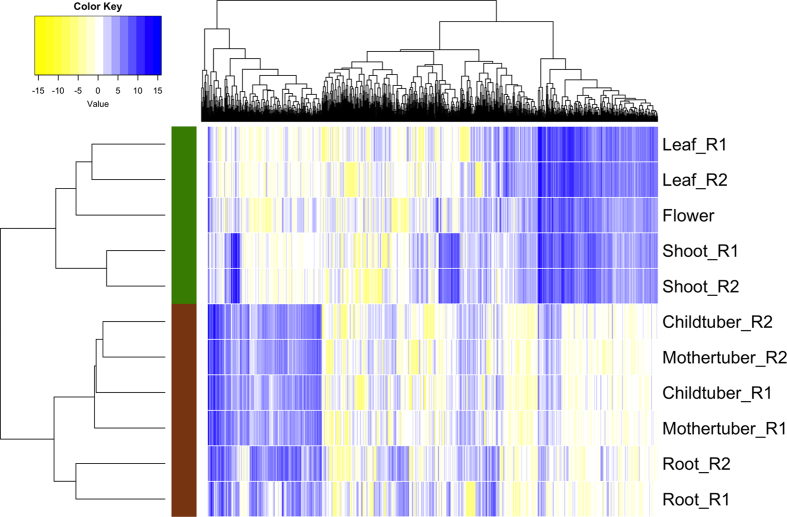
Heat map of the normalized RNA-Seq data showing expression of transcripts in six tissues of accession 2127. The normalized RNA-Seq data is in log_2_ scale. One thousand transcripts with highest variances across the 11 samples were utilized to make the heat map. Letters R1 and R2 represent replicates 1 and 2 of the corresponding tissues. The aboveground and belowground tissues clustered separately and are highlighted in green and brown color respectively.

We also constructed a gene expression catalog, using an average of 14.6 million reads from each of the 11 samples–including six tissues, each with two biological replicates except for flower, which had one sample (Supplementary Table 2). Nearly 90% of the quality-trimmed reads from these samples could be mapped back to the assembly (Supplementary Table 2). We found 56,735 transcripts (Supplementary Data 5), and 28,738 components (Supplementary Data 6) expressed in at least one of the 11 samples. A heat map utilizing expression of 1,000 transcripts with highest variances clustered the samples into two primary subgroups: one consisting of the aboveground tissues, and the other of belowground tissues (Fig. 2).

### Marker discovery, validation and genotyping

The 52 genotypes were grown for three months under field conditions. Four plants grown from four independent seed tubers represented biological replicates for an accession. A young leaf close to the shoot tip was collected from each of the four plants and pooled for RNA isolation. The leaf samples from all the accessions were sampled on the same day within a two-hour window, and at mid-day to minimize the effect of environmental differences on gene expression and to provide relatively uniform staging of tissues. Leaf transcriptomes were sequenced on Illumina Hi-Seq, giving 1.32 billion reads, with an average of 25.3 million reads per genotype (Table 2; Supplementary Data 1). Additionally, transcriptome sequencing of the pooled (shoot and root tissue) samples from four genotypes (as biological replicates), grown for 1.5 months under greenhouse conditions, generated 45.71 million reads. On an average, >88.6% of the reads from each of the 56 samples could be mapped to the transcriptome assembly (Supplementary Data 7). Using stringent filtering criteria (Methods), 58,154 high-quality SNP markers were identified, in 9,338 components (Table 2; Supplementary Data 8). The average reproducibility of the 58,154 high-quality SNP markers tested using four biological replicates was 90.6% (Table 2; Supplementary Table 3). This value is comparable to the reproducibility (78% to 92.9%) observed in other recent studies^22^,^23^,^24^,^25^,^26^. The allele frequency of the G and C alleles was slightly higher in the dataset than the A and T alleles, which is expected, considering the sequences’ transcribed origin (Supplementary Table 4). The percentage of missing SNPs per accession was on average 1.5%, and the average heterozygosity per accession was 37.6 (Supplementary Data 9 and 10). Reads from each of the 52 genotypes mapped to the reference assembly provided 39,609 transcripts that were expressed in at least one genotype. These were used as gene-expression-markers (GEMs) for structure and association analyses (Supplementary Data 11 and 12).

### Diversity, inbreeding and pedigree

We estimated two measures of diversity: (1) the average pair-wise divergence among genotypes, or nucleotide diversity per bp, π (pi), was 0.35; and (2) the expected number of polymorphic sites per nucleotide, or expectation of π, θ (theta), was 0.22. These values are somewhat higher than that of four soybean populations (including elite North American soybean cultivars, Asian landrace founders of these elite cultivars, other Asian landraces, and accessions of the wild progenitor species *Glycine soja*^27^). Tajima’s D was 2.16. Tajima’s D is a normalized measure of the difference between observed (π) and expected (θ) nucleotide diversity. A large positive value of this measurement suggests either that selection has maintained variation in the population, or that the population has contracted–either of which may have occurred during development of this breeding collection.

Inbreeding coefficient estimates for each genotype are analogous to the proportion of heterozygous loci in each genotype, but in the opposite direction–that is, the correlation coefficient is −1.0 between the inbreeding coefficients and heterozygosity of each genotype (Supplementary Data 10). The inbreeding coefficients estimated for each genotype ranged from −0.22 to 0.15, with an average of −0.07–which indicates the generally high heterozygosity of genotypes in the collection (Supplementary Data 10). In fact, inbreeding coefficient values of only five of the genotypes (accessions 1718, 1846, 2148, 2153 and 2170) were positive, indicating that these genotypes are more homozygous than the average of the population^28^. A statistically significant (*P* = 0.0004, *r* = −0.47) negative correlation was observed between inbreeding coefficients and “number of tubers/plant” (Supplementary Table 5; Supplementary Fig. 3). Positive correlations were observed between inbreeding coefficients and three phenotypic traits: leaflets measured 2 months after planting (*P* = 0.0071, *r* = 0.37), mother tuber weight (*P* = 0.0089, *r* = 0.36), and mother tuber width (*P* = 0.019, *r* = 0.32).

Using estimates of Identity-by-Descent (IBD) and the proportion of IBD values, we identified parent-child or half-sib relationships between 24 pairs of genotypes (Supplementary Table 6). Of the 24 pairs, nine pairs were validated using the partial pedigree information available for genotypes (Fig. 1b); one pair (807 and 2003) had conflicting results between IBD analysis and known pedigree information; and the remaining 14 pairs identified by IBD analysis did not have prior pedigree information. All of the pairs identified in the IBD analysis were also identified by fastSTRUCTURE (described in the next section) either by inspecting the proportion of genomes shared, or the clustering of genotypes in the same group.

### Population structure

**Figure 3:**
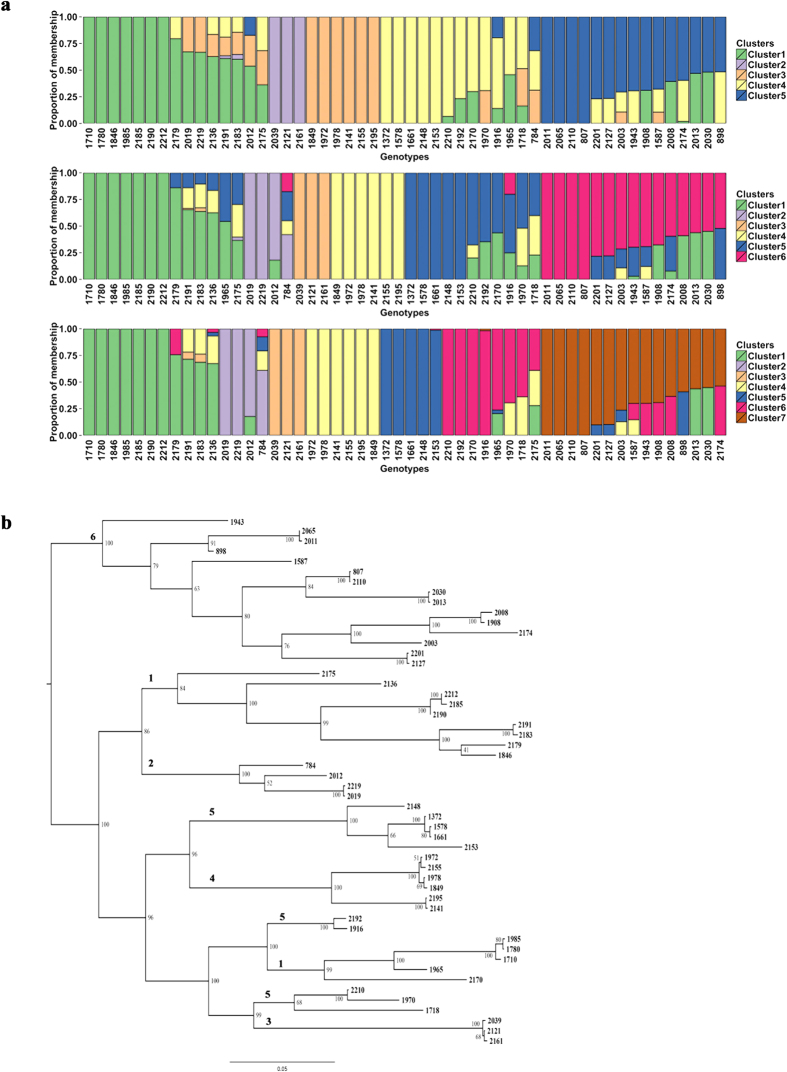
Population structure. (**a**) Population structure using variational Bayesian framework–implemented in the program fastSTRUCTURE. The possible 5, 6 or 7 clusters (K = 5, 6 or 7) identified are shown. The Y-axis represents the proportion of membership of a genotype to the respective cluster, and the X-axis indicates genotypes in the collection. (**b**) Phylogeny built using maximum likelihood implemented in the package SNPhylo. Numbers 1 to 6 represent the clusters identified in Fig. 3a.

**Figure 4:**
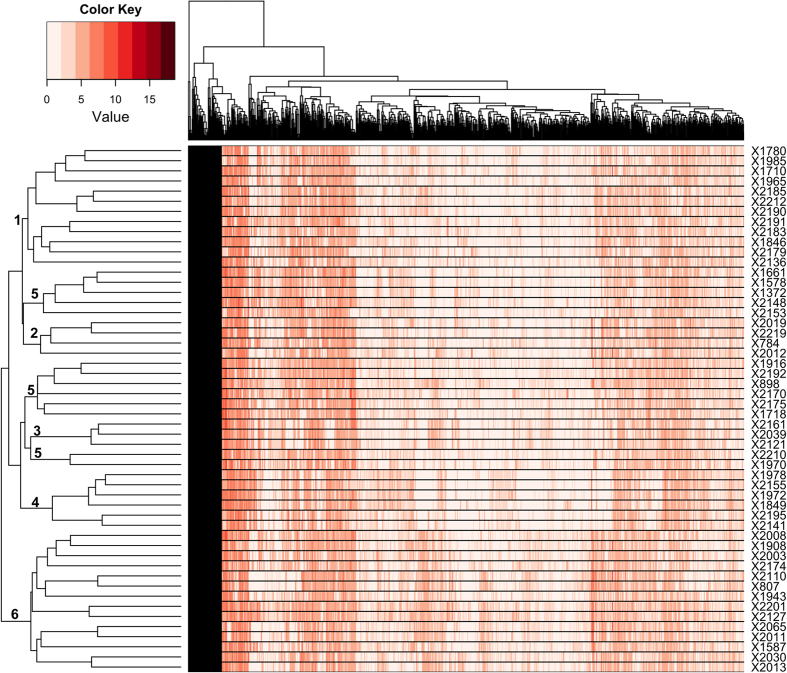
Population structure derived from gene expression markers (GEMs). Phylogeny built using 1,000 GEMs (in log_2_ scale) that show highest variances across the 52 samples. A Euclidean distance matrix was utilized followed by hierarchical clustering with Ward’s linkage. Numbers 1 to 6 represent the clusters identified in Fig. 3a.

We analyzed the structure using five approaches (see Methods for details). These identified at least six clusters (Fig. 3). Bayesian analysis implemented using the program fastSTRUCTURE suggested five (k = 5) to seven (k = 7) clusters (Fig. 3a). The main difference between K = 5 and K = 6 is the inclusion of an additional cluster comprised of genotypes 784, 2012, 2019 and 2219. Genotype 784 is the maternal parent of the genotypes 2012 and 2019 (Fig. 1b), so the additional cluster in K = 6 is reasonable. In the case of K = 7, the additional cluster is formed by splitting the fifth cluster of K = 6. The fifth cluster in K = 6 comprises individuals that are admixed; these split to give the additional cluster in K = 7. However, the admixed individuals have >60% proportion of membership in K = 6, and there is insufficient basis for splitting this cluster. Hence, there are at least six clusters in the collection based on the variational Bayesian analysis conducted using fastSTRUCTURE. In the maximum likelihood approach, fastSTRUCTURE clusters 2, 3, 4, and 6 remained intact, whereas the admixed individuals from clusters 1 and 5 split up and re-grouped with the other clusters (Fig. 3b). Similar results were observed in the IBS and Ward’s clustering approaches with the exception that the fifth cluster split into two clusters as opposed to three in the maximum likelihood approach (Supplementary Fig. 4). The PCA identified six clusters, but a seventh cluster was formed which comprised individuals from clusters 1, 2 and 5 (Supplementary Fig. 5). Interestingly, GEMs also identified similar patterns of structure (Fig. 4). The four clusters (2, 3, 4, and 6) remained intact, and the same was true for cluster 1 that contained a few admixed individuals. However, in the GEM-based analysis, cluster 5 was split up and individuals were re-grouped with other clusters. In summary, the SNP and GEM markers identify at least six clusters–of which five remain intact in fastSTRUCTURE and GEM-based phylogenies. Four of the clusters are consistent across all the five approaches. As a final step, we compared the performance of each of the five approaches in accurately clustering the genotypes based on the pedigree, and the highest success was obtained using fastSTRUCTURE (Supplementary Table 7).

### Linkage disequilibrium (LD)

**Figure 5:**
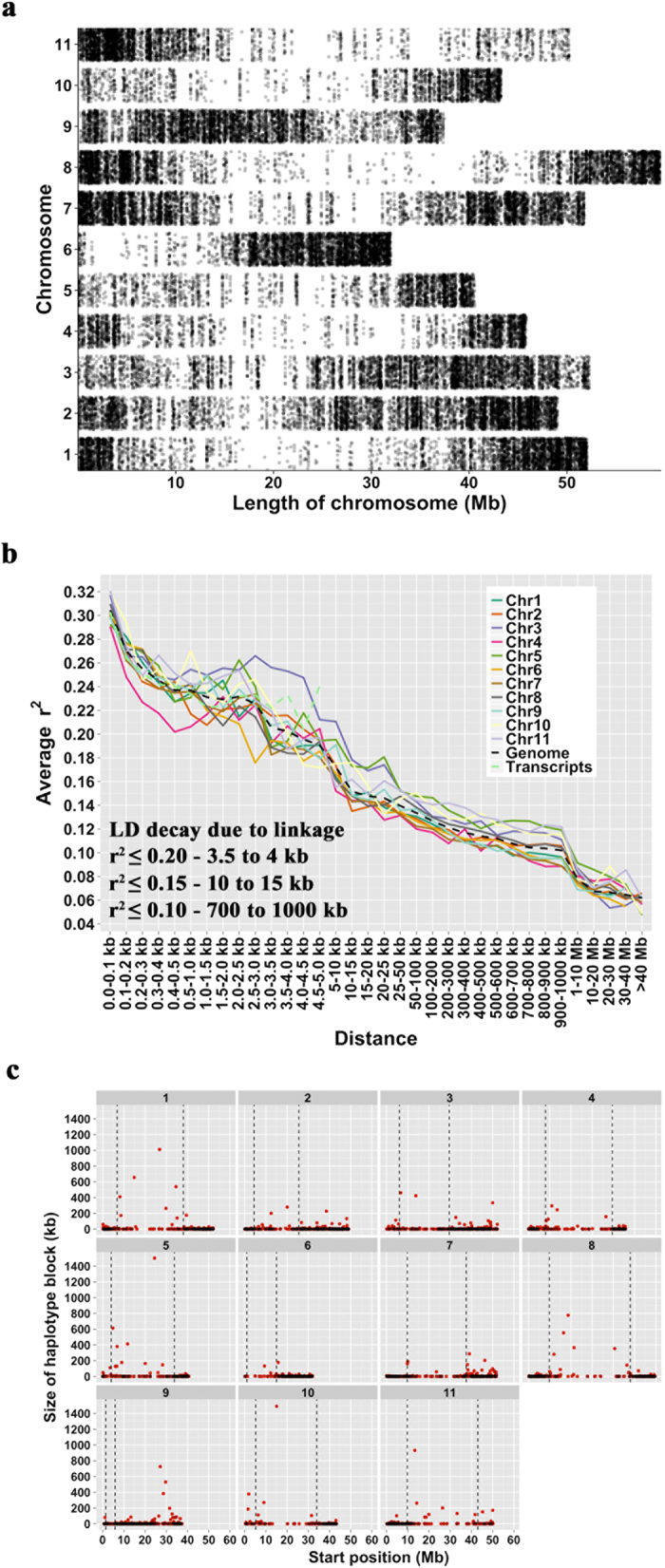
Genome-wide SNP distribution, linkage disequilibrium and haplotype blocks. (**a**) Distribution of SNPs identified in the Apios collection along the 11 *Phaseolus vulgaris* chromosomes. Apios transcripts were mapped to the *Phaseolus vulgaris* genome assembly (version 1.0), and location information was retrieved for 46,852 of the 58,154 SNP markers. (**b**) Decay of linkage disequilibrium along each of the putative chromosomes, across the genome, and transcripts. (**c**) Distribution of haplotype blocks along each of the chromosomes. The black dashed genotypes represent pericentromeric start and end coordinates of each of the chromosomes obtained from the *P. vulgaris* genome.

We investigated linkage disequilibrium by mapping the Apios transcripts to *P. vulgaris* chromosomes, under the assumption that the Apios and *P. vulgaris* chromosomes are generally syntenic–an assumption that we believe is warranted considering that most species in the Milletieae tribe have 11 chromosomes^29^,^30^, and various divergent species in the Phaseoleae have strongly collinear chromosomes^31^,^32^. We were able to place 46,852 of the 58,154 Apios SNP markers. These SNPs are distributed along each of the 11 chromosomes, with enrichment toward the chromosome ends (Fig. 5a). The enrichment at the chromosomal ends is undoubtedly because the SNPs are derived from transcripts, and the chromosome ends are gene rich^33^. The number of SNPs averaged 4,259 per chromosome (Supplementary Fig. 6). The decay of LD was investigated at different r^2^ thresholds, as well as along each chromosome, across the genome, and along the transcripts (Fig. 5b and Supplementary Table 8). On average, LD extends up to 10 to 15 kb (at r^2^ ≤ 0.15) across the genome. Although LD decays rapidly across the genome, it is well known that the extent of LD varies in different regions of the genome. Using a sliding window of 2 Mb along each chromosome, we identified 4,222 haplotype blocks (see Methods for details on haplotype block), averaging 7.4 kb in size. The distribution of haplotype blocks along each of the chromosomes indicated enrichment of large haplotype blocks in the pericentromeric regions–this result is unsurprising, because pericentromeric regions experience lower rates of recombination (Fig. 5c). The two largest haplotype blocks (~1,500 kb) were in pericentromeric regions on chromosomes 5 and 10. In summary, mapping of Apios SNPs to the *P. vulgaris* chromosomes provided putative location information for ~81% of the SNPs; and using these SNPs, LD was found to decay substantially within 10–15 kb.

### Marker-trait associations

We performed association analysis using five linear mixed models (see Methods), and implemented them in two packages. We inspected the performance of each of the mixed model by using quantile-quantile (QQ) plots. Based on the QQ plots, the most successful model (implemented in the GCTA package) accounted for population structure, using a familial relatedness matrix (Supplementary Fig. 7). For 19 of the 20 traits, it was apparent that incorporating the familial relatedness matrix in the mixed model analysis performed the best. For one of the traits (child tuber weight), two mixed models performed equally well. These two models accounted either for subpopulations and familial relatedness together, or just familial relatedness (Supplementary Fig. 7). Thus, for marker-trait associations, we proceeded with incorporating the familial relatedness to account for population structure, and the mixed model analysis performed in the GCTA package. This method identified twenty-one SNP markers to be associated with 14 phenotypic traits, including six aboveground and eight belowground traits (Table 3). Each marker associated with a trait originated from a different transcript. Additional details (favorable allele, allele frequency, effect size, and annotation of the transcript containing the SNP) are provided in Table 3. The marker-trait associations are also displayed in Manhattan plots (Supplementary Fig. 8). We also evaluated potential parental selections for the purpose of further cultivar improvement. We identified genotypes that contained beneficial alleles with large effect sizes for yield-related traits (Supplementary Table 9). Many of these genotypes were among the top 10% of the performers in field evaluations^6^.

### Marker-trait associations using gene expression markers (GEMs)

**Figure 6:**
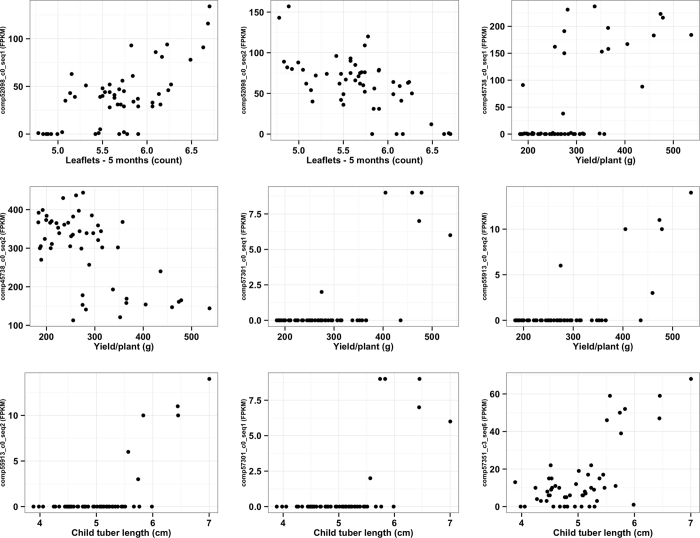
Scatter plots of nine interesting marker-trait associations identified using gene expression markers (GEMs). Linear regression analysis was performed with 39,609 GEMs as dependent variables, and each of the 20 phenotypic traits as independent variables. We identified 28 GEM-trait associations, nine of which are shown in this figure. The Y-axis of each plot represents FPKM normalized expression values of a transcript for 52 genotypes, and X-axis represents REML-based LS means for the phenotypic trait measured on these genotypes.

The reads from the leaf transcriptome from each genotype were mapped to 92,092 transcript assemblies out of the 96,560 transcript assemblies in the *de novo* reference assembly. A normalized expression dataset was generated from these mappings (Supplementary Data 11). The normalized expression dataset was further filtered for transcripts expressed (expression value of greater than or equal to 2) in at least one genotype, which yielded 39,609 transcripts, or GEMs across the collection (Supplementary Data 12). The regression analysis performed using GEMs, and filtered by applying Bonferroni correction (adj-*P* < 0.0000013), resulted in 34 GEM-trait associations (Supplementary Figs 9 and 10). Six of these GEM-trait associations were excluded because they violated the linearity assumption of linear regression analysis (Supplementary Figs 9 and 10). Finally, 28 GEM-trait associations were identified for four aboveground and five belowground traits (Table 4 and Supplementary Figs 11 and 12). Nine GEM-trait associations (Fig. 6) are particularly interesting, and can be broadly classified into three categories–(1) The expression of isoforms of the same gene are correlated with the same trait, but one of the isoforms is positively correlated with that trait while the other one is negatively correlated. This may suggest an autoregulatory feedback mechanism, or opposite roles in controlling the phenotype by these isoforms; (2) Transcripts are expressed only in the five highest-performing genotypes (excepting one outlier), whereas the rest of the genotypes showed no expression, suggesting the transcripts’ possible role in improved performance of the genotypes for the respective trait; and (3) A transcript has lower expression (~FPKM < 20) in genotypes that produce shorter child tubers, and has higher expression (~FPKM > 40) in genotypes that produce longer child tubers, suggesting the role of the transcript in regulating child tuber length. Overall, association analysis conducted using GEMs has revealed candidate genes for several traits.

## Discussion

Mining the diversity of plant collections is critical for developing cultivars. Evaluation of the breeding collection produced by Blackmon and Reynolds’ work has revealed Apios germplasm exhibiting significant improvements in key traits–for example, tuber yields of more than 1,500 g per plant for elite genotypes^6^. Nevertheless, despite ~10 years of phenotypic selection, statistical estimates of diversity (π and θ) indicate that significant nucleotide diversity remains in the collection. Thus, the Blackmon and Reynolds collection has potential for continued improvement and cultivar development.

Apios is clonally propagated through tubers similar to many tuber crops, such as potato, cassava, sweet potato, and yams. Tuber crops are often highly heterozygous and exhibit hybrid vigor^34^. An advantage of clonal propagation is that once a superior hybrid is identified, it can be fixed and propagated in the heterotic state. The downside is that high heterozygosity makes these crops vulnerable to inbreeding depression, since self-pollination will generally increase the proportion of homozygous (and presumably often deleterious) alleles. In this study, a strong negative correlation was observed between “inbreeding coefficients estimated for each genotype” and tubers produced per plant. Conversely, positive correlations were observed between inbreeding coefficients and leaflets measured 2 months after planting, mother tuber weight, and mother tuber width. The trait for leaflets recorded 2 months after planting is highly correlated with mother tuber weight and length, and higher values of these three phenotypic traits have previously been shown to be correlated with a “stout” tuber phenotype–i.e., one large mother tuber with just a few or no associated child tubers^6^. Thus, it seems that in Apios, higher heterozygosity is associated with genotypes that produce many child tubers (and generally higher yield) rather than a large mother/seed tuber; and lower heterozygosity is associated with genotypes that produce a large mother tuber/seed tuber at the expense of child tubers. These patterns may reflect hybrid vigor, with the heterotic state generally being higher-yielding and partitioning more biomass into many child tubers. If yield gains are associated with heterosis, then Apios may be susceptible to inbreeding depression when forced to self-pollinate or when crosses are made between genetically related genotypes.

Understanding the population structure is useful for effectively utilizing genotypes for breeding purposes. We find clear population structure in the collection, with approximately six genotypic clusters. Interestingly, classification on the basis of gene expression (using GEMs) was consistent with classification based on SNP genotyping: five of the six groups identified by fastSTRUCTURE remained intact in the phylogeny generated using GEMs. This validates the GEMs and suggests that gene expression data can be effectively used as molecular markers for understanding the population structure, and its accuracy may be comparable with SNP-based methods.

A clear understanding of pollination biology is essential for performing hybridization experiments. According to Bruneau and Anderson^11^, Apios flowers are predominantly out-crossing. Although self-pollination may occur when the plants are made to self, the success rate is quite low. Based on the Index of Self-Incompatibility (ISI), the authors suggest existence of partial self-incompatibility with characteristics of a gametophytic self-incompatibility system. We find LD in the Apios collection generally decaying within 10 to 15 kb. Linkage disequilibrium extending to such small distances is mainly observed in cross-pollinating species^35^,^36^. In self-pollinating species, the effective recombination rate is severely reduced, leading to increased LD. Hence, the LD results obtained in this study suggest out-crossing biology, consistent with observations made about the pollination by Bruneau and Anderson^11^.

Marker-trait associations identified using association analysis can be utilized in at least two ways: (1) implementation of marker-assisted selection (MAS) in a breeding program; (2) identification of potential parents containing favorable alleles (i.e. parental selections). The high heterozygosity and clear population structure in our dataset prompted us to test different models and packages for association analysis. Of the five models tested, the one that best accounted for population structure incorporated “familial relatedness” in the mixed model and was implemented in the GCTA package. This also appeared to give the most reliable results, based on QQ-plots. Controlling for population structure by incorporating “familial relatedness” has recently been shown to be helpful in association analysis involving highly structured populations with admixture^37^. One of the limitations of our study in performing association analysis is the relatively small population size (52 individuals). A similar population size (53 individuals) had been used in a study in *Brasssica napus*, and the marker-trait association identified and validated^38^. Nonetheless, marker-trait associations identified in this study should be considered cautiously, and will require validation using another germplasm collection or bi-parental populations grown in multiple environments.

SNP markers used in this study are from the transcribed regions, and this allows for preliminary verification of marker-trait associations using the annotations of the transcripts containing SNPs of interest. A few of the marker-trait associations for which there are suggestive transcript annotations are: (1) S_23737486 (SNP)/comp54771_c0_seq1 (transcript; a serine/threonine protein kinase), associated with “weeks to first leaf emergence.” This enzyme has been previously shown to regulate seedling germination^39^,^40^. (2) S_23095079/comp54598_c3_seq1 (Pectinesterase, a widely studied enzyme in fruits, and a key enzyme in potato involved in regulating firmness of the tubers^41^,^42^) associated with mother tuber width. (3) S_18970167/comp53339_c0_seq2 (a glucose-6-phosphate transmembrane transporter that catalyses the transfer of glucose-6-phosphate from one side of the membrane to the other), associated with mother tuber width. A study conducted in potato links the glucose-6-phosphate transmembrane transporter to tuber size. Sucrose is the major form of sugar transported to tubers from green tissues; unloading and subsequent mobilization of sucrose during tuber initiation and enlargement involves conversion of sucrose to fructose-6-phosphate through a glucose-6-phosphate intermediate^43^. Increased sucrose mobilization in the cytosol of the photosynthate sink has been shown to increase tuber number and reduce tuber size. On the other hand, a rise in sucrose mobilization in the apoplast increases tuber size and decreases tuber number^44^.

Selection of parents is the first key step in cultivar development. In our previous study^6^, we had recommended potential candidate parents and crossing schemes based only on phenotypic evaluations. The first scheme was suggested for development of high-yielding genotypes, and involved the utilization of the top 10% of high-yielding genotypes to make a “good x good” cross. The genotypes that comprised the top 10% in each of the four environments include accessions 1972, 2191, 898, 2127, 1849, 2155, 2201, 1970, and 2065^6^. Using the population structure results from this study, we can now identify genotypes that are the most genetically diverse, reducing the likelihood of inbreeding depression, and promoting hybrid vigor. We may now recommend hybridizations between the genotypes in genetically distinct clusters: cluster 1–2191; cluster 2: 1972, 1849, and 2155; cluster 3–1970; and cluster 4–898, 2127, 2201, and 2065. In addition, favorable alleles for yield-related traits are identified in the following genotypes that are among the top 10% based on their phenotype (Supplementary Table 9): Genotype 2191 (tubers/plant), 1972 (yield/plant, mother tuber length, child tuber weight and length), 1849 (yield/plant, mother tuber length), 2155 (yield/plant, tubers/plant, mother tuber length), 1970 (mother tuber length), and 898 (tubers/plant). For example, a cross of 1972 × 2191 or 1972 × 898 might be expected to produce some high-yielding genotypes with a reasonably good number of large sized child tubers. A similar strategy of integrating results from population structure and association analysis should be considered for other crossing schemes. In particular, it should be applied to the selections derived by Belamkar *et al*.^6^, which were primarily based on the phenotypic data available at the time.

Gene expression in the genotypes can be correlated with their phenotypes, and is valuable in identification of candidate genes for phenotypic traits^38^. Gene expression is affected by many factors including staging of tissues for sampling, environment, and plant growth and development. Nevertheless, recent evidence suggests expression data can be more powerful or at least as informative as the SNP markers for building kinship matrices and for clustering, PCA, expression-based association analysis and genomic predictions (especially for complex traits with low heritability)^38^,^45^,^46^,^47^,^48^. RNA-Seq and microarray can both provide insights into the global transcriptome. However, per sample costs are still higher compared to SNP genotyping assays. Hence, expression based marker-trait association studies have relied on sequencing one-pooled samples, comprising biological replicates^38^,^47^,^48^. In this study, 28 GEM-trait associations were identified, and they include transcripts for four aboveground and five belowground traits. Interestingly, only five of these transcripts contained SNPs. These valuable associations would not have been captured in the association analysis conducted solely using SNP markers. The expression of the transcript “comp57351_c3_seq6” was lower in genotypes with shorter child tubers, and was extremely high in genotypes with longer child tubers, resulting in clustering of the population into two groups based on its expression. This transcript is annotated as being involved in the Jasmonic Acid (JA) signaling pathway, and JA has a well established role as an effective inducer of tuberization in potato^49^,^50^. Two of the transcripts (comp57301_c0_seq1 and comp55913_c0_seq2), associated with yield/plant and child tuber length, were only expressed in the top five performing genotypes (1849, 1972, 2127, 2155 and 2201), and 1978 (good-performing, but not exceptional). These two transcripts were mapped to Chromosome 8 of *P. vulgaris*, where they are separated by 318.1 kb. Although there are no SNPs within either transcript, there are 96 SNPs identified in genes falling between them (assuming conserved synteny with Phaseolus). The average LD (r^2^) among these 96 SNPs was 0.18, which is slightly higher than the background LD value of 0.15. Further investigation is required to test whether this region was selected during the Blackmon and Reynolds’s breeding program. Overall, GEM-based association analysis provided several candidate genes that merit further functional evaluations. GEM-based associations will need to be validated in a different collection or in bi-parental mapping populations prior to implementation in gene discovery or marker-assisted selection efforts.

In summary, this study in Apios demonstrates how a combination of high-throughput genomics and phenotypic information may accelerate the mining of a germplasm collection for a little-studied crop. We have built a large collection of genomic resources for Apios, including a high-quality reference *de novo* transcriptome assembly, an expression atlas of six tissues, and 58,154 highly reproducible SNPs and 39,609 GEMs across the collection. Both SNPs and GEMs successfully identified the pedigree and population structure, and association analysis identified favorable alleles and potential candidate genes for both aboveground and belowground traits. Lastly, the success of GEMs here is exciting and suggests broad utility of this method for both new and well-studied crops.

## Methods

### Historical and morphological evaluation of the *Apios americana* collection

The collection used in the study was developed by Blackmon and Reynolds^14^,^15^ at Louisiana State University Agricultural Experiment Station in Baton Rouge, LA during 1985–1994 (Fig. 1a). The breeding program involved (i) collection of wild germplasm from different states of the USA and Canada with the majority of them originating from Louisiana; (ii) germplasm evaluation for traits of interest; (iii) open pollinations; and (iv) phenotype-based selections for superior genotypes (Fig. 1a). The open pollinations made it impossible to conclusively determine the paternal parent; instead, the genotypes growing adjacent to the female genotypes (the probable pollen donors), were generally recorded as the “likely paternal parents”. Genotypes that produced seeds were documented as female parents. Partial pedigree information derived from maternal lineage is available for 35 of the 53 accessions in the collection (Fig. 1b). The collection was screened^6^ for phenotypic variation in 2011–2012 in Iowa, Virginia and Pennsylvania under field-conditions and in pots and grow-bags. Twenty phenotypic traits, including 10 aboveground and 10 belowground traits, were recorded for the entire collection. The REML-based least square (LS) means generated for the 20 traits recorded at Ames, IA during 2011 and 2012 were used in this study^6^. The genome sizes of 25 of the 53 genotypes, as well as six additional wild accessions collected from Iowa, New York and Canada were estimated using flow cytometry at the Iowa State University Flow Cytometry Facility (http://www.biotech.iastate.edu/facilities/flow/).

### *De novo* transcriptome assembly, annotation and expression catalog

Total RNA was isolated from 14 samples, including six different tissues from accession 2127 (Supplementary Data 1), using Qiagen RNeasy^®^ Plant mini kit and following the manufacturer’s protocol. The RNA samples were treated with Ambion^®^ TURBO DNA-free™ DNase to eliminate any DNA contamination, and the quality and quantity were then inspected using an Agilent 2100 Bioanalyzer. Illumina^®^ libraries were prepared, and the samples were sequenced on Illumina^®^ GAIIx, and Illumina^®^ HiSeq 2000 platforms (Supplementary Data 1). Both single- and paired-end reads of length 50 to 90 bp were generated. The reads were trimmed to exclude the Q2 (read segment control indicator) bases from the ends of the reads using a custom script (https://goo.gl/Q73j48). The reads that passed the quality trimming and were at least 25 bp in length were utilized to build the *de novo* transcriptome assembly using the Trinity package^51^ (release 2013-02-25). Assembled transcripts were examined for full-length transcripts, and sequence conservation across species, by performing a BLASTX search with a threshold of 1E-05 against the proteomes of six sequenced legumes, Swiss-Prot non-redundant database (release-2013_04), and the model plant *Arabidopsis thaliana* (v 10.0; release 04/16/2012). The six legume species included *Glycine max* (assembly v 1.01, JGI Glyma 1.1 annotation), *Medicago truncatula* (v 3.5.1), *Cajanus cajan* (v 1.0), *Phaseolus vulgaris* (v 1.0), *Lotus japonicus* (v 2.5) and *Cicer arietinum* (v 1.0). Functional annotation of transcripts was performed using Trinotate^51^, an annotation suite within the Trinity package. The likely coding region (open reading frame) in the transcripts were extracted using TransDecoder with default settings, and the option to utilize pfam using hmmscan (–search_pfam), and the minimum peptide length changed to 67 amino acids to match with the minimum length of transcripts. The functional annotation of these peptides was performed as follows: a homology search with BLASTP against the Swiss-Prot non-redundant database; protein domain identification using HMMER and Pfam-A (v 27.0); signal peptide identification using SignalP (v 4.0); transmembrane region prediction using tmHMM; and annotation by comparison with the eggNog (evolutionary genealogy of genes) and Gene Ontology databases. Lastly, the quality-trimmed reads from 11 samples–six different tissues and two biological replicates per tissue (except for flower sample)–were aligned to the *de novo* transcriptome assembly using Bowtie, and the abundance estimation per transcript and component (loosely termed as gene) were estimated using RNA-Seq by Expectation Maximization (RSEM)^52^.

### Genotyping using RNA-Seq

The collection was grown in 2012 at Ames, IA as described in Belamkar *et al*.^6^. Young leaf close to the shoot tip was excised from 52 genotypes, 3 months after planting, on a single day, within a two-hour window and at mid-day to minimize the effect of environment and provide relatively uniform staging of tissues for gene expression. The leaf tissue from four plants that were grown from four independent seed tubers (represent a genotype) were pooled and frozen in liquid nitrogen. Total RNA was isolated and the quality was inspected as described earlier for *de novo* transcriptome assembly construction. Illumina^®^ libraries were prepared at the DNA Facility at Iowa State University (http://www.dna.iastate.edu), and the samples were sequenced on Illumina^®^ HiSeq at National Center for Genome Resources (NCGR), Santa Fe, NM to generate single-end, 50 bp reads (Supplementary Data 1). RNA was isolated and sequenced from pooled samples of shoots and roots of four genotypes that were grown in the greenhouse for quality control purposes (Supplementary Data 1). All of the sequence data generated are available in the Short Read Archive (SRA) at NCBI (BioProject: PRJNA313331; SRA: SRP071120). The reads from the 56 samples were mapped to the *de novo* transcriptome assembly to identify “variants” and “transcript abundances for each genotype” using the Alpheus^TM^ pipeline^53^. For calling single nucleotide polymorphisms (SNPs) in each genotype, a minimum read depth of ≥5 reads, frequency of variant allele ≥20%, and average quality of bases calling the variant allele ≥10 were used. We further filtered and excluded SNP markers with minor allele frequency ≤0.1% and maximum missing percentage ≥10%, and generated 58,154 high-quality SNP markers. Allele frequencies, and summary statistics for each SNP (major and minor allele frequency, missing %, heterozygous accessions %) and genotype (number of SNPs, missing SNPs %, heterozygous SNP marker sites %) were generated using the “Genotype summary” option in TASSEL v 5.0 (ref. 54). The “transcript abundance per individual” was also utilized as a marker, and will be referred to as a gene expression marker (GEM). A transcript was considered expressed if the normalized expression value was greater than or equal to 2 in at least one of the genotypes, which resulted in 39,609 GEMs across the collection.

### Diversity, inbreeding and pedigree estimates

The two commonly used diversity estimates, average pairwise divergence or nucleotide diversity per bp, π (pi), and number of segregating sites per nucleotide, θ (theta) were generated using the option “Diversity” in TASSEL. Similarly, the Tajima’s D was produced in TASSEL to understand the evolutionary history of the breeding collection. The extent of heterozygosity in the collection, and its effect on phenotype, was investigated by estimating inbreeding coefficients for each genotype using the “–het” option in PLINK. The correlations between inbreeding coefficients and REML-based LS means of phenotypic traits recorded in Ames, IA in 2011–2012 were performed in R (http://www.r-project.org/). Lastly, pedigree relationships between genotypes in the collection such as “parent-child” and “half-sib” were identified using estimates of identity-by-descent (IBD) as described in Stevens *et al*.^55^. The IBD and the proportion of IBD values were generated using the “–genome” option in PLINK with an assumption of homogenous population. The SNP data set used for IBD analysis was restricted to retain only the SNPs that were reproducible in the four control genotypes, and had minor allele frequency ≥0.1% and maximum missing percentages ≤10%, which provided 14,321 SNP markers across the collection.

### Population structure

The population structure of the collection was investigated using both SNPs and GEMs. Four approaches were tested using SNPs–(1) Phylogeny reconstruction using the package SNPhylo^56^. Briefly, in this package the SNP information of each genotype is used to generate sequences. The sequences are then aligned using MUSCLE, and the phylogeny tree is built using maximum likelihood. SNPhylo was utilized with the following settings (-l 0.0, -b and -A), and the resulting tree was visualized with midpoint rooting using FigTree v 1.4.2; (2) Identity-by-state (IBS)–a distance matrix based on (1-IBS) values was generated using PLINK v 1.90b2pNL, and hierarchical clustering was performed using the Ward’s linkage in R; (3) Principal component analysis (PCA)–performed in the program GCTA v 1.24 (ref. 57), and the plot of PC1 versus PC2 was made in R; (4) Variational Bayesian framework–implemented in the program fastSTRUCTURE^58^. The program fastSTRUCTURE was run with a prior of 1 to 10 subgroups in the collection (K = 1 to 10), and the output was parsed with “choosing model complexity” script to determine the possible range of subgroups. The potential subgroups identified were then inspected with known pedigree information and coefficient of coancestry values generated for each genotype to precisely identify the number of subgroups in the collection. The results were visualized using a plot made in R. Furthermore population structure analysis was also performed using GEMs. The normalized expression counts of 39,609 GEMs were transformed to log_2_ scale, and 1000 GEMs with the highest variances across the collection were utilized to generate a Euclidean distance matrix followed by hierarchical clustering using Ward’s linkage method in R. A confusion table was built to compare the performance of the five different approaches to the known partial pedigree information, and decipher the population structure in the collection.

### Linkage disequilibrium (LD), and LD decay

Linkage disequilibrium in the collection was investigated using the LD statistic “r^2^.” The r^2^ values were generated for all marker pairs located within and between transcripts using the options “–r2, inter-chr and –ld-window-r2 0.0” in PLINK. We further mapped the Apios transcripts to the *Phaseolus vulgaris* genome assembly (version 1.0) using gmap (2014-05-15.v3) with the following settings: “–cross-species, –format = coords, –npaths = 0, –chimera-margin = 0, intronlength = 10034, –totallength = 60969,” and retrieved the likely location information for ~81% of the SNPs. These marker locations were then utilized to investigate the decay of LD along each of the “pseudo-Apios” chromosomes, as well as across the genome, with the assumption that the genome structure is conserved between the two phaseoloid legumes *A. americana* and *P. vulgaris*. The background LD estimated as the 90^th^ percentile of the r^2^ value of marker-pairs on different chromosomes, and the commonly used criteria (r^2^ = 0.1 and 0.2) across the genome, were used to set a threshold to determine the LD decay. Haplotype blocks were estimated using the options “–blocks, no-pheno-req and –blocks-max-kb 2000” in PLINK. The maximum haplotype block size was set to 2 Mb. In this method two SNPs are considered to be in ‘strong LD’ if the lower limit of the 90% D-prime confidence interval is greater than 0.7, and the upper limit of the confidence interval is at least 0.98. Generally, for a haplotype block the number of ‘strong LD pairs’ must be more than 95% of the total number of informative pairs. The pericentromeric start and end coordinates of each of the chromosomes were obtained from the *P. vulgaris* genome browser hosted at the Legume Information System (legumeinfo.org).

### Association analysis using SNP markers

The phenotypic dataset used for association analysis is previously reported in Belamkar *et al*.^6^, and contains REML-based LS means of 20 phenotypic traits that include 10 aboveground and 10 belowground measurements recorded on 52 genotypes in Ames, IA during 2011 and 2012. The genotypic dataset contains 58,154 SNPs of high quality, after filtering and excluding SNPs with minor allele frequency ≤0.1% and maximum missing percentage ≥10%. Population structure was accounted for either by including only familial relatedness, or familial relatedness together with subpopulations in the linear mixed models (LMMs). Association analysis was first performed in the software program TASSEL^59^ by incorporating (1) familial relatedness matrix (generated in PLINK) in the linear mixed model as random effect; and (2) familial relatedness (as random effect) and subpopulation membership coefficients generated using fastSTRUCTURE as covariates. Association analysis was also conducted using “GCTA: a tool for Genome-wide Complex Trait Analysis^60^”, in which familial relatedness matrix was generated with GCTA, and either six or 10 principal components were used to account for presence of subpopulations. The performance of each of the models was tested using quantile-quantile (QQ) plots generated using “qqman” package in R. Marker-trait associations with a *P*-value less than 0.0001 were considered as significant associations^38^,^61^, and were tabulated and represented in Manhattan plots generated using “qqman” package in R. The marker-trait associations were further assessed by comparing the biological function of the transcript containing the SNP marker (derived from its annotation) to the associated phenotypic trait.

### Association analysis using gene expression markers

Linear regression analysis was performed in R with 39,609 GEMs as dependent variables, and each of the 20 phenotypic traits as independent variables. The adjusted-r^2^ and significance (*P*) values were recorded per trait for each of the GEMs. The associations were: (1) filtered by applying Bonferroni correction of *P* < 0.05, which resulted in a new threshold of *P* < 0.0000013 (0.05/39609). (2) The associations that passed the Bonferroni test were examined for assumptions of linear regression. FPKM normalized expression values were represented along Y-axis, and REML-based LS means for the phenotypic trait were plotted along the X-axis. The significant associations that passed both the Bonferroni test and met the assumptions were tabulated, and also displayed using Manhattan plots. The significant associations were further verified by comparing the associated phenotypic trait with the annotations of transcripts.

### Statistical analyses

The statistical analyses described in this article were performed in R (http://www.r-project.org/). R code for replicating the analysis and plots are provided in Supplementary Methods 1.

## Additional Information

**How to cite this article**: Belamkar, V. *et al*. Genomics-assisted characterization of a breeding collection of *Apios americana*, an edible tuberous legume. *Sci. Rep*. **6**, 34908; doi: 10.1038/srep34908 (2016).

## Supplementary Material

Supplementary Tables

Supplementary Figures

Supplementary Methods

Supplementary Data S1

Supplementary Data S2

Supplementary Data S3

Supplementary Data S4

Supplementary Data S5

Supplementary Data S6

Supplementary Data S7

Supplementary Data S7A

Supplementary Data S8

Supplementary Data S9

Supplementary Data S10

Supplementary Data S11

Supplementary Data S11A

Supplementary Data S12

## Figures and Tables

**Table 1:** Statistics of the *Apios americana* transcriptome assembly built using accession 2127.

Metric	Count
Sequencing reads	
Total number of reads	210,018,551
Total number of nucleotides (Gbp)	11.7
Total number of nucleotides used (Gbp)	11.5
Total number of nucleotides unused (Gbp)	0.2
*De novo* transcriptome assembly	
Number of putative transcripts	96,560
Number of components (generally correspond with genes)	48,615
Number of components with splice variants	11,215
N50	1,863
Total number of nucleotides in the assembly (bp)	113,238,654
Peptides	
Number of peptides (alternative splice variants included)	60,880
Number of peptides (alternative splice variants excluded)	23,691
Annotation of peptides (alternative splice variants excluded)	
Swiss-Prot	16,711
Pfam-A	17,087
Signal peptide	2,240
Transmembrane helices	5,847
eggNOG (evolutionary genealogy of genes)	13,524
Gene Ontology	15,647
Expression	
Number of transcripts expressed in at least one tissue	56,735
Number of components expressed in at least one tissue	28,738

**Table 2:** Discovery of variants across 52 genotypes in the collection.

Metric	Count
Number of accessions in the Apios collection	52
Total number of reads generated	1,315,730,442
Average number of reads generated per accession	25,302,509
Average % of reads mapped to the assembly per accession	89.5
Average % of reads mapped uniquely to the assembly per accession	61.4
Number of variants (SNP and Indels) identified with base criteria	1,582,730
Number of variants identified (base criteria + read depth ≥ 5)	299,145
Number of SNPs identified (base criteria + read depth ≥ 5)	271,170
Number of SNPs (base criteria + read depth ≥ 5 + MAF ≥ 0.1 + Maximum missing percentage ≤ 10%)	58,154
Average % reproducibility of SNPs (tested using 4 biological replicates)	90.6
Number of components (a.k.a genes) harboring SNPs	9,338
% of components in the assembly harboring SNPs	19.2
Average accessions missing per SNP (%)	1.5
Average heterozygous accessions per SNP (%)	37.5
Number of gene expression markers identified	39,609

Base criteria: At least 2 reads calling variant within at least 1 accession; ≥20% of the reads calling the variant allele in that sample; and average quality of bases calling the variant is ≥10).

**Table 3:** Marker-trait associations identified in the collection using SNP-based association analysis.

Trait	SNP	Reference transcript	Position	Chr^1^	Position^2^	Alleles^3^	Freq^4^	Effect^5^	SE^6^	*P*-value	Annotation^7^
First leaf emergence	S_23737486	comp54771_c0_seq1	1,673	04	43,022,415	A/G	0.23	0.16	0.04	8.71E-05	Serine/threonine protein kinase
Ground to first leaf	S_4036060	comp43747_c0_seq1	1,279	NA^8^	NA	C/T	0.21	1.35	0.33	3.84E-05	Succinyl-CoA ligase [ADP-forming] subunit alpha-2
Ground to first leaf	S_8354256	comp48670_c0_seq1	510	09	21,384,837	T/C	0.18	1.65	0.39	2.12E-05	Ubiquitin-fold modifier-conjugating enzyme
Ground to first leaf	S_12390026	comp50830_c0_seq2	355	01	269,531	T/C	0.16	1.39	0.35	5.47E-05	Glutathione S-transferase
Ground to first leaf	S_17227020	comp52764_c0_seq3	354	NA	NA	C/T	0.12	1.32	0.34	9.52E-05	PAP2 superfamily
Leaflets-2 months	S_11450514	comp50326_c0_seq1	552	10	43,192,696	A/T	0.50	-0.44	0.11	7.39E-05	StAR-related lipid transfer protein
Plant vigor	S_23474932	comp54688_c0_seq2	1,435	03	30,967,083	G/A	0.15	0.39	0.09	3.26E-05	NA
Stem diameter-5 months	S_30601377	comp56353_c0_seq1	1,880	02	47,724,772	A/T	0.33	-0.35	0.09	9.09E-05	Synaptotagmin-5
SPAD	S_31213411	comp56475_c0_seq1	2,559	05	14,781,013	G/T	0.46	0.97	0.25	9.82E-05	Nuclear pore complex protein Nup98-Nup96
Yield/plant	S_806532	comp29700_c0_seq1	260	NA	NA	T/C	0.20	97.65	23.40	3.02E-05	Glutaredoxin-C2
Tubers/plant	S_25048846	comp55124_c0_seq4	465	05	9,906,844	T/C	0.46	-9.91	2.54	9.73E-05	NA
Tubers/plant	S_41908941	comp58209_c0_seq1	117	01	50,369,688	T/A	0.48	-14.07	3.23	1.32E-05	Clathrin heavy chain 2; endocytosis
Stolon length	S_7515611	comp48154_c1_seq1	805	05	43,471	T/C	0.27	-9.89	2.54	9.78E-05	Deoxynucleoside kinases
Stolon length	S_14498332	comp51702_c0_seq1	1,505	03	40,709,216	G/A	0.24	-10.22	2.55	6.35E-05	RNA processing and splicing; WW domain-binding protein
Stolon length	S_17413318	comp52828_c0_seq1	183	NA	NA	T/A	0.26	-9.05	2.19	3.71E-05	NA
Mother tuber weight	S_18970167	comp53339_c0_seq2	904	08	57,655,132	T/C	0.48	-85.81	20.86	3.88E-05	Glucose-6-phosphate transmembrane transporter
Mother tuber length	S_25933856	comp55332_c0_seq1	1,714	02	499,012	G/A	0.38	0.79	0.20	6.22E-05	Regulator of nonsense transcripts
Mother tuber width	S_23095079	comp54598_c3_seq1	295	10	39,313,260	C/T	0.19	-0.90	0.23	9.28E-05	Pectinesterase
Child tuber weight	**S_12121278**	comp50661_c0_seq1	1,089	02	45,978,408	A/C	0.12	15.30	3.59	2.00E-05	PC-Esterase GDSL/SGNH-like Acyl-Esterase
Child tuber weight	S_28587045	comp55916_c1_seq1	1,482	08	7,701,745	T/A	0.15	11.83	3.03	9.55E-05	BTB/POZ domain-containing protein; signal transduction
Child tuber length	**S_12121278**	comp50661_c0_seq1	1,089	02	45,978,408	A/C	0.12	0.96	0.24	6.62E-05	PC-Esterase GDSL/SGNH-like Acyl-Esterase
Child tuber length	S_30406303	comp56304_c4_seq2	1,682	06	29,252,859	G/A	0.13	0.74	0.19	7.84E-05	Zinc finger CCCH; DNA binding; photomorphogenesis

^1^Chr represents chromosomal location of SNP on *Phaseolus vulgaris* genome.

^2^Position represents location of SNP on *Phaseolus vulgaris* genome.

^3^Effective allele/other allele at the SNP location.

^4^Frequency of effective allele in the collection.

^5^SNP effect corresponding to the effective allele.

^6^Standard error associated with the SNP effect.

^7^Annotation of reference transcript containing the SNP marker.

^8^NA represents information not available. SNPs associated with multiple traits are in bold.

**Table 4:** Marker-trait associations identified in the collection using gene expression markers.

Trait	Transcript	Chr^1^	Start^2^	Estimate^3^	SE^4^	t-value^5^	*P*-value	Adj-r^2^	Annotations
Internode length	comp56323_c0_seq5	NA^6^	NA	−2.74	0.46	−5.99	2.26E-07	0.41	NA
Stem diameter–2 months	**comp57243_c2_seq2**	02	3,455,626	73.48	13.0	5.65	7.57E-07	0.38	RNA-binding protein 8A; mRNA processing
	comp52044_c2_seq3	10	42,053,143	−13.38	2.35	−5.69	6.68E-07	0.38	Heat shock 70 kDa protein 15/Molecular chaperone
Leaflets–2 months	comp49135_c0_seq1	10	6,001,220	11.10	1.56	7.10	4.24E-09	0.49	CER1-like 1/Fatty acid hydroxylase
	comp55774_c3_seq6	05	34,676,406	−4.11	0.72	−5.72	5.92E-07	0.38	NA
Leaflets–5 months	comp52098_c0_seq1	02	33,390,379	45.40	6.89	6.59	2.59E-08	0.45	Geranylgeranyl pyrophosphate synthase
	comp52098_c0_seq2	02	33,390,379	−45.56	7.62	−5.98	2.35E-07	0.41	Geranylgeranyl pyrophosphate synthase
Yield/plant	**comp57301_c0_seq1**	08	51,938,872	0.02	0.00	6.96	6.96E-09	0.48	GDSL esterase/lipase At1g71250; Geranylgeranyl reductase, choloroplastic
	**comp55913_c0_seq2**	08	52,258,272	0.03	0.00	6.70	1.80E-08	0.46	Integral membrane protein; Late exocytosis, Golgi transport
	comp49849_c0_seq1	05	30,357,198	0.04	0.01	6.48	3.93E-08	0.45	NA; Possesses a signal peptide cleavage site
	comp45738_c0_seq1	05	28,501,950	0.64	0.11	6.11	1.50E-07	0.42	14-3-3-like protein B
	comp32096_c0_seq1	05	20,713,186	−0.03	0.01	-5.76	5.17E-07	0.39	Uncharacterized protein At1g18480; Calcineurin-like phosphoesterase
	**comp55939_c2_seq5**	03	23,432,180	0.01	0.00	5.73	5.83E-07	0.38	UDP-glucose flavonoid 3-O-glucosyltransferase 7
	comp45738_c0_seq2	05	28,501,950	−0.69	0.12	−5.57	1.01E-06	0.37	14-3-3-like protein B
Tubers/plant	comp52843_c0_seq8	07	38,262,470	0.46	0.07	6.25	8.83E-08	0.43	ADP-ribosylation factor GTPase-activating protein AGD7
	comp57897_c0_seq2	08	4,984,724	0.60	0.11	5.66	7.42E-07	0.38	N-terminal kinase-like protein
	comp53873_c0_seq2	08	9,973,071	0.40	0.07	5.58	9.71E-07	0.37	Ferrochelatase-2, chloroplastic
	comp46887_c0_seq3	09	21,886,278	0.24	0.04	5.58	9.94E-07	0.37	NA
	comp54966_c0_seq3	11	8,691,266	−0.94	0.17	−5.56	1.06E-06	0.37	Nucleoside-diphosphate-sugar epimerases; UDP-glucuronic acid oxidase
Mother tuber length	comp57964_c0_seq4	07	41,689,316	−4.11	0.68	−6.07	1.70E-07	0.41	Mannosylglycoprotein endo-beta-mannosidase; Carbohydrate metabolism
Child tuber weight	**comp55913_c0_seq2**	08	52,258,272	0.21	0.04	5.75	5.34E-07	0.39	Integral membrane protein; Late exocytosis, Golgi transport
Child tuber length	**comp55913_c0_seq2**	08	52,258,272	3.41	0.50	6.79	1.26E-08	0.47	Integral membrane protein; Late exocytosis, Golgi transport
	comp57351_c3_seq6	02	47,947,761	19.12	2.83	6.76	1.42E-08	0.47	Jasmonate ZIM domain-containing protein 3; JA signaling pathway
	**comp55939_c2_seq5**	03	23,432,180	1.72	0.29	5.93	2.80E-07	0.40	UDP-glucose flavonoid 3-O-glucosyltransferase 7
	comp56129_c4_seq6	NA	NA	8.05	1.36	5.92	2.87E-07	0.40	NA
	comp56118_c0_seq1	02	43,809,153	2.12	0.37	5.72	6.02E-07	0.38	NA
	**comp57301_c0_seq1**	08	51,938,872	2.38	0.42	5.71	6.13E-07	0.38	GDSL esterase/lipase At1g71250; Geranylgeranyl reductase, choloroplastic
	**comp57243_c2_seq2**	02	3,455,626	31.05	5.45	5.70	6.40E-07	0.38	RNA-binding protein 8A; mRNA processing

^1^Chr represents chromosomal location of transcript on *Phaseolus vulgaris* genome.

^2^Start represents start position of the transcript mapped to *Phaseolus vulgaris* genome.

^3^Estimate obtained from linear regression analysis.

^4^Standard error associated with the estimate.

^5^t-value is from a test with null hypothesis that the estimate is equal to zero (no effect).

^6^NA represents information not available. Transcripts associated with multiple traits are in bold.
