# Supplementary material for: Genomics-assisted characterization of a breeding collection of Apios americana, an edible tuberous legume
Source: Sci Rep. 2016 Oct 10;6:34908. doi: 10.1038/srep34908 (PMC5056515; doi:10.1038/srep34908)
Supplement: Supplementary Tables [file srep34908-s1.doc]

**Title page for Supplementary Tables 1-9, associated with the following paper:**

**Genomics-assisted characterization of a breeding collection of *Apios americana*, an edible tuberous legume**

Vikas Belamkar, Andrew D. Farmer, Nathan T. Weeks, Scott R. Kalberer, William J. Blackmon, Steven B. Cannon

**Supplementary Table 1.** **Genome size estimates of 25 genotypes from Blackmon-Reynolds collection and wild samples estimated using flow cytometry**

| **Run** | **Sample No.** | **Apios#** | **Genome Size (pg) (1C)** | **Genome Size (1C) (Mb)** |
| --- | --- | --- | --- | --- |
| 1 | 1 | 807 | 1.69 | 1656 |
| 1 | 2 | 1372 | 1.64 | 1601 |
| 1 | 3 | 1710 | 1.67 | 1636 |
| 1 | 4 | 1718 | 1.75 | 1710 |
| 1 | 5 | 1846 | 1.68 | 1647 |
| 1 | 6 | 1908 | 1.68 | 1648 |
| 1 | 7 | 1916 | 1.66 | 1627 |
| 1 | 8 | 1943 | 1.71 | 1677 |
| 1 | 9 | 1972 | 1.74 | 1700 |
| 1 | 10 | 2003 | 1.69 | 1655 |
| 1 | 11 | 2012 | 1.65 | 1614 |
| 1 | 12 | 2030 | 1.70 | 1662 |
| 1 | 13 | 2039 | 1.65 | 1617 |
| 1 | 14 | 2065 | 1.62 | 1584 |
| 1 | 15 | 2109 | 1.74 | 1697 |
| 1 | 16 | 2110 | 1.66 | 1622 |
| 1 | 17 | 2121 | 1.69 | 1658 |
| 1 | 18 | 2161 | 1.65 | 1618 |
| 1 | 19 | 2175 | 1.62 | 1585 |
| 1 | 20 | 2179 | 1.71 | 1668 |
| 1 | 21 | 2185 | 1.73 | 1688 |
| 1 | 22 | 2192 | 1.68 | 1645 |
| 1 | 23 | 2201 | 1.67 | 1632 |
| 1 | 24 | 2210 | 1.69 | 1657 |
| 1 | 25 | 2219 | 1.64 | 1608 |
|  |  | **Mean** | **1.68** | **1644** |
|  |  | **Standard deviation (SD)** | **0.04** | **34.38** |
|  |  |  |  |  |
| **Run** | **Sample No.** | **Apios#** | **Genome Size (pg)** | **Genome Size (Mb)** |
| 2 | 1 | 2127 | 1.53 | 1499 |
| 2 | 2 | Ledges Sand/Cliff, IA | 2.41 | 2356 |
| 2 | 3 | Ledges Below Cliff, IA | 2.46 | 2408 |
| 2 | 4 | Hamilton, IA | 2.46 | 2407 |
| 2 | 5 | Boone, IA | 2.45 | 2397 |
| 2 | 6 | Cornell, Ithaca 1677-1, NY | 2.40 | 2345 |
| 2 | 7 | Quebec, Canada | 2.42 | 2365 |
|  |  | **Mean (Except 2127)** | **2.43** | **2380** |
|  |  | **Standard Deviation (SD)** | **0.03** | **27.59** |

Soybean leaf samples were used as controls, and the genome size is 1,115 Mb.

1pg = 78Mb. Hence, for soybean genome 1C=1.14 pg.

**Supplementary Table 2. Summary of RNA-Seq data and expression in different tissues of genotype 2127**

| **Tissue - Replicate** | **Number of reads (Million)** | **Number of reads used after quality trimming (Million)** | **% of reads mapped to assembly** | **Number of transcripts expressed** | **% of transcripts expressed** | **Number of components expressed** | **% of components expressed** |
| --- | --- | --- | --- | --- | --- | --- | --- |
| Shoot - 1 | 13.1 | 12.9 | 87.8 | 29,328 | 30.4 | 17,934 | 36.9 |
| Shoot - 2 | 13.8 | 13.5 | 88.1 | 29,228 | 30.3 | 18,076 | 37.2 |
| Leaf - 1 | 15.4 | 15.2 | 91.4 | 26,396 | 27.3 | 16,658 | 34.3 |
| Leaf - 2 | 13.1 | 12.9 | 90.5 | 25,657 | 26.6 | 16,873 | 34.7 |
| Root - 1 | 12.0 | 11.7 | 90.0 | 25,224 | 26.1 | 16,597 | 34.1 |
| Root - 2 | 13.0 | 12.7 | 89.3 | 28,071 | 29.1 | 17,156 | 35.3 |
| Mother tuber - 1 | 11.2 | 11.0 | 89.7 | 24,827 | 25.7 | 15,725 | 32.3 |
| Mother tuber - 2 | 14.9 | 14.6 | 89.9 | 25,066 | 26.0 | 16,207 | 33.3 |
| Child tuber - 1 | 16.0 | 15.8 | 89.4 | 26,175 | 27.1 | 16,424 | 33.8 |
| Child tuber - 2 | 16.4 | 16.1 | 90.7 | 24,135 | 25.0 | 15,616 | 32.1 |
| Flower -1 | 24.0 | 23.9 | 95.0 | 32,147 | 33.3 | 19,461 | 40.0 |

**Supplementary Table 3. Validation of single nucleotide polymorphisms (SNPs) using biological replicates**

| **Accessions versus accession replicate** | **Number of common SNP sites between the two samples** | **Number of identical SNPs between the two samples** | **% of identical SNPs between the two samples** |
| --- | --- | --- | --- |
| 784 vs. 784 Rep | 35,158 | 31,612 | 89.9 |
| 807 vs. 807 Rep | 40,686 | 37,076 | 91.1 |
| 1849 vs. 1849 Rep | 24,819 | 22,147 | 89.2 |
| 2003 vs. 2003 Rep | 40,469 | 37,271 | 92.1 |
|  |  | **Average** | **90.6** |

Note: Filtering was done to exclude SNPs with minor allele frequency ≤ 0.1% and maximum missing genotype ≥ 10% before generating above statistics.

**Supplementary Table 4. Frequency of alleles in the SNP dataset used in this study**

| **Alleles** | **Genotype** | **Number** | **Frequency** |
| --- | --- | --- | --- |
| C | C | 499,646 | 0.17 |
| G | G | 485,228 | 0.16 |
| A | A | 438,369 | 0.15 |
| T | T | 435,494 | 0.15 |
| Y | C:T | 342,314 | 0.11 |
| R | A:G | 334,179 | 0.11 |
| W | A:T | 130,091 | 0.04 |
| M | A:C | 111,845 | 0.04 |
| K | G:T | 108,063 | 0.04 |
| S | C:G | 92,732 | 0.03 |
| N | Missing | 46,047 | 0.02 |

Dataset was filtered to exclude SNP makers with minor allele frequency ≤ 0.1 % and maximum missing percentage ≥ 10%.

**Supplementary Table 5. Correlations between inbreeding coefficients estimated for each genotype and phenotypic measurements recorded in Ames, IA in 2011-2012**

| **Traits** | **Correlation coefficients** | ***P*-values** |
| --- | --- | --- |
| Emergence time, week | 0.09 | 0.5464 |
| First leaf emergence, week | 0.09 | 0.5462 |
| Ground to first leaf, cm | 0.1 | 0.4682 |
| Internode length, cm | 0.14 | 0.3083 |
| Stem diameter - 2 months, mm | 0.05 | 0.6999 |
| Leaflets - 2 months, count | **0.37** | **0.0071** |
| Plant vigor, units | -0.04 | 0.7974 |
| Leaflets - 5 months, count | 0.28 | 0.0479 |
| Stem diameter - 5 months, mm | 0.03 | 0.8536 |
| SPAD | -0.14 | 0.3378 |
| Yield/plant, g | -0.13 | 0.3617 |
| Tubers/plant, count | **-0.47** | **0.0004** |
| Tuber-to-tuber distance, cm | 0.13 | 0.3455 |
| Stolon length, cm | -0.05 | 0.7097 |
| Mother tuber weight, g | **0.36** | **0.0089** |
| Mother tuber length, cm | 0.25 | 0.0753 |
| Mother tuber width, cm | **0.32** | **0.019** |
| Child tuber weight, g | 0.03 | 0.8348 |
| Child tuber length, cm | -0.03 | 0.8165 |
| Child tuber width, cm | 0.11 | 0.4552 |

Statistically significant (*P* < 0.05) correlations are in bold. SPAD, soil plant analysis development.

**Supplementary Table 6. Parent-child, and half-sib relationships (0.46>=PI_HAT<=0.54) identified in the Apios collection**

| **S. No.** | **Genotype-pairs^1^** | | **Z0^2^** | **Z1^2^** | **Z2^2^** | **PI_HAT^3^** | **Pedigree^4^** | **Pedigree relationship^5^** | **fastSTRUCTURE^6^** |
| --- | --- | --- | --- | --- | --- | --- | --- | --- | --- |
| 1 | 1372 | 2153 | 0.10 | 0.72 | 0.18 | 0.54 | NA**^7^** | NA | TRUE |
| 2 | 1578 | 898 | 0.07 | 0.93 | 0.00 | 0.46 | TRUE | Half-Sib | TRUE |
| 3 | 1587 | 2110 | 0.00 | 1.00 | 0.00 | 0.50 | NA | NA | TRUE |
| 4 | 1587 | 807 | 0.00 | 1.00 | 0.00 | 0.50 | TRUE | Half-Sib | TRUE |
| 5 | 1661 | 2153 | 0.09 | 0.74 | 0.17 | 0.54 | NA | NA | TRUE |
| 6 | 1846 | 2179 | 0.10 | 0.86 | 0.04 | 0.47 | NA | NA | TRUE |
| 7 | 1846 | 2190 | 0.08 | 0.83 | 0.09 | 0.51 | NA | NA | TRUE |
| 8 | 1908 | 2110 | 0.08 | 0.90 | 0.02 | 0.47 | NA | NA | TRUE |
| 9 | 1908 | 807 | 0.05 | 0.94 | 0.01 | 0.48 | TRUE | Parent-Child | TRUE |
| 10 | 1965 | 1985 | 0.10 | 0.88 | 0.02 | 0.46 | TRUE | Half-Sib | TRUE |
| 11 | 1978 | 2141 | 0.27 | 0.38 | 0.35 | 0.54 | NA | NA | TRUE |
| 12 | 1978 | 2195 | 0.27 | 0.38 | 0.35 | 0.54 | NA | NA | TRUE |
| 13 | 2003 | 807 | 0.05 | 0.95 | 0.00 | 0.47 | FALSE | NA | TRUE |
| 14 | 2008 | 2212 | 0.00 | 1.00 | 0.00 | 0.50 | NA | NA | TRUE |
| 15 | 2011 | 2110 | 0.10 | 0.73 | 0.17 | 0.54 | NA | NA | TRUE |
| 16 | 2012 | 2019 | 0.21 | 0.62 | 0.16 | 0.48 | TRUE | Half-Sib | TRUE |
| 17 | 2012 | 2219 | 0.21 | 0.64 | 0.16 | 0.47 | NA | NA | TRUE |
| 18 | 2012 | 784 | 0.00 | 1.00 | 0.00 | 0.50 | TRUE | Parent-Child | TRUE |
| 19 | 2013 | 807 | 0.07 | 0.93 | 0.00 | 0.47 | TRUE | Parent-Child | TRUE |
| 20 | 2019 | 784 | 0.00 | 1.00 | 0.00 | 0.50 | TRUE | Parent-Child | TRUE |
| 21 | 2065 | 2110 | 0.08 | 0.76 | 0.16 | 0.54 | NA | NA | TRUE |
| 22 | 2127 | 807 | 0.00 | 1.00 | 0.00 | 0.50 | NA | NA | TRUE |
| 23 | 2219 | 784 | 0.00 | 1.00 | 0.00 | 0.50 | NA | NA | TRUE |
| 24 | 784 | 898 | 0.07 | 0.93 | 0.00 | 0.46 | TRUE | Half-Sib | TRUE |

^1^Genotype-pairs identified as having parent-child, or half-sib relationship using identity-by-descent (IBD); ^2^Z0, Z1 and Z2 - P(IBD=0), P(IBD=1) and P(IBD=2) respectively; ^3^PI_HAT - Proportion IBD, P(IBD=2) + 0.5*P(IBD=1); ^4^Pedigree - TRUE, when the relationship identified in IBD analysis can be validated using the known pedigree information (Fig. 1b), and FALSE otherwise; ^5^Relationship between genotypes known from the available pedigree information (Fig. 1b); ^6^fastSTRUCTURE - TRUE, if the genotypes belong the same cluster, or share 50 % of the ancestry in the structure analysis (Fig. 3a); ^7^NA - Pedigree information not available from the Field-books of Blackmon-Reynolds breeding program.

**Supplementary Table 7. Comparison of five different approaches of population structure analyses with known maternal pedigree information available from Blackmon-Reynolds breeding program**

| **Accession** | **SNPhylo** | **Identity-by-state/Ward** | **PCA** | **fastSTRUCTURE** | **Expression/Ward** |
| --- | --- | --- | --- | --- | --- |
| 784 | 1 | 1 | 1 | 1 | 1 |
| 807 | 1 | 1 | 1 | 1 | 1 |
| 898 | 0 | 0 | 0 | 2 | 0 |
| 1661 | 0 | 0 | 0 | 2 | 0 |
| 1710 | 0 | 0 | 0 | 2 | 0 |
| 1718 | 1 | 1 | 1 | 1 | 1 |
| 1849 | 0 | 0 | 0 | 0 | 0 |
| 1908 | 1 | 1 | 1 | 1 | 1 |
| 1916 | 0 | 0 | 1 | 2 | 0 |
| 1943 | 1 | 1 | 0 | 1 | 1 |
| 1965 | 0 | 0 | 0 | 1 | 1 |
| 1970 | 1 | 1 | 1 | 1 | 1 |
| 1972 | 1 | 1 | 1 | 1 | 1 |
| 1978 | 0 | 0 | 0 | 0 | 0 |
| 1985 | 0 | 0 | 0 | 2 | 0 |
| 2003 | 1 | 1 | 1 | 1 | 0 |
| 2008 | 1 | 0 | 0 | 1 | 1 |
| 2011 | 1 | 0 | 0 | 1 | 1 |
| 2012 | 1 | 1 | 1 | 1 | 1 |
| 2013 | 1 | 1 | 1 | 1 | 1 |
| 2019 | 1 | 1 | 1 | 1 | 1 |
| 2030 | 0 | 0 | 0 | 1 | 0 |
| 2039 | 0 | 0 | 0 | 0 | 0 |
| 2065 | 1 | 1 | 0 | 1 | 1 |
| 2121 | 0 | 0 | 1 | 0 | 0 |
| 2127 | 1 | 1 | 1 | 1 | 0 |
| 2136 | 0 | 0 | 0 | 1 | 1 |
| 2141 | 1 | 1 | 1 | 1 | 1 |
| TRUE | 16 | 14 | 14 | 19 (1) + 5(2) | 16 |
| Proportion | 57.1 | 50.0 | 50.0 | 67.9 (1), 85.7 (1+2) | 57.1 |

^0^Accessions sharing same maternal parent, but placed in a different cluster

^1^Accessions sharing same maternal parent, and placed in the same cluster

^2^Accessions sharing same maternal parent, placed in a different cluster, but the pedigree can be verified using proportion of membership to the cluster.

**Supplementary Table 8. Linkage disequilibrium decay along the chromosomes, whole genome and transcripts at different r^2^ thresholds**

| **Chromosome** | **LD decay (kb)** | **LD decay (kb)** | **LD decay (Mb)** |
| --- | --- | --- | --- |
|  | **r^2^ ≤ 0.20** | **r^2^ ≤ 0.15** | **r^2^ ≤ 0.10** |
| 01 | 3-3.5 | 10-15 | 0.6-0.7 |
| 02 | 4-4.5 | 10-15 | 1-10 |
| 03 | 10-15 | 25-50 | 1-10 |
| 04 | 0.4-0.5 / 3-3.5 | 5-10 | 0.4-0.5 |
| 05 | 3-3.5 | 25-50 | 1-10 |
| 06 | 2.5-3 | 10-15 | 0.6-0.7 |
| 07 | 3-3.5 | 10-15 | 0.4-0.5 |
| 08 | 3-3.5 | 10-15 | 0.9-1 |
| 09 | 4-4.5 | 10-15 | 0.5-0.6 |
| 10 | 3.5-4 | 20-25 | 1-10 |
| 11 | 3.5-4.0 | 15-20 | 1-10 |
| Genome | 3.5-4 | 10-15 | 0.7-0.8 |
| Transcripts | 4-4.5 | - | - |
| Categorical Median | 3.5-4 | 10-15 | 0.7-1 |

**Supplementary Table 9. Potential donor lines based on SNP based marker-trait associations identified in the collection**

| **Trait** | **SNP** | **Alleles^1^** | **Freq_EE^2^** | **Freq_EO^3^** | **Freq_OO^4^** | **Effect ± SE^5^** | **Mean ± SE^6^** | **Min.^7^** | **Max. ^8^** | **Favorable^9^** | **Accessions with favorable genotypes** |
| --- | --- | --- | --- | --- | --- | --- | --- | --- | --- | --- | --- |
| Yield/plant, g | S_806532 | T/C | 0.10 | 0.21 | 0.69 | 97.7 ± 23.4 | 281.0 ± 11.6 | 183.2 | 537.0 | TT | 1849 (2)^10^, 1972 (4), 2141, 2155 (1), 2195 |
| Tubers/plant, count | S_25048846 | T/C | 0.00 | 0.92 | 0.08 | -9.9 ± 2.5 | 19.0 ± 0.7 | 10.6 | 38.4 | CC | 2155 (2), 2183, 2191 (1), 898 (3) |
| Tubers/plant, count | S_41908941 | T/A | 0.00 | 0.96 | 0.04 | -14.1 ± 3.2 | 19.0 ± 0.7 | 10.6 | 38.4 | AA | 2155 (2), 898 (3) |
| Mother tuber weight, g | S_18970167 | T/C | 0.00 | 0.96 | 0.04 | -85.8 ± 20.9 | 72.7 ± 4.1 | 23.5 | 154.4 | CC | 1908 (2), 2170 (2) |
| Mother tuber length, cm | S_25933856 | G/A | 0.16 | 0.44 | 0.40 | 0.8 ± 0.20 | 5.8 ± 0.1 | 4.0 | 7.5 | GG | 1661 (3), 1849 (1), 1970, 1972 (1), 1978, 2148 (2), 2155, 2210 |
| Mother tuber width, cm | S_23095079 | C/T | 0.00 | 0.38 | 0.62 | -0.9 ± 0.23 | 4.1 ± 0.1 | 2.6 | 6.1 | TT | ~32 accessions |
| Child tuber weight, g | **S_12121278** | A/C | 0.02 | 0.19 | 0.79 | 15.3 ± 3.6 | 37.6 ± 1.3 | 23.7 | 78.8 | AA | 1972 (4) |
| Child tuber weight, g | S_28587045 | T/A | 0.04 | 0.23 | 0.73 | 11.8 ± 3.0 | 37.6 ± 1.3 | 23.7 | 78.8 | TT | 1972 (4), 2011 |
| Child tuber length, cm | **S_12121278** | A/C | 0.02 | 0.19 | 0.79 | 1.0 ± 0.2 | 5.0 ± 0.1 | 3.9 | 7.0 | AA | 1972 (4) |
| Child tuber length, cm | S_30406303 | G/A | 0.00 | 0.26 | 0.74 | 0.7 ± 0.2 | 5.0 ± 0.1 | 3.9 | 7.0 | GG | No homozygote accessions |

^1^Effective allele/other allele at the SNP location; ^2^Frequency of homozygous accessions with effective allele in the collection; ^3^Frequency of heterozygous accessions in the population; ^4^Frequency of homozygous accessions for other allele in the collection; ^5^SNP effect and associated standard error corresponding to the effective allele; ^6^Phenotypic mean and associated standard error based on the field trials conduct in Ames, IA 2011-2012; ^7^Minimum phenotypic value recorded in the collection; ^8^Maximum phenotypic value recorded in the collection; ^9^Favorable genotype; ^10^Number of environments in which the accession was among the top 10% of the performers for the same trait, or a highly (*r*>0.83, *p*<0.01) correlated trait.
