# Supplementary material for: Genomics-assisted characterization of a breeding collection of Apios americana, an edible tuberous legume
Source: Sci Rep. 2016 Oct 10;6:34908. doi: 10.1038/srep34908 (PMC5056515; doi:10.1038/srep34908)
Supplement: Supplementary Figures [file srep34908-s2.doc]

**Title page for Supplementary Figures 1-12, associated with the following paper:**

**Genomics-assisted characterization of a breeding collection of *Apios americana*, an edible tuberous legume**

Vikas Belamkar, Andrew D. Farmer, Nathan T. Weeks, Scott R. Kalberer, William J. Blackmon, Steven B. Cannon

**
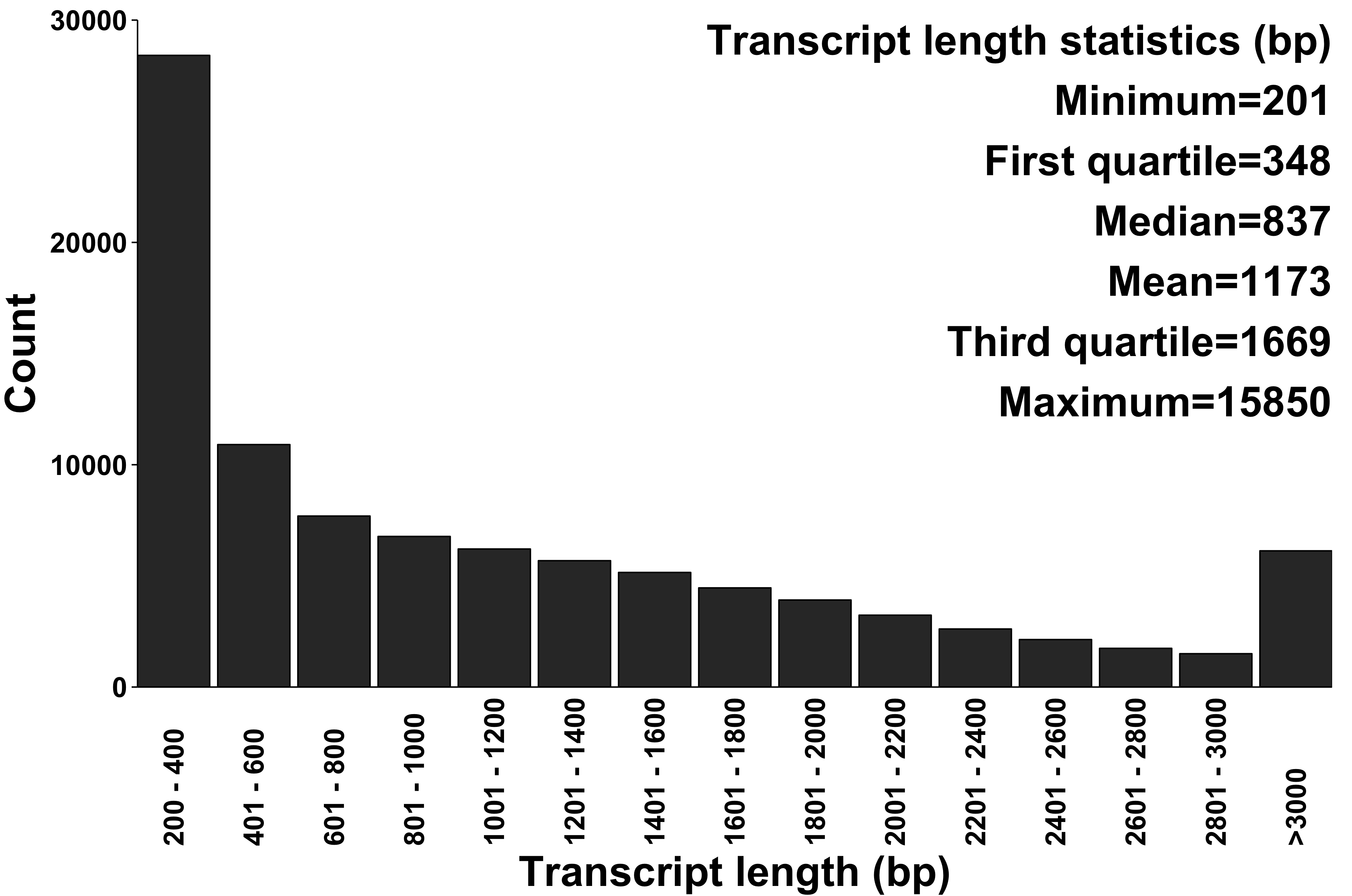
**

**Supplementary Figure 1. Transcript length distribution of the 96,560 transcripts in the *Apios americana* *de novo* assembly.**

**
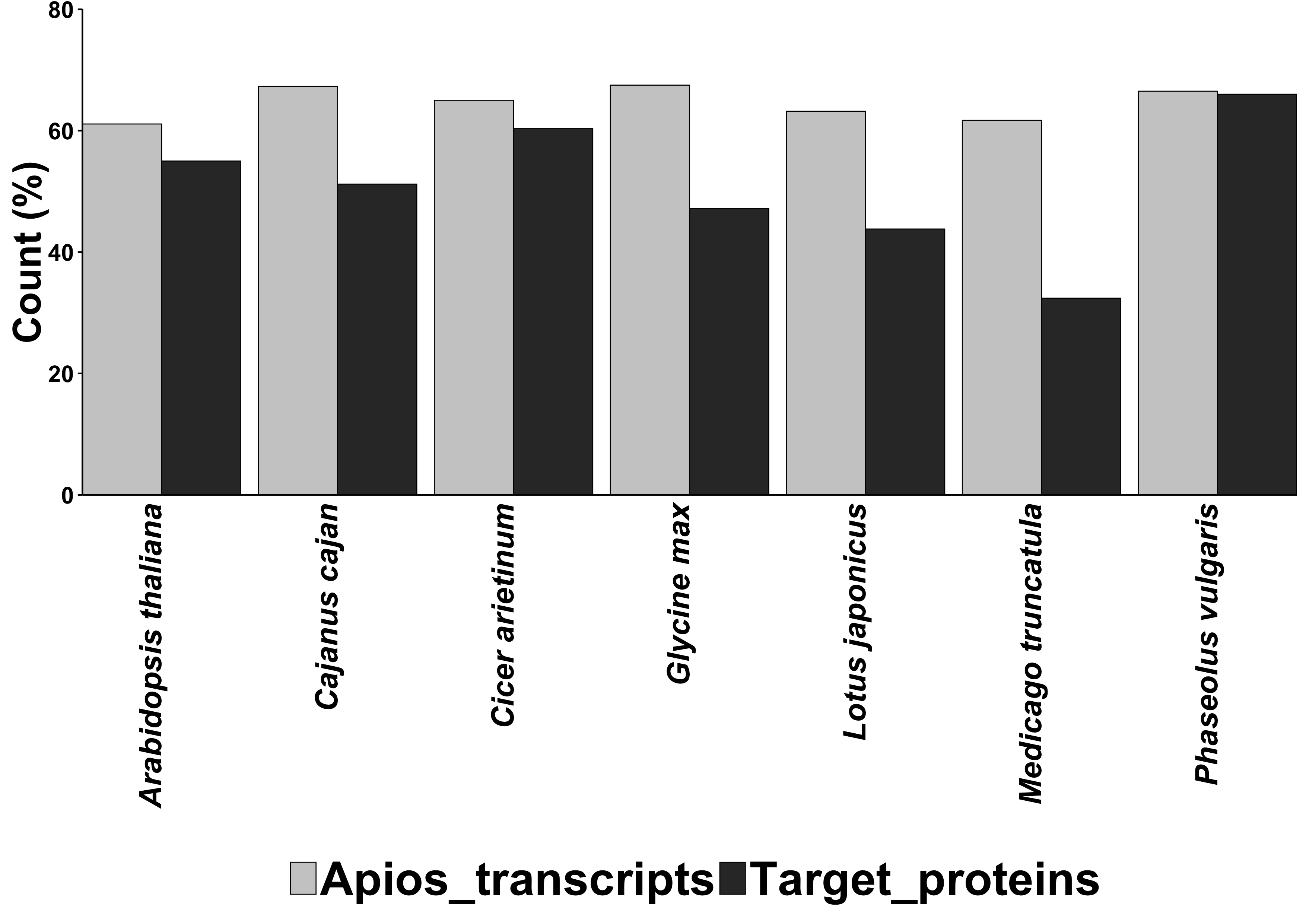
**

**Supplementary Figure 2. Sequence conservation of transcripts inspected by performing a BLASTX search with a threshold of 1E-05 against the proteomes of six related legumes and *Arabidopsis thaliana*.** Gray: percentage of transcripts in the assembly matching peptides; black: percentages of peptides in the respective species matching Apios transcripts (allowing only one match per Apios transcript).

**
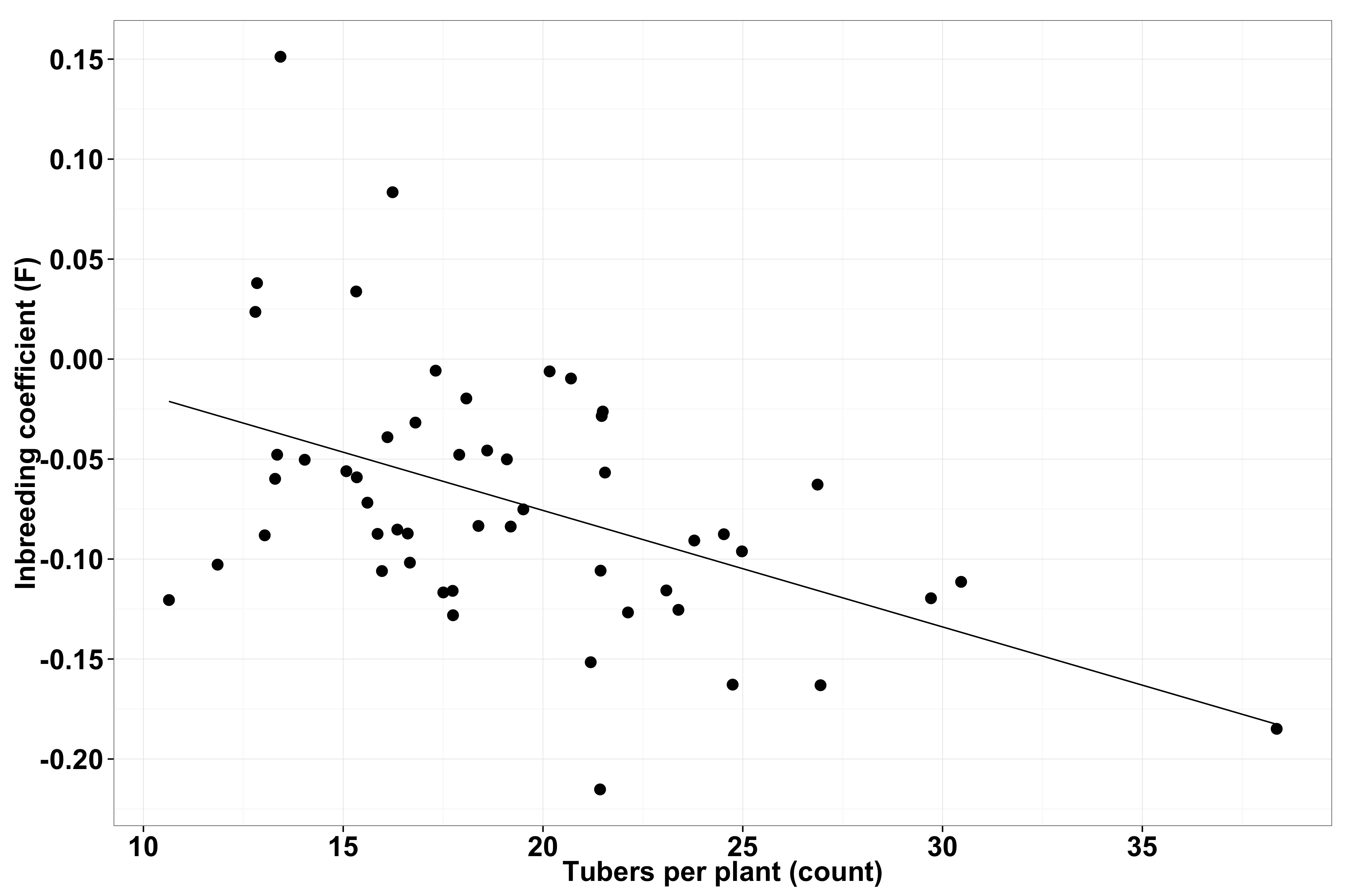
**

**Supplementary Figure 3. Negative correlation between inbreeding coefficients estimated for each genotype and tubers produced per plant recorded in Ames, IA during 2011-2012.** A linear regression was performed with tubers/plant as response variable and inbreeding coefficients as independent variables. The estimate was -38.57, standard error 10.13, t-value -3.807, *P*=0.000386, and the adjusted R-squared was 0.21.


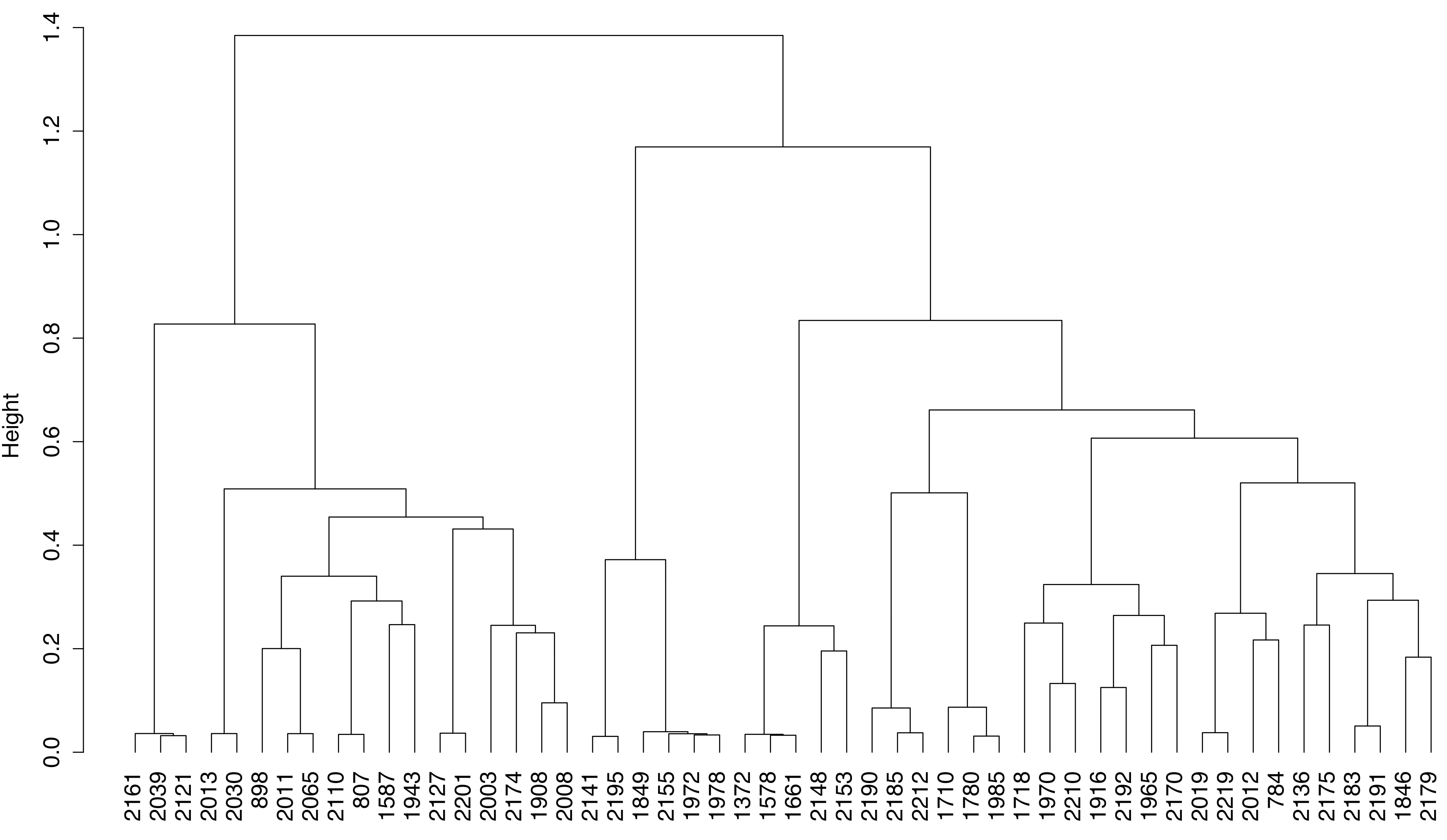


**6**

**1**

**4**

**1**

**5**

**2**

**5**

**3**

**Supplementary Figure 4.** **Phylogeny built using a distance matrix (1-IBS) based on Identity-by-state (IBS), and performing a hierarchical clustering using Ward’s linkage.** Numbers 1 to 6 represent the clusters identified in Fig. 3a.

**
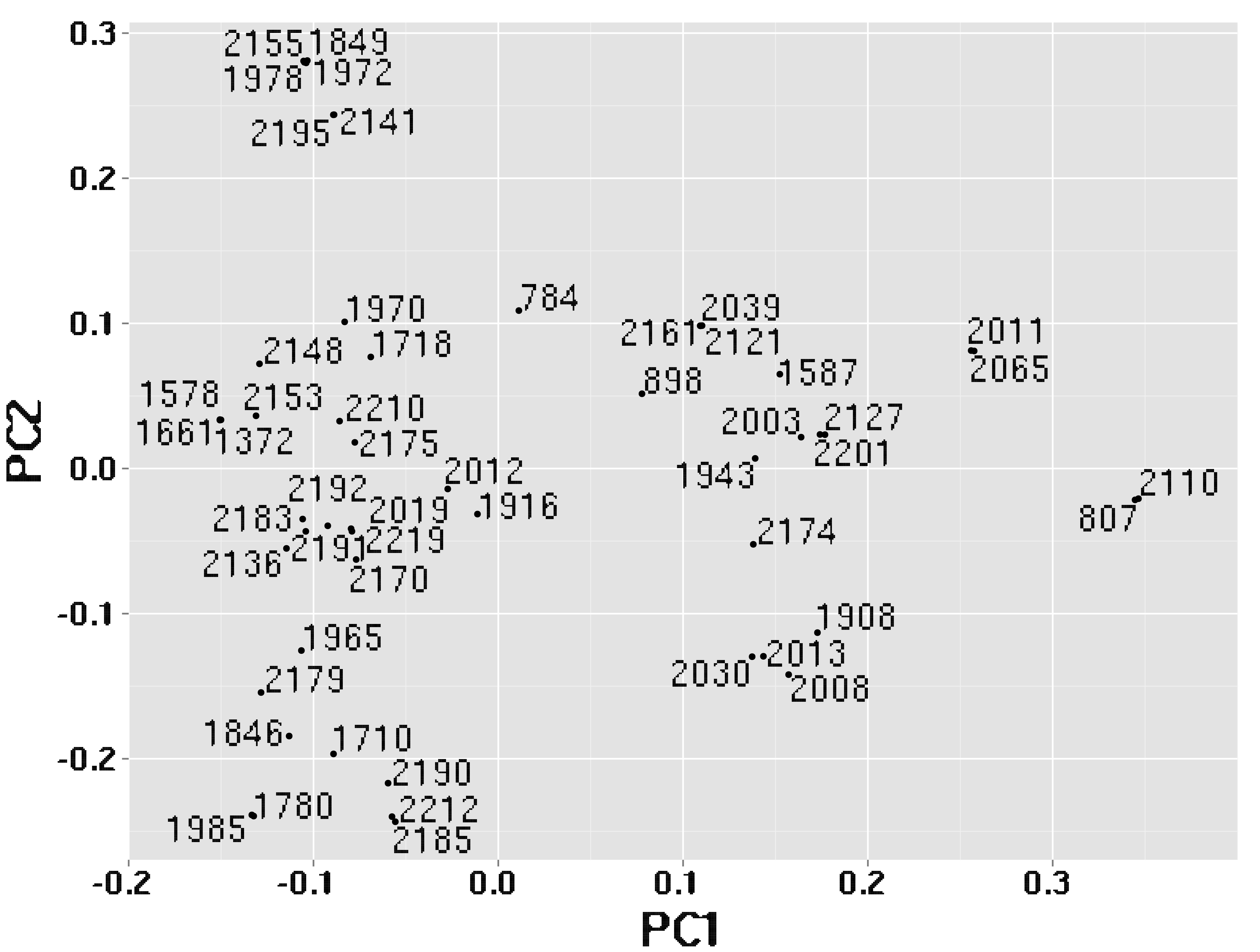
**

**Supplementary Figure 5.** **Principal component analysis.** PC1 and PC2 represent principal component 1 and 2 respectively.


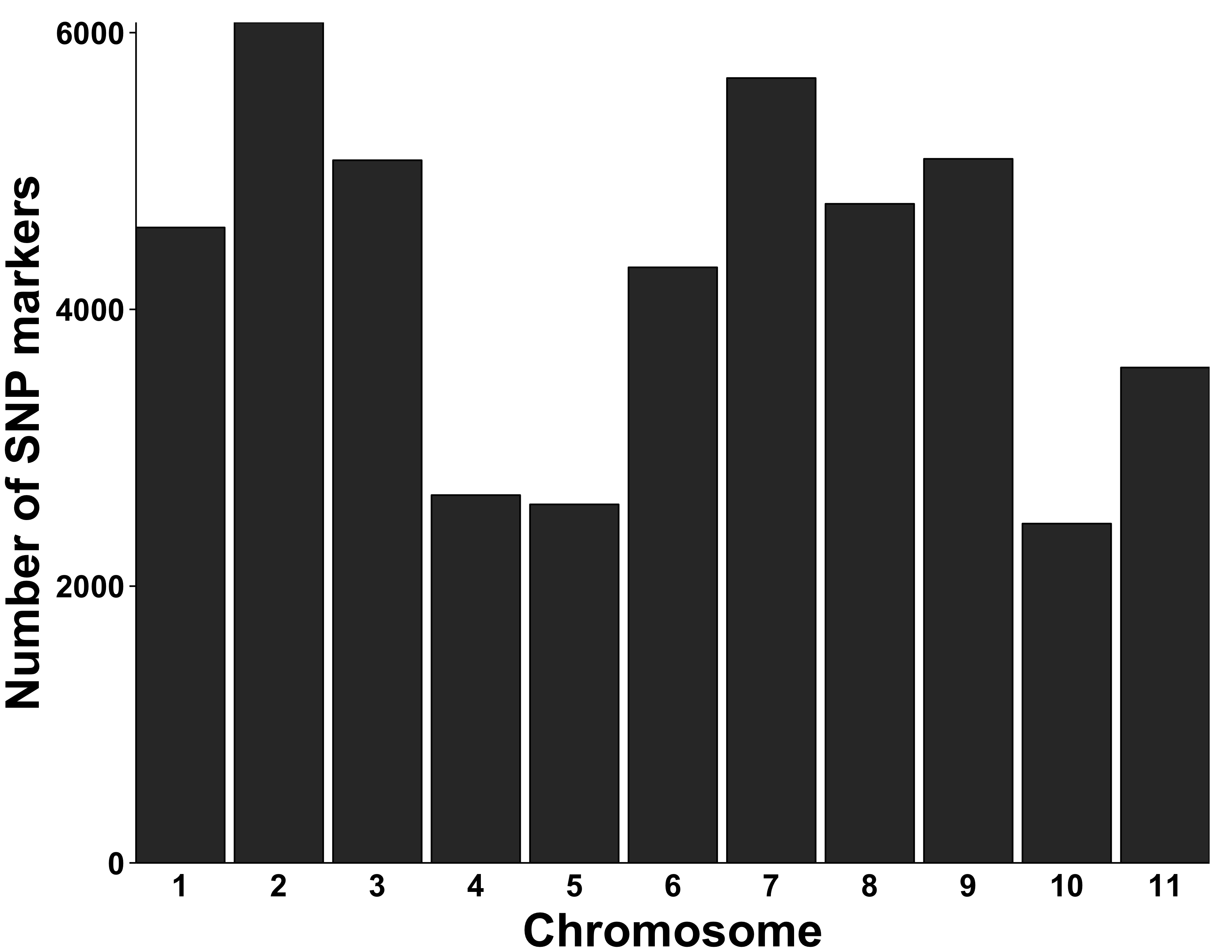


**Supplementary Figure 6. Number of Apios SNPs on each of the 11 *P. vulgaris* chromosomes.** Nearly 81% of the SNPs indentified in the Apios collection were mapped to the common bean genome. The mean and median numbers of SNPs per chromosome are 4,259 and 4,592, respectively.

**
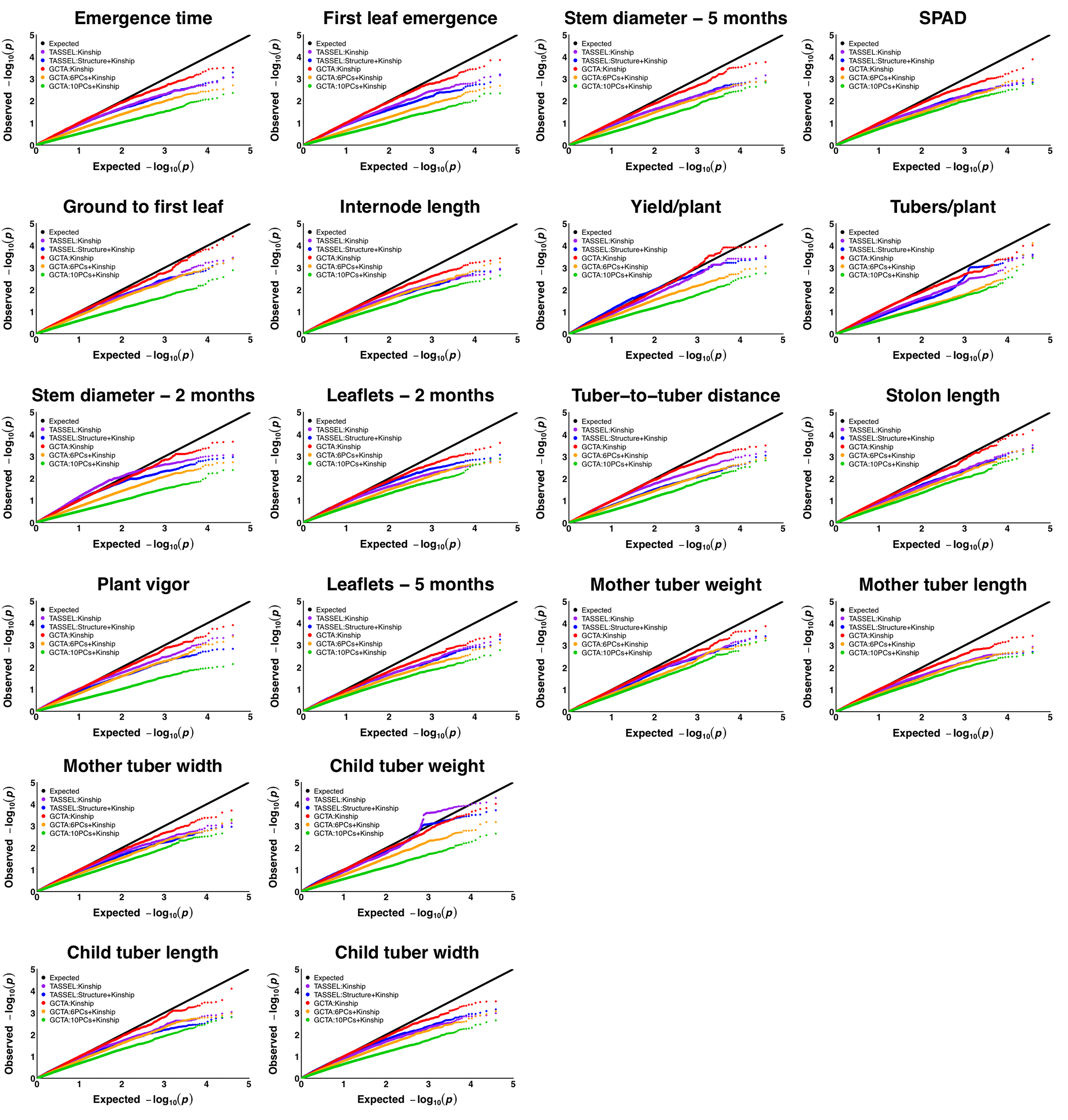
**

**Supplementary Figure 7. Quantile-Quantile (QQ) plots for evaluating performances of the different models and software packages utilized for association anlaysis.** Association analysis was first performed in the software program TASSEL by incorporating (1) familial relatedness matrix (kinship; generated in PLINK) in the linear mixed model as random effect; and (2) familial relatedness (as random effect) and subpopulation membership coefficients generated using fastSTRUCTURE as covariates. Association analysis was also conducted using “GCTA: a tool for Genome-wide Complex Trait Analysis” with the difference being familial relatedness matrix generated in GCTA, and either six or 10 principal components used to account for presence of subpopulations.

**
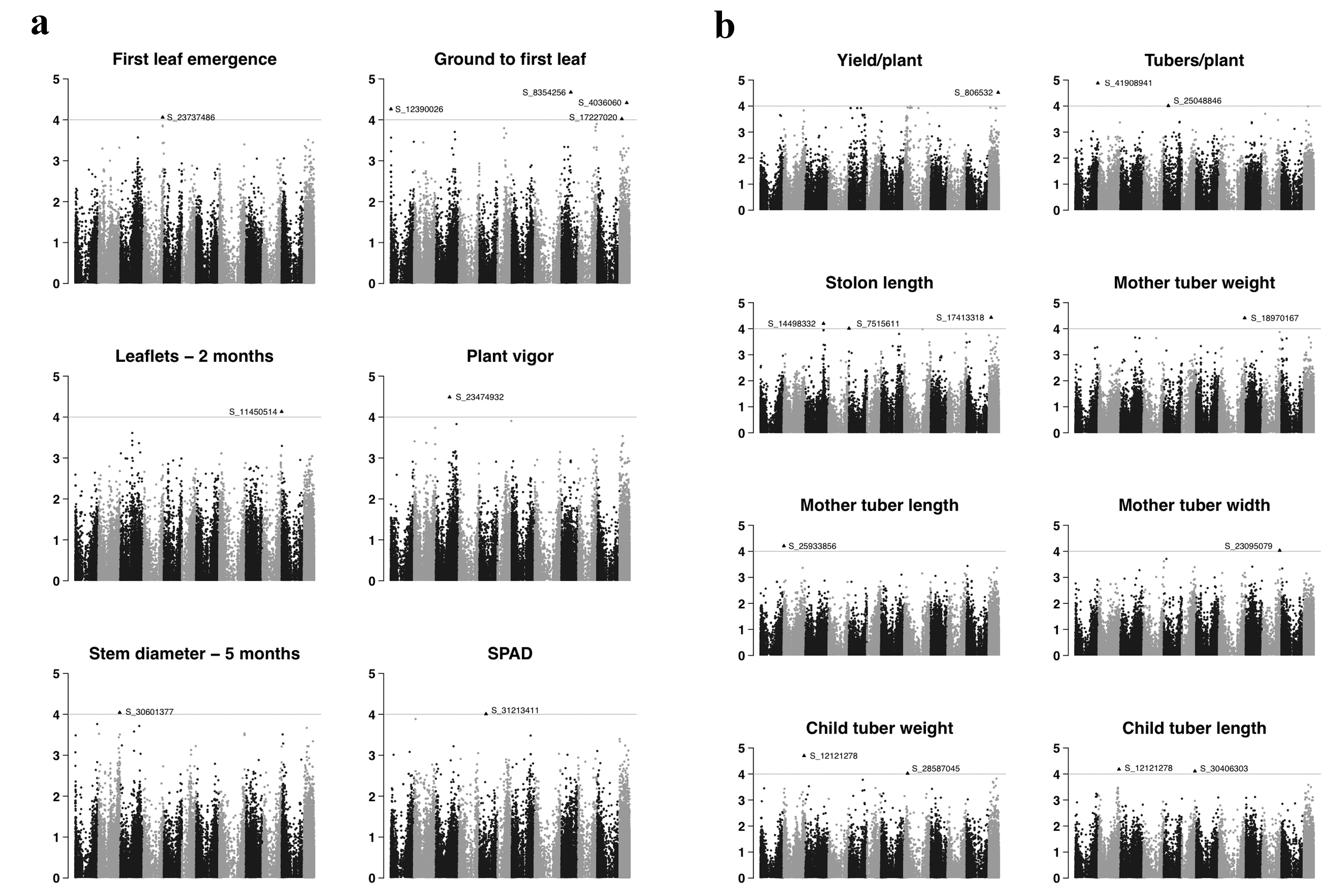
**

**Supplementary Figure 8. Manhattan plots displaying SNP marker-trait associations identified in the collection.** (a) Marker-trait associations with the aboveground traits. (b) Marker-trait associations with the belowground traits.

**
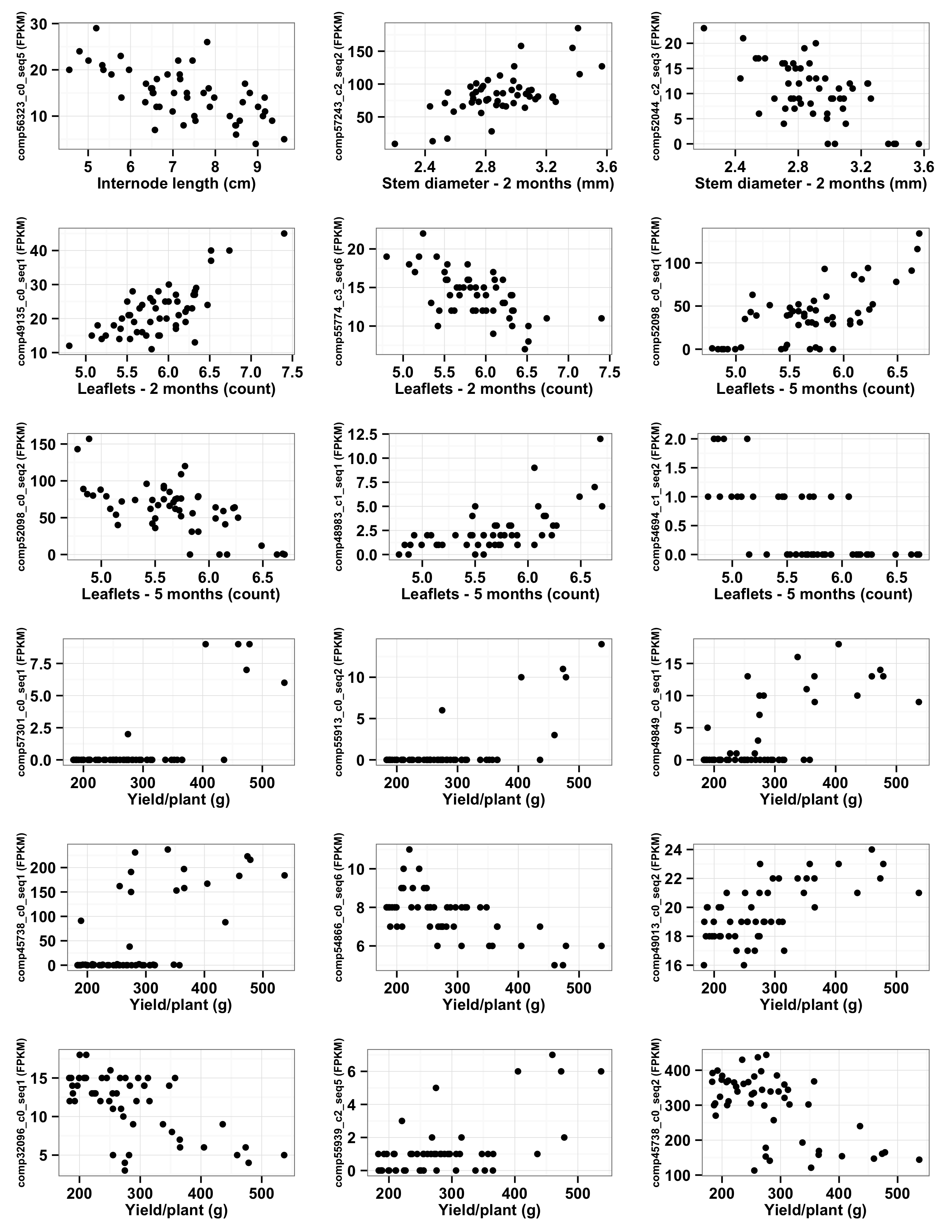
**

**Supplementary Figure 9. Gene expression markers (GEMs) associated with aboveground measurements and yield/plant after correcting for multiple-testing using Bonferroni correction of *P*<0.05 (new threshold *P*<0.0000013).** Each scatter plot corresponds to a GEM associated with a trait. The Y-axis represents normalized expression value of GEM in the 52 genotypes, and X-axis corresponds to the phenotypic measurement of the 52 genotypes for the respective trait.

**
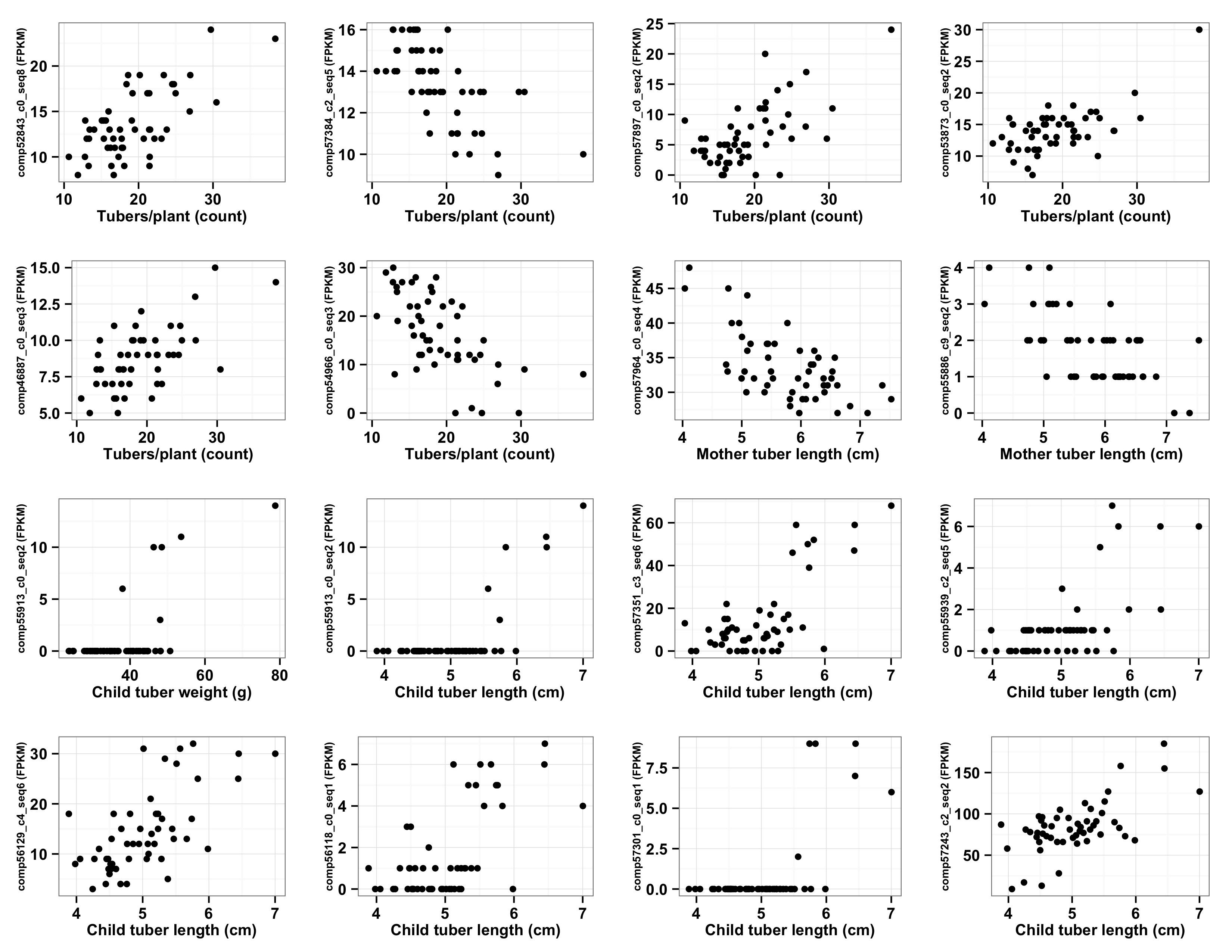
**

**Supplementary Figure 10. Gene expression markers (GEMs) associated with belowground measurements (except yield/plant) after correcting for multiple-testing using Bonferroni correction of *P*<0.05 (new threshold *P*<0.0000013).** Each scatter plot corresponds to a GEM associated with a trait. The Y-axis represents normalized expression value of GEM in the 52 genotypes, and X-axis corresponds to the phenotypic measurement of the 52 genotypes for the respective trait.

**
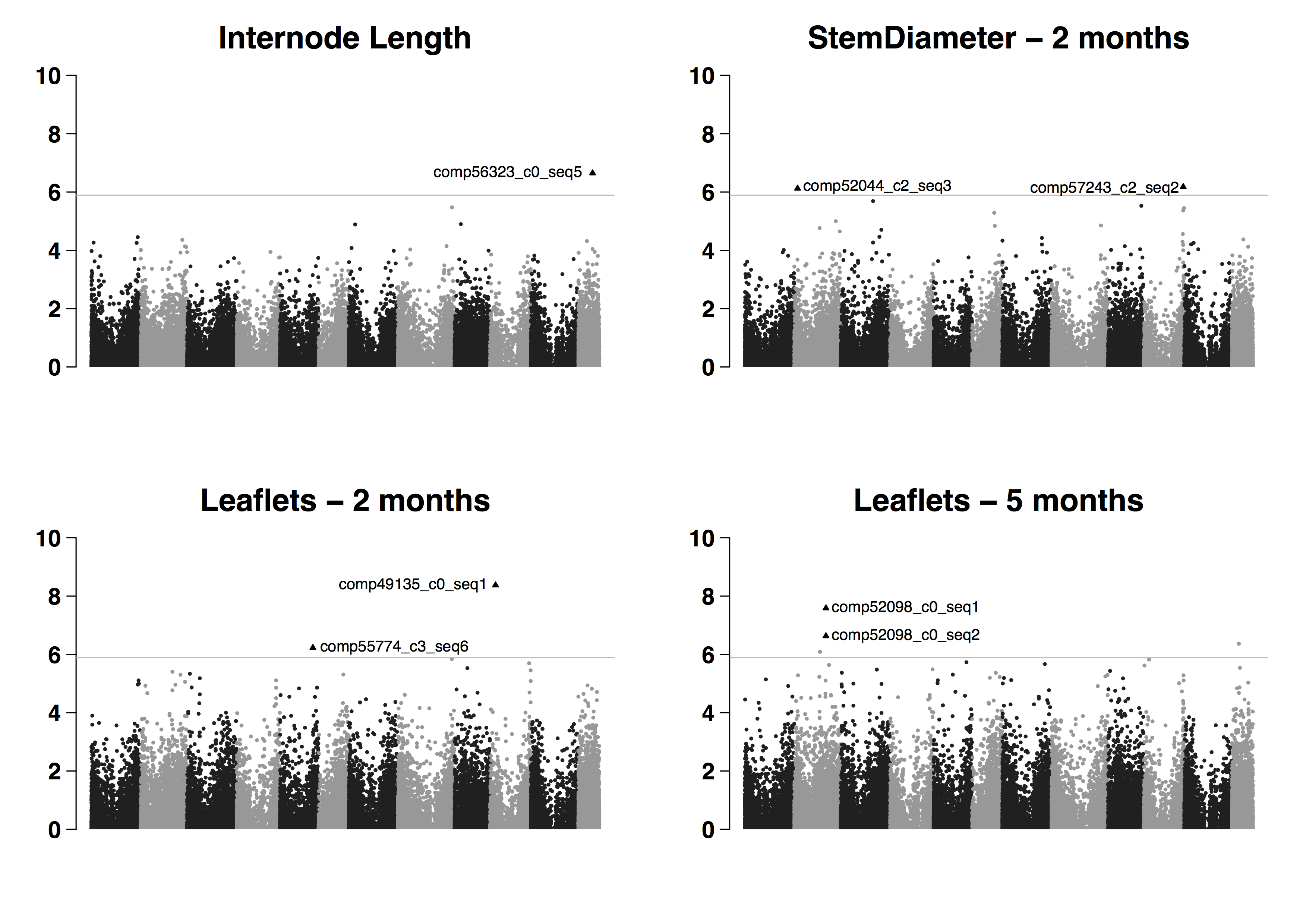
**

**Supplementary Figure 11. Manhattan plots displaying gene expression markers associated with the aboveground traits.**

**
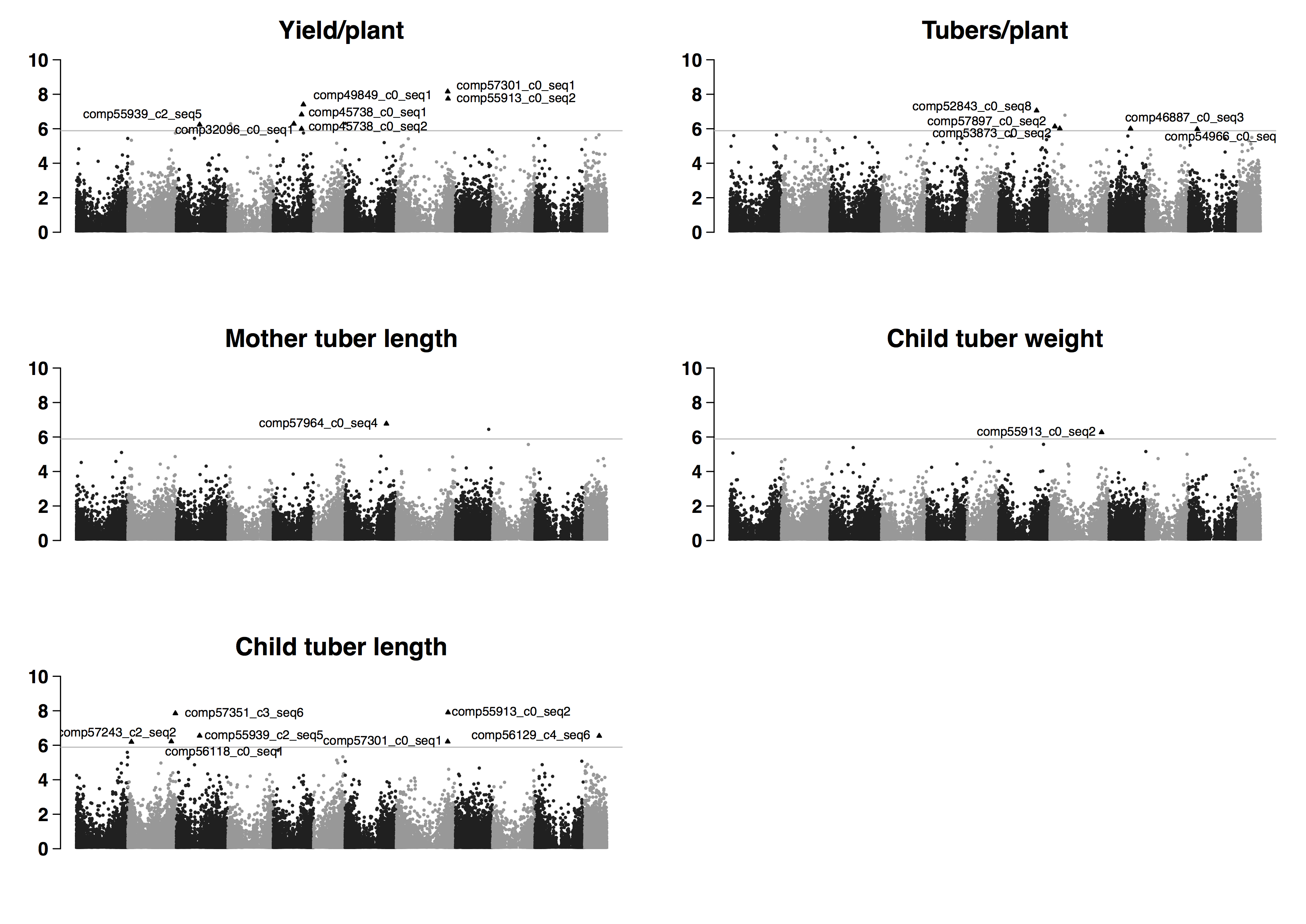
**

**Supplementary Figure 12. Manhattan plots displaying gene expression markers associated with the belowground trait**
