# Supplementary material for: Genomics-assisted characterization of a breeding collection of Apios americana, an edible tuberous legume
Source: Sci Rep. 2016 Oct 10;6:34908. doi: 10.1038/srep34908 (PMC5056515; doi:10.1038/srep34908)
Supplement: Supplementary Methods [file srep34908-s3.doc]

**Title page for Supplementary Methods 1, associated with the following paper:**

**Genomics-assisted characterization of a breeding collection of *Apios americana*, an edible tuberous legume**

Vikas Belamkar, Andrew D. Farmer, Nathan T. Weeks, Scott R. Kalberer, William J. Blackmon, Steven B. Cannon

Supplementary Methods 1: R code for replicating the analyses and plots in the article.

##########################################################################################################################################

##Figure2: Heat map of the normalized RNA-Seq data showing expression of transcripts in

##six tissues of accession 2127.

#Example input file

Transcript Shoot_R1 Shoot_R2 Leaf_R1 Leaf_R2 Root_R1 Root_R2 Mothertuber_R1 Mothertuber_R2 Childtuber_R1 Childtuber_R2 Flower

comp34266_c1_seq1 0.85 1.00 4.24 4.50 0.76 6.29 0.67 1.47 0.60 2.73 3.53

comp47272_c1_seq2 2.25 1.65 6.91 3.10 2.63 4.39 4.48 5.48 7.56 1.33 4.19

comp31609_c0_seq1 3.40 4.94 4.05 2.10 11.37 3.97 4.86 3.10 7.28 7.95 2.82

comp38917_c0_seq1 0.00 0.00 0.59 0.00 0.00 0.00 0.00 0.00 0.00 0.00 3.81

#R-code for Figure 2

#load the necessary libraries

#install.packages("RColorBrewer")

#install.packages("gplots")

library(RColorBrewer)

library(gplots)

#load file and change to matrix

expression=read.table("corrected_expressed_transcripts_FPKM", header=TRUE, sep="\t", row.names=1)

log2expression=log2(expression[1:56734,1:11])

#Replace -inf with zero

log2expression[log2expression == -Inf] = 0

#Select 1000 genes with highest variance across the samples

genes.var <- apply(log2expression, 1, var)

genes.var.select.1k <- order(genes.var, decreasing = T)[1:1000]

dat.s.1k <- log2expression[genes.var.select.1k, ]

#The above file dat.s.1k is a table of 1000 genes with highest variance across samples;

#and log2 fold changes values of transcritps are listed

#have output file a pdf/tiff

tiff("Log2_1000highestvariances_transcripts_heatmap_YellowToBlue_v2.tiff", height=8.27,

width=11.69, units="in", compression=c("lzw"), res=300)

#the gplots heatmap.2

heatmap.2(t(dat.s.1k),

Rowv=TRUE,

Colv=TRUE,

dendrogram= c("both"),

distfun = dist,

#hclustfun = hclust,

#xlab = "Response variables", ylab = "Predictors",

#xlab = "Tissues", ylab = "HD-ZipI",

labCol = NA,

cexRow=2.3, #Adjusting the size of the tissue labels

key=TRUE,

keysize=1.2,

trace="none",

density.info=c("none"),

#margins=c(12, 14),

margins=c(5, 15),

colsep=c(0:14), #Change this for different number of columns; Extra columns does not matter

rowsep=c(0:57000), #Change this for different number of rows; Extra rows does not matter

#sepcolor='black',

sepwidth=c(0.001,0.001),

#col=brewer.pal(11, "PiYG"),

#col=brewer.pal(9, "OrRd"),

#col=brewer.pal(9, "Greys"),

#col=bluered,

col=colorRampPalette(c("yellow","white","blue")),

#col=colorRampPalette(c("green","white","magenta")),

#col=colorRampPalette(c("turquoise","white","red")),

#col=greenred,

#lmat=rbind( c(0, 3.5), c(2.5,1.5), c(0,4.5) ),

#lmat=rbind( c(0, 3), c(2,1), c(0,4) ),

#lhei=c(0.25, 1.3, 0.15 ),

#lhei=c(1.5, 6, 1),

#lwid=c(1.5,6),

#col=redgreen(75),

#lty=2, #line style

#lwd=10, #line width

scale = c("none"),

RowSideColors=c(rep("#458B00",4), rep("#8B4513",6), rep("#458B00",1)),

offsetRow=0.0, #Adjust spacing between labels and heatmap

offsetCol=0.0

)

# unload the libraries

#detach("package:RColorBrewer")

# change back to the original directory

#setwd(initial.dir)

dev.off()

#display.brewer.all()

#brewer.pal.info

##########################################################################################################################################

##Figure 3 - Population structure. (a) Population structure using variational Bayesian

#framework - implemented in the program fastSTRUCTURE.

#Example input file

Genotypes Cluster1 Cluster2 Cluster3 Cluster4 Cluster5

1710 0.999993 0.000002 0.000002 0.000002 0.000002

1780 0.999993 0.000002 0.000002 0.000002 0.000002

1846 0.999993 0.000002 0.000002 0.000002 0.000002

1985 0.999993 0.000002 0.000002 0.000002 0.000002

2185 0.999993 0.000002 0.000002 0.000002 0.000002

2190 0.999993 0.000002 0.000002 0.000002 0.000002

#R-code for Figure 3a

data_k5=read.csv("R_run2_10_20_2_5_simple_K5_meanQ_sorted_ColumnClusterDeleted2MatchColumnReorder.txt",

header=TRUE, sep="\t")

#Converting Genotype to factors

data_k5$Genotypes=as.factor(data_k5$Genotypes)

#install.packages("reshape")

#install.packages("RColorBrewer")

library(RColorBrewer)

library(ggplot2)

library(reshape)

#Covert the data from table form to long form

df_k5 <- melt(data_k5, id.vars = c("Genotypes"), variable_name = 'series', na.rm=FALSE)

#http://stackoverflow.com/questions/14402242/keep-same-order-as-in-data-files-when-using-ggplot

#Important to match the order in the X-axis

df_k5=transform(df_k5, Genotypes=factor(Genotypes, levels=unique(Genotypes)))

# Bar plot, each cluster in the population colored differently --

plot_k5=ggplot(df_k5, aes(x=Genotypes, y=value, fill=series)) +

geom_bar(stat="identity", colour="black") + theme_bw() +

theme(axis.text.x = element_text(colour = 'black', face="bold", angle = 90, size = 16, vjust=0.5, hjust=1)) +

theme(axis.text.y = element_text(colour = 'black', face="bold", size = 16)) +

theme(axis.title.x = element_text(colour = 'black', face="bold", size = 16, vjust=0)) +

theme(axis.title.y = element_text(colour = 'black', face="bold", size = 16, angle=90)) +

labs(y="Proportion of membership", x="Genotypes", fill="Clusters") +

scale_fill_brewer(palette = "Accent") +

theme(

plot.background = element_blank()

,panel.grid.major = element_blank()

,panel.grid.minor = element_blank()

,panel.border = element_blank()

) +

theme(axis.ticks.x = element_blank()) +

theme(legend.text = element_text(size = 16, colour = "black", face="bold")) +

theme(legend.title = element_text(size = 16, colour = "black", face="bold")) +

scale_y_continuous(expand = c(0,0)) #This line of code deletes the extra space above and below bar plots

#Plot for k=6

data_k6=read.csv("R_run2_10_20_2_5_simple_K6_meanQ_sorted_ColumnsReordered.txt",

header=TRUE, sep="\t")

#Converting Genotype variable to factor

data_k6$Genotypes=as.factor(data_k6$Genotypes)

#Covert the data from table form to long form

df_k6 <- melt(data_k6, id.vars = c("Genotypes"), variable_name = 'series', na.rm=FALSE)

#http://stackoverflow.com/questions/14402242/keep-same-order-as-in-data-files-when-using-ggplot

#Important to match the order in the X-axis

df_k6=transform(df_k6, Genotypes=factor(Genotypes, levels=unique(Genotypes)))

# Bar plot, each cluster in the population colored differently --

plot_k6=ggplot(df_k6, aes(x=Genotypes, y=value, fill=series)) +

geom_bar(stat="identity", colour="black") + theme_bw() +

theme(axis.text.x = element_text(colour = 'black', face="bold", angle = 90, size = 16, vjust=0.5, hjust=1)) +

theme(axis.text.y = element_text(colour = 'black', face="bold", size = 16)) +

theme(axis.title.x = element_text(colour = 'black', face="bold", size = 16, vjust=0)) +

theme(axis.title.y = element_text(colour = 'black', face="bold", size = 16, angle=90)) +

labs(y="Proportion of membership", x="Genotypes", fill="Clusters") +

scale_fill_brewer(palette = "Accent") +

theme(

plot.background = element_blank()

,panel.grid.major = element_blank()

,panel.grid.minor = element_blank()

,panel.border = element_blank()

) +

theme(axis.ticks.x = element_blank()) +

theme(legend.text = element_text(size = 16, colour = "black", face="bold")) +

theme(legend.title = element_text(size = 16, colour = "black", face="bold")) +

scale_y_continuous(expand = c(0,0)) #This line of code deletes the extra space above and below bar plots

#Plot for k=7

data_k7=read.csv("R_run2_10_20_2_5_simple_K7_meanQ_sorted_ColumnsReordered.txt",

header=TRUE, sep="\t")

#Converting Genotype variable to factor

data_k7$Genotypes=as.factor(data_k7$Genotypes)

#Covert the data from table form to long form

df_k7 <- melt(data_k7, id.vars = c("Genotypes"), variable_name = 'series', na.rm=FALSE)

#http://stackoverflow.com/questions/14402242/keep-same-order-as-in-data-files-when-using-ggplot

#Important to match the order in the X-axis

df_k7=transform(df_k7, Genotypes=factor(Genotypes, levels=unique(Genotypes)))

# Bar plot, each cluster in the population colored differently --

plot_k7=ggplot(df_k7, aes(x=Genotypes, y=value, fill=series)) +

geom_bar(stat="identity", colour="black") + theme_bw() +

theme(axis.text.x = element_text(colour = 'black', face="bold", angle = 90, size = 16, vjust=0.5, hjust=1)) +

theme(axis.text.y = element_text(colour = 'black', face="bold", size = 16)) +

theme(axis.title.x = element_text(colour = 'black', face="bold", size = 16, vjust=0)) +

theme(axis.title.y = element_text(colour = 'black', face="bold", size = 16, angle=90)) +

labs(y="Proportion of membership", x="Genotypes", fill="Clusters") +

scale_fill_brewer(palette = "Accent") +

theme(

plot.background = element_blank()

,panel.grid.major = element_blank()

,panel.grid.minor = element_blank()

,panel.border = element_blank()

) +

theme(axis.ticks.x = element_blank()) +

theme(legend.text = element_text(size = 16, colour = "black", face="bold")) +

theme(legend.title = element_text(size = 16, colour = "black", face="bold")) +

scale_y_continuous(expand = c(0,0)) #This line of code deletes the extra space above and below bar plots

##########################################

# Multiple plot function

#

# ggplot objects can be passed in ..., or to plotlist (as a list of ggplot objects)

# - cols: Number of columns in layout

# - layout: A matrix specifying the layout. If present, 'cols' is ignored.

#

# If the layout is something like matrix(c(1,2,3,3), nrow=2, byrow=TRUE),

# then plot 1 will go in the upper left, 2 will go in the upper right, and

# 3 will go all the way across the bottom.

#

multiplot <- function(..., plotlist=NULL, file, cols=1, layout=NULL) {

require(grid)

# Make a list from the ... arguments and plotlist

plots <- c(list(...), plotlist)

numPlots = length(plots)

# If layout is NULL, then use 'cols' to determine layout

if (is.null(layout)) {

# Make the panel

# ncol: Number of columns of plots

# nrow: Number of rows needed, calculated from # of cols

layout <- matrix(seq(1, cols * ceiling(numPlots/cols)),

ncol = cols, nrow = ceiling(numPlots/cols))

}

if (numPlots==1) {

print(plots[[1]])

} else {

# Set up the page

grid.newpage()

pushViewport(viewport(layout = grid.layout(nrow(layout), ncol(layout))))

# Make each plot, in the correct location

for (i in 1:numPlots) {

# Get the i,j matrix positions of the regions that contain this subplot

matchidx <- as.data.frame(which(layout == i, arr.ind = TRUE))

print(plots[[i]], vp = viewport(layout.pos.row = matchidx$row,

layout.pos.col = matchidx$col))

}

}

}

##########################################

#Open a image file and then use mutliplot to combine pictures, and save it into the opened image file

tiff("K5K6K7_ClustersMatched.tiff", height=10.91, width=17.71, units='in', res=300)

multiplot(plot_k5, plot_k6, plot_k7, cols=1)

dev.off()

#Helpful resource - To arrange multiple plots in a grid manner;

#http://stackoverflow.com/questions/13294952/left-align-two-graph-edges-ggplot

##########################################################################################################################################

##Figure 4 - Population structure derived from gene expression markers (GEMs).

##Example input file

Transcript 1372 1578 1587 1661 1710 1718 1780 1846 1849 1908 1916 1943 1965 1970 1972 1978 1985 2003 2008 2011 2012 2013 2019 2030 2039 2065 2110 2121 2127 2136 2141 2148 2153 2155 2161 2170 2174 2175 2179 2183 2185 2190 2191 2192 2195 2201 2210 2212 2219 784 807 898

comp34266_c1_seq1 0 1 0 1 1 2 2 1 1 1 1 2 1 1 0 1 1 0 1 0 1 1 3 0 1 0 1 1 1 1 1 1 0 1 1 1 1 2 1 2 1 2 1 2 1 1 2 1 1 2 1 1

comp47272_c1_seq2 4 4 4 4 4 4 4 3 4 4 4 3 5 4 3 5 4 5 4 4 2 5 3 4 4 4 4 4 4 4 5 5 4 5 4 2 3 5 4 4 3 3 4 3 5 4 4 3 4 2 5 2

comp31609_c0_seq1 4 2 3 2 2 2 2 2 2 3 3 2 2 1 1 1 3 2 2 3 3 3 1 3 3 2 3 3 2 1 2 4 2 2 2 3 2 2 3 3 2 2 4 2 2 2 2 2 1 2 3 2

comp57517_c0_seq11 1 1 1 1 1 1 1 2 0 1 1 1 1 1 0 0 1 1 1 1 1 1 0 1 0 1 1 0 0 1 0 2 0 0 0 1 1 0 1 1 1 1 1 1 0 1 1 1 0 0 1 1

#load the necessary libraries

#install.packages("RColorBrewer")

#install.packages("gplots")

library(RColorBrewer)

library(gplots)

#load file

expression=read.table("apportioned.apios.TMM_normalized_headerEdited_GenoFiltered_ExWild_expressed_XinHeaderExc",

header=TRUE, sep="\t", row.names=1)

log2expression=log2(expression[1:39609,1:52])

#Replace -inf with zero

log2expression[log2expression == -Inf] = 0

#Select 1000 transcripts with highest variance across the samples

genes.var <- apply(log2expression, 1, var)

genes.var.select.1k <- order(genes.var, decreasing = T)[1:1000]

dat.s.1k <- log2expression[genes.var.select.1k, ]

#The above file dat.s.1k is a table of 1000 genes with highest variance across samples;

and log2 fold changes values of transcritps are listed

#calculate distance

t_dat.s.1k=t(dat.s.1k) #transpose the dataset

t_dat.s.1k.dist <- dist(t_dat.s.1k)

length(t_dat.s.1k.dist)

#The above len should be 1326 because (52*51/2) there are 52 genotypes

#To do a hierarchical cluster analysis use the function hclust.

#Single Linkage method

#t_data.std.dist.hc.single <- hclust(t_data.std.dist, method="single")

#plot(t_data.std.dist.hc.single, hang=-1)

#Wards method

t_dat.s.1k.dist.ward <- hclust(t_dat.s.1k.dist, method="ward")

plot(t_dat.s.1k.dist.ward, hang=-1)

#have output file a pdf/tiff

tiff("Log2_1000highestvariances_transcripts_52Genotypes_heatmap_DendrogramBoth.tiff",

height=8.5, width=11.0, units="in", compression=c("lzw"), res=300)

#the gplots heatmap.2

heatmap.2(t(dat.s.1k),

Rowv=TRUE,

Colv=TRUE,

dendrogram= c("both"),

distfun = dist,

#hclustfun = hclust,

#xlab = "Response variables", ylab = "Predictors",

#xlab = "Tissues", ylab = "HD-ZipI",

labCol = NA,

cexRow=1.0, #Adjusting the size of the tissue labels

key=TRUE,

keysize=1.0,

trace="none",

density.info=c("none"),

#margins=c(12, 14),

margins=c(5, 15),

colsep=c(0:60), #Change this for different number of columns; Extra columns does not matter

rowsep=c(0:57000), #Change this for different number of rows; Extra rows does not matter

sepcolor='black',

sepwidth=c(0.01,0.01),

#col=brewer.pal(11, "PiYG"), # the number in paranthesis (eg 11) indicates the number of colors in the palette, minimum is 3

#col=brewer.pal(3, "OrRd"),

#col=brewer.pal(9, "Greys"),

#col=brewer.pal(11, "Spectral"),

#col=brewer.pal(9, "YlOrRd"),

col=brewer.pal(9, "Reds"),

#col=bluered,

#col=greenred,

#lmat=rbind( c(0, 3.5), c(2.5,1.5), c(0,4.5) ),

#lmat=rbind( c(0, 3), c(2,1), c(0,4) ),

#lhei=c(0.25, 1.3, 0.15 ),

#lhei=c(1.5, 6, 1),

#lwid=c(1.5,6),

#col=redgreen(75),

scale = c("none"),

offsetRow=0.0, #Adjust spacing between labels and heatmap

offsetCol=0.0

)

dev.off()

##########################################################################################################################################

##Figure 5 - Genome-wide SNP distribution, linkage disequilibrium and haplotype blocks.

##(a) Distribution of SNPs identified in the Apios collection along the 11 Phaseolus

##vulgaris chromosomes.

#Example input file

Chromosome bp

01 125588

01 125597

01 125754

01 125800

#R-code for Figure 5a

data=read.csv("Chr_bp_Pv_10_20_2_5_Blackmon_SNPs_maf0.1_Max-mis0.90.recode", header=TRUE, sep="\t")

#Converting Chromosome to factors

data$Chromosome=as.factor(data$Chromosome)

#Converting scale from bp to Mb

data$Mb=data$bp*0.000001

library(RColorBrewer)

library(ggplot2)

library(reshape)

#Plot

ggplot(data, aes(x=Mb, y=Chromosome)) + theme_classic() +

#geom_point(colour="red", size=2) +

#scale_x_continuous(breaks = seq(0, 60, 10)) +

theme(axis.text.x = element_text(colour = 'black', face="bold", size = 20, vjust=0.5)) +

theme(axis.text.y = element_text(colour = 'black', face="bold", size = 20)) +

theme(axis.title.x = element_text(colour = 'black', face="bold", size = 25, vjust=-0.2)) +

theme(axis.title.y = element_text(colour = 'black', face="bold", size = 25, angle=90)) +

xlab("Length of chromosome (Mb)") +

ylab("Chromosome") +

geom_jitter(alpha=I(0.3)) +

theme(plot.background=element_blank(),panel.grid.major=element_blank(),panel.grid.minor=element_blank(),

panel.border=element_blank()) +

#theme(axis.ticks.x = element_blank()) +

scale_x_continuous(expand = c(0,0)) + scale_y_discrete(expand = c(0,0))

#Save the above plot in tiff format and 300 dpi resolution

ggsave("Pv_SNP_coverage_theme_classic.tiff", dpi=300, width=11, height=8.5, units="in")

##########################################################################################################################################

##Figure 5 - Genome-wide SNP distribution, linkage disequilibrium and haplotype blocks.

##(b) Decay of linkage disequilibrium along each of the putative chromosomes, across the genome, and transcripts.

#Example input file

Scale_Bins Chr1 Chr2 Chr3 Chr4 Chr5 Chr6 Chr7 Chr8 Chr9 Chr10 Chr11 Genome Transcripts

0.0-0.1 kb 0.29762 0.29997 0.31732 0.29065 0.2925 0.30289 0.30123 0.30967 0.2983 0.31923 0.32075 0.3044 0.30286

0.1-0.2 kb 0.28186 0.26828 0.26548 0.2481 0.27701 0.27571 0.26206 0.2713 0.26595 0.29397 0.27403 0.27017 0.26383

0.2-0.3 kb 0.26025 0.24417 0.26498 0.22685 0.2688991 0.27154 0.24801 0.27099 0.2473 0.2458955 0.24929 0.25493 0.25169

0.3-0.4 kb 0.24314 0.23814 0.24816 0.21764 0.25767 0.24932 0.23862 0.245 0.2482 0.25433 0.26641 0.2453 0.242

0.4-0.5 kb 0.22711 0.24421 0.24587 0.20187 0.22728 0.23984 0.23474 0.23565 0.2419 0.238342 0.25131 0.23709 0.24018

#R-code for Figure 5b

data=read.csv("R_Pv_10_20_2_5_Blackmon_LDDecay_Means.txt", header=TRUE, sep="\t")

#install.packages("reshape")

#install.packages("RColorBrewer")

library(RColorBrewer)

library(ggplot2)

library(reshape)

# plot the average correlation for each bin distance

df <- melt(data, id.vars = 'Scale_Bins', variable_name = 'series', na.rm=FALSE)

#http://stackoverflow.com/questions/4877357/how-to-plot-all-the-columns-of-a-data-frame-in-r

#Plot idea from above link

#http://stackoverflow.com/questions/14402242/keep-same-order-as-in-data-files-when-using-ggplot

#Important to match the order in the X-axis

df=transform(df, Scale_Bins=factor(Scale_Bins, levels=unique(Scale_Bins)))

#Plot on same grid, each series colored differently and also using solid and dashed lines

ggplot(df, aes(x=Scale_Bins, y=value, colour=series, linetype=series)) + #theme_classic() +

geom_line(aes(group = series), size=1.1) +

scale_linetype_manual(values = c(rep("solid", 11), rep("dashed", 2))) +

scale_color_manual(values = c(brewer.pal(8, "Dark2"), brewer.pal(3, "Set3"), c(1, "LightGreen"))) +

scale_y_continuous(minor_breaks = seq(0.0, 0.36, 0.01), breaks = seq(0.0, 0.36, 0.02)) +

theme(axis.text.x = element_text(colour = 'black', face="bold", angle = 90, size = 20, vjust=0.4, hjust=1.0)) +

theme(axis.text.y = element_text(colour = 'black', face="bold", size = 20)) +

theme(axis.title.x = element_text(colour = 'black', face="bold", size = 25)) +

theme(axis.title.y = element_text(colour = 'black', face="bold", size = 25, angle=90, vjust=1.5)) +

xlab("Distance") +

labs(y=expression(bold(Average~~""*r^"2"*""))) + #To bold and get the superscript on the Y-axis

theme(legend.text = element_text(size = 20, colour = "black", face="bold")) +

theme(legend.title=element_blank()) + #this will get rid of the word "series" as the title of the legend

#theme(legend.background = element_rect(fill="gray90", size=0.5, linetype="dotted")) + #Modifying the legend box

theme(legend.position=c(0.8, 0.7)) #Placing the legend in the top right corner of the plot

#Saving the above plot

ggsave("R_plot_DashedLine_10_20_2_5_Blackmon_LDDecay_AestheticsEdited.tiff", dpi=300, width=11, height=8.5, units="in")

##########################################################################################################################################

##Figure 5 - Genome-wide SNP distribution, linkage disequilibrium and haplotype blocks.

##(c) Distribution of haplotype blocks along each of the chromosomes.

#Example input file

CHR BP1 KB

1 144445 0.314

1 144836 60.693

1 245611 23.921

1 313312 0.631

1 333753 1.561

1 462893 0.92

1 464510 1.827

1 487975 0.001

1 494512 0.128

1 587373 0.008

1 587574 0.002

1 592272 0.042

1 592411 0.913

1 819233 0.002

#R-code for Figure 5c

data=read.csv("R_Pv_10_20_2_5_Blackmon_ChrStartKB_blocks", header=TRUE, sep="\t")

#Converting CHR to factors

data$CHR=as.factor(data$CHR)

#Converting BP1 scale from bp to Mb

data$BP1Mb=data$BP1*0.000001

#install.packages("reshape")

library(RColorBrewer)

library(ggplot2)

library(reshape)

#Make a dataframe with pericentromeric boundary coordinates to display in the below image

vline.data=data.frame(z=c(6.8,38,4.5,25.5,6,29.5,8,39.5,4,33.8,1,15,9.8,37.5,9.8,48,1.5,5.8,5.2,34,9.8,43),

CHR=c(1,1,2,2,3,3,4,4,5,5,6,6,7,7,8,8,9,9,10,10,11,11))

vline.data$CHR=as.factor(vline.data$CHR)

#Plot

ggplot(data, aes(x=BP1Mb, y=KB)) +

geom_point(colour="red", size=2) +

facet_wrap(~CHR) +

scale_x_continuous(breaks = seq(0, 60, 10)) +

scale_y_continuous(breaks = seq(0, 2000, 200)) +

theme(axis.text.x = element_text(colour = 'black', face="bold", size = 15, vjust=0.5)) +

theme(axis.text.y = element_text(colour = 'black', face="bold", size = 15)) +

theme(axis.title.x = element_text(colour = 'black', face="bold", size = 20, vjust=-0.25)) +

theme(axis.title.y = element_text(colour = 'black', face="bold", size = 20, angle=90, vjust=1.5)) +

xlab("Start position (Mb)") +

ylab("Size of haplotype block (kb)") +

geom_jitter(alpha=I(0.2))+

theme(strip.text.x = element_text(size = 15, face="bold", colour = "black")) +

geom_vline(aes(xintercept=z), vline.data, colour="black", linetype="dashed", size=0.5)

#Save the above plot

ggsave("R_plot_10_20_2_5_Blackmon_LDBlocksAlongChr_2MbSlidingWindow_Pericentromere_AestheticsEdited.tiff", dpi=300, width=11, height=8.5, units="in")

###############################################################################################################################################################

##Figure 6 - Scatter plots of nine interesting marker-trait associations identified

##using gene expression markers (GEMs).

##Example input file

Genotype Germination_Wk First_Leaf_Wk Soil_to_1stLeaf_cm Avg_Internode_len_cm Stem_dia_EarlyS_mm Leaflets_EarlySeason Plant_Vigor Leaflets_LateSeason Stem_dia_LateS_mm SPAD Avg_Yield_PerPlant_g Avg_Tubers_PerPlant Avg_tuber_tuber_dist_cm Approx_stolon_length_cm Max_tuber_wt_mother_g Max_tuber_length_moth_cm Max_tuber_width_moth_cm Max_tuber_wt_child_g Max_tuber_length_child_cm Max_Tuber_width_child_cm comp34266_c1_seq1 comp47272_c1_seq2 comp31609_c0_seq1 comp57517_c0_seq11 comp43553_c2_seq1 comp48316_c2_seq1 comp56533_c0_seq1 comp57528_c12_seq8 comp46624_c0_seq1 comp41914_c1_seq3 comp44688_c0_seq1 comp46100_c1_seq1 comp58124_c1_seq3 comp52693_c0_seq3 comp58079_c3_seq2 comp56122_c0_seq1 comp55973_c1_seq3 comp55682_c0_seq4 comp54084_c1_seq4 comp56033_c0_seq4 comp48642_c1_seq1 comp56043_c0_seq5 comp59404_c0_seq1 comp54008_c0_seq3 comp45052_c0_seq1 comp56737_c5_seq15 comp30309_c0_seq1 comp57784_c3_seq9 comp56617_c5_seq2 comp48757_c0_seq2 comp57775_c0_seq2 comp49710_c0_seq1 comp46365_c0_seq1 comp41418_c0_seq1 comp56572_c11_seq2 comp55706_c0_seq1 comp52504_c0_seq5 comp54525_c0_seq4 comp50300_c0_seq1 comp57227_c1_seq8 comp45068_c0_seq1 comp54664_c1_seq1 comp49756_c0_seq2 comp56075_c2_seq8 comp52157_c0_seq1 comp57499_c6_seq31 comp57987_c2_seq2 comp52957_c4_seq1 comp56799_c0_seq7 comp52793_c3_seq4 comp51059_c0_seq1 comp57087_c1_seq6 comp58213_c0_seq9 comp50573_c0_seq2 comp57743_c3_seq24 comp43917_c1_seq1 comp56209_c1_seq2 comp49555_c1_seq1 comp49198_c0_seq4 comp56648_c1_seq2 comp46018_c0_seq2 comp55130_c2_seq1 comp55931_c0_seq4 comp32845_c0_seq1 comp57344_c0_seq2 comp55062_c0_seq6 comp55273_c1_seq2 comp51882_c0_seq5 comp53771_c1_seq1 comp57223_c0_seq7 comp53915_c0_seq1 comp54266_c0_seq8 comp50473_c1_seq3 comp56470_c1_seq5 comp48120_c0_seq2 comp45963_c0_seq5 comp58199_c0_seq4 comp56659_c10_seq1 comp48557_c0_seq1 comp51623_c0_seq3 comp48899_c2_seq2 comp53013_c1_seq4 comp51471_c1_seq1 comp54548_c1_seq1 comp49783_c0_seq3 comp56700_c8_seq1 comp54171_c1_seq8 comp58246_c3_seq1 comp57128_c0_seq3 comp58166_c1_seq7 comp50521_c1_seq1 comp49411_c0_seq1 comp42309_c0_seq1 comp57849_c2_seq3 comp58173_c0_seq3 comp55045_c0_seq2 comp45122_c1_seq2 comp55987_c1_seq1 comp37541_c1_seq1 comp54378_c2_seq5 comp57232_c0_seq2 comp57658_c6_seq5 comp54075_c3_seq1 comp45865_c2_seq1 comp45498_c0_seq1 comp57671_c3_seq3 comp52317_c1_seq1 comp58277_c1_seq1 comp55122_c1_seq4 comp49228_c0_seq1 comp55250_c0_seq1 comp55600_c0_seq2 comp53956_c1_seq2 comp55497_c1_seq1 comp28611_c0_seq1 comp51276_c0_seq1 comp52968_c0_seq5 comp54651_c1_seq2 comp57140_c6_seq3 comp16369_c0_seq1 comp46415_c0_seq1 comp57789_c0_seq8 comp56667_c0_seq18 comp56610_c3_seq10 comp42516_c0_seq1 comp54018_c0_seq1 comp46906_c0_seq3 comp33182_c0_seq4 comp55255_c0_seq1 comp47459_c0_seq2 comp51790_c0_seq2 comp50574_c0_seq1 comp54704_c0_seq1 comp59985_c0_seq1 comp54865_c0_seq1 comp53511_c1_seq7 comp52004_c3_seq2 comp53206_c0_seq1 comp46945_c2_seq1 comp46092_c0_seq1 comp64668_c0_seq1 comp51621_c2_seq2 comp57320_c1_seq2 comp52320_c0_seq1 comp52767_c0_seq4 comp49621_c1_seq1 comp55718_c1_seq6 comp57956_c0_seq7 comp49482_c0_seq2 comp45804_c0_seq1 comp58113_c2_seq1 comp32235_c0_seq1 comp53773_c0_seq6 comp47243_c0_seq3 comp54637_c1_seq4 comp56563_c3_seq3 comp49132_c0_seq3 comp52499_c2_seq2 comp57873_c0_seq1 comp44727_c2_seq1 comp57393_c1_seq3 comp57515_c1_seq15 comp53865_c0_seq1 comp58285_c1_seq29 comp53176_c1_seq1 comp51046_c0_seq4 comp55596_c1_seq4 comp57153_c1_seq11 comp57588_c4_seq2 comp51020_c0_seq1 comp55477_c1_seq2 comp57574_c1_seq1 comp46025_c0_seq2 comp55581_c1_seq15 comp55653_c0_seq2 comp28682_c0_seq1 comp55875_c1_seq4 comp45706_c0_seq1 comp55100_c0_seq1 comp56749_c0_seq3 comp50410_c0_seq3 comp49732_c0_seq3 comp47844_c0_seq1 comp53427_c0_seq1 comp56585_c1_seq1 comp47569_c1_seq1 comp47734_c0_seq1 comp58067_c0_seq2 comp53168_c0_seq5 comp53880_c0_seq4 comp57951_c0_seq24 comp48562_c0_seq2 comp56331_c1_seq1 comp58289_c0_seq1 comp51599_c1_seq3 comp49989_c1_seq3 comp55881_c1_seq12 comp56447_c5_seq2 comp51997_c0_seq2 comp51126_c0_seq1 comp44789_c1_seq2 comp48029_c1_seq2 comp50588_c1_seq2 comp54011_c1_seq2 comp55747_c1_seq15 comp55817_c1_seq10 comp58062_c4_seq5 comp57751_c0_seq3 comp57807_c7_seq4 comp54541_c0_seq2 comp44576_c0_seq3 comp55573_c0_seq3 comp52320_c0_seq8 comp56341_c3_seq16 comp57389_c0_seq16 comp42969_c0_seq3 comp56716_c0_seq1 comp48239_c0_seq1 comp51837_c1_seq1 comp53271_c0_seq3 comp54997_c2_seq3 comp56420_c0_seq2 comp46027_c0_seq4 comp58080_c2_seq6 comp53098_c0_seq2 comp58196_c0_seq9 comp47218_c0_seq2 comp43418_c1_seq1 comp45805_c0_seq2 comp30294_c0_seq1 comp43780_c0_seq1 comp57058_c0_seq14 comp57879_c0_seq10 comp48743_c2_seq1 comp57988_c4_seq22 comp57434_c4_seq5 comp56408_c1_seq1 comp30477_c0_seq1 comp56660_c1_seq1 comp51671_c0_seq1 comp49775_c0_seq2 comp50269_c0_seq1 comp53018_c1_seq3 comp55556_c0_seq7 comp57038_c0_seq6 comp100882_c0_seq1 comp52969_c0_seq2 comp48022_c0_seq1 comp54616_c0_seq1 comp46397_c0_seq1 comp53886_c0_seq4 comp55522_c0_seq1 comp50947_c1_seq1 comp43024_c0_seq1 comp46172_c0_seq1 comp52950_c0_seq1 comp58234_c0_seq26 comp54146_c1_seq1 comp57034_c0_seq3 comp32717_c0_seq1 comp51459_c1_seq6 comp57270_c1_seq1 comp58037_c4_seq8 comp57681_c1_seq19 comp50067_c0_seq1 comp55993_c1_seq3 comp57106_c3_seq2 comp56254_c0_seq1 comp57602_c0_seq6 comp51174_c0_seq1 comp42949_c1_seq2 comp50848_c0_seq9 comp57387_c0_seq3 comp55749_c11_seq4 comp58202_c2_seq2 comp58939_c0_seq1 comp28666_c0_seq1 comp41880_c2_seq2 comp53423_c1_seq8 comp56913_c1_seq1 comp31014_c0_seq1 comp57341_c5_seq11 comp44873_c0_seq1 comp58040_c3_seq4 comp53599_c0_seq2 comp55495_c3_seq2 comp51329_c0_seq1 comp48801_c0_seq2 comp56893_c0_seq1 comp28280_c0_seq2 comp52434_c0_seq2 comp54214_c0_seq1 comp28046_c0_seq1 comp52438_c0_seq8 comp48522_c3_seq5 comp58101_c0_seq32 comp52157_c0_seq7 comp53061_c2_seq1 comp58184_c0_seq3 comp44344_c0_seq1 comp31322_c0_seq1 comp51587_c2_seq4 comp57023_c1_seq2 comp52850_c0_seq3 comp58105_c1_seq41 comp53136_c0_seq3 comp46360_c1_seq1 comp51895_c0_seq5 comp51947_c2_seq1 comp52622_c0_seq1 comp57955_c2_seq2 comp57497_c0_seq4 comp55236_c4_seq1 comp56022_c3_seq5 comp55982_c0_seq1 comp56064_c0_seq8 comp50587_c1_seq1 comp58153_c0_seq21 comp57055_c4_seq3 comp53902_c1_seq2 comp48978_c0_seq3 comp57670_c0_seq4 comp58171_c0_seq3 comp46679_c0_seq4 comp55377_c0_seq1 comp49948_c1_seq2 comp43712_c0_seq2 comp51876_c1_seq1 comp53364_c0_seq1 comp28899_c0_seq1 comp50344_c3_seq1 comp58258_c0_seq10 comp52696_c0_seq1 comp61379_c0_seq1 comp55680_c0_seq8 comp51216_c1_seq1 comp51892_c1_seq1 comp57023_c1_seq21 comp75034_c0_seq1 comp48617_c0_seq2 comp52535_c0_seq2 comp54564_c0_seq3 comp102001_c0_seq1 comp51542_c0_seq2 comp56596_c3_seq1 comp52199_c0_seq4 comp46703_c0_seq3 comp48436_c0_seq1 comp52884_c1_seq2 comp54927_c1_seq1 comp313947_c0_seq1 comp55889_c0_seq1 comp57531_c0_seq3 comp55753_c1_seq1 comp30041_c0_seq1 comp50797_c0_seq1 comp53049_c1_seq6 comp53378_c0_seq4 comp51436_c0_seq3 comp57483_c0_seq3 comp50903_c0_seq1 comp56336_c0_seq17 comp58087_c0_seq2 comp50529_c0_seq1 comp49078_c1_seq3 comp46392_c0_seq1 comp56940_c2_seq1 comp57467_c0_seq7 comp57848_c0_seq17 comp54889_c3_seq22 comp46185_c0_seq2 comp50014_c0_seq1 comp58074_c5_seq29 comp54757_c3_seq2 comp52854_c0_seq9 comp47230_c0_seq1 comp53269_c1_seq1 comp49909_c0_seq1 comp48213_c2_seq1 comp57813_c6_seq1 comp52757_c2_seq2 comp56080_c3_seq14 comp54525_c1_seq1 comp48429_c1_seq1 comp47805_c0_seq3 comp57484_c0_seq1 comp51734_c0_seq1 comp55645_c0_seq4 comp57274_c4_seq4 comp57158_c0_seq1 comp57257_c4_seq5 comp46756_c0_seq1 comp56969_c0_seq2 comp55410_c3_seq1 comp56845_c0_seq8 comp49361_c3_seq1 comp56989_c0_seq1 comp58059_c4_seq1 comp3280_c0_seq1 comp52615_c0_seq9 comp54510_c0_seq1 comp57789_c0_seq12 comp53752_c1_seq1 comp51365_c0_seq1 comp39284_c0_seq2 comp46247_c0_seq1 comp47940_c0_seq1 comp50228_c0_seq4 comp57525_c0_seq6 comp53888_c0_seq1 comp57789_c0_seq5 comp51919_c2_seq1 comp55048_c0_seq6 comp49810_c0_seq1 comp40511_c0_seq1 comp31345_c0_seq1 comp53675_c1_seq4 comp52480_c2_seq15 comp52865_c0_seq2 comp29406_c0_seq1 comp49265_c0_seq1 comp57936_c5_seq7 comp56813_c1_seq1 comp48025_c0_seq1 comp53317_c0_seq4 comp55880_c0_seq1 comp45778_c0_seq1 comp53841_c0_seq2 comp58048_c4_seq16 comp56872_c2_seq5 comp44674_c0_seq1 comp55839_c2_seq1 comp53078_c1_seq2 comp57965_c0_seq10 comp48473_c0_seq1 comp53524_c0_seq2 comp41720_c0_seq2 comp56134_c0_seq1 comp58143_c0_seq7 comp50998_c1_seq1 comp29389_c0_seq1 comp56948_c0_seq1 comp53626_c0_seq6 comp48382_c0_seq1 comp57041_c0_seq4 comp56515_c3_seq2 comp51100_c0_seq1 comp43750_c0_seq1 comp49489_c1_seq1 comp58074_c5_seq35 comp44422_c0_seq1 comp56243_c1_seq4 comp48518_c0_seq2 comp33824_c0_seq1 comp56880_c0_seq3 comp58102_c1_seq2 comp43949_c0_seq1 comp56066_c0_seq2 comp56692_c3_seq2 comp51002_c3_seq1 comp29641_c0_seq1 comp54973_c0_seq2 comp50522_c0_seq3 comp54666_c0_seq1 comp45752_c0_seq1 comp57087_c1_seq12 comp56431_c1_seq2 comp51251_c1_seq2 comp45995_c0_seq1 comp53046_c0_seq1 comp57510_c0_seq1 comp57232_c2_seq1 comp46537_c0_seq1 comp51502_c4_seq1 comp52619_c0_seq1 comp58240_c9_seq1 comp44339_c0_seq1 comp53462_c0_seq3 comp47549_c0_seq1 comp51676_c0_seq3 comp58257_c0_seq11 comp55962_c2_seq1 comp48869_c0_seq1 comp55644_c0_seq7 comp57978_c0_seq1 comp57487_c5_seq14 comp58200_c2_seq9 comp55129_c1_seq2 comp51521_c0_seq1 comp56443_c0_seq2 comp57007_c0_seq1 comp51808_c0_seq3 comp55799_c0_seq2 comp56772_c0_seq8 comp46893_c1_seq1 comp43879_c0_seq1 comp56039_c0_seq1 comp56823_c1_seq3 comp49413_c0_seq1 comp43989_c0_seq1 comp54336_c2_seq3 comp56987_c10_seq1 comp55944_c0_seq3 comp57073_c5_seq10 comp46801_c1_seq1 comp57495_c4_seq3 comp57846_c0_seq3 comp53482_c0_seq5 comp46431_c0_seq3 comp32720_c0_seq1 comp50190_c0_seq1 comp50476_c0_seq1 comp57687_c0_seq1 comp53856_c0_seq4 comp57357_c0_seq2 comp49982_c1_seq2 comp52647_c0_seq1 comp57795_c0_seq2 comp54547_c1_seq13 comp31413_c0_seq1 comp57307_c0_seq12 comp57710_c1_seq3 comp32991_c0_seq1 comp56010_c0_seq3 comp53688_c0_seq2 comp57583_c0_seq17 comp42652_c0_seq1 comp54860_c0_seq1 comp54467_c0_seq2 comp49975_c0_seq1 comp46531_c1_seq1 comp47169_c0_seq1 comp43455_c5_seq1 comp55315_c0_seq1 comp57829_c1_seq5 comp46965_c0_seq2 comp57378_c1_seq2 comp57151_c0_seq4 comp55790_c3_seq7 comp57501_c1_seq2 comp58241_c0_seq20 comp53331_c0_seq1 comp49046_c2_seq1 comp54304_c1_seq1 comp56617_c0_seq1 comp57678_c0_seq3 comp55593_c1_seq3 comp47522_c2_seq1 comp50583_c0_seq1 comp57820_c0_seq2 comp47235_c1_seq1 comp56885_c0_seq1 comp55278_c1_seq1 comp52173_c0_seq3 comp55557_c0_seq5 comp53340_c0_seq2 comp31551_c1_seq1 comp57500_c2_seq1 comp43822_c1_seq1 comp52998_c0_seq4 comp54685_c1_seq4 comp55877_c1_seq11 comp33653_c0_seq1 comp55762_c2_seq4 comp54949_c1_seq5 comp54227_c0_seq6 comp57758_c2_seq7 comp56216_c0_seq1 comp52359_c1_seq1 comp46784_c5_seq1 comp57830_c1_seq1 comp54383_c0_seq2 comp57713_c1_seq1 comp57499_c6_seq12 comp57849_c0_seq1 comp50093_c0_seq1 comp50921_c0_seq6 comp29855_c0_seq1 comp55569_c0_seq5 comp56135_c0_seq5 comp58092_c2_seq35 comp31992_c1_seq1 comp58108_c1_seq5 comp44215_c0_seq1 comp55103_c0_seq6 comp57368_c6_seq1 comp42617_c0_seq1 comp57831_c2_seq7 comp51245_c2_seq1 comp53319_c0_seq1 comp55072_c0_seq5 comp52853_c0_seq1 comp48125_c1_seq4 comp55484_c0_seq21 comp31116_c0_seq1 comp57298_c5_seq1 comp48044_c0_seq1 comp53743_c0_seq2 comp56650_c1_seq1 comp55209_c0_seq1 comp55307_c0_seq2 comp56578_c1_seq1 comp55366_c0_seq1 comp54418_c3_seq2 comp55619_c1_seq22 comp34540_c0_seq1 comp58285_c1_seq5 comp46831_c3_seq1 comp53060_c1_seq1 comp31080_c0_seq1 comp49690_c1_seq1 comp54212_c0_seq2 comp56408_c3_seq1 comp30291_c0_seq1 comp46541_c3_seq1 comp57682_c2_seq2 comp56196_c2_seq1 comp55803_c1_seq1 comp58137_c2_seq21 comp57626_c8_seq6 comp57848_c0_seq4 comp57368_c7_seq14 comp55870_c0_seq4 comp51389_c0_seq1 comp36832_c1_seq2 comp57658_c6_seq1 comp41031_c0_seq1 comp48555_c1_seq1 comp48641_c0_seq2 comp57539_c2_seq1 comp52232_c1_seq2 comp55367_c0_seq7 comp49767_c0_seq1 comp49140_c1_seq1 comp56345_c4_seq6 comp46467_c0_seq5 comp51685_c0_seq1 comp55101_c0_seq2 comp57776_c3_seq3 comp53127_c1_seq2 comp54286_c0_seq4 comp57243_c2_seq2 comp54676_c1_seq3 comp56436_c0_seq8 comp48469_c1_seq1 comp53721_c1_seq12 comp51787_c0_seq3 comp55917_c0_seq1 comp57159_c1_seq5 comp57145_c3_seq15 comp53571_c0_seq6 comp56235_c3_seq10 comp52961_c0_seq1 comp49723_c1_seq1 comp50519_c1_seq1 comp46749_c0_seq4 comp54407_c0_seq1 comp50606_c2_seq2 comp47257_c0_seq1 comp54788_c0_seq5 comp41505_c0_seq2 comp55466_c1_seq19 comp60403_c0_seq1 comp53833_c0_seq1 comp57900_c3_seq2 comp57748_c2_seq4 comp57533_c1_seq7 comp57241_c0_seq1 comp53708_c0_seq2 comp53695_c1_seq5 comp55128_c5_seq3 comp55238_c9_seq4 comp57253_c0_seq7 comp57136_c0_seq1 comp53955_c2_seq1 comp58016_c3_seq6 comp31790_c0_seq1 comp48883_c0_seq1 comp57637_c0_seq3 comp49737_c0_seq5 comp50556_c0_seq1 comp53403_c0_seq2 comp57758_c0_seq2 comp57539_c1_seq4 comp58101_c0_seq48 comp55685_c1_seq1 comp51510_c0_seq1 comp55034_c1_seq4 comp48123_c0_seq1 comp53410_c0_seq3 comp51971_c0_seq1 comp57698_c0_seq6 comp57821_c5_seq2 comp18096_c0_seq1 comp51042_c1_seq1 comp48163_c0_seq1 comp42671_c0_seq1 comp53562_c1_seq1 comp58237_c5_seq4 comp57654_c3_seq6 comp51335_c0_seq1 comp53914_c3_seq13 comp32091_c0_seq1 comp52007_c0_seq2 comp45962_c0_seq1 comp44789_c0_seq1 comp53053_c0_seq3 comp55804_c0_seq2 comp57788_c1_seq7 comp56226_c0_seq3 comp56274_c2_seq6 comp52730_c1_seq1 comp49431_c0_seq1 comp56071_c0_seq3 comp54765_c0_seq2 comp57129_c7_seq3 comp48746_c2_seq1 comp58472_c0_seq1 comp43900_c0_seq1 comp52027_c0_seq1 comp50431_c0_seq3 comp53595_c0_seq1 comp47801_c0_seq1 comp54148_c0_seq2 comp41755_c0_seq1 comp58265_c2_seq18 comp51548_c0_seq1 comp46750_c2_seq1 comp55226_c0_seq2 comp48271_c0_seq3 comp46096_c0_seq3 comp56401_c0_seq2 comp57904_c1_seq2 comp57761_c0_seq2 comp45476_c0_seq1 comp55162_c0_seq1 comp56688_c2_seq17 comp51325_c0_seq1 comp57770_c1_seq4 comp38833_c0_seq2 comp58158_c2_seq15 comp45066_c1_seq4 comp54378_c4_seq15 comp33690_c0_seq1 comp46282_c0_seq1 comp53921_c0_seq3 comp57397_c9_seq52 comp29421_c0_seq1 comp56357_c4_seq2 comp57684_c0_seq10 comp47650_c0_seq3 comp54453_c2_seq1 comp58298_c0_seq8 comp43460_c0_seq1 comp342425_c0_seq1 comp57806_c0_seq5 comp47591_c0_seq1 comp57681_c1_seq18 comp51230_c1_seq12 comp57904_c3_seq1 comp55395_c0_seq1 comp56204_c0_seq1 comp57105_c8_seq8 comp55859_c6_seq2 comp58134_c0_seq7 comp55531_c0_seq1 comp60466_c0_seq1 comp56248_c1_seq4 comp57850_c3_seq3 comp50620_c0_seq1 comp46342_c0_seq1 comp44808_c0_seq1 comp57481_c0_seq8 comp55389_c1_seq2 comp58011_c1_seq7 comp43385_c0_seq1 comp56088_c0_seq2 comp58248_c0_seq9 comp50831_c0_seq7 comp58294_c2_seq2 comp53679_c0_seq8 comp52167_c2_seq2 comp56709_c1_seq2 comp46426_c0_seq3 comp48135_c0_seq2 comp31572_c0_seq1 comp52694_c1_seq3 comp54724_c1_seq1 comp39332_c0_seq2 comp56938_c4_seq2 comp57774_c5_seq2 comp49317_c0_seq1 comp42716_c0_seq2 comp32880_c0_seq1 comp47199_c0_seq2 comp58246_c1_seq10 comp53660_c1_seq3 comp55994_c0_seq11 comp57298_c0_seq1 comp54593_c0_seq1 comp57598_c0_seq2 comp58279_c1_seq5 comp56091_c0_seq2 comp46820_c0_seq3 comp51133_c2_seq1 comp56374_c0_seq4 comp58003_c0_seq5 comp58098_c2_seq3 comp53481_c0_seq1 comp43519_c0_seq1 comp58259_c1_seq1 comp43178_c0_seq4 comp38711_c0_seq1 comp35698_c0_seq2 comp29832_c0_seq1 comp51243_c0_seq1 comp51924_c2_seq1 comp59273_c0_seq1 comp56936_c0_seq7 comp57095_c1_seq11 comp55687_c0_seq1 comp55603_c3_seq12 comp51672_c1_seq1 comp50662_c0_seq1 comp56338_c1_seq1 comp33935_c0_seq1 comp45850_c0_seq2 comp32572_c0_seq1 comp47111_c1_seq1 comp49976_c0_seq2 comp56489_c0_seq6 comp29375_c0_seq1 comp53898_c0_seq1 comp57504_c2_seq9 comp56095_c3_seq8 comp57012_c0_seq2 comp43270_c1_seq1 comp57360_c0_seq2 comp59463_c0_seq1 comp54024_c0_seq1 comp55606_c0_seq1 comp54477_c1_seq1 comp58197_c8_seq1 comp57609_c0_seq2 comp29234_c0_seq1 comp33384_c0_seq1 comp41580_c1_seq2 comp48389_c0_seq2 comp3045_c0_seq1 comp55297_c0_seq2 comp50097_c3_seq2 comp55540_c1_seq4 comp53833_c0_seq5 comp47011_c1_seq1 comp50023_c0_seq1 comp51002_c1_seq1 comp54601_c1_seq1 comp52737_c0_seq1 comp55495_c3_seq3 comp55409_c0_seq4 comp56630_c1_seq6 comp55124_c0_seq3 comp41611_c1_seq1 comp56031_c1_seq2 comp56221_c0_seq3 comp57981_c0_seq2 comp57031_c1_seq2 comp57805_c1_seq18 comp51646_c0_seq4 comp52260_c0_seq2 comp45929_c1_seq1 comp55989_c0_seq1 comp42503_c0_seq1 comp52396_c3_seq1 comp54817_c16_seq1 comp56462_c1_seq6 comp48871_c0_seq2 comp54276_c0_seq3 comp55204_c1_seq2 comp53296_c0_seq1 comp57499_c6_seq4 comp43647_c0_seq4 comp44036_c2_seq3 comp39162_c1_seq1 comp55994_c0_seq4 comp54105_c0_seq4 comp57514_c2_seq3 comp48953_c0_seq4 comp57298_c4_seq2 comp44822_c1_seq4 comp56964_c0_seq2 comp49717_c0_seq4 comp57783_c0_seq2 comp56665_c1_seq8 comp51527_c0_seq1 comp46318_c0_seq1 comp47747_c2_seq1 comp32078_c0_seq1 comp56338_c2_seq2 comp57170_c1_seq8 comp47445_c0_seq1 comp51446_c0_seq3 comp53206_c1_seq1 comp55830_c4_seq2 comp61239_c0_seq1 comp44280_c0_seq2 comp40967_c2_seq1 comp52682_c0_seq2 comp52205_c0_seq1 comp53742_c1_seq1 comp51996_c0_seq3 comp50056_c0_seq1 comp55093_c2_seq1 comp57741_c0_seq2 comp52325_c0_seq2 comp57525_c0_seq12 comp57528_c10_seq3 comp31583_c0_seq1 comp53301_c2_seq2 comp46924_c0_seq1 comp46817_c1_seq1 comp57086_c1_seq1 comp53952_c0_seq1 comp57843_c1_seq3 comp53400_c1_seq1 comp55003_c0_seq1 comp56632_c2_seq2 comp48339_c0_seq4 comp51693_c1_seq1 comp56336_c0_seq8 comp49029_c1_seq1 comp56326_c2_seq2 comp56570_c0_seq1 comp49689_c1_seq1 comp53524_c0_seq4 comp55065_c0_seq4 comp49130_c0_seq2 comp51857_c1_seq5 comp58576_c0_seq1 comp56503_c0_seq1 comp53576_c0_seq6 comp56720_c1_seq10 comp43806_c1_seq1 comp50971_c0_seq1 comp56345_c2_seq2 comp55263_c1_seq1 comp53138_c0_seq2 comp50804_c0_seq1 comp58137_c2_seq16 comp57476_c0_seq1 comp57596_c3_seq2 comp56856_c0_seq1 comp54372_c0_seq2 comp58270_c3_seq41 comp50360_c0_seq4 comp52882_c2_seq1 comp54854_c2_seq5 comp46383_c0_seq2 comp54483_c0_seq2 comp52030_c1_seq5 comp44735_c0_seq1 comp53853_c1_seq4 comp56809_c2_seq7 comp56768_c2_seq1 comp35258_c0_seq1 comp52822_c0_seq4 comp54396_c2_seq1 comp57848_c0_seq10 comp48967_c4_seq1 comp28264_c0_seq2 comp56953_c2_seq1 comp53343_c1_seq4 comp52971_c1_seq1 comp50697_c4_seq1 comp52474_c1_seq6 comp53522_c0_seq1 comp56942_c0_seq3 comp57800_c0_seq3 comp50233_c0_seq1 comp56290_c1_seq3 comp51965_c0_seq1 comp55262_c1_seq1 comp57646_c0_seq7 comp29396_c0_seq1 comp42102_c0_seq2 comp56733_c0_seq12 comp53985_c0_seq2 comp48908_c1_seq7 comp46122_c0_seq1 comp56957_c4_seq10 comp49565_c0_seq1 comp53684_c0_seq1 comp167861_c0_seq1 comp48738_c0_seq1 comp53398_c1_seq1 comp51835_c0_seq3 comp45384_c0_seq1 comp52432_c0_seq7 comp51758_c0_seq1 comp57167_c0_seq1 comp58050_c3_seq4 comp30080_c0_seq1 comp47959_c0_seq1 comp59177_c0_seq1 comp58275_c4_seq27 comp55767_c0_seq3 comp47498_c0_seq2 comp29119_c0_seq1 comp56335_c0_seq2 comp54786_c0_seq8 comp55469_c0_seq1 comp55270_c0_seq6 comp58021_c3_seq2 comp49191_c0_seq1 comp37111_c0_seq1 comp55640_c3_seq1 comp59068_c0_seq1 comp57145_c3_seq14 comp48927_c0_seq1 comp41996_c0_seq1 comp56719_c0_seq2 comp42507_c1_seq1 comp57649_c3_seq17 comp56842_c0_seq11 comp53924_c0_seq1 comp55449_c0_seq2 comp57252_c0_seq1 comp58197_c7_seq5 comp45524_c0_seq1 comp56580_c0_seq6 comp50454_c0_seq1 comp52081_c0_seq3 comp54678_c2_seq2 comp42464_c0_seq1 comp57446_c0_seq5 comp57122_c3_seq3 comp53099_c0_seq1 comp42765_c0_seq1 comp51370_c0_seq1 comp57291_c5_seq3 comp50356_c1_seq6 comp56829_c1_seq12 comp57188_c1_seq4 comp58121_c0_seq1 comp44217_c0_seq3 comp46034_c0_seq3 comp54295_c0_seq1 comp55783_c0_seq7 comp55454_c1_seq5 comp48590_c0_seq1 comp57459_c0_seq1 comp54944_c0_seq1 comp56654_c3_seq4 comp50120_c0_seq3 comp52119_c0_seq1 comp53635_c2_seq2 comp52141_c0_seq2 comp57525_c0_seq3 comp53194_c0_seq6 comp54663_c0_seq2 comp56705_c0_seq13 comp49498_c0_seq2 comp42844_c1_seq2 comp56699_c0_seq2 comp56477_c0_seq4 comp50845_c0_seq2 comp57369_c5_seq1 comp35317_c0_seq1 comp50151_c0_seq2 comp56353_c0_seq1 comp43117_c0_seq1 comp55863_c1_seq1 comp54563_c0_seq7 comp30011_c0_seq1 comp50960_c0_seq4 comp56136_c0_seq5 comp50066_c0_seq8 comp49742_c0_seq4 comp54804_c1_seq1 comp49349_c0_seq1 comp56737_c5_seq4 comp46393_c0_seq2 comp52922_c5_seq3 comp56646_c1_seq2 comp54994_c0_seq9 comp58236_c0_seq19 comp48804_c0_seq2 comp53753_c2_seq1 comp57153_c1_seq26 comp56992_c2_seq15 comp51212_c1_seq3 comp56006_c3_seq5 comp45015_c2_seq4 comp49357_c0_seq1 comp46069_c0_seq1 comp59120_c0_seq1 comp51474_c0_seq5 comp53821_c2_seq1 comp55606_c4_seq6 comp50630_c0_seq2 comp48193_c0_seq2 comp57951_c0_seq15 comp30532_c0_seq1 comp57116_c1_seq1 comp43395_c1_seq1 comp41751_c0_seq1 comp58212_c2_seq1 comp55430_c1_seq10 comp42653_c0_seq1 comp48903_c2_seq1 comp28945_c0_seq1 comp58076_c4_seq2 comp56946_c2_seq3 comp94698_c0_seq1 comp57750_c1_seq15 comp52090_c0_seq1 comp57474_c2_seq2 comp52727_c0_seq1 comp56700_c9_seq1 comp52582_c0_seq1 comp56243_c3_seq2 comp53234_c0_seq4 comp53533_c1_seq5 comp53764_c2_seq1 comp57549_c0_seq3 comp56256_c0_seq4 comp55647_c0_seq2 comp41118_c0_seq1 comp30637_c0_seq1 comp57708_c1_seq3 comp56722_c1_seq7 comp57103_c0_seq1 comp56093_c0_seq1 comp56756_c4_seq1 comp53836_c0_seq1 comp51884_c0_seq1 comp58048_c4_seq37 comp144394_c0_seq1 comp54577_c0_seq1 comp57603_c3_seq7 comp54394_c1_seq4 comp55605_c0_seq2 comp55190_c0_seq3 comp53383_c1_seq3 comp56178_c1_seq1 comp57961_c1_seq5 comp29351_c0_seq1 comp52299_c0_seq1 comp49655_c0_seq1 comp55604_c2_seq7 comp53071_c1_seq1 comp28227_c0_seq1 comp48226_c0_seq1 comp48604_c1_seq1 comp49567_c0_seq1 comp50167_c0_seq1 comp28942_c0_seq1 comp58075_c0_seq3 comp58037_c4_seq4 comp51576_c1_seq2 comp57975_c2_seq10 comp53631_c0_seq10 comp45915_c1_seq1 comp56298_c4_seq8 comp51939_c0_seq5 comp49883_c0_seq1 comp56246_c1_seq1 comp52799_c0_seq1 comp57345_c4_seq3 comp58159_c0_seq24 comp53746_c0_seq1 comp45474_c1_seq1 comp42208_c0_seq1 comp53083_c0_seq2 comp58001_c1_seq1 comp49510_c1_seq3 comp55392_c0_seq5 comp53501_c0_seq2 comp57856_c15_seq1 comp58280_c1_seq1 comp55145_c1_seq1 comp58575_c0_seq1 comp58136_c0_seq5 comp51679_c4_seq5 comp52925_c0_seq4 comp52382_c3_seq4 comp44825_c0_seq2 comp50549_c0_seq1 comp54668_c0_seq3 comp55278_c2_seq5 comp55157_c0_seq3 comp52544_c0_seq1 comp32251_c0_seq1 comp53890_c1_seq2 comp57127_c0_seq3 comp50982_c0_seq3 comp56422_c2_seq2 comp51565_c0_seq1 comp58139_c1_seq2 comp52887_c0_seq1 comp56630_c4_seq6 comp32751_c0_seq1 comp45101_c1_seq3 comp57659_c1_seq2 comp56451_c0_seq1 comp53108_c0_seq1 comp50303_c0_seq1 comp32240_c0_seq1 comp55466_c1_seq8 comp57520_c0_seq2 comp57676_c0_seq1 comp55593_c1_seq1 comp28163_c0_seq1 comp54494_c2_seq1 comp51299_c1_seq3 comp27967_c0_seq1 comp174322_c0_seq1 comp50569_c1_seq5 comp57899_c2_seq19 comp51412_c0_seq1 comp55270_c0_seq16 comp55849_c0_seq1 comp46270_c0_seq1 comp49495_c0_seq2 comp56567_c1_seq3 comp52341_c0_seq1 comp49546_c1_seq4 comp55120_c0_seq2 comp49540_c0_seq2 comp51655_c1_seq1 comp51954_c0_seq1 comp50036_c0_seq1 comp46279_c0_seq1 comp48856_c0_seq1 comp54614_c0_seq3 comp53253_c0_seq3 comp53685_c0_seq1 comp55774_c3_seq5 comp57729_c2_seq8 comp54169_c0_seq1 comp32310_c2_seq1 comp55689_c1_seq5 comp57792_c0_seq9 comp52582_c6_seq1 comp56941_c0_seq6 comp49285_c0_seq1 comp50985_c0_seq1 comp58277_c0_seq11 comp48146_c0_seq1 comp56062_c5_seq5 comp57783_c0_seq6 comp55556_c0_seq1 comp56595_c0_seq15 comp54354_c0_seq1 comp53996_c1_seq2 comp56620_c3_seq2 comp57601_c0_seq2 comp57624_c2_seq9 comp58291_c0_seq10 comp53191_c0_seq3 comp54139_c2_seq5 comp57263_c1_seq1 comp43852_c0_seq1 comp57464_c0_seq3 comp56276_c0_seq2 comp57789_c0_seq13 comp50637_c1_seq1 comp29114_c0_seq1 comp54549_c1_seq7 comp46290_c1_seq2 comp52171_c1_seq1 comp52220_c1_seq1 comp44908_c3_seq1 comp54840_c4_seq12 comp55982_c0_seq4 comp52143_c0_seq2 comp32637_c0_seq1 comp57737_c0_seq5 comp29356_c0_seq1 comp31126_c0_seq1 comp53463_c1_seq4 comp58021_c3_seq11 comp53950_c0_seq4 comp4386_c0_seq1 comp31584_c0_seq1 comp50634_c0_seq2 comp57837_c1_seq3 comp57008_c0_seq21 comp43188_c0_seq1 comp55260_c1_seq1 comp57471_c2_seq4 comp52500_c1_seq1 comp53146_c0_seq4 comp32525_c0_seq1 comp54065_c0_seq3 comp53366_c0_seq3 comp50926_c0_seq1 comp48510_c1_seq1 comp57620_c3_seq7 comp55150_c4_seq1 comp56127_c0_seq2 comp55556_c0_seq6 comp41850_c0_seq1 comp46831_c4_seq1 comp58001_c3_seq6 comp52735_c0_seq1 comp55997_c0_seq5 comp54211_c0_seq1 comp61687_c0_seq1 comp56516_c2_seq6 comp53290_c0_seq1 comp48313_c3_seq5 comp48629_c0_seq2 comp51540_c0_seq2 comp55747_c1_seq10 comp55039_c1_seq1 comp48584_c1_seq1 comp54036_c0_seq2 comp42650_c1_seq1 comp54703_c2_seq8 comp61053_c0_seq1 comp46028_c0_seq1 comp55939_c2_seq3 comp55512_c1_seq2 comp43874_c0_seq1 comp56952_c6_seq33 comp56094_c0_seq2 comp57495_c7_seq1 comp55184_c5_seq2 comp56939_c0_seq10 comp49766_c0_seq1 comp48674_c0_seq1 comp56931_c1_seq1 comp56967_c1_seq3 comp59448_c0_seq1 comp55103_c0_seq9 comp56357_c0_seq1 comp51154_c0_seq1 comp56457_c0_seq2 comp56920_c5_seq2 comp50054_c0_seq5 comp55687_c0_seq5 comp46886_c1_seq1 comp56592_c3_seq1 comp44879_c0_seq2 comp43179_c0_seq1 comp57295_c0_seq2 comp57511_c0_seq7 comp48742_c0_seq3 comp58267_c1_seq8 comp50981_c0_seq1 comp49457_c0_seq1 comp29265_c0_seq1 comp57625_c4_seq20 comp50638_c1_seq1 comp58257_c0_seq10 comp55748_c0_seq1 comp53089_c0_seq5 comp57232_c1_seq1 comp56336_c0_seq4 comp44339_c0_seq2 comp59945_c0_seq1 comp54761_c0_seq1 comp30860_c0_seq1 comp56278_c0_seq8 comp51045_c0_seq1 comp53199_c1_seq1 comp57958_c0_seq3 comp49659_c0_seq3 comp35267_c0_seq1 comp54172_c0_seq2 comp57462_c0_seq7 comp53560_c2_seq3 comp57739_c6_seq3 comp52924_c0_seq1 comp46142_c0_seq2 comp52488_c0_seq1 comp55581_c1_seq8 comp57145_c3_seq4 comp55944_c5_seq1 comp55525_c1_seq14 comp48930_c1_seq3 comp36332_c0_seq1 comp52191_c0_seq2 comp54165_c3_seq5 comp51614_c1_seq2 comp54707_c1_seq4 comp43808_c1_seq1 comp42835_c0_seq1 comp50545_c1_seq1 comp37610_c0_seq2 comp58065_c7_seq7 comp56502_c0_seq1 comp52524_c0_seq7 comp50663_c1_seq1 comp50755_c1_seq2 comp31865_c1_seq1 comp50441_c1_seq1 comp55242_c0_seq3 comp53710_c1_seq6 comp52747_c1_seq1 comp56225_c0_seq2 comp48758_c0_seq1 comp55294_c0_seq1 comp50709_c0_seq2 comp56228_c3_seq1 comp57584_c0_seq5 comp58205_c5_seq1 comp57699_c1_seq7 comp55597_c1_seq5 comp54558_c0_seq1 comp66567_c0_seq1 comp57771_c0_seq10 comp43604_c0_seq1 comp55160_c1_seq1 comp52453_c2_seq5 comp51564_c0_seq1 comp54064_c1_seq3 comp44644_c0_seq1 comp52901_c0_seq1 comp53403_c0_seq3 comp57112_c1_seq1 comp51587_c2_seq1 comp56986_c0_seq2 comp50918_c0_seq1 comp48436_c0_seq2 comp54587_c0_seq1 comp59961_c0_seq1 comp57122_c0_seq2 comp56180_c0_seq2 comp53384_c0_seq5 comp46681_c0_seq1 comp56376_c0_seq2 comp56082_c0_seq1 comp46849_c1_seq1 comp29095_c0_seq2 comp55451_c1_seq3 comp28955_c0_seq1 comp57658_c6_seq9 comp57078_c2_seq1 comp51595_c0_seq1 comp53782_c0_seq1 comp55196_c0_seq5 comp56084_c3_seq4 comp43271_c0_seq1 comp46057_c0_seq3 comp30122_c0_seq1 comp43919_c0_seq1 comp56228_c1_seq2 comp36075_c1_seq1 comp41761_c1_seq1 comp57432_c0_seq5 comp57532_c3_seq14 comp57369_c1_seq10 comp27927_c0_seq1 comp56952_c6_seq24 comp48681_c0_seq2 comp31203_c1_seq1 comp57254_c9_seq5 comp48796_c2_seq4 comp58109_c1_seq1 comp50040_c0_seq3 comp49491_c2_seq1 comp51400_c1_seq4 comp41437_c0_seq1 comp51274_c0_seq4 comp47512_c0_seq1 comp55141_c0_seq1 comp45482_c1_seq1 comp46281_c0_seq1 comp54606_c0_seq4 comp52438_c0_seq2 comp55970_c0_seq1 comp45730_c2_seq1 comp56447_c9_seq2 comp57309_c0_seq1 comp48221_c1_seq1 comp48671_c1_seq2 comp44036_c0_seq1 comp44010_c0_seq1 comp56472_c0_seq6 comp52463_c0_seq2 comp47282_c0_seq1 comp53806_c0_seq2 comp53014_c2_seq2 comp55963_c1_seq15 comp47258_c0_seq1 comp51066_c0_seq1 comp30070_c0_seq1 comp53499_c2_seq4 comp39438_c0_seq1 comp30283_c0_seq2 comp57923_c0_seq1 comp53284_c0_seq1 comp56905_c0_seq4 comp56369_c0_seq2 comp51490_c0_seq1 comp55762_c4_seq12 comp50313_c1_seq1 comp47916_c0_seq2 comp58202_c2_seq5 comp32151_c0_seq1 comp57583_c0_seq1 comp58120_c1_seq3 comp55640_c1_seq2 comp49589_c0_seq4 comp51194_c1_seq1 comp52424_c0_seq2 comp57124_c1_seq18 comp45941_c0_seq1 comp56341_c3_seq12 comp33813_c0_seq2 comp50906_c2_seq5 comp50815_c1_seq3 comp151746_c0_seq1 comp46737_c0_seq1 comp54193_c11_seq13 comp57975_c2_seq3 comp55267_c0_seq1 comp50141_c1_seq3 comp49989_c1_seq5 comp50477_c0_seq2 comp56534_c1_seq10 comp53102_c1_seq3 comp58023_c1_seq4 comp45743_c0_seq1 comp54120_c6_seq1 comp56259_c1_seq2 comp50240_c1_seq1 comp54075_c2_seq1 comp58236_c0_seq15 comp53431_c2_seq10 comp33121_c0_seq1 comp49666_c2_seq1 comp38302_c0_seq1 comp51166_c1_seq1 comp43283_c0_seq1 comp43768_c0_seq1 comp49886_c0_seq1 comp57544_c0_seq8 comp48794_c2_seq1 comp58173_c0_seq10 comp43588_c0_seq1 comp57057_c0_seq1 comp54280_c1_seq4 comp49783_c0_seq6 comp56317_c0_seq1 comp53079_c0_seq1 comp53972_c0_seq1 comp55059_c4_seq1 comp53002_c0_seq6 comp55136_c0_seq2 comp55084_c0_seq4 comp51320_c0_seq2 comp49756_c0_seq1 comp46413_c2_seq1 comp56361_c2_seq9 comp56628_c1_seq6 comp55964_c0_seq9 comp55032_c0_seq4 comp50586_c0_seq8 comp32958_c0_seq1 comp53310_c0_seq5 comp57175_c0_seq1 comp57833_c0_seq13 comp35235_c1_seq1 comp55092_c0_seq1 comp57264_c0_seq1 comp55886_c9_seq8 comp52154_c0_seq1 comp54464_c0_seq3 comp44858_c2_seq1 comp53687_c0_seq1 comp50159_c0_seq1 comp49023_c0_seq2 comp47751_c0_seq1 comp57499_c6_seq32 comp49006_c0_seq2 comp54044_c0_seq1 comp54193_c11_seq17 comp58145_c0_seq46 comp53963_c1_seq1 comp54550_c0_seq3 comp51234_c0_seq1 comp58071_c2_seq4 comp52566_c0_seq4 comp55807_c1_seq2 comp55872_c0_seq1 comp54391_c1_seq3 comp57972_c0_seq12 comp55491_c3_seq3 comp30633_c0_seq1 comp48154_c1_seq2 comp46009_c0_seq1 comp31123_c0_seq1 comp54938_c0_seq2 comp56111_c2_seq1 comp52582_c6_seq5 comp55731_c1_seq4 comp55557_c0_seq8 comp46078_c0_seq2 comp54872_c0_seq1 comp53283_c0_seq1 comp32247_c0_seq1 comp53688_c0_seq3 comp50244_c2_seq1 comp75883_c0_seq1 comp46712_c0_seq1 comp48265_c0_seq4 comp46958_c1_seq2 comp57824_c3_seq1 comp58188_c0_seq2 comp58140_c7_seq4 comp57604_c1_seq16 comp56394_c1_seq5 comp55245_c1_seq2 comp57078_c0_seq4 comp51727_c0_seq1 comp53165_c1_seq3 comp169057_c0_seq1 comp49955_c0_seq1 comp56218_c3_seq1 comp45853_c0_seq1 comp57977_c0_seq5 comp38415_c0_seq1 comp55886_c6_seq2 comp55911_c4_seq1 comp54397_c3_seq5 comp56874_c4_seq40 comp49314_c2_seq4 comp56994_c1_seq3 comp42493_c0_seq1 comp56849_c4_seq5 comp55784_c0_seq1 comp56123_c1_seq1 comp51259_c2_seq3 comp48309_c0_seq1 comp56710_c2_seq4 comp55827_c2_seq8 comp57844_c1_seq1 comp57896_c0_seq1 comp52968_c0_seq2 comp58041_c0_seq8 comp51757_c3_seq1 comp55164_c0_seq6 comp48310_c0_seq1 comp96332_c0_seq1 comp57338_c1_seq4 comp53940_c0_seq5 comp52254_c0_seq4 comp57996_c5_seq12 comp56898_c0_seq8 comp58076_c3_seq1 comp50896_c1_seq5 comp36042_c0_seq1 comp57931_c0_seq4 comp57244_c0_seq4 comp51158_c2_seq1 comp245251_c0_seq1 comp51893_c1_seq5 comp58103_c5_seq1 comp56987_c9_seq4 comp57228_c0_seq5 comp52711_c1_seq1 comp57810_c0_seq4 comp51467_c0_seq3 comp46944_c0_seq1 comp58087_c9_seq1 comp51620_c0_seq2 comp57134_c1_seq1 comp57308_c1_seq2 comp58318_c0_seq1 comp56262_c0_seq2 comp57324_c1_seq9 comp54174_c2_seq2 comp56141_c2_seq23 comp53803_c1_seq1 comp50883_c0_seq2 comp53917_c0_seq2 comp58169_c0_seq11 comp55136_c0_seq6 comp48933_c0_seq2 comp50009_c0_seq1 comp49083_c0_seq1 comp51092_c0_seq2 comp50734_c1_seq1 comp49514_c0_seq2 comp51647_c0_seq1 comp55697_c1_seq1 comp50384_c1_seq2 comp57848_c0_seq7 comp58114_c2_seq10 comp54086_c0_seq6 comp52151_c1_seq1 comp57671_c3_seq6 comp57048_c0_seq3 comp50544_c0_seq5 comp49426_c0_seq2 comp47109_c0_seq1 comp49894_c0_seq2 comp52142_c5_seq3 comp44395_c0_seq1 comp57983_c0_seq7 comp41257_c0_seq2 comp42561_c0_seq1 comp55199_c1_seq6 comp55636_c0_seq2 comp56793_c0_seq2 comp57326_c5_seq17 comp52329_c1_seq2 comp57921_c0_seq6 comp41677_c0_seq1 comp33552_c0_seq2 comp32731_c0_seq1 comp57643_c3_seq2 comp46464_c0_seq1 comp56440_c0_seq11 comp55505_c3_seq7 comp54840_c4_seq4 comp56948_c0_seq2 comp57526_c0_seq2 comp45615_c1_seq1 comp56116_c0_seq1 comp54313_c0_seq4 comp54165_c1_seq1 comp51250_c0_seq1 comp55543_c0_seq4 comp55901_c0_seq2 comp54830_c2_seq16 comp57667_c1_seq13 comp57558_c4_seq4 comp58271_c2_seq7 comp48833_c0_seq1 comp53480_c1_seq1 comp30604_c0_seq1 comp55873_c0_seq2 comp29768_c0_seq1 comp43867_c0_seq1 comp53367_c0_seq1 comp51254_c0_seq2 comp55493_c0_seq11 comp58050_c3_seq1 comp50208_c3_seq3 comp41606_c0_seq1 comp58235_c0_seq2 comp30107_c0_seq1 comp57118_c0_seq3 comp53576_c0_seq1 comp57278_c4_seq2 comp54067_c0_seq1 comp44219_c0_seq1 comp61558_c0_seq1 comp43946_c0_seq3 comp58204_c0_seq5 comp55861_c1_seq1 comp54556_c1_seq1 comp51237_c0_seq1 comp56571_c0_seq7 comp53551_c2_seq1 comp54465_c0_seq3 comp50406_c0_seq2 comp56992_c2_seq8 comp57617_c7_seq3 comp57569_c1_seq27 comp48985_c0_seq2 comp50442_c0_seq1 comp55514_c1_seq4 comp56920_c0_seq6 comp51906_c0_seq1 comp58176_c1_seq10 comp53729_c0_seq1 comp52417_c0_seq1 comp54864_c5_seq2 comp57639_c12_seq5 comp57547_c1_seq2 comp59581_c0_seq1 comp57227_c1_seq9 comp55900_c8_seq53 comp58100_c1_seq7 comp56765_c0_seq10 comp55478_c2_seq1 comp49645_c1_seq1 comp56304_c4_seq1 comp57368_c10_seq1 comp58079_c3_seq3 comp54435_c1_seq3 comp53894_c0_seq1 comp57544_c6_seq12 comp58223_c6_seq39 comp57346_c3_seq13 comp57552_c2_seq4 comp52886_c1_seq4 comp57879_c0_seq1 comp56384_c0_seq3 comp48190_c1_seq2 comp57563_c1_seq8 comp50302_c2_seq1 comp56909_c0_seq2 comp52826_c0_seq2 comp51572_c1_seq3 comp50947_c1_seq2 comp58001_c1_seq2 comp42675_c0_seq1 comp52131_c1_seq5 comp44904_c0_seq1 comp57446_c0_seq12 comp50281_c0_seq1 comp52380_c0_seq4 comp54432_c0_seq2 comp40760_c0_seq1 comp52720_c2_seq1 comp51923_c0_seq1 comp57112_c0_seq4 comp51857_c1_seq6 comp53562_c2_seq11 comp210562_c0_seq1 comp47589_c4_seq1 comp52635_c0_seq3 comp48865_c0_seq1 comp56018_c1_seq3 comp56691_c1_seq1 comp56704_c2_seq2 comp58240_c1_seq16 comp50894_c1_seq1 comp57660_c0_seq4 comp55889_c0_seq8 comp48005_c0_seq1 comp57054_c1_seq19 comp4575_c0_seq1 comp54378_c3_seq1 comp49792_c0_seq3 comp57982_c1_seq8 comp49485_c0_seq1 comp57818_c2_seq1 comp58156_c0_seq4 comp45213_c0_seq1 comp55959_c0_seq4 comp58095_c0_seq5 comp44499_c0_seq2 comp50869_c0_seq2 comp50705_c0_seq1 comp43363_c0_seq1 comp42645_c0_seq1 comp56564_c0_seq6 comp33757_c1_seq1 comp55869_c1_seq4 comp57780_c7_seq1 comp57807_c12_seq2 comp55976_c1_seq2 comp57659_c1_seq6 comp49700_c0_seq1 comp49266_c1_seq1 comp51836_c0_seq1 comp51169_c0_seq3 comp49049_c0_seq2 comp45931_c0_seq1 comp50357_c1_seq1 comp56322_c13_seq1 comp45033_c0_seq1 comp54672_c0_seq1 comp51613_c0_seq9 comp51605_c0_seq1 comp55884_c0_seq2 comp56808_c0_seq4 comp52699_c0_seq2 comp56239_c2_seq6 comp45307_c0_seq1 comp48173_c0_seq1 comp57900_c5_seq1 comp57290_c1_seq10 comp57076_c0_seq4 comp54394_c4_seq12 comp53942_c1_seq5 comp51055_c2_seq3 comp50636_c1_seq1 comp55593_c0_seq1 comp56059_c0_seq5 comp51257_c0_seq4 comp53381_c1_seq2 comp57534_c0_seq1 comp58075_c0_seq2 comp50987_c0_seq1 comp49789_c1_seq1 comp52702_c1_seq1 comp56595_c0_seq12 comp50001_c1_seq1 comp52882_c2_seq2 comp56938_c5_seq1 comp58222_c2_seq19 comp54387_c1_seq2 comp46326_c2_seq1 comp50813_c0_seq4 comp47135_c0_seq1 comp48110_c0_seq1 comp47912_c0_seq1 comp57468_c0_seq4 comp53110_c2_seq1 comp50729_c0_seq1 comp52552_c0_seq2 comp48984_c0_seq1 comp32915_c0_seq1 comp56290_c1_seq4 comp51416_c4_seq4 comp52831_c4_seq8 comp57713_c0_seq5 comp57909_c0_seq4 comp52464_c0_seq2 comp45688_c0_seq1 comp53243_c0_seq2 comp55635_c1_seq1 comp56221_c0_seq8 comp53734_c0_seq5 comp30806_c0_seq2 comp54378_c6_seq3 comp113803_c0_seq1 comp37269_c1_seq1 comp51957_c2_seq1 comp56521_c4_seq6 comp53072_c2_seq4 comp52512_c3_seq2 comp49737_c0_seq1 comp45738_c0_seq1 comp58058_c0_seq2 comp56859_c1_seq2 comp57617_c6_seq2 comp56883_c1_seq1 comp57738_c0_seq6 comp57999_c1_seq7 comp57618_c0_seq15 comp52580_c1_seq1 comp57307_c0_seq1 comp52668_c4_seq4 comp55929_c2_seq1 comp49421_c0_seq1 comp54458_c6_seq1 comp47251_c0_seq4 comp54798_c1_seq1 comp52399_c0_seq1 comp62570_c0_seq1 comp51915_c0_seq2 comp55607_c0_seq2 comp56660_c1_seq2 comp57687_c1_seq3 comp55256_c4_seq3 comp53909_c1_seq1 comp55087_c0_seq3 comp54857_c0_seq4 comp57856_c15_seq11 comp58277_c0_seq12 comp51546_c0_seq2 comp67207_c0_seq1 comp56952_c6_seq3 comp54113_c0_seq4 comp56391_c3_seq14 comp50511_c0_seq4 comp56540_c0_seq19 comp44069_c0_seq2 comp54696_c1_seq3 comp51940_c2_seq1 comp57473_c0_seq22 comp28512_c0_seq1 comp56906_c0_seq3 comp53059_c0_seq1 comp52193_c0_seq3 comp50210_c0_seq1 comp50939_c0_seq1 comp41661_c0_seq1 comp51593_c0_seq3 comp52385_c0_seq2 comp57533_c1_seq4 comp51440_c1_seq1 comp109806_c0_seq1 comp56691_c1_seq5 comp46282_c0_seq3 comp54839_c0_seq1 comp52753_c0_seq2 comp31747_c0_seq1 comp56979_c5_seq5 comp53764_c4_seq7 comp57780_c6_seq3 comp51149_c1_seq1 comp50237_c0_seq1 comp53997_c0_seq1 comp29821_c0_seq2 comp29492_c0_seq1 comp47091_c0_seq1 comp29529_c0_seq1 comp49973_c0_seq6 comp53790_c0_seq1 comp52384_c1_seq1 comp56239_c2_seq12 comp53628_c0_seq1 comp53459_c0_seq2 comp58134_c0_seq4 comp49862_c0_seq2 comp55687_c1_seq2 comp53718_c0_seq1 comp58197_c7_seq6 comp48919_c0_seq1 comp56129_c0_seq1 comp56797_c2_seq4 comp51801_c1_seq9 comp57373_c0_seq4 comp30928_c0_seq1 comp53955_c2_seq6 comp57281_c0_seq2 comp57239_c2_seq22 comp57296_c0_seq14 comp55411_c1_seq3 comp56166_c1_seq1 comp46634_c0_seq1 comp50474_c0_seq6 comp46045_c0_seq2 comp51528_c0_seq1 comp55356_c0_seq4 comp48424_c0_seq1 comp56907_c3_seq1 comp57504_c2_seq23 comp56993_c0_seq5 comp53332_c0_seq3 comp57494_c7_seq7 comp46453_c0_seq1 comp48223_c0_seq2 comp56304_c1_seq1 comp56152_c0_seq10 comp55198_c1_seq4 comp45200_c0_seq1 comp54100_c0_seq2 comp52831_c0_seq8 comp36103_c0_seq1 comp50714_c1_seq3 comp57992_c0_seq2 comp45617_c0_seq1 comp55779_c0_seq6 comp55213_c0_seq1 comp52220_c0_seq2 comp57663_c0_seq11 comp54434_c8_seq1 comp52126_c0_seq1 comp57341_c0_seq7 comp53547_c1_seq2 comp43143_c0_seq1 comp51576_c1_seq1 comp50637_c0_seq1 comp57909_c0_seq7 comp55492_c1_seq2 comp55048_c0_seq2 comp56192_c4_seq10 comp56734_c2_seq1 comp48245_c1_seq1 comp58006_c7_seq2 comp42122_c0_seq1 comp52477_c1_seq2 comp51629_c3_seq2 comp53499_c1_seq1 comp53164_c0_seq2 comp47436_c0_seq1 comp54740_c0_seq3 comp55323_c0_seq2 comp46914_c2_seq1 comp57124_c1_seq3 comp52051_c0_seq2 comp54102_c0_seq1 comp55969_c0_seq5 comp52758_c0_seq1 comp42595_c0_seq2 comp53928_c0_seq3 comp58281_c4_seq5 comp54360_c2_seq1 comp55762_c4_seq3 comp48541_c0_seq1 comp55774_c4_seq4 comp56380_c0_seq4 comp56049_c0_seq4 comp30766_c0_seq1 comp39665_c0_seq1 comp44564_c0_seq2 comp51095_c1_seq3 comp57991_c2_seq11 comp56192_c4_seq3 comp46860_c1_seq1 comp49200_c1_seq1 comp52310_c0_seq2 comp57539_c1_seq9 comp58145_c0_seq51 comp55986_c3_seq5 comp58191_c1_seq4 comp51458_c0_seq2 comp48196_c0_seq1 comp28339_c0_seq1 comp54983_c0_seq1 comp56987_c0_seq4 comp50126_c0_seq1 comp58278_c0_seq5 comp54084_c4_seq3 comp48245_c0_seq2 comp58296_c0_seq6 comp53147_c2_seq1 comp46449_c0_seq2 comp47628_c0_seq2 comp48449_c0_seq1 comp56304_c2_seq4 comp42991_c0_seq2 comp55253_c2_seq2 comp51225_c1_seq3 comp57824_c1_seq5 comp50509_c0_seq1 comp50169_c0_seq1 comp177068_c0_seq1 comp52320_c0_seq12 comp54296_c2_seq5 comp57402_c3_seq5 comp54408_c0_seq2 comp58084_c0_seq6 comp51256_c0_seq1 comp47343_c2_seq1 comp47192_c1_seq2 comp56408_c0_seq1 comp56428_c4_seq1 comp57123_c0_seq3 comp49920_c0_seq2 comp55034_c1_seq5 comp57099_c0_seq6 comp49061_c0_seq1 comp57916_c1_seq2 comp54382_c0_seq2 comp46377_c0_seq1 comp41193_c0_seq1 comp48588_c0_seq3 comp54706_c1_seq10 comp46178_c0_seq2 comp45261_c1_seq3 comp56035_c0_seq3 comp54881_c0_seq2 comp54112_c0_seq1 comp57727_c0_seq4 comp51856_c1_seq3 comp52767_c0_seq2 comp52192_c0_seq1 comp51899_c0_seq1 comp56357_c4_seq14 comp49924_c0_seq1 comp52496_c0_seq2 comp49251_c0_seq1 comp49972_c0_seq4 comp56624_c0_seq1 comp55344_c1_seq2 comp56171_c0_seq1 comp54756_c0_seq5 comp197121_c0_seq1 comp50166_c1_seq5 comp49889_c0_seq1 comp56783_c3_seq1 comp52600_c0_seq4 comp43795_c0_seq1 comp52383_c0_seq4 comp58189_c0_seq3 comp48204_c1_seq3 comp57927_c3_seq9 comp45511_c1_seq1 comp55445_c0_seq1 comp47306_c1_seq1 comp53779_c2_seq2 comp40935_c0_seq1 comp48760_c1_seq4 comp56159_c0_seq6 comp48142_c0_seq2 comp57017_c0_seq4 comp48135_c2_seq1 comp55790_c3_seq1 comp56580_c0_seq3 comp50278_c0_seq2 comp52957_c2_seq1 comp55709_c1_seq3 comp56182_c0_seq9 comp56653_c1_seq8 comp27932_c0_seq1 comp49760_c0_seq2 comp51450_c1_seq1 comp55562_c1_seq1 comp51923_c0_seq4 comp44896_c0_seq1 comp42204_c0_seq1 comp48236_c3_seq1 comp56658_c0_seq5 comp46523_c2_seq1 comp56884_c1_seq6 comp57242_c2_seq4 comp58024_c0_seq3 comp47082_c0_seq1 comp56827_c0_seq5 comp29069_c1_seq1 comp51092_c0_seq6 comp55434_c0_seq3 comp51106_c1_seq5 comp52077_c4_seq7 comp52647_c1_seq2 comp50127_c2_seq3 comp49648_c1_seq4 comp46636_c0_seq2 comp55473_c0_seq3 comp54678_c2_seq6 comp55548_c1_seq17 comp57976_c0_seq3 comp55659_c0_seq1 comp55941_c0_seq1 comp45794_c0_seq2 comp56426_c2_seq1 comp52139_c0_seq1 comp48448_c0_seq1 comp50391_c0_seq1 comp47793_c0_seq1 comp52314_c0_seq1 comp52835_c1_seq3 comp53055_c1_seq2 comp54348_c1_seq2 comp58191_c1_seq14 comp55248_c1_seq11 comp58335_c0_seq1 comp58263_c1_seq17 comp47296_c0_seq3 comp54333_c0_seq3 comp49424_c0_seq2 comp54505_c0_seq2 comp50754_c0_seq6 comp46761_c2_seq1 comp292092_c0_seq1 comp55787_c2_seq2 comp49185_c0_seq1 comp56703_c3_seq5 comp58108_c1_seq11 comp57405_c0_seq1 comp49757_c0_seq2 comp51020_c1_seq1 comp53210_c0_seq1 comp47304_c0_seq1 comp51101_c0_seq3 comp46565_c0_seq1 comp54401_c8_seq4 comp54301_c0_seq1 comp39384_c1_seq1 comp53360_c5_seq3 comp50974_c1_seq3 comp56241_c0_seq1 comp52010_c0_seq2 comp56217_c0_seq6 comp46259_c0_seq1 comp56748_c2_seq5 comp57644_c0_seq4 comp53496_c0_seq3 comp57209_c0_seq6 comp53922_c0_seq4 comp50879_c0_seq4 comp51542_c0_seq6 comp52958_c0_seq1 comp47981_c0_seq2 comp55454_c1_seq8 comp57092_c1_seq9 comp31940_c0_seq1 comp41808_c0_seq1 comp56322_c14_seq1 comp51570_c1_seq1 comp47880_c0_seq1 comp54401_c8_seq15 comp57880_c2_seq6 comp57263_c0_seq3 comp52741_c0_seq2 comp58169_c0_seq10 comp49450_c0_seq1 comp29369_c0_seq1 comp57407_c1_seq15 comp56774_c1_seq1 comp55946_c0_seq2 comp48373_c0_seq1 comp47713_c0_seq1 comp55370_c0_seq7 comp46255_c0_seq2 comp41428_c0_seq1 comp56119_c1_seq4 comp58076_c5_seq1 comp56197_c4_seq12 comp50580_c1_seq1 comp56675_c0_seq3 comp58089_c2_seq2 comp46383_c0_seq6 comp51392_c0_seq2 comp49293_c0_seq3 comp52377_c0_seq2 comp48534_c0_seq3 comp35458_c1_seq4 comp47955_c3_seq2 comp54785_c1_seq22 comp54414_c1_seq6 comp52354_c0_seq4 comp55097_c3_seq1 comp49268_c0_seq1 comp47742_c2_seq1 comp42700_c0_seq1 comp53610_c3_seq1 comp56848_c0_seq6 comp47763_c0_seq1 comp57753_c1_seq1 comp48573_c0_seq1 comp51904_c0_seq1 comp56785_c5_seq2 comp56020_c2_seq4 comp57488_c3_seq7 comp55845_c0_seq1 comp47180_c0_seq3 comp56141_c2_seq4 comp50611_c0_seq1 comp42926_c0_seq1 comp57489_c0_seq3 comp51811_c0_seq4 comp29118_c0_seq1 comp57696_c0_seq4 comp53112_c2_seq1 comp55846_c0_seq5 comp57091_c9_seq4 comp198923_c0_seq1 comp53163_c0_seq1 comp41452_c0_seq2 comp57427_c1_seq2 comp55757_c0_seq4 comp53892_c2_seq1 comp57777_c0_seq1 comp57023_c1_seq25 comp45798_c0_seq1 comp58192_c0_seq17 comp51033_c3_seq1 comp53985_c0_seq5 comp57899_c2_seq7 comp56314_c1_seq2 comp50722_c5_seq1 comp53220_c0_seq1 comp51607_c0_seq1 comp42593_c0_seq1 comp48535_c0_seq1 comp56339_c0_seq2 comp55089_c1_seq1 comp38974_c1_seq1 comp42995_c0_seq1 comp46388_c0_seq1 comp55385_c0_seq2 comp46124_c0_seq4 comp53786_c2_seq4 comp54664_c0_seq2 comp50712_c0_seq2 comp53649_c0_seq2 comp57878_c0_seq4 comp48010_c0_seq1 comp46147_c0_seq1 comp51619_c0_seq1 comp49504_c0_seq5 comp29673_c0_seq1 comp30885_c0_seq1 comp56429_c0_seq14 comp53229_c0_seq3 comp48983_c1_seq1 comp56175_c0_seq10 comp56453_c2_seq1 comp59093_c0_seq1 comp29888_c0_seq2 comp35876_c0_seq1 comp55571_c2_seq2 comp52212_c0_seq2 comp55503_c6_seq3 comp53401_c1_seq8 comp54810_c3_seq3 comp56537_c0_seq2 comp56047_c0_seq1 comp54840_c1_seq1 comp49393_c0_seq1 comp55773_c3_seq1 comp55519_c0_seq1 comp53426_c2_seq1 comp55723_c2_seq2 comp47043_c0_seq1 comp54437_c0_seq3 comp45968_c0_seq1 comp32882_c0_seq1 comp57926_c8_seq16 comp54937_c0_seq1 comp51717_c0_seq2 comp59493_c0_seq1 comp55995_c4_seq4 comp44671_c4_seq1 comp54916_c0_seq1 comp57647_c3_seq5 comp57428_c0_seq1 comp56035_c0_seq1 comp47140_c2_seq1 comp57371_c0_seq2 comp31510_c0_seq1 comp38522_c0_seq1 comp56451_c0_seq4 comp54903_c1_seq4 comp56200_c0_seq20 comp57792_c0_seq4 comp50683_c0_seq2 comp57054_c1_seq22 comp56286_c1_seq4 comp57989_c0_seq9 comp49418_c0_seq4 comp51343_c0_seq3 comp53369_c0_seq1 comp64151_c0_seq1 comp52389_c0_seq6 comp46270_c1_seq1 comp51990_c2_seq19 comp55513_c2_seq1 comp53580_c1_seq3 comp50212_c4_seq1 comp50679_c0_seq2 comp52640_c0_seq2 comp56860_c3_seq14 comp31480_c0_seq1 comp57784_c1_seq1 comp53575_c0_seq1 comp56576_c1_seq3 comp55184_c15_seq1 comp52229_c1_seq2 comp32536_c0_seq1 comp53104_c0_seq5 comp57170_c1_seq5 comp39017_c0_seq1 comp53452_c2_seq1 comp38396_c0_seq2 comp54514_c0_seq4 comp52510_c0_seq2 comp56783_c1_seq1 comp55634_c3_seq1 comp57351_c3_seq16 comp58253_c1_seq3 comp47976_c0_seq1 comp54278_c0_seq3 comp51686_c3_seq2 comp58111_c3_seq33 comp38213_c0_seq1 comp57235_c2_seq2 comp54006_c0_seq7 comp57944_c1_seq1 comp55230_c1_seq5 comp58031_c2_seq20 comp51771_c0_seq1 comp54416_c0_seq13 comp57946_c6_seq5 comp51834_c0_seq4 comp55071_c0_seq4 comp53441_c0_seq1 comp47855_c1_seq2 comp56870_c0_seq2 comp55873_c0_seq9 comp4355_c0_seq1 comp56634_c3_seq10 comp43126_c0_seq1 comp58071_c2_seq10 comp55688_c1_seq1 comp55543_c0_seq9 comp57647_c3_seq1 comp51875_c0_seq2 comp46080_c0_seq1 comp56415_c0_seq8 comp45455_c0_seq1 comp57297_c1_seq4 comp41259_c0_seq1 comp58059_c7_seq3 comp56040_c1_seq6 comp55584_c0_seq4 comp57080_c0_seq3 comp53598_c0_seq1 comp54674_c0_seq1 comp55506_c0_seq2 comp54233_c0_seq6 comp55624_c0_seq6 comp44723_c0_seq3 comp57916_c1_seq11 comp56943_c0_seq2 comp54620_c1_seq1 comp55744_c3_seq1 comp55414_c5_seq20 comp50627_c1_seq1 comp56299_c2_seq7 comp53675_c1_seq2 comp53299_c0_seq2 comp49647_c0_seq1 comp50859_c3_seq1 comp58202_c2_seq10 comp57123_c0_seq5 comp48608_c0_seq2 comp55670_c0_seq14 comp57669_c0_seq41 comp55744_c9_seq4 comp34540_c0_seq3 comp55788_c0_seq2 comp56798_c1_seq10 comp43016_c0_seq1 comp49262_c1_seq1 comp50668_c2_seq1 comp50285_c1_seq1 comp57395_c2_seq5 comp44810_c0_seq1 comp55827_c2_seq10 comp57838_c0_seq3 comp58403_c0_seq1 comp54042_c0_seq2 comp56286_c1_seq1 comp48981_c0_seq4 comp33813_c0_seq1 comp36205_c1_seq1 comp57640_c1_seq12 comp56397_c0_seq8 comp57240_c1_seq7 comp56858_c3_seq3 comp46144_c0_seq2 comp46140_c0_seq2 comp52570_c0_seq2 comp57076_c0_seq1 comp57862_c4_seq2 comp54483_c0_seq1 comp56196_c2_seq3 comp58086_c1_seq28 comp45146_c0_seq2 comp55205_c0_seq3 comp56357_c4_seq20 comp55413_c3_seq2 comp52366_c0_seq1 comp49909_c0_seq3 comp51476_c0_seq3 comp58258_c0_seq9 comp53019_c0_seq2 comp56726_c2_seq21 comp55187_c0_seq2 comp56978_c0_seq2 comp56447_c3_seq2 comp48084_c0_seq3 comp52558_c4_seq5 comp55175_c0_seq3 comp55886_c8_seq2 comp57346_c3_seq7 comp56576_c1_seq7 comp53825_c0_seq1 comp52507_c0_seq2 comp49100_c0_seq6 comp55139_c1_seq3 comp56119_c1_seq1 comp49523_c0_seq2 comp47408_c1_seq2 comp56378_c3_seq4 comp57363_c1_seq15 comp57019_c0_seq3 comp51516_c0_seq1 comp55483_c1_seq1 comp52280_c1_seq3 comp58274_c2_seq20 comp175237_c0_seq1 comp46803_c0_seq1 comp55637_c0_seq2 comp56290_c1_seq2 comp55066_c0_seq6 comp58230_c0_seq6 comp53181_c1_seq3 comp52500_c3_seq2 comp30426_c0_seq2 comp52151_c0_seq1 comp55278_c2_seq6 comp58118_c3_seq2 comp43887_c0_seq5 comp56192_c4_seq4 comp50201_c3_seq1 comp51689_c0_seq4 comp298670_c0_seq1 comp29879_c0_seq1 comp49843_c0_seq1 comp58086_c1_seq20 comp58215_c2_seq4 comp56367_c2_seq3 comp49257_c1_seq1 comp52472_c0_seq5 comp49314_c2_seq3 comp49818_c0_seq1 comp46840_c0_seq2 comp46889_c0_seq1 comp50663_c2_seq2 comp53028_c0_seq2 comp56784_c2_seq4 comp44323_c0_seq1 comp53541_c0_seq2 comp54960_c2_seq10 comp33359_c0_seq1 comp57241_c1_seq1 comp54830_c2_seq11 comp55369_c3_seq1 comp49619_c0_seq5 comp54797_c0_seq1 comp56378_c4_seq10 comp47689_c0_seq1 comp36382_c1_seq1 comp55845_c0_seq2 comp50958_c4_seq1 comp40197_c1_seq1 comp42954_c0_seq2 comp57239_c2_seq6 comp54048_c1_seq1 comp56187_c0_seq5 comp56523_c2_seq1 comp47003_c0_seq1 comp48717_c0_seq1 comp56748_c2_seq7 comp53339_c0_seq2 comp50903_c0_seq4 comp54494_c2_seq2 comp30685_c0_seq2 comp56476_c1_seq2 comp49693_c2_seq1 comp52240_c1_seq2 comp57687_c1_seq5 comp50874_c0_seq1 comp55607_c0_seq4 comp47586_c1_seq1 comp51189_c2_seq1 comp52096_c0_seq6 comp42844_c0_seq2 comp52725_c0_seq2 comp54488_c0_seq1 comp47165_c0_seq1 comp56813_c2_seq1 comp46499_c0_seq2 comp51060_c1_seq1 comp51536_c2_seq4 comp56586_c2_seq24 comp32498_c0_seq1 comp43731_c0_seq1 comp55402_c0_seq2 comp53064_c1_seq1 comp57150_c0_seq5 comp46055_c0_seq1 comp46048_c0_seq3 comp49120_c0_seq3 comp36659_c0_seq1 comp43738_c0_seq2 comp47094_c0_seq3 comp55767_c0_seq2 comp50950_c0_seq4 comp52321_c0_seq2 comp53609_c0_seq5 comp48360_c1_seq1 comp51549_c0_seq3 comp54670_c2_seq1 comp54119_c1_seq4 comp57689_c2_seq13 comp45749_c2_seq1 comp53396_c0_seq1 comp55157_c0_seq2 comp47053_c2_seq1 comp57540_c0_seq4 comp32004_c0_seq2 comp50196_c0_seq1 comp51206_c1_seq1 comp56171_c0_seq3 comp55656_c5_seq5 comp45496_c0_seq2 comp54055_c0_seq1 comp55915_c1_seq2 comp50378_c2_seq1 comp27741_c0_seq1 comp57739_c8_seq5 comp28198_c0_seq1 comp58130_c0_seq12 comp45260_c1_seq1 comp51051_c0_seq4 comp57364_c2_seq1 comp42435_c0_seq1 comp51817_c1_seq2 comp52933_c1_seq1 comp43291_c0_seq1 comp57681_c1_seq7 comp43501_c0_seq1 comp55270_c0_seq13 comp55141_c0_seq10 comp58259_c1_seq14 comp52019_c0_seq3 comp56429_c0_seq9 comp52649_c0_seq2 comp53339_c1_seq1 comp31434_c0_seq1 comp45688_c0_seq3 comp56346_c0_seq7 comp54156_c0_seq1 comp58084_c0_seq18 comp48711_c1_seq2 comp54526_c0_seq1 comp57946_c4_seq6 comp46424_c0_seq1 comp50979_c0_seq1 comp51131_c0_seq2 comp57805_c1_seq2 comp49049_c0_seq1 comp53381_c1_seq1 comp57645_c1_seq5 comp56163_c0_seq3 comp42188_c0_seq3 comp58117_c1_seq3 comp54632_c0_seq24 comp51106_c1_seq4 comp57964_c0_seq3 comp32202_c0_seq1 comp57932_c0_seq6 comp27757_c0_seq1 comp56265_c0_seq8 comp55403_c1_seq1 comp55606_c4_seq12 comp47606_c0_seq1 comp54537_c0_seq1 comp58111_c3_seq39 comp57029_c0_seq2 comp47470_c0_seq2 comp53116_c0_seq2 comp56686_c1_seq1 comp58176_c0_seq2 comp50088_c0_seq1 comp50425_c0_seq1 comp57953_c3_seq4 comp58031_c2_seq21 comp53736_c0_seq2 comp53278_c0_seq1 comp48158_c0_seq1 comp44524_c0_seq1 comp49127_c0_seq1 comp209852_c0_seq1 comp57262_c0_seq2 comp56556_c0_seq2 comp49006_c0_seq3 comp52438_c0_seq7 comp54935_c0_seq2 comp57682_c1_seq2 comp29280_c0_seq1 comp43354_c0_seq1 comp51382_c0_seq1 comp53139_c1_seq6 comp54870_c0_seq2 comp28364_c0_seq1 comp56391_c3_seq7 comp51089_c0_seq2 comp53283_c0_seq6 comp50690_c0_seq1 comp29664_c0_seq1 comp58141_c1_seq7 comp46526_c0_seq1 comp59384_c0_seq1 comp49904_c2_seq5 comp57489_c0_seq27 comp57626_c12_seq1 comp46301_c0_seq1 comp50839_c0_seq3 comp51115_c0_seq1 comp53821_c1_seq3 comp58097_c2_seq20 comp54509_c2_seq7 comp56228_c4_seq5 comp38431_c0_seq1 comp47823_c0_seq1 comp54444_c1_seq1 comp31544_c0_seq1 comp54247_c0_seq1 comp52086_c0_seq4 comp51937_c1_seq3 comp52190_c0_seq2 comp54878_c1_seq1 comp55687_c1_seq1 comp56790_c0_seq1 comp28451_c0_seq1 comp57794_c0_seq2 comp57089_c0_seq2 comp56960_c0_seq3 comp29793_c0_seq2 comp56143_c0_seq1 comp50748_c0_seq2 comp57832_c1_seq6 comp57702_c3_seq1 comp44433_c0_seq1 comp32226_c0_seq1 comp57688_c0_seq6 comp53731_c3_seq2 comp57626_c5_seq1 comp51978_c0_seq1 comp55130_c3_seq1 comp47229_c0_seq1 comp40442_c0_seq1 comp58080_c2_seq9 comp55612_c0_seq1 comp48749_c0_seq1 comp54856_c0_seq2 comp54387_c1_seq11 comp57477_c0_seq1 comp57920_c0_seq5 comp44642_c0_seq1 comp50343_c0_seq2 comp46852_c0_seq1 comp55573_c2_seq3 comp51317_c1_seq1 comp57232_c1_seq13 comp54037_c1_seq2 comp36010_c0_seq4 comp64826_c0_seq1 comp54021_c1_seq5 comp46824_c0_seq1 comp48077_c3_seq1 comp45873_c0_seq1 comp56772_c0_seq3 comp51014_c0_seq1 comp55329_c0_seq3 comp50983_c1_seq1 comp43692_c1_seq1 comp48461_c0_seq1 comp54719_c0_seq6 comp56922_c2_seq2 comp54540_c1_seq3 comp58210_c0_seq7 comp52685_c0_seq1 comp55328_c0_seq1 comp56071_c0_seq2 comp52826_c0_seq1 comp55457_c0_seq1 comp52148_c0_seq2 comp58096_c3_seq13 comp57509_c3_seq6 comp57487_c5_seq25 comp55324_c0_seq2 comp57024_c0_seq5 comp50301_c0_seq2 comp56490_c0_seq8 comp55867_c3_seq4 comp54131_c0_seq1 comp57022_c5_seq2 comp57424_c1_seq1 comp49321_c1_seq3 comp56828_c0_seq3 comp53906_c2_seq1 comp55551_c1_seq1 comp55495_c4_seq3 comp49655_c1_seq5 comp56909_c1_seq1 comp56623_c0_seq1 comp56278_c0_seq3 comp56020_c2_seq2 comp57093_c0_seq1 comp57675_c2_seq1 comp58217_c0_seq47 comp54708_c1_seq4 comp52876_c0_seq3 comp56320_c0_seq1 comp55639_c0_seq7 comp51535_c0_seq1 comp41787_c2_seq1 comp51754_c1_seq1 comp36365_c0_seq1 comp57833_c0_seq5 comp50510_c1_seq1 comp57485_c0_seq2 comp32709_c1_seq1 comp29248_c0_seq1 comp57369_c3_seq1 comp55747_c1_seq13 comp57892_c1_seq2 comp57995_c6_seq13 comp47724_c1_seq1 comp49294_c1_seq3 comp49068_c0_seq2 comp56672_c1_seq5 comp39774_c0_seq1 comp53173_c0_seq4 comp51194_c0_seq1 comp38923_c0_seq1 comp57959_c0_seq5 comp50229_c0_seq1 comp57982_c3_seq1 comp42310_c2_seq1 comp55796_c0_seq3 comp57696_c0_seq2 comp56534_c2_seq1 comp54206_c0_seq6 comp57858_c2_seq1 comp53759_c0_seq4 comp54144_c0_seq6 comp51248_c1_seq1 comp55034_c1_seq3 comp57850_c3_seq2 comp56081_c0_seq3 comp29722_c0_seq1 comp57316_c1_seq1 comp27687_c0_seq1 comp52964_c0_seq2 comp58100_c1_seq8 comp51180_c1_seq3 comp53439_c2_seq1 comp52066_c0_seq2 comp53408_c3_seq3 comp54786_c0_seq5 comp55497_c0_seq4 comp56687_c1_seq1 comp57232_c1_seq4 comp56216_c1_seq4 comp54285_c1_seq2 comp53102_c1_seq6 comp52780_c3_seq2 comp50575_c0_seq1 comp51334_c3_seq10 comp50654_c0_seq1 comp53389_c2_seq1 comp56233_c6_seq5 comp55786_c0_seq3 comp49146_c0_seq3 comp57738_c0_seq13 comp57347_c0_seq3 comp30826_c0_seq1 comp40242_c0_seq1 comp57701_c2_seq10 comp53275_c1_seq3 comp46603_c0_seq1 comp52683_c0_seq2 comp55291_c0_seq2 comp57598_c0_seq3 comp57407_c1_seq17 comp57670_c0_seq2 comp55141_c0_seq2 comp31367_c0_seq1 comp50389_c0_seq5 comp58110_c0_seq7 comp56169_c0_seq3 comp57731_c0_seq6 comp57870_c0_seq1 comp48112_c1_seq1 comp57435_c1_seq2 comp57563_c1_seq5 comp54971_c0_seq2 comp57058_c0_seq12 comp55466_c3_seq2 comp57848_c0_seq22 comp57919_c0_seq9 comp57925_c1_seq28 comp131175_c0_seq1 comp54555_c0_seq4 comp53104_c0_seq2 comp46263_c0_seq3 comp56356_c0_seq5 comp57671_c7_seq24 comp48662_c0_seq1 comp57030_c2_seq7 comp56359_c0_seq1 comp49200_c1_seq2 comp49863_c0_seq1 comp55556_c0_seq9 comp57321_c0_seq9 comp53047_c0_seq1 comp46692_c0_seq3 comp54880_c0_seq5 comp55414_c5_seq18 comp56735_c0_seq15 comp45973_c0_seq1 comp57966_c0_seq8 comp56929_c4_seq1 comp48877_c0_seq1 comp57926_c6_seq3 comp57423_c0_seq6 comp55531_c0_seq2 comp56422_c1_seq7 comp52424_c0_seq1 comp56073_c1_seq1 comp49404_c1_seq5 comp48534_c0_seq1 comp55872_c0_seq2 comp40518_c1_seq1 comp56979_c5_seq7 comp39925_c0_seq1 comp56193_c2_seq1 comp58036_c1_seq12 comp49168_c0_seq7 comp52157_c0_seq9 comp53204_c0_seq1 comp53387_c1_seq6 comp43690_c0_seq1 comp49598_c0_seq6 comp58300_c0_seq12 comp54993_c0_seq5 comp52211_c0_seq1 comp56439_c1_seq6 comp47334_c0_seq2 comp55525_c1_seq9 comp55814_c0_seq1 comp48513_c0_seq1 comp54458_c8_seq13 comp46206_c0_seq1 comp56934_c0_seq2 comp51438_c1_seq1 comp53967_c0_seq2 comp48082_c0_seq3 comp56513_c0_seq5 comp52381_c0_seq3 comp56293_c0_seq1 comp56625_c1_seq15 comp52906_c1_seq1 comp58287_c0_seq1 comp29425_c0_seq1 comp56987_c9_seq1 comp54471_c0_seq6 comp56221_c0_seq12 comp52128_c0_seq1 comp32604_c0_seq1 comp53851_c0_seq8 comp49678_c1_seq1 comp46498_c4_seq1 comp53314_c3_seq1 comp57728_c2_seq2 comp30341_c0_seq1 comp54966_c0_seq1 comp57181_c1_seq2 comp57138_c8_seq1 comp29490_c0_seq1 comp51486_c0_seq3 comp51865_c0_seq9 comp58927_c0_seq1 comp49588_c0_seq4 comp29446_c0_seq1 comp56889_c0_seq3 comp42602_c0_seq2 comp47698_c2_seq1 comp54333_c0_seq4 comp57423_c0_seq2 comp55874_c1_seq1 comp44018_c0_seq1 comp58131_c2_seq3 comp55192_c13_seq1 comp57924_c0_seq3 comp56378_c4_seq19 comp57282_c1_seq32 comp57238_c0_seq3 comp42535_c0_seq1 comp46925_c0_seq2 comp54706_c1_seq4 comp54560_c0_seq1 comp57856_c15_seq24 comp56666_c0_seq7 comp30137_c0_seq1 comp54389_c0_seq2 comp39043_c2_seq1 comp57313_c0_seq6 comp44324_c1_seq1 comp53328_c1_seq2 comp29294_c0_seq1 comp46683_c0_seq1 comp48598_c1_seq1 comp50208_c0_seq1 comp52232_c1_seq1 comp263179_c0_seq1 comp57425_c0_seq1 comp58033_c0_seq6 comp57857_c1_seq14 comp58141_c1_seq8 comp53447_c0_seq1 comp44013_c0_seq1 comp48341_c0_seq4 comp45695_c0_seq1 comp53543_c1_seq3 comp53069_c0_seq3 comp57392_c0_seq5 comp49589_c0_seq3 comp48209_c0_seq1 comp56248_c1_seq3 comp52873_c1_seq1 comp58166_c1_seq3 comp55560_c3_seq5 comp41371_c0_seq1 comp56407_c0_seq1 comp51142_c1_seq1 comp58096_c7_seq1 comp54268_c7_seq1 comp52187_c0_seq6 comp57351_c3_seq11 comp47511_c1_seq2 comp32741_c2_seq1 comp57394_c1_seq7 comp53836_c0_seq3 comp57671_c4_seq1 comp53983_c1_seq8 comp52188_c0_seq2 comp57415_c0_seq5 comp45733_c0_seq2 comp52553_c2_seq4 comp42657_c0_seq1 comp49434_c1_seq1 comp57118_c0_seq5 comp54231_c0_seq1 comp57610_c5_seq2 comp51415_c1_seq2 comp57509_c3_seq1 comp43575_c0_seq1 comp51490_c0_seq3 comp48489_c0_seq2 comp57776_c3_seq18 comp57937_c1_seq2 comp54907_c1_seq3 comp31543_c0_seq1 comp35762_c0_seq1 comp54041_c1_seq4 comp51030_c0_seq2 comp56427_c1_seq2 comp168422_c0_seq1 comp51199_c2_seq6 comp52688_c1_seq2 comp57869_c0_seq14 comp57124_c3_seq2 comp55065_c0_seq1 comp58286_c1_seq31 comp57917_c0_seq4 comp52453_c2_seq1 comp30312_c0_seq1 comp57372_c1_seq4 comp55448_c1_seq5 comp52306_c0_seq4 comp52919_c0_seq5 comp54253_c0_seq2 comp47398_c1_seq1 comp58192_c0_seq12 comp53247_c3_seq1 comp46537_c1_seq1 comp56804_c1_seq26 comp58199_c0_seq7 comp55635_c2_seq3 comp57605_c2_seq15 comp57902_c1_seq15 comp46501_c0_seq3 comp49493_c0_seq1 comp43143_c0_seq2 comp49250_c0_seq1 comp50613_c0_seq1 comp55334_c0_seq3 comp57488_c3_seq4 comp51018_c0_seq1 comp35235_c2_seq1 comp57691_c0_seq4 comp52106_c1_seq1 comp50180_c2_seq1 comp55990_c0_seq2 comp54563_c0_seq1 comp46970_c0_seq4 comp45106_c4_seq1 comp54904_c0_seq4 comp30698_c0_seq1 comp271346_c0_seq1 comp42888_c0_seq1 comp49520_c0_seq1 comp31327_c0_seq1 comp57964_c0_seq8 comp47053_c1_seq1 comp52372_c1_seq2 comp49958_c1_seq2 comp56277_c0_seq2 comp57440_c0_seq2 comp54892_c0_seq2 comp56000_c2_seq10 comp55595_c0_seq2 comp50603_c1_seq1 comp56521_c4_seq3 comp43523_c3_seq1 comp49207_c0_seq2 comp46979_c0_seq2 comp56042_c0_seq13 comp56244_c1_seq8 comp53900_c0_seq3 comp52001_c1_seq2 comp57174_c1_seq1 comp56548_c0_seq1 comp58153_c0_seq8 comp55757_c0_seq1 comp48498_c2_seq1 comp58191_c2_seq1 comp55495_c1_seq1 comp56306_c2_seq1 comp52444_c1_seq4 comp54039_c0_seq2 comp55886_c0_seq1 comp52149_c0_seq3 comp57727_c0_seq8 comp50392_c0_seq1 comp57161_c2_seq1 comp42407_c0_seq1 comp56832_c5_seq2 comp55953_c0_seq8 comp57883_c0_seq3 comp57951_c0_seq25 comp51571_c3_seq1 comp57848_c0_seq8 comp58067_c0_seq1 comp50754_c0_seq5 comp57351_c3_seq19 comp52778_c0_seq5 comp54359_c2_seq1 comp55744_c7_seq2 comp56676_c1_seq1 comp56867_c1_seq2 comp51436_c0_seq4 comp57083_c1_seq10 comp56739_c0_seq1 comp47501_c0_seq1 comp45501_c2_seq1 comp57558_c4_seq2 comp55993_c1_seq2 comp56435_c1_seq4 comp57857_c1_seq18 comp49799_c0_seq4 comp55484_c0_seq24 comp54122_c0_seq1 comp58109_c1_seq7 comp54911_c1_seq7 comp52320_c0_seq9 comp48908_c1_seq1 comp48142_c0_seq1 comp36218_c0_seq1 comp45993_c1_seq1 comp52949_c0_seq4 comp55307_c0_seq1 comp119005_c0_seq1 comp56758_c5_seq9 comp53958_c2_seq1 comp54635_c0_seq1 comp47387_c1_seq1 comp56402_c0_seq3 comp52408_c0_seq3 comp57612_c0_seq16 comp45293_c0_seq3 comp53400_c1_seq7 comp57753_c3_seq4 comp41459_c0_seq1 comp57337_c0_seq3 comp51311_c0_seq6 comp55975_c0_seq5 comp55151_c0_seq1 comp58121_c0_seq4 comp45248_c0_seq2 comp45940_c0_seq1 comp54957_c1_seq4 comp52710_c1_seq2 comp39032_c0_seq1 comp29987_c0_seq2 comp56239_c2_seq2 comp51868_c0_seq3 comp55878_c1_seq4 comp57901_c0_seq13 comp51662_c1_seq1 comp51423_c1_seq3 comp41487_c0_seq3 comp54351_c1_seq1 comp57344_c0_seq1 comp53383_c1_seq1 comp51213_c0_seq2 comp53500_c0_seq3 comp49007_c0_seq1 comp48760_c1_seq1 comp57071_c0_seq2 comp51199_c2_seq2 comp55385_c1_seq2 comp46098_c2_seq2 comp53041_c1_seq1 comp54636_c0_seq5 comp27989_c0_seq1 comp57324_c1_seq1 comp50966_c0_seq4 comp43740_c0_seq1 comp58033_c0_seq3 comp46720_c0_seq1 comp53213_c0_seq2 comp52998_c0_seq5 comp55084_c0_seq3 comp51413_c2_seq2 comp55964_c0_seq4 comp50125_c1_seq1 comp49584_c0_seq2 comp28383_c0_seq1 comp55070_c1_seq13 comp55525_c1_seq12 comp57673_c1_seq4 comp264689_c0_seq1 comp58087_c6_seq1 comp56306_c4_seq2 comp57530_c3_seq4 comp50903_c0_seq7 comp51908_c0_seq2 comp46126_c1_seq2 comp53061_c1_seq1 comp52653_c1_seq1 comp33105_c0_seq1 comp57272_c2_seq2 comp45473_c0_seq4 comp49836_c0_seq2 comp31155_c0_seq1 comp32916_c0_seq4 comp57775_c0_seq6 comp55370_c1_seq9 comp54462_c0_seq3 comp58133_c3_seq8 comp57196_c0_seq1 comp56231_c1_seq1 comp53509_c0_seq3 comp50855_c0_seq1 comp55995_c10_seq67 comp49615_c0_seq1 comp48545_c0_seq1 comp57289_c0_seq4 comp54313_c0_seq3 comp50235_c0_seq6 comp55008_c0_seq4 comp56794_c0_seq1 comp50410_c0_seq4 comp48663_c1_seq2 comp58252_c3_seq18 comp57495_c8_seq7 comp54054_c1_seq3 comp50968_c0_seq1 comp53432_c0_seq1 comp53191_c0_seq4 comp54001_c0_seq1 comp56112_c1_seq4 comp47373_c0_seq1 comp55824_c2_seq2 comp50252_c0_seq1 comp55302_c0_seq7 comp55694_c0_seq1 comp51626_c7_seq3 comp56510_c0_seq3 comp57278_c4_seq1 comp56693_c3_seq1 comp53401_c1_seq12 comp32455_c0_seq3 comp51498_c0_seq3 comp41481_c0_seq1 comp43692_c0_seq1 comp42581_c0_seq2 comp57566_c0_seq1 comp32966_c0_seq3 comp46268_c2_seq1 comp54393_c0_seq5 comp54807_c0_seq1 comp57904_c3_seq11 comp57892_c1_seq6 comp56637_c0_seq1 comp50279_c0_seq5 comp55779_c0_seq4 comp58050_c2_seq1 comp48761_c0_seq1 comp51048_c0_seq1 comp56414_c0_seq4 comp54453_c3_seq1 comp54983_c0_seq4 comp57516_c1_seq10 comp58194_c0_seq8 comp45411_c0_seq1 comp43374_c1_seq1 comp56796_c3_seq1 comp43722_c0_seq1 comp52519_c0_seq3 comp56559_c0_seq2 comp43758_c0_seq1 comp56632_c2_seq4 comp56228_c0_seq6 comp51176_c0_seq1 comp41766_c0_seq1 comp54784_c0_seq1 comp48036_c0_seq3 comp55484_c0_seq3 comp57894_c0_seq4 comp58113_c5_seq5 comp52687_c0_seq1 comp55638_c0_seq3 comp57712_c2_seq2 comp58087_c4_seq8 comp55037_c0_seq2 comp52161_c0_seq1 comp49786_c2_seq1 comp57992_c0_seq4 comp49395_c0_seq1 comp43708_c0_seq2 comp57285_c0_seq9 comp54926_c2_seq7 comp57028_c0_seq1 comp47042_c0_seq1 comp30151_c0_seq1 comp55818_c3_seq4 comp57877_c0_seq6 comp58048_c4_seq11 comp54849_c0_seq3 comp52568_c8_seq1 comp51638_c0_seq2 comp50353_c1_seq1 comp50208_c3_seq1 comp57050_c0_seq1 comp56887_c1_seq1 comp56762_c3_seq2 comp58001_c3_seq16 comp57852_c0_seq2 comp56390_c3_seq6 comp58021_c3_seq20 comp47952_c0_seq1 comp55720_c1_seq1 comp55156_c1_seq4 comp53958_c4_seq1 comp57064_c2_seq1 comp50813_c0_seq3 comp29910_c0_seq1 comp55778_c1_seq2 comp39850_c0_seq1 comp57468_c0_seq13 comp58080_c2_seq2 comp39774_c0_seq3 comp57528_c11_seq1 comp51639_c0_seq2 comp57766_c0_seq18 comp45728_c0_seq1 comp53502_c0_seq7 comp55339_c2_seq1 comp30392_c0_seq1 comp56209_c1_seq7 comp56032_c11_seq10 comp57036_c0_seq2 comp49573_c0_seq1 comp28342_c0_seq2 comp59712_c0_seq1 comp56907_c3_seq4 comp54124_c1_seq2 comp55124_c0_seq1 comp53109_c0_seq2 comp50069_c0_seq1 comp56090_c1_seq1 comp54272_c1_seq1 comp55739_c0_seq5 comp57667_c1_seq6 comp55048_c0_seq5 comp52567_c0_seq2 comp55203_c1_seq3 comp56688_c2_seq1 comp47312_c0_seq1 comp48276_c0_seq1 comp57591_c2_seq8 comp54194_c0_seq5 comp52790_c0_seq3 comp36380_c0_seq1 comp58238_c1_seq4 comp54793_c1_seq2 comp31799_c0_seq2 comp39150_c0_seq1 comp53003_c0_seq1 comp50629_c0_seq1 comp29347_c0_seq1 comp50441_c0_seq1 comp57301_c0_seq1 comp51963_c1_seq1 comp52387_c0_seq5 comp48846_c1_seq1 comp36713_c0_seq2 comp57251_c0_seq2 comp55533_c0_seq14 comp51431_c0_seq1 comp46231_c0_seq1 comp56500_c0_seq1 comp46409_c0_seq1 comp52694_c1_seq1 comp58096_c8_seq2 comp57764_c7_seq44 comp54586_c3_seq2 comp57721_c0_seq6 comp47407_c0_seq1 comp45123_c0_seq1 comp54947_c2_seq4 comp57926_c5_seq1 comp51448_c1_seq3 comp47449_c0_seq2 comp57481_c0_seq5 comp48844_c0_seq8 comp31487_c0_seq1 comp57963_c2_seq3 comp49515_c0_seq4 comp31612_c0_seq1 comp29812_c0_seq1 comp57786_c1_seq8 comp29093_c0_seq1 comp57805_c1_seq12 comp50330_c0_seq3 comp56225_c0_seq1 comp56030_c0_seq2 comp50846_c0_seq1 comp52110_c1_seq4 comp55310_c0_seq3 comp49649_c0_seq1 comp57045_c1_seq3 comp45756_c0_seq2 comp46568_c0_seq1 comp57547_c2_seq3 comp46986_c1_seq1 comp57442_c1_seq5 comp55581_c3_seq2 comp54830_c2_seq4 comp41914_c1_seq2 comp30789_c0_seq1 comp55832_c0_seq1 comp58190_c2_seq11 comp53448_c1_seq3 comp51958_c2_seq5 comp56957_c4_seq5 comp54905_c0_seq1 comp55501_c1_seq3 comp53756_c0_seq1 comp52970_c0_seq1 comp56799_c0_seq6 comp58023_c1_seq9 comp57400_c1_seq2 comp52979_c2_seq2 comp30042_c0_seq1 comp51338_c0_seq2 comp56065_c1_seq1 comp53431_c2_seq11 comp53602_c1_seq2 comp57481_c0_seq3 comp52158_c0_seq1 comp55759_c0_seq2 comp56159_c0_seq1 comp55256_c4_seq11 comp54222_c0_seq1 comp57911_c2_seq8 comp55924_c0_seq7 comp55589_c3_seq4 comp49825_c0_seq1 comp52581_c0_seq2 comp55296_c0_seq2 comp57307_c0_seq8 comp48475_c1_seq1 comp47827_c1_seq1 comp48656_c0_seq3 comp33133_c0_seq1 comp56311_c0_seq2 comp57587_c2_seq10 comp56751_c2_seq14 comp47369_c0_seq1 comp53834_c1_seq1 comp50555_c1_seq2 comp43002_c1_seq1 comp50828_c0_seq2 comp55540_c1_seq3 comp56952_c6_seq8 comp54060_c0_seq3 comp51147_c1_seq1 comp55704_c1_seq2 comp56481_c2_seq13 comp29955_c0_seq1 comp56032_c11_seq1 comp53666_c0_seq1 comp57603_c18_seq10 comp54506_c2_seq3 comp46303_c0_seq1 comp54982_c1_seq4 comp57244_c0_seq6 comp54337_c0_seq17 comp53165_c1_seq1 comp57285_c0_seq4 comp56182_c0_seq4 comp29302_c0_seq1 comp55529_c0_seq9 comp54397_c2_seq3 comp55430_c1_seq4 comp57145_c3_seq22 comp56084_c3_seq3 comp56872_c2_seq6 comp51129_c0_seq1 comp57410_c1_seq4 comp55856_c1_seq4 comp50472_c0_seq1 comp57509_c3_seq12 comp50227_c1_seq2 comp45930_c1_seq1 comp53000_c1_seq1 comp56610_c3_seq11 comp53752_c1_seq4 comp51343_c2_seq2 comp52049_c0_seq3 comp57445_c0_seq16 comp55210_c0_seq2 comp50066_c0_seq2 comp48988_c1_seq2 comp55640_c5_seq24 comp55322_c0_seq5 comp56167_c1_seq4 comp57972_c0_seq15 comp51832_c0_seq2 comp32011_c0_seq1 comp54802_c0_seq1 comp58064_c0_seq15 comp57997_c4_seq19 comp49596_c3_seq1 comp56515_c4_seq8 comp52381_c0_seq4 comp56200_c0_seq25 comp47218_c0_seq1 comp58050_c1_seq5 comp9317_c0_seq1 comp35924_c1_seq1 comp54439_c0_seq1 comp55064_c0_seq2 comp55661_c0_seq1 comp49585_c0_seq3 comp57704_c3_seq7 comp44485_c0_seq1 comp57825_c2_seq17 comp58103_c3_seq2 comp57719_c1_seq1 comp55604_c2_seq5 comp60496_c0_seq1 comp338751_c0_seq1 comp55564_c0_seq5 comp57948_c0_seq24 comp49224_c0_seq1 comp50117_c3_seq3 comp53040_c0_seq2 comp58189_c0_seq6 comp56855_c0_seq1 comp54488_c1_seq5 comp41554_c0_seq3 comp44994_c1_seq1 comp46372_c2_seq1 comp49544_c0_seq5 comp55984_c2_seq4 comp51599_c3_seq1 comp30117_c0_seq2 comp48375_c0_seq2 comp50693_c4_seq1 comp56809_c5_seq2 comp55504_c0_seq4 comp31345_c0_seq2 comp55606_c1_seq2 comp55858_c0_seq5 comp57984_c3_seq4 comp29218_c0_seq2 comp54329_c0_seq1 comp57270_c1_seq2 comp48412_c0_seq3 comp54857_c0_seq2 comp49795_c0_seq1 comp56962_c3_seq10 comp57051_c6_seq8 comp43803_c0_seq1 comp58253_c1_seq21 comp49785_c0_seq2 comp51160_c0_seq1 comp52948_c0_seq1 comp56304_c7_seq1 comp28561_c0_seq1 comp57179_c0_seq4 comp49797_c0_seq5 comp43470_c0_seq1 comp51084_c0_seq2 comp46895_c0_seq4 comp58146_c4_seq12 comp47430_c0_seq1 comp48351_c0_seq2 comp56783_c2_seq5 comp57898_c3_seq7 comp53547_c1_seq3 comp51568_c0_seq2 comp51839_c0_seq2 comp52125_c1_seq2 comp55242_c1_seq2 comp52337_c1_seq3 comp58127_c0_seq4 comp58026_c0_seq4 comp29263_c1_seq1 comp55575_c2_seq1 comp52218_c0_seq3 comp55488_c0_seq1 comp48165_c1_seq1 comp54960_c2_seq5 comp56931_c0_seq16 comp53699_c1_seq4 comp58123_c0_seq13 comp57926_c6_seq2 comp44945_c2_seq1 comp58149_c1_seq4 comp57863_c4_seq10 comp54861_c0_seq1 comp43792_c0_seq1 comp57943_c0_seq1 comp53392_c0_seq7 comp57804_c2_seq5 comp51073_c0_seq1 comp53734_c0_seq2 comp58032_c0_seq16 comp56724_c1_seq2 comp52698_c0_seq5 comp35198_c0_seq1 comp46055_c0_seq2 comp56642_c0_seq15 comp53917_c1_seq1 comp53400_c1_seq4 comp51382_c0_seq2 comp26680_c0_seq1 comp44560_c0_seq1 comp57254_c6_seq1 comp45011_c0_seq5 comp47695_c0_seq1 comp57401_c0_seq3 comp43622_c0_seq1 comp57627_c0_seq1 comp30951_c0_seq1 comp29509_c1_seq1 comp46994_c0_seq1 comp50635_c1_seq1 comp49860_c0_seq6 comp55715_c0_seq2 comp54084_c4_seq11 comp56716_c6_seq4 comp58167_c0_seq4 comp52139_c0_seq5 comp54811_c1_seq1 comp57929_c1_seq11 comp45641_c1_seq1 comp48769_c0_seq1 comp57912_c0_seq8 comp57549_c0_seq1 comp55155_c0_seq2 comp56462_c0_seq7 comp52883_c0_seq1 comp55085_c1_seq1 comp52564_c0_seq7 comp41848_c1_seq1 comp47387_c0_seq1 comp55087_c0_seq5 comp39506_c0_seq1 comp50240_c0_seq1 comp52310_c0_seq3 comp29527_c0_seq1 comp52502_c0_seq1 comp57839_c0_seq5 comp43928_c0_seq1 comp53609_c0_seq2 comp55787_c3_seq2 comp44826_c1_seq3 comp55764_c2_seq1 comp56252_c0_seq2 comp41551_c0_seq2 comp51450_c1_seq2 comp55440_c0_seq1 comp58947_c0_seq1 comp58209_c0_seq3 comp49687_c0_seq4 comp53013_c1_seq5 comp52568_c2_seq3 comp55881_c1_seq9 comp54083_c0_seq2 comp56938_c4_seq12 comp57476_c2_seq1 comp52712_c3_seq2 comp32468_c0_seq1 comp49777_c0_seq1 comp53732_c3_seq2 comp56653_c1_seq26 comp55259_c0_seq2 comp51895_c0_seq1 comp48298_c2_seq1 comp57313_c0_seq1 comp55708_c0_seq1 comp51334_c3_seq4 comp48722_c0_seq1 comp55969_c0_seq4 comp32155_c0_seq2 comp53279_c1_seq3 comp46918_c0_seq2 comp55774_c3_seq3 comp185288_c0_seq1 comp47972_c0_seq1 comp55613_c0_seq1 comp58304_c1_seq5 comp57583_c0_seq12 comp58292_c0_seq2 comp50103_c3_seq1 comp50026_c0_seq2 comp57381_c0_seq3 comp55096_c0_seq1 comp49976_c0_seq1 comp53239_c0_seq2 comp53170_c2_seq1 comp59670_c0_seq1 comp50800_c0_seq1 comp53395_c0_seq4 comp50176_c1_seq1 comp48676_c0_seq1 comp54145_c0_seq1 comp42267_c0_seq3 comp56993_c0_seq6 comp56678_c0_seq2 comp29040_c0_seq1 comp47066_c0_seq2 comp52377_c0_seq1 comp51032_c0_seq1 comp57717_c0_seq3 comp58245_c1_seq37 comp51540_c2_seq3 comp53202_c4_seq9 comp55685_c1_seq3 comp53049_c1_seq1 comp57457_c2_seq20 comp58037_c4_seq11 comp48294_c1_seq2 comp42285_c2_seq1 comp50315_c0_seq1 comp57311_c0_seq2 comp55021_c0_seq1 comp43738_c0_seq1 comp50222_c1_seq2 comp30573_c0_seq1 comp41433_c0_seq2 comp52828_c0_seq1 comp49935_c2_seq1 comp32345_c0_seq1 comp53779_c2_seq17 comp56625_c1_seq14 comp58223_c6_seq27 comp46649_c0_seq1 comp57505_c1_seq11 comp50664_c0_seq1 comp57397_c9_seq1 comp57030_c2_seq8 comp57617_c7_seq11 comp53010_c0_seq5 comp44431_c0_seq1 comp56540_c0_seq6 comp51275_c1_seq1 comp57149_c6_seq20 comp55402_c0_seq1 comp56514_c0_seq1 comp54883_c0_seq1 comp50100_c0_seq2 comp50572_c3_seq2 comp32307_c0_seq1 comp53213_c1_seq1 comp51791_c0_seq2 comp54949_c0_seq1 comp58167_c0_seq10 comp47755_c0_seq1 comp54867_c1_seq3 comp57709_c0_seq1 comp57665_c2_seq1 comp57756_c3_seq3 comp51679_c4_seq3 comp55627_c0_seq7 comp46237_c0_seq1 comp53188_c2_seq1 comp57363_c1_seq9 comp57967_c2_seq10 comp52854_c0_seq2 comp57995_c6_seq18 comp55670_c6_seq1 comp51532_c0_seq2 comp50861_c0_seq2 comp54682_c0_seq5 comp51345_c0_seq1 comp40619_c0_seq1 comp57509_c3_seq9 comp57446_c0_seq3 comp56079_c4_seq5 comp54993_c0_seq2 comp52149_c0_seq7 comp56047_c3_seq4 comp203014_c0_seq1 comp48332_c0_seq2 comp55363_c1_seq4 comp49748_c0_seq1 comp55156_c2_seq4 comp56654_c3_seq2 comp42864_c0_seq1 comp56325_c0_seq1 comp56379_c2_seq1 comp54551_c0_seq1 comp58205_c6_seq1 comp44448_c0_seq1 comp57087_c1_seq10 comp30584_c0_seq1 comp28810_c0_seq1 comp55698_c0_seq5 comp47071_c0_seq5 comp50389_c0_seq6 comp52258_c3_seq3 comp58093_c5_seq4 comp54480_c0_seq1 comp57170_c1_seq6 comp42867_c0_seq1 comp48860_c2_seq1 comp51955_c0_seq2 comp49684_c0_seq13 comp57126_c4_seq1 comp30763_c0_seq1 comp52319_c0_seq1 comp42342_c0_seq2 comp56175_c0_seq4 comp46228_c0_seq1 comp38772_c0_seq2 comp51133_c0_seq3 comp58113_c2_seq5 comp41630_c0_seq1 comp51356_c0_seq1 comp53757_c0_seq2 comp51230_c1_seq11 comp51443_c0_seq1 comp32168_c0_seq1 comp48853_c0_seq2 comp96975_c0_seq1 comp53135_c2_seq3 comp34836_c0_seq1 comp49672_c0_seq1 comp54573_c0_seq1 comp47516_c1_seq1 comp45364_c0_seq2 comp55816_c0_seq2 comp35054_c0_seq1 comp56553_c0_seq6 comp56858_c3_seq4 comp51467_c0_seq2 comp51558_c0_seq1 comp52131_c0_seq1 comp53751_c0_seq1 comp55647_c0_seq1 comp54524_c0_seq2 comp47871_c1_seq2 comp55817_c1_seq9 comp50688_c0_seq1 comp52120_c0_seq1 comp51940_c1_seq1 comp52568_c10_seq2 comp53283_c0_seq7 comp51483_c2_seq2 comp47299_c3_seq1 comp42584_c4_seq4 comp55134_c0_seq1 comp54157_c7_seq26 comp55783_c0_seq2 comp29399_c0_seq1 comp61802_c0_seq1 comp56967_c1_seq1 comp58203_c5_seq5 comp51613_c0_seq3 comp51661_c1_seq1 comp30211_c0_seq1 comp57145_c3_seq10 comp47455_c2_seq1 comp57814_c0_seq1 comp34027_c0_seq1 comp57615_c1_seq5 comp54411_c0_seq1 comp53803_c1_seq3 comp49024_c0_seq1 comp51959_c0_seq2 comp43348_c0_seq1 comp56954_c2_seq3 comp58268_c2_seq16 comp55806_c1_seq1 comp55767_c0_seq7 comp50908_c0_seq2 comp57754_c2_seq3 comp47874_c0_seq1 comp58301_c1_seq7 comp54434_c4_seq1 comp51446_c0_seq1 comp58062_c4_seq8 comp50606_c0_seq1 comp34417_c1_seq1 comp58277_c0_seq24 comp55130_c1_seq2 comp56829_c1_seq3 comp52122_c0_seq1 comp58305_c0_seq15 comp56673_c1_seq7 comp52586_c0_seq1 comp49945_c2_seq3 comp51436_c0_seq1 comp56287_c3_seq1 comp50013_c0_seq1 comp56653_c1_seq15 comp53789_c1_seq2 comp49654_c0_seq8 comp46839_c4_seq1 comp49729_c0_seq3 comp51083_c0_seq1 comp41654_c0_seq3 comp46567_c0_seq2 comp54468_c1_seq10 comp28906_c0_seq1 comp55087_c1_seq5 comp54840_c4_seq2 comp55052_c0_seq4 comp56838_c0_seq2 comp52838_c0_seq2 comp57297_c1_seq1 comp58226_c0_seq6 comp53544_c1_seq3 comp51618_c0_seq4 comp57520_c0_seq1 comp43415_c0_seq4 comp32956_c0_seq5 comp48844_c0_seq10 comp48887_c1_seq2 comp37101_c0_seq1 comp54294_c0_seq1 comp47247_c0_seq3 comp55926_c0_seq4 comp52599_c0_seq2 comp45926_c0_seq1 comp49781_c2_seq1 comp55924_c0_seq6 comp52477_c0_seq1 comp53764_c4_seq6 comp42178_c0_seq1 comp57966_c0_seq6 comp55082_c1_seq6 comp48334_c1_seq3 comp46171_c0_seq1 comp56235_c3_seq4 comp50501_c2_seq1 comp55168_c15_seq1 comp56338_c2_seq1 comp48301_c0_seq1 comp56748_c3_seq1 comp56249_c0_seq2 comp49531_c0_seq3 comp56829_c1_seq14 comp55491_c2_seq1 comp53423_c1_seq14 comp29254_c0_seq1 comp55023_c0_seq2 comp48637_c0_seq1 comp43906_c0_seq1 comp46046_c0_seq1 comp57327_c2_seq1 comp53313_c0_seq1 comp51753_c2_seq1 comp54843_c1_seq1 comp55458_c0_seq4 comp37791_c0_seq2 comp56283_c1_seq7 comp53422_c0_seq3 comp51697_c0_seq1 comp56629_c0_seq4 comp45780_c1_seq2 comp49008_c1_seq3 comp55329_c0_seq1 comp58670_c0_seq1 comp56322_c10_seq1 comp50127_c0_seq1 comp54815_c2_seq2 comp56683_c1_seq36 comp49029_c0_seq1 comp32526_c0_seq1 comp42742_c1_seq1 comp53639_c1_seq3 comp58252_c3_seq21 comp57058_c0_seq11 comp51360_c1_seq4 comp56065_c5_seq4 comp58064_c0_seq1 comp48316_c1_seq1 comp50640_c1_seq3 comp52526_c0_seq3 comp56817_c6_seq1 comp47431_c2_seq1 comp56994_c0_seq1 comp56440_c0_seq3 comp57258_c0_seq1 comp56540_c0_seq1 comp58105_c1_seq17 comp51918_c0_seq4 comp55403_c1_seq4 comp52500_c3_seq3 comp53122_c0_seq2 comp57260_c0_seq7 comp56603_c3_seq7 comp46781_c0_seq1 comp56833_c0_seq2 comp53353_c0_seq6 comp57494_c3_seq1 comp56705_c2_seq1 comp50346_c0_seq1 comp58010_c0_seq21 comp56654_c2_seq7 comp36059_c0_seq1 comp48526_c1_seq1 comp55950_c0_seq1 comp57622_c1_seq3 comp40289_c0_seq1 comp57906_c1_seq7 comp57907_c1_seq1 comp45329_c1_seq1 comp51925_c1_seq3 comp55195_c3_seq5 comp55537_c0_seq14 comp53077_c1_seq2 comp57797_c1_seq2 comp49907_c0_seq1 comp51125_c0_seq2 comp57128_c0_seq2 comp50227_c0_seq1 comp42504_c0_seq1 comp56904_c0_seq19 comp51672_c0_seq3 comp57203_c0_seq4 comp56910_c0_seq1 comp57291_c5_seq1 comp56466_c0_seq7 comp53182_c0_seq1 comp54191_c0_seq2 comp45843_c0_seq1 comp48411_c0_seq1 comp55238_c10_seq2 comp56544_c0_seq3 comp54857_c0_seq3 comp41341_c0_seq2 comp48099_c0_seq2 comp57825_c2_seq9 comp54610_c2_seq1 comp52944_c0_seq3 comp53554_c5_seq1 comp129580_c0_seq1 comp57626_c17_seq1 comp55353_c1_seq14 comp57603_c10_seq7 comp50857_c0_seq1 comp46559_c1_seq1 comp48631_c0_seq3 comp53320_c7_seq3 comp45738_c0_seq2 comp133277_c0_seq1 comp56515_c3_seq4 comp46726_c1_seq1 comp56396_c0_seq5 comp57007_c5_seq1 comp49835_c0_seq2 comp57025_c0_seq1 comp55776_c0_seq1 comp54304_c6_seq1 comp55342_c0_seq1 comp57008_c0_seq5 comp56596_c0_seq1 comp52767_c0_seq3 comp47954_c0_seq1 comp56622_c5_seq4 comp54881_c0_seq1 comp56822_c0_seq2 comp31632_c0_seq1 comp53754_c0_seq2 comp28385_c0_seq1 comp42946_c0_seq2 comp55248_c1_seq7 comp57464_c0_seq9 comp48339_c0_seq3 comp55785_c0_seq6 comp57332_c0_seq2 comp52689_c0_seq1 comp31112_c0_seq1 comp53115_c0_seq1 comp37543_c0_seq2 comp54811_c1_seq5 comp57566_c15_seq2 comp46345_c0_seq4 comp47240_c0_seq1 comp191826_c0_seq1 comp55800_c0_seq1 comp54613_c2_seq3 comp48991_c0_seq2 comp57955_c1_seq1 comp53805_c0_seq3 comp55270_c0_seq7 comp43310_c1_seq2 comp58277_c0_seq32 comp44994_c2_seq1 comp56240_c0_seq2 comp30679_c0_seq1 comp53364_c0_seq2 comp53889_c0_seq1 comp48686_c0_seq1 comp56516_c2_seq4 comp55344_c1_seq6 comp57392_c0_seq4 comp56626_c4_seq3 comp51696_c2_seq2 comp50234_c1_seq1 comp54398_c0_seq5 comp56259_c3_seq2 comp57985_c3_seq23 comp55417_c2_seq4 comp52014_c0_seq1 comp55617_c0_seq8 comp55445_c1_seq2 comp49163_c0_seq2 comp57886_c0_seq1 comp55330_c0_seq1 comp56720_c1_seq1 comp56831_c0_seq2 comp47616_c0_seq2 comp57431_c9_seq4 comp46523_c0_seq1 comp56498_c0_seq2 comp53800_c0_seq1 comp54296_c2_seq6 comp57864_c0_seq1 comp56920_c0_seq4 comp58163_c1_seq1 comp56404_c0_seq6 comp55690_c0_seq2 comp52320_c0_seq4 comp52210_c1_seq7 comp52705_c0_seq1 comp37547_c0_seq1 comp57068_c0_seq1 comp54806_c0_seq8 comp54686_c0_seq2 comp51425_c0_seq3 comp54576_c0_seq3 comp55978_c1_seq2 comp48884_c0_seq6 comp57099_c0_seq7 comp54829_c0_seq1 comp58084_c0_seq16 comp48580_c1_seq1 comp52022_c3_seq3 comp56781_c0_seq2 comp56519_c0_seq2 comp43665_c0_seq1 comp58140_c4_seq1 comp54007_c1_seq2 comp58394_c0_seq1 comp54788_c0_seq4 comp54075_c1_seq3 comp55923_c0_seq7 comp56566_c1_seq1 comp45320_c0_seq1 comp51629_c3_seq1 comp57150_c1_seq1 comp48812_c0_seq1 comp57511_c0_seq1 comp52472_c0_seq1 comp46505_c0_seq1 comp31694_c0_seq2 comp32931_c0_seq1 comp46866_c0_seq3 comp50028_c3_seq2 comp58057_c2_seq10 comp55508_c0_seq1 comp56233_c6_seq4 comp57629_c3_seq7 comp44370_c1_seq2 comp45484_c0_seq3 comp54181_c1_seq2 comp57612_c0_seq23 comp54613_c5_seq4 comp55168_c12_seq1 comp49361_c0_seq1 comp57845_c1_seq1 comp58053_c2_seq11 comp51522_c0_seq1 comp55708_c0_seq4 comp57824_c3_seq5 comp36545_c0_seq1 comp53601_c0_seq2 comp57884_c3_seq9 comp40758_c1_seq1 comp58127_c0_seq5 comp45751_c0_seq1 comp52398_c0_seq6 comp57740_c6_seq13 comp55504_c3_seq2 comp51936_c2_seq4 comp55538_c1_seq2 comp58008_c2_seq15 comp57603_c3_seq3 comp49236_c1_seq4 comp48712_c1_seq1 comp52718_c0_seq1 comp32090_c0_seq2 comp53373_c0_seq1 comp57992_c0_seq1 comp48902_c2_seq1 comp58253_c1_seq19 comp57919_c0_seq11 comp56720_c1_seq12 comp51689_c0_seq5 comp42783_c0_seq2 comp114769_c0_seq1 comp55037_c0_seq12 comp55389_c1_seq3 comp56428_c4_seq2 comp58170_c1_seq39 comp50261_c0_seq2 comp57571_c0_seq3 comp55356_c0_seq5 comp54560_c3_seq13 comp52306_c0_seq1 comp57107_c0_seq3 comp56357_c4_seq18 comp46728_c6_seq1 comp52277_c0_seq1 comp53821_c2_seq2 comp55383_c2_seq4 comp51399_c1_seq1 comp54086_c0_seq9 comp30376_c0_seq1 comp47098_c5_seq2 comp56773_c0_seq2 comp50221_c2_seq1 comp46205_c0_seq3 comp55640_c1_seq1 comp51333_c0_seq5 comp51803_c0_seq2 comp53571_c0_seq12 comp57793_c1_seq1 comp53155_c0_seq1 comp31722_c0_seq1 comp50821_c2_seq2 comp48217_c1_seq2 comp57615_c1_seq9 comp55785_c0_seq2 comp55776_c1_seq1 comp49247_c1_seq2 comp45477_c2_seq1 comp55190_c0_seq6 comp53891_c0_seq2 comp57626_c14_seq3 comp54391_c5_seq1 comp54041_c1_seq2 comp54606_c0_seq2 comp51904_c2_seq2 comp53204_c0_seq5 comp52982_c0_seq6 comp52754_c0_seq1 comp50479_c0_seq1 comp43663_c0_seq1 comp54803_c0_seq2 comp56714_c3_seq9 comp45710_c0_seq1 comp35555_c0_seq1 comp57697_c1_seq1 comp56308_c0_seq3 comp56256_c0_seq7 comp57338_c1_seq5 comp57598_c0_seq7 comp55561_c1_seq1 comp55392_c0_seq2 comp29703_c0_seq1 comp55029_c0_seq2 comp54748_c0_seq1 comp53479_c0_seq1 comp50051_c0_seq1 comp54832_c0_seq6 comp57082_c0_seq6 comp49650_c2_seq1 comp56130_c0_seq4 comp54537_c1_seq1 comp51464_c0_seq2 comp50147_c1_seq2 comp55883_c2_seq1 comp47313_c0_seq1 comp52513_c1_seq3 comp56373_c0_seq2 comp50921_c0_seq5 comp57436_c0_seq2 comp51945_c0_seq1 comp54458_c8_seq7 comp43946_c0_seq4 comp50495_c0_seq1 comp54308_c0_seq4 comp48505_c0_seq1 comp45820_c0_seq1 comp59729_c0_seq1 comp58029_c1_seq2 comp49239_c1_seq1 comp57501_c1_seq1 comp32953_c0_seq1 comp58274_c2_seq19 comp57805_c1_seq9 comp56441_c1_seq5 comp57276_c6_seq6 comp51979_c0_seq4 comp55555_c0_seq1 comp53054_c1_seq1 comp52554_c0_seq4 comp43535_c0_seq2 comp42750_c0_seq1 comp53432_c0_seq2 comp32282_c0_seq1 comp47265_c0_seq2 comp132616_c0_seq1 comp51758_c0_seq11 comp44914_c0_seq1 comp52381_c0_seq9 comp58253_c1_seq24 comp58262_c0_seq1 comp57289_c2_seq1 comp55583_c1_seq3 comp37546_c0_seq1 comp54782_c1_seq8 comp57727_c0_seq7 comp55355_c0_seq2 comp56941_c0_seq7 comp78599_c0_seq1 comp140561_c0_seq1 comp57378_c2_seq1 comp39272_c0_seq2 comp49930_c1_seq2 comp54928_c0_seq1 comp55988_c0_seq2 comp57185_c0_seq1 comp58144_c1_seq4 comp46729_c0_seq1 comp50187_c0_seq1 comp30259_c0_seq1 comp55650_c1_seq1 comp54372_c0_seq4 comp55315_c0_seq2 comp50608_c2_seq1 comp62988_c0_seq1 comp57618_c0_seq20 comp54168_c1_seq2 comp53192_c0_seq1 comp43109_c1_seq1 comp56209_c1_seq4 comp51447_c0_seq2 comp56232_c2_seq1 comp44927_c0_seq1 comp51699_c1_seq1 comp51784_c0_seq6 comp50959_c0_seq1 comp51444_c0_seq2 comp48049_c0_seq1 comp45790_c0_seq1 comp48839_c0_seq1 comp43314_c0_seq1 comp58064_c0_seq5 comp41542_c0_seq1 comp55292_c0_seq2 comp57554_c1_seq6 comp51542_c0_seq3 comp57878_c0_seq6 comp42650_c1_seq2 comp55331_c1_seq3 comp43310_c0_seq1 comp30688_c0_seq1 comp49490_c0_seq1 comp57645_c1_seq3 comp57639_c12_seq6 comp56751_c0_seq4 comp51777_c1_seq1 comp51454_c1_seq4 comp58061_c0_seq19 comp51185_c1_seq1 comp53096_c0_seq3 comp51825_c0_seq3 comp57159_c1_seq8 comp58220_c0_seq2 comp47974_c1_seq2 comp31016_c0_seq2 comp28116_c0_seq1 comp52187_c0_seq2 comp57608_c1_seq4 comp57253_c0_seq2 comp52276_c0_seq4 comp50888_c0_seq1 comp41578_c0_seq1 comp124720_c0_seq1 comp57899_c2_seq1 comp57582_c2_seq5 comp49112_c0_seq1 comp57320_c1_seq13 comp56793_c0_seq1 comp52581_c0_seq1 comp57304_c0_seq5 comp54466_c6_seq3 comp57725_c0_seq9 comp52209_c0_seq2 comp47762_c0_seq1 comp54949_c1_seq1 comp55168_c7_seq1 comp49770_c8_seq2 comp42748_c1_seq1 comp58104_c2_seq6 comp57536_c1_seq11 comp51017_c0_seq1 comp55515_c0_seq3 comp54737_c0_seq2 comp57309_c0_seq9 comp54830_c2_seq17 comp47690_c1_seq1 comp58072_c1_seq16 comp55264_c7_seq2 comp36465_c1_seq1 comp56816_c0_seq12 comp43448_c0_seq4 comp45946_c0_seq1 comp49052_c0_seq1 comp58274_c2_seq12 comp55860_c0_seq4 comp41356_c0_seq1 comp46756_c0_seq2 comp42685_c0_seq4 comp57265_c0_seq1 comp56576_c1_seq2 comp55733_c0_seq1 comp51484_c0_seq1 comp48455_c0_seq1 comp54019_c1_seq5 comp36491_c0_seq1 comp55238_c9_seq1 comp56696_c2_seq12 comp52244_c0_seq4 comp52048_c0_seq3 comp50571_c0_seq1 comp52012_c5_seq5 comp52158_c1_seq2 comp57077_c0_seq3 comp52346_c0_seq3 comp54148_c0_seq4 comp54508_c0_seq1 comp50967_c0_seq2 comp42820_c0_seq1 comp58001_c3_seq24 comp55877_c1_seq7 comp58190_c2_seq3 comp46099_c0_seq2 comp42919_c0_seq2 comp56752_c1_seq2 comp42183_c0_seq1 comp52884_c1_seq3 comp31739_c0_seq1 comp68931_c0_seq1 comp59889_c0_seq1 comp58256_c1_seq10 comp58143_c0_seq18 comp57607_c0_seq2 comp44473_c0_seq1 comp51939_c0_seq6 comp44962_c1_seq1 comp50823_c0_seq1 comp42192_c0_seq1 comp53604_c0_seq1 comp41925_c0_seq1 comp53772_c2_seq6 comp57748_c3_seq5 comp50281_c0_seq2 comp52751_c0_seq1 comp56091_c1_seq1 comp54724_c2_seq3 comp54290_c0_seq2 comp53834_c0_seq1 comp57586_c0_seq1 comp49143_c0_seq1 comp50947_c1_seq3 comp53436_c0_seq1 comp56386_c0_seq9 comp50998_c1_seq2 comp44698_c0_seq1 comp53743_c0_seq5 comp55084_c0_seq8 comp57264_c0_seq2 comp53966_c4_seq2 comp52302_c0_seq3 comp57691_c0_seq3 comp56529_c5_seq1 comp52746_c0_seq1 comp55807_c1_seq1 comp49892_c4_seq3 comp55854_c0_seq4 comp58013_c0_seq2 comp29153_c0_seq1 comp52569_c0_seq3 comp57558_c1_seq1 comp56767_c0_seq4 comp56299_c2_seq14 comp54748_c2_seq4 comp57087_c1_seq4 comp32433_c0_seq1 comp54904_c0_seq1 comp58045_c0_seq11 comp44647_c1_seq1 comp49438_c1_seq1 comp52684_c1_seq2 comp47721_c0_seq1 comp52549_c0_seq1 comp52832_c0_seq2 comp52191_c0_seq1 comp55526_c5_seq1 comp52915_c0_seq1 comp29777_c0_seq1 comp56525_c0_seq1 comp32560_c0_seq1 comp43593_c0_seq1 comp42470_c0_seq1 comp58159_c0_seq20 comp55100_c1_seq1 comp55067_c1_seq1 comp54627_c1_seq3 comp57109_c2_seq3 comp56962_c3_seq6 comp49588_c0_seq1 comp55649_c0_seq3 comp54516_c2_seq1 comp57260_c0_seq15 comp216995_c0_seq1 comp48586_c0_seq1 comp55467_c4_seq3 comp54548_c1_seq2 comp53721_c1_seq3 comp52326_c0_seq1 comp53554_c5_seq2 comp50112_c0_seq2 comp51035_c0_seq1 comp53261_c0_seq1 comp55987_c1_seq2 comp28495_c0_seq1 comp42387_c0_seq1 comp50163_c0_seq2 comp51230_c1_seq3 comp51552_c0_seq1 comp57723_c0_seq1 comp53146_c0_seq7 comp52076_c0_seq2 comp57863_c4_seq6 comp57241_c2_seq6 comp53294_c1_seq6 comp58072_c1_seq24 comp29599_c0_seq1 comp50500_c1_seq1 comp56606_c0_seq1 comp47703_c0_seq1 comp47400_c1_seq1 comp57197_c1_seq6 comp57508_c0_seq1 comp53456_c0_seq1 comp56403_c0_seq5 comp57027_c0_seq1 comp53463_c1_seq3 comp55850_c0_seq2 comp54430_c0_seq3 comp53476_c0_seq2 comp57086_c1_seq4 comp48440_c0_seq3 comp42749_c0_seq1 comp50868_c0_seq5 comp55046_c0_seq3 comp30904_c0_seq1 comp51350_c2_seq4 comp54006_c0_seq2 comp58284_c6_seq7 comp56167_c1_seq1 comp43269_c1_seq4 comp56545_c0_seq2 comp45551_c0_seq1 comp50431_c0_seq4 comp52004_c3_seq1 comp56849_c4_seq6 comp50726_c0_seq1 comp57646_c0_seq1 comp48266_c0_seq1 comp57288_c2_seq1 comp53051_c1_seq2 comp46755_c0_seq2 comp51932_c2_seq1 comp57848_c0_seq13 comp57929_c1_seq4 comp49642_c2_seq1 comp55436_c5_seq5 comp51842_c1_seq2 comp58391_c0_seq1 comp56680_c0_seq4 comp58240_c1_seq7 comp55791_c0_seq3 comp46317_c0_seq2 comp53394_c0_seq1 comp51614_c0_seq1 comp55273_c1_seq1 comp56032_c11_seq4 comp58141_c1_seq11 comp46328_c0_seq1 comp54011_c1_seq4 comp57500_c2_seq13 comp47865_c0_seq1 comp56207_c1_seq10 comp47009_c0_seq1 comp52106_c1_seq4 comp53820_c0_seq4 comp54274_c0_seq5 comp54050_c3_seq3 comp31193_c0_seq1 comp57880_c2_seq9 comp57414_c0_seq1 comp47641_c0_seq1 comp49858_c0_seq1 comp56106_c4_seq4 comp54841_c7_seq4 comp38438_c0_seq1 comp53207_c0_seq4 comp53761_c2_seq2 comp55567_c0_seq1 comp57000_c5_seq3 comp48335_c1_seq1 comp54549_c1_seq6 comp57269_c0_seq1 comp57499_c6_seq9 comp55206_c0_seq2 comp57345_c2_seq9 comp48165_c1_seq2 comp40625_c0_seq1 comp52605_c0_seq3 comp56230_c14_seq16 comp55886_c9_seq6 comp50186_c0_seq2 comp52436_c0_seq2 comp49350_c0_seq1 comp100969_c0_seq1 comp57512_c0_seq1 comp53402_c0_seq2 comp56269_c0_seq1 comp56000_c2_seq11 comp38755_c0_seq1 comp47873_c1_seq1 comp54378_c3_seq2 comp48491_c0_seq1 comp51459_c0_seq1 comp56646_c1_seq1 comp49750_c1_seq4 comp57804_c2_seq9 comp56832_c0_seq2 comp46567_c0_seq7 comp52567_c1_seq1 comp56530_c1_seq4 comp46725_c1_seq1 comp56486_c1_seq4 comp58080_c2_seq5 comp56436_c0_seq3 comp54469_c0_seq1 comp57787_c2_seq1 comp57208_c3_seq20 comp56796_c0_seq1 comp51306_c0_seq2 comp56107_c0_seq3 comp53213_c2_seq1 comp50907_c0_seq1 comp48784_c2_seq1 comp55466_c1_seq18 comp46538_c0_seq2 comp56400_c0_seq2 comp51928_c0_seq1 comp57051_c12_seq2 comp57936_c5_seq8 comp57522_c1_seq4 comp57096_c1_seq2 comp51885_c0_seq1 comp43000_c0_seq5 comp52831_c0_seq3 comp42347_c1_seq1 comp55507_c1_seq2 comp58053_c2_seq4 comp50073_c3_seq3 comp57500_c0_seq1 comp53785_c2_seq1 comp48818_c0_seq2 comp54547_c1_seq15 comp57269_c0_seq5 comp50669_c0_seq6 comp57942_c0_seq3 comp52441_c0_seq1 comp54224_c3_seq1 comp56697_c0_seq1 comp53462_c0_seq2 comp45748_c1_seq1 comp57907_c12_seq5 comp58207_c0_seq3 comp56183_c0_seq1 comp53816_c1_seq2 comp56174_c1_seq7 comp56745_c2_seq2 comp54622_c0_seq1 comp57610_c6_seq3 comp56907_c3_seq7 comp55632_c0_seq1 comp52808_c4_seq4 comp4422_c0_seq1 comp29692_c0_seq2 comp57995_c1_seq1 comp56389_c1_seq3 comp27920_c0_seq1 comp56452_c0_seq3 comp48682_c1_seq1 comp54056_c0_seq2 comp45933_c0_seq1 comp57660_c0_seq5 comp44917_c0_seq1 comp58268_c2_seq12 comp57011_c0_seq1 comp56905_c0_seq3 comp48860_c5_seq1 comp51406_c1_seq2 comp57086_c1_seq9 comp50976_c2_seq3 comp46002_c0_seq1 comp57700_c0_seq1 comp57092_c1_seq11 comp46216_c4_seq2 comp49603_c2_seq1 comp54472_c0_seq2 comp46955_c1_seq2 comp53942_c1_seq1 comp57341_c5_seq2 comp56759_c2_seq1 comp47806_c1_seq1 comp51134_c0_seq1 comp46212_c0_seq1 comp57487_c5_seq3 comp57929_c2_seq1 comp57031_c1_seq16 comp48361_c0_seq1 comp31750_c0_seq2 comp39499_c0_seq1 comp57953_c3_seq21 comp54460_c1_seq1 comp54898_c0_seq1 comp43292_c0_seq2 comp56572_c9_seq4 comp49518_c0_seq1 comp57970_c1_seq3 comp38074_c0_seq1 comp47596_c0_seq2 comp57658_c6_seq2 comp58644_c0_seq1 comp30868_c0_seq1 comp55612_c0_seq2 comp57416_c1_seq5 comp57516_c1_seq11 comp33160_c0_seq1 comp49979_c1_seq7 comp45387_c0_seq1 comp58168_c5_seq1 comp57667_c1_seq5 comp38839_c1_seq1 comp52355_c0_seq1 comp49716_c0_seq1 comp184915_c0_seq1 comp57740_c2_seq25 comp57528_c12_seq5 comp54623_c1_seq5 comp31360_c0_seq1 comp51244_c1_seq1 comp49737_c0_seq8 comp54393_c0_seq8 comp54069_c0_seq2 comp49359_c0_seq1 comp53360_c0_seq1 comp56346_c0_seq10 comp30485_c0_seq1 comp57289_c0_seq3 comp53442_c1_seq2 comp45064_c0_seq1 comp56323_c0_seq5 comp51886_c0_seq2 comp49732_c0_seq2 comp31461_c0_seq1 comp56852_c2_seq4 comp33611_c0_seq1 comp55160_c1_seq2 comp48195_c0_seq3 comp29495_c1_seq1 comp58247_c0_seq30 comp55193_c2_seq1 comp48910_c1_seq2 comp55528_c0_seq1 comp28353_c0_seq1 comp49216_c1_seq1 comp55328_c2_seq1 comp56634_c3_seq1 comp52198_c0_seq1 comp37695_c0_seq1 comp47967_c0_seq1 comp41684_c0_seq1 comp56476_c0_seq1 comp57852_c0_seq1 comp58182_c0_seq3 comp47826_c0_seq1 comp62364_c0_seq1 comp55682_c0_seq2 comp43853_c2_seq1 comp54378_c4_seq11 comp46126_c1_seq1 comp57055_c7_seq2 comp53877_c2_seq3 comp56970_c0_seq1 comp144993_c0_seq1 comp54656_c1_seq4 comp54053_c0_seq1 comp57487_c5_seq24 comp48720_c1_seq1 comp52647_c1_seq1 comp52680_c0_seq3 comp56286_c3_seq5 comp52451_c0_seq1 comp57787_c2_seq4 comp52203_c0_seq3 comp53827_c3_seq2 comp47259_c0_seq2 comp45326_c1_seq1 comp41480_c0_seq2 comp54539_c0_seq2 comp49249_c0_seq1 comp48663_c0_seq1 comp58190_c2_seq10 comp56071_c0_seq1 comp56199_c0_seq18 comp48923_c0_seq2 comp55370_c1_seq5 comp54079_c2_seq1 comp29953_c0_seq1 comp46184_c0_seq1 comp49460_c1_seq3 comp58237_c0_seq3 comp56841_c0_seq3 comp32187_c0_seq4 comp43251_c0_seq1 comp53707_c0_seq14 comp22072_c0_seq1 comp58133_c3_seq10 comp56099_c0_seq16 comp55993_c5_seq3 comp53528_c0_seq1 comp56770_c0_seq2 comp57554_c1_seq11 comp55638_c0_seq4 comp55876_c0_seq1 comp43461_c0_seq1 comp55994_c0_seq6 comp54623_c1_seq3 comp54189_c0_seq3 comp53895_c0_seq1 comp57772_c2_seq3 comp50480_c0_seq1 comp57166_c0_seq4 comp58189_c0_seq1 comp54534_c1_seq3 comp57073_c5_seq16 comp112749_c0_seq1 comp56634_c5_seq3 comp48022_c0_seq4 comp54035_c1_seq1 comp51474_c0_seq3 comp49856_c0_seq1 comp57413_c1_seq1 comp54490_c2_seq1 comp58291_c0_seq15 comp51344_c0_seq4 comp58008_c2_seq4 comp42779_c0_seq1 comp56836_c1_seq1 comp56626_c4_seq8 comp49602_c0_seq2 comp48576_c3_seq1 comp57852_c0_seq3 comp50639_c0_seq1 comp57523_c0_seq1 comp55319_c0_seq1 comp46546_c1_seq1 comp57833_c0_seq11 comp52018_c0_seq1 comp58280_c0_seq2 comp51570_c0_seq1 comp44642_c0_seq4 comp53439_c2_seq2 comp55777_c1_seq1 comp57030_c1_seq1 comp51504_c0_seq1 comp58283_c4_seq4 comp56591_c0_seq2 comp46075_c1_seq1 comp47939_c2_seq2 comp54713_c0_seq5 comp54869_c0_seq1 comp50252_c0_seq4 comp29497_c0_seq1 comp55739_c0_seq4 comp55260_c1_seq6 comp54529_c0_seq1 comp51328_c0_seq1 comp57983_c0_seq9 comp50996_c1_seq2 comp55857_c5_seq2 comp51833_c0_seq2 comp48791_c0_seq1 comp214212_c0_seq1 comp56253_c0_seq1 comp57961_c1_seq2 comp56192_c4_seq6 comp56964_c3_seq2 comp44481_c0_seq1 comp54905_c0_seq2 comp57347_c0_seq2 comp55835_c0_seq1 comp57995_c1_seq7 comp56993_c0_seq3 comp55095_c1_seq1 comp41903_c1_seq1 comp57468_c0_seq14 comp51354_c0_seq2 comp42436_c1_seq4 comp51856_c1_seq1 comp32166_c0_seq2 comp47005_c0_seq1 comp58609_c0_seq1 comp48236_c1_seq1 comp52202_c0_seq4 comp49232_c1_seq1 comp56705_c0_seq15 comp33064_c0_seq2 comp47444_c1_seq1 comp29106_c0_seq1 comp56688_c2_seq7 comp52067_c0_seq1 comp51760_c0_seq3 comp56610_c4_seq1 comp48784_c0_seq1 comp56537_c0_seq3 comp50353_c4_seq1 comp54003_c0_seq2 comp56762_c0_seq4 comp57681_c0_seq1 comp51395_c0_seq1 comp41994_c0_seq1 comp56338_c2_seq5 comp57863_c7_seq3 comp55339_c2_seq4 comp57809_c3_seq2 comp56748_c2_seq4 comp58231_c1_seq5 comp46551_c0_seq2 comp58042_c1_seq13 comp53629_c0_seq8 comp53788_c0_seq2 comp58196_c3_seq1 comp55209_c0_seq3 comp51074_c0_seq4 comp56583_c2_seq3 comp54870_c0_seq3 comp36062_c0_seq1 comp57022_c7_seq4 comp54502_c2_seq2 comp57351_c3_seq4 comp48521_c1_seq2 comp32891_c0_seq1 comp51009_c0_seq1 comp48999_c0_seq1 comp54664_c0_seq1 comp58087_c4_seq7 comp59403_c0_seq1 comp57646_c0_seq2 comp46782_c0_seq1 comp49227_c0_seq3 comp53155_c0_seq3 comp57946_c2_seq1 comp48031_c0_seq1 comp58063_c2_seq4 comp57514_c2_seq1 comp48924_c0_seq4 comp57517_c4_seq1 comp52500_c3_seq1 comp54267_c0_seq4 comp56968_c0_seq6 comp54023_c0_seq6 comp57997_c3_seq1 comp51995_c1_seq1 comp53820_c0_seq1 comp55878_c1_seq5 comp55070_c1_seq1 comp46271_c1_seq1 comp42240_c0_seq3 comp31208_c0_seq1 comp56182_c0_seq3 comp56664_c1_seq3 comp58118_c1_seq3 comp52518_c0_seq5 comp57573_c1_seq6 comp58242_c0_seq21 comp57403_c2_seq1 comp46570_c0_seq1 comp53652_c0_seq1 comp32671_c0_seq1 comp56096_c4_seq10 comp53833_c0_seq3 comp54750_c0_seq4 comp56566_c1_seq5 comp52081_c0_seq1 comp56304_c7_seq2 comp56654_c3_seq1 comp58045_c0_seq19 comp50517_c1_seq3 comp58140_c5_seq1 comp50934_c0_seq8 comp50072_c1_seq1 comp51224_c0_seq1 comp44036_c2_seq1 comp51802_c1_seq1 comp52644_c0_seq1 comp54456_c0_seq1 comp58050_c1_seq2 comp53734_c0_seq7 comp56875_c0_seq7 comp52954_c0_seq3 comp48611_c0_seq3 comp58158_c2_seq17 comp57699_c5_seq1 comp56558_c0_seq5 comp56516_c2_seq12 comp58053_c2_seq6 comp62453_c0_seq1 comp56683_c1_seq24 comp58026_c0_seq7 comp57499_c6_seq19 comp56832_c0_seq3 comp56792_c1_seq2 comp42502_c0_seq1 comp54600_c0_seq1 comp56429_c0_seq18 comp32646_c0_seq2 comp53103_c0_seq1 comp53328_c1_seq1 comp56595_c0_seq8 comp40150_c0_seq1 comp45954_c0_seq1 comp48498_c2_seq2 comp46059_c0_seq1 comp54113_c0_seq2 comp57178_c0_seq6 comp49205_c1_seq2 comp55847_c0_seq3 comp54706_c1_seq5 comp54906_c4_seq2 comp56481_c2_seq10 comp44626_c0_seq1 comp55150_c6_seq1 comp57786_c1_seq2 comp55418_c0_seq1 comp57454_c9_seq1 comp57730_c2_seq8 comp53110_c2_seq3 comp57869_c0_seq1 comp55633_c2_seq4 comp56117_c1_seq6 comp55503_c2_seq3 comp54509_c2_seq13 comp57333_c0_seq3 comp58271_c1_seq1 comp57037_c0_seq1 comp54620_c0_seq1 comp46330_c0_seq1 comp57410_c1_seq7 comp57927_c3_seq11 comp55324_c0_seq3 comp57850_c3_seq5 comp58001_c3_seq3 comp57307_c0_seq3 comp52420_c1_seq3 comp51550_c0_seq1 comp51343_c2_seq1 comp50720_c0_seq2 comp56096_c4_seq7 comp57813_c5_seq2 comp50097_c3_seq5 comp48171_c0_seq2 comp56619_c0_seq1 comp45848_c0_seq1 comp56625_c0_seq4 comp53969_c0_seq1 comp53172_c0_seq3 comp46954_c0_seq1 comp56719_c0_seq5 comp29831_c0_seq2 comp55797_c0_seq1 comp50582_c1_seq1 comp57143_c0_seq2 comp52554_c0_seq1 comp56575_c0_seq8 comp147783_c0_seq1 comp58033_c0_seq5 comp53906_c2_seq2 comp28418_c0_seq1 comp55670_c5_seq7 comp52389_c0_seq4 comp46610_c0_seq1 comp55337_c1_seq4 comp55150_c3_seq21 comp57307_c0_seq7 comp55480_c0_seq1 comp54864_c4_seq1 comp57556_c0_seq3 comp50282_c0_seq1 comp57794_c0_seq1 comp57001_c3_seq4 comp51774_c0_seq1 comp36780_c1_seq1 comp54224_c3_seq5 comp55356_c0_seq10 comp50826_c0_seq1 comp54595_c0_seq1 comp57271_c0_seq2 comp48832_c0_seq2 comp56298_c4_seq3 comp48770_c1_seq1 comp57598_c0_seq4 comp56327_c1_seq3 comp49244_c0_seq2 comp30702_c0_seq2 comp51317_c1_seq2 comp32574_c0_seq1 comp56706_c1_seq3 comp50118_c2_seq1 comp50589_c0_seq1 comp31209_c0_seq1 comp51518_c1_seq3 comp55529_c0_seq8 comp49036_c0_seq1 comp57502_c5_seq1 comp50356_c1_seq4 comp41662_c0_seq1 comp52496_c0_seq1 comp56018_c2_seq3 comp55332_c0_seq1 comp52139_c0_seq8 comp39178_c0_seq1 comp57133_c0_seq8 comp49429_c0_seq2 comp30917_c0_seq1 comp46217_c0_seq1 comp57225_c6_seq8 comp35594_c0_seq1 comp52533_c6_seq1 comp43749_c0_seq1 comp49262_c1_seq2 comp55886_c11_seq1 comp53234_c0_seq1 comp57386_c0_seq1 comp49891_c1_seq1 comp49767_c0_seq2 comp49696_c0_seq1 comp55877_c1_seq1 comp55394_c0_seq1 comp51939_c0_seq3 comp57123_c0_seq6 comp42188_c0_seq2 comp57357_c2_seq4 comp57170_c1_seq2 comp56670_c0_seq9 comp57798_c1_seq2 comp52997_c0_seq1 comp55696_c0_seq3 comp48777_c0_seq1 comp47141_c0_seq1 comp58005_c0_seq21 comp53958_c0_seq1 comp48395_c1_seq1 comp29531_c0_seq1 comp57665_c1_seq19 comp57495_c8_seq6 comp56074_c0_seq8 comp49974_c0_seq1 comp57054_c1_seq13 comp55547_c0_seq3 comp58037_c4_seq2 comp56784_c2_seq5 comp46534_c1_seq1 comp50493_c0_seq1 comp42829_c0_seq1 comp28877_c0_seq1 comp48379_c0_seq1 comp56696_c2_seq11 comp44454_c0_seq1 comp57388_c1_seq1 comp55836_c0_seq6 comp49973_c0_seq5 comp55230_c1_seq3 comp116786_c0_seq1 comp52783_c1_seq3 comp57799_c1_seq7 comp28133_c0_seq2 comp55671_c1_seq1 comp57897_c0_seq8 comp56733_c0_seq5 comp57947_c0_seq9 comp57577_c14_seq1 comp57312_c3_seq5 comp57088_c2_seq4 comp50314_c0_seq4 comp36615_c0_seq1 comp55328_c1_seq2 comp57631_c0_seq3 comp56091_c0_seq4 comp56570_c0_seq5 comp50292_c0_seq1 comp52575_c0_seq2 comp50343_c1_seq1 comp43219_c0_seq1 comp47612_c0_seq2 comp57579_c2_seq3 comp45968_c1_seq1 comp55065_c0_seq2 comp56656_c1_seq1 comp56831_c1_seq1 comp57013_c0_seq1 comp27826_c0_seq1 comp58137_c2_seq17 comp31273_c0_seq1 comp55877_c1_seq9 comp55607_c0_seq3 comp50935_c0_seq2 comp58203_c5_seq1 comp53177_c0_seq6 comp47064_c3_seq2 comp57899_c2_seq14 comp58144_c1_seq2 comp53639_c1_seq1 comp51106_c1_seq3 comp52474_c1_seq4 comp51758_c0_seq3 comp38463_c0_seq1 comp54748_c2_seq2 comp55491_c3_seq6 comp46912_c0_seq1 comp31327_c0_seq2 comp55744_c8_seq2 comp28411_c0_seq1 comp57834_c2_seq15 comp50944_c1_seq1 comp38389_c0_seq1 comp40709_c0_seq1 comp47162_c2_seq1 comp57260_c0_seq11 comp55314_c0_seq7 comp53480_c1_seq4 comp58042_c1_seq9 comp56564_c0_seq1 comp53033_c0_seq6 comp41823_c0_seq1 comp50114_c1_seq4 comp53154_c0_seq3 comp58160_c1_seq7 comp57976_c0_seq1 comp54658_c0_seq2 comp31563_c0_seq1 comp56125_c0_seq2 comp55389_c1_seq5 comp49007_c0_seq2 comp58263_c1_seq11 comp47455_c1_seq1 comp47033_c2_seq2 comp57134_c1_seq5 comp51044_c0_seq2 comp50241_c0_seq1 comp57904_c3_seq12 comp58223_c4_seq1 comp56304_c10_seq1 comp55656_c5_seq4 comp56641_c3_seq7 comp46230_c0_seq1 comp58246_c1_seq8 comp51299_c1_seq5 comp56659_c12_seq1 comp51370_c1_seq1 comp4475_c1_seq1 comp56827_c0_seq7 comp57618_c0_seq8 comp54069_c0_seq4 comp52085_c0_seq1 comp57273_c1_seq2 comp51334_c3_seq2 comp58136_c0_seq7 comp57198_c4_seq7 comp57013_c1_seq4 comp57741_c0_seq9 comp45802_c0_seq1 comp54339_c2_seq4 comp57729_c2_seq11 comp46640_c0_seq1 comp57315_c0_seq1 comp55443_c0_seq2 comp52316_c0_seq1 comp50279_c0_seq3 comp55087_c1_seq1 comp54684_c0_seq1 comp49644_c0_seq1 comp50433_c0_seq2 comp55414_c5_seq5 comp52130_c1_seq4 comp57272_c2_seq7 comp48006_c2_seq1 comp53835_c0_seq1 comp56391_c3_seq4 comp54334_c1_seq4 comp53977_c0_seq1 comp52582_c6_seq3 comp56932_c0_seq1 comp39510_c0_seq1 comp57676_c0_seq3 comp56716_c1_seq1 comp57440_c0_seq3 comp35830_c1_seq1 comp47786_c0_seq2 comp28327_c0_seq1 comp45787_c0_seq1 comp47620_c1_seq3 comp50484_c0_seq2 comp55640_c5_seq10 comp58223_c6_seq3 comp58124_c0_seq3 comp55278_c2_seq7 comp56522_c0_seq3 comp55053_c0_seq1 comp58040_c3_seq7 comp49855_c0_seq1 comp55354_c0_seq1 comp132306_c0_seq1 comp52143_c0_seq4 comp35138_c0_seq1 comp56556_c0_seq3 comp55652_c1_seq2 comp58287_c0_seq11 comp48102_c1_seq1 comp52822_c0_seq2 comp31554_c0_seq1 comp51097_c1_seq1 comp57430_c1_seq27 comp58133_c3_seq13 comp49325_c1_seq2 comp54411_c0_seq4 comp32951_c0_seq2 comp58344_c0_seq1 comp57409_c1_seq4 comp28027_c0_seq1 comp52262_c0_seq1 comp41721_c0_seq1 comp55460_c1_seq3 comp56751_c2_seq4 comp47397_c0_seq1 comp55142_c1_seq1 comp56489_c0_seq2 comp54499_c1_seq2 comp58286_c1_seq10 comp44671_c1_seq1 comp51051_c0_seq3 comp36044_c0_seq1 comp54917_c0_seq2 comp47974_c1_seq1 comp51801_c1_seq1 comp49247_c0_seq1 comp59489_c0_seq1 comp57443_c11_seq13 comp50013_c0_seq2 comp51092_c0_seq5 comp59347_c0_seq1 comp31396_c0_seq1 comp56162_c0_seq2 comp57831_c1_seq3 comp55190_c0_seq5 comp54697_c0_seq1 comp30754_c0_seq1 comp55238_c6_seq11 comp56054_c4_seq2 comp49023_c3_seq1 comp46173_c2_seq1 comp56684_c0_seq8 comp48051_c0_seq1 comp50350_c2_seq1 comp48167_c0_seq2 comp56644_c0_seq1 comp53908_c0_seq2 comp51775_c0_seq1 comp53383_c1_seq5 comp42873_c0_seq1 comp57556_c0_seq7 comp49536_c0_seq1 comp55882_c5_seq11 comp58048_c4_seq5 comp56974_c0_seq3 comp50394_c1_seq1 comp46591_c0_seq1 comp57608_c1_seq11 comp46834_c1_seq1 comp50830_c0_seq1 comp51498_c0_seq1 comp45888_c0_seq1 comp49228_c0_seq2 comp40830_c0_seq1 comp43019_c1_seq1 comp40416_c0_seq1 comp55490_c0_seq1 comp55923_c0_seq5 comp58247_c0_seq12 comp55076_c1_seq1 comp53556_c1_seq1 comp54759_c0_seq1 comp48646_c1_seq2 comp58173_c0_seq9 comp55111_c0_seq4 comp55667_c1_seq4 comp58088_c12_seq1 comp43121_c0_seq1 comp47021_c1_seq1 comp54171_c1_seq6 comp47767_c0_seq1 comp52383_c0_seq1 comp56518_c4_seq1 comp33724_c0_seq1 comp42626_c0_seq1 comp55125_c0_seq1 comp53978_c0_seq1 comp50381_c0_seq1 comp54228_c0_seq2 comp52674_c0_seq2 comp48975_c0_seq3 comp55833_c0_seq5 comp52507_c1_seq1 comp57338_c2_seq1 comp54018_c0_seq3 comp44992_c0_seq2 comp54864_c6_seq2 comp57552_c2_seq11 comp53580_c1_seq5 comp57487_c5_seq11 comp54617_c0_seq1 comp55122_c1_seq2 comp55617_c0_seq7 comp53701_c0_seq1 comp55682_c0_seq6 comp56625_c1_seq16 comp50266_c0_seq1 comp54359_c2_seq3 comp55653_c0_seq4 comp54610_c0_seq3 comp55748_c1_seq1 comp56011_c1_seq1 comp54403_c0_seq4 comp56622_c3_seq1 comp49666_c3_seq1 comp45213_c0_seq2 comp57232_c1_seq3 comp55547_c3_seq1 comp45840_c0_seq1 comp31066_c0_seq1 comp45482_c1_seq2 comp55289_c0_seq4 comp54572_c0_seq1 comp56653_c1_seq17 comp29559_c0_seq1 comp56065_c2_seq1 comp55010_c0_seq3 comp55553_c0_seq1 comp52567_c1_seq5 comp56705_c1_seq3 comp56792_c0_seq2 comp46352_c0_seq1 comp55655_c3_seq2 comp40359_c0_seq1 comp51862_c0_seq2 comp55895_c0_seq10 comp57805_c0_seq2 comp53966_c1_seq1 comp58080_c2_seq8 comp44537_c1_seq1 comp57819_c0_seq4 comp55082_c6_seq1 comp48057_c1_seq2 comp51737_c0_seq2 comp35913_c0_seq1 comp58185_c2_seq3 comp47169_c2_seq1 comp54830_c2_seq6 comp45749_c1_seq1 comp52121_c0_seq1 comp42479_c1_seq1 comp51350_c2_seq3 comp54321_c0_seq4 comp30519_c1_seq1 comp57936_c5_seq4 comp55094_c0_seq1 comp51958_c2_seq7 comp48274_c0_seq2 comp55980_c3_seq1 comp38713_c0_seq2 comp41331_c1_seq1 comp56920_c0_seq9 comp43476_c0_seq1 comp54143_c0_seq2 comp48413_c1_seq3 comp29775_c0_seq1 comp52714_c0_seq2 comp50459_c0_seq1 comp49300_c0_seq2 comp58066_c1_seq4 comp55154_c1_seq2 comp49943_c0_seq1 comp50344_c1_seq1 comp50766_c0_seq1 comp54686_c0_seq4 comp53650_c1_seq2 comp56145_c8_seq2 comp58242_c0_seq15 comp57883_c0_seq13 comp50335_c0_seq1 comp57907_c12_seq8 comp52237_c1_seq2 comp58296_c0_seq5 comp41839_c0_seq2 comp56065_c0_seq1 comp47805_c0_seq4 comp29581_c0_seq1 comp49596_c2_seq4 comp52706_c0_seq1 comp51203_c0_seq1 comp56717_c0_seq4 comp50022_c1_seq3 comp56716_c2_seq7 comp52465_c2_seq1 comp52876_c0_seq2 comp57825_c2_seq3 comp54428_c0_seq7 comp51218_c0_seq1 comp55645_c0_seq7 comp50824_c0_seq4 comp52114_c0_seq3 comp51662_c1_seq2 comp47651_c0_seq2 comp33262_c0_seq2 comp52150_c1_seq5 comp50098_c0_seq2 comp55541_c1_seq4 comp42705_c1_seq1 comp57274_c1_seq2 comp57767_c2_seq1 comp56962_c3_seq11 comp57958_c0_seq1 comp56991_c1_seq3 comp55700_c0_seq3 comp55280_c3_seq2 comp57505_c2_seq1 comp46165_c0_seq1 comp56127_c0_seq1 comp57967_c2_seq1 comp57721_c0_seq4 comp49716_c3_seq1 comp45849_c0_seq3 comp58081_c1_seq7 comp53119_c0_seq1 comp57847_c0_seq3 comp55993_c1_seq1 comp56005_c0_seq3 comp51572_c0_seq1 comp56243_c2_seq16 comp58153_c0_seq6 comp55744_c8_seq17 comp58250_c1_seq16 comp32757_c0_seq1 comp54760_c0_seq14 comp54226_c0_seq1 comp28286_c0_seq1 comp46686_c0_seq1 comp52678_c0_seq3 comp58063_c0_seq20 comp47639_c0_seq4 comp49418_c0_seq2 comp52226_c1_seq1 comp55584_c0_seq1 comp56889_c0_seq2 comp57291_c3_seq2 comp56096_c4_seq17 comp58125_c1_seq8 comp55517_c0_seq2 comp55056_c0_seq4 comp47814_c0_seq3 comp51533_c0_seq1 comp51465_c0_seq1 comp56737_c4_seq3 comp51512_c2_seq2 comp57777_c0_seq3 comp32817_c0_seq2 comp57254_c9_seq1 comp57221_c0_seq10 comp49655_c1_seq6 comp56832_c5_seq1 comp57953_c3_seq24 comp53661_c0_seq11 comp45891_c0_seq2 comp51228_c2_seq2 comp47721_c0_seq2 comp53738_c0_seq1 comp54849_c0_seq2 comp57963_c2_seq4 comp46246_c2_seq1 comp58000_c0_seq5 comp50075_c0_seq1 comp36539_c0_seq1 comp58020_c0_seq5 comp50886_c1_seq1 comp51858_c0_seq1 comp56008_c0_seq2 comp57669_c0_seq3 comp57605_c2_seq1 comp43888_c0_seq1 comp53108_c1_seq1 comp252904_c0_seq1 comp29266_c0_seq1 comp50066_c0_seq3 comp56441_c1_seq9 comp51696_c2_seq3 comp46652_c0_seq1 comp52504_c0_seq2 comp56584_c3_seq9 comp41516_c0_seq1 comp28623_c0_seq1 comp46889_c0_seq2 comp57123_c0_seq2 comp53506_c0_seq1 comp50389_c0_seq8 comp39546_c0_seq1 comp52320_c0_seq6 comp50714_c0_seq1 comp53064_c3_seq1 comp54525_c0_seq2 comp53264_c0_seq3 comp45963_c0_seq3 comp29502_c0_seq1 comp56117_c1_seq13 comp56856_c2_seq5 comp55717_c1_seq2 comp48832_c0_seq1 comp57237_c3_seq1 comp56597_c0_seq4 comp56084_c3_seq2 comp54448_c0_seq4 comp55430_c1_seq1 comp43789_c0_seq2 comp57601_c0_seq3 comp56517_c0_seq3 comp55742_c0_seq7 comp46161_c1_seq1 comp56580_c0_seq5 comp56047_c0_seq8 comp43778_c0_seq1 comp57799_c1_seq3 comp55064_c0_seq1 comp50007_c0_seq1 comp56217_c0_seq3 comp54339_c0_seq6 comp58253_c1_seq13 comp57684_c0_seq6 comp50884_c0_seq4 comp51252_c1_seq1 comp51554_c2_seq2 comp57636_c0_seq10 comp42643_c0_seq1 comp50286_c2_seq4 comp57680_c0_seq10 comp46206_c0_seq2 comp65937_c0_seq1 comp29574_c0_seq1 comp57871_c0_seq2 comp55936_c0_seq3 comp46565_c1_seq1 comp57984_c3_seq5 comp35386_c0_seq1 comp55950_c0_seq6 comp50905_c0_seq2 comp49977_c0_seq1 comp54605_c2_seq7 comp47140_c3_seq1 comp45582_c4_seq1 comp57007_c3_seq3 comp48646_c2_seq1 comp38315_c0_seq1 comp55491_c3_seq2 comp56734_c0_seq1 comp49168_c0_seq4 comp49055_c2_seq1 comp55577_c1_seq1 comp55556_c0_seq5 comp56249_c0_seq4 comp52740_c0_seq1 comp54657_c0_seq2 comp32966_c0_seq2 comp56999_c2_seq2 comp57296_c0_seq9 comp53468_c0_seq1 comp55463_c0_seq3 comp57222_c3_seq7 comp56586_c2_seq17 comp57664_c5_seq3 comp53555_c1_seq3 comp57909_c0_seq10 comp57721_c5_seq1 comp54349_c0_seq2 comp54830_c6_seq1 comp56367_c2_seq2 comp50847_c0_seq1 comp56881_c0_seq9 comp44830_c1_seq1 comp42212_c0_seq1 comp48763_c1_seq1 comp29681_c0_seq1 comp35424_c0_seq1 comp49110_c2_seq2 comp57026_c0_seq10 comp51027_c0_seq1 comp53223_c0_seq1 comp63871_c0_seq1 comp57087_c1_seq8 comp57862_c4_seq5 comp50403_c0_seq2 comp46369_c0_seq1 comp57306_c4_seq2 comp46501_c0_seq2 comp49792_c0_seq1 comp56880_c0_seq1 comp56266_c0_seq6 comp54818_c0_seq1 comp48318_c0_seq1 comp54604_c0_seq5 comp32793_c0_seq1 comp50016_c2_seq2 comp56310_c0_seq1 comp30244_c0_seq1 comp56065_c5_seq2 comp52810_c0_seq1 comp43673_c0_seq1 comp49683_c0_seq5 comp56384_c0_seq9 comp55976_c1_seq8 comp42294_c0_seq2 comp55415_c1_seq3 comp29259_c0_seq1 comp60222_c0_seq1 comp46422_c0_seq1 comp54830_c2_seq14 comp53538_c4_seq1 comp52382_c3_seq2 comp57261_c0_seq4 comp52619_c0_seq3 comp57608_c1_seq8 comp57518_c6_seq1 comp54628_c0_seq1 comp52654_c0_seq3 comp57165_c1_seq43 comp52553_c2_seq5 comp38900_c1_seq1 comp57144_c1_seq2 comp57073_c5_seq2 comp52757_c0_seq1 comp52381_c0_seq5 comp56146_c0_seq1 comp55195_c3_seq3 comp47172_c1_seq1 comp52859_c0_seq3 comp54183_c1_seq2 comp55041_c0_seq3 comp51024_c1_seq3 comp57697_c0_seq1 comp58153_c0_seq9 comp30833_c0_seq1 comp31627_c1_seq1 comp44269_c1_seq3 comp42086_c0_seq3 comp51221_c1_seq1 comp31793_c0_seq2 comp57313_c0_seq7 comp51486_c0_seq2 comp56264_c0_seq5 comp50564_c0_seq2 comp52025_c0_seq1 comp57087_c1_seq14 comp56692_c3_seq4 comp52433_c0_seq4 comp48789_c0_seq3 comp55243_c0_seq5 comp45989_c2_seq2 comp55886_c0_seq2 comp53881_c1_seq3 comp51819_c0_seq1 comp56684_c0_seq18 comp54802_c0_seq2 comp32892_c0_seq1 comp54910_c0_seq1 comp50754_c0_seq4 comp50818_c0_seq1 comp45221_c0_seq1 comp52863_c0_seq3 comp56047_c3_seq3 comp56709_c1_seq7 comp48827_c0_seq3 comp54655_c1_seq3 comp55765_c1_seq3 comp49682_c0_seq2 comp54205_c1_seq1 comp52231_c1_seq2 comp56054_c0_seq1 comp51375_c0_seq1 comp46187_c0_seq2 comp55103_c0_seq1 comp56447_c7_seq1 comp54175_c3_seq4 comp53074_c0_seq1 comp55727_c0_seq2 comp46423_c0_seq1 comp46151_c1_seq2 comp46544_c0_seq3 comp49459_c0_seq1 comp32956_c0_seq3 comp57995_c6_seq17 comp58086_c1_seq29 comp48816_c0_seq2 comp53448_c3_seq2 comp58062_c4_seq3 comp48837_c0_seq1 comp52662_c0_seq9 comp54653_c0_seq1 comp55744_c9_seq5 comp46400_c1_seq1 comp30219_c0_seq1 comp56583_c1_seq1 comp52460_c2_seq2 comp57445_c0_seq15 comp54939_c0_seq5 comp44273_c0_seq1 comp54075_c4_seq1 comp47740_c0_seq1 comp28320_c0_seq1 comp53238_c0_seq1 comp55164_c0_seq4 comp29449_c1_seq1 comp49322_c0_seq1 comp30942_c0_seq1 comp49361_c2_seq1 comp51413_c2_seq1 comp58180_c3_seq9 comp49203_c0_seq1 comp57635_c1_seq1 comp51484_c0_seq3 comp50115_c0_seq1 comp53272_c0_seq3 comp46901_c0_seq1 comp53300_c0_seq1 comp52885_c0_seq2 comp54712_c0_seq3 comp29891_c0_seq1 comp50671_c0_seq1 comp56440_c0_seq5 comp57490_c1_seq3 comp56255_c8_seq4 comp56409_c1_seq1 comp38260_c0_seq1 comp55097_c4_seq7 comp56659_c10_seq3 comp49580_c0_seq3 comp48329_c1_seq1 comp54817_c3_seq3 comp53166_c0_seq2 comp51523_c2_seq1 comp56696_c2_seq2 comp57743_c4_seq1 comp51302_c2_seq1 comp48158_c0_seq2 comp45725_c0_seq1 comp2893_c0_seq1 comp50242_c1_seq1 comp53142_c0_seq3 comp54890_c1_seq2 comp57647_c3_seq17 comp46858_c0_seq1 comp48239_c1_seq3 comp53398_c4_seq6 comp43912_c0_seq1 comp54509_c2_seq2 comp57850_c0_seq1 comp56867_c4_seq1 comp53821_c1_seq2 comp58196_c0_seq6 comp55119_c1_seq1 comp54871_c2_seq13 comp57691_c0_seq5 comp52881_c0_seq1 comp52004_c1_seq1 comp49681_c1_seq2 comp46186_c0_seq1 comp42241_c1_seq1 comp54750_c5_seq1 comp50087_c0_seq3 comp47841_c1_seq2 comp58054_c0_seq18 comp55752_c0_seq1 comp46587_c0_seq3 comp55184_c5_seq1 comp49913_c1_seq2 comp42953_c0_seq2 comp51804_c1_seq2 comp54908_c1_seq2 comp56634_c5_seq8 comp49329_c0_seq2 comp55811_c0_seq1 comp56007_c0_seq1 comp53802_c0_seq3 comp47877_c0_seq1 comp57848_c0_seq6 comp35028_c0_seq1 comp34231_c0_seq1 comp54349_c3_seq2 comp49504_c0_seq4 comp54488_c1_seq2 comp57198_c2_seq1 comp57710_c1_seq5 comp43887_c0_seq6 comp46450_c0_seq1 comp58205_c9_seq1 comp56738_c0_seq1 comp56238_c1_seq7 comp53400_c1_seq6 comp55502_c0_seq5 comp57358_c0_seq2 comp58225_c0_seq3 comp57229_c2_seq2 comp29523_c0_seq1 comp54063_c1_seq2 comp52842_c0_seq6 comp51877_c0_seq1 comp57716_c2_seq13 comp55271_c0_seq2 comp31083_c0_seq1 comp56408_c2_seq1 comp43370_c0_seq1 comp46251_c1_seq6 comp56382_c1_seq5 comp57254_c9_seq8 comp57022_c12_seq2 comp52796_c1_seq1 comp48132_c0_seq2 comp44334_c0_seq1 comp57652_c1_seq2 comp57891_c0_seq3 comp53730_c1_seq5 comp51514_c0_seq1 comp43175_c0_seq1 comp54612_c3_seq6 comp56466_c0_seq3 comp53704_c3_seq3 comp48341_c0_seq2 comp48727_c0_seq1 comp54725_c0_seq1 comp53232_c0_seq2 comp53341_c1_seq1 comp57570_c0_seq3 comp58370_c0_seq1 comp15804_c0_seq1 comp53784_c0_seq3 comp42512_c1_seq1 comp53350_c0_seq2 comp57151_c0_seq2 comp55225_c0_seq1 comp30293_c0_seq1 comp54884_c1_seq2 comp53609_c0_seq4 comp53637_c1_seq3 comp52789_c0_seq2 comp35296_c1_seq1 comp57895_c0_seq4 comp50102_c0_seq1 comp42460_c0_seq1 comp44472_c0_seq1 comp45190_c1_seq1 comp51536_c2_seq5 comp55131_c0_seq5 comp53943_c0_seq4 comp51223_c0_seq1 comp53979_c0_seq2 comp45683_c0_seq2 comp58259_c1_seq13 comp52170_c0_seq1 comp49853_c0_seq1 comp29362_c0_seq1 comp51459_c1_seq4 comp55931_c0_seq2 comp48955_c5_seq1 comp57627_c0_seq3 comp50279_c0_seq6 comp53124_c0_seq1 comp53984_c1_seq5 comp56113_c0_seq7 comp52470_c0_seq2 comp58910_c0_seq1 comp60292_c0_seq1 comp53817_c0_seq1 comp57992_c0_seq3 comp48481_c0_seq1 comp57956_c0_seq10 comp58288_c0_seq15 comp52125_c0_seq7 comp56322_c11_seq1 comp45052_c0_seq5 comp55644_c0_seq2 comp51498_c0_seq4 comp53149_c0_seq1 comp58209_c0_seq6 comp53596_c1_seq1 comp54841_c3_seq3 comp54731_c1_seq6 comp57052_c1_seq6 comp53955_c2_seq9 comp50291_c0_seq2 comp52730_c0_seq1 comp54162_c0_seq2 comp51629_c0_seq1 comp41384_c0_seq1 comp54988_c0_seq4 comp56372_c1_seq9 comp55725_c0_seq1 comp56569_c0_seq6 comp55181_c0_seq6 comp54113_c0_seq1 comp51191_c0_seq1 comp54114_c0_seq5 comp41363_c0_seq1 comp49999_c0_seq3 comp57072_c0_seq4 comp49467_c3_seq2 comp54240_c0_seq2 comp48314_c0_seq1 comp57237_c7_seq6 comp57532_c0_seq1 comp58096_c8_seq3 comp56900_c0_seq1 comp54050_c3_seq2 comp57775_c0_seq4 comp47190_c0_seq1 comp43263_c1_seq1 comp57073_c7_seq1 comp46932_c1_seq1 comp45944_c0_seq2 comp49895_c0_seq2 comp47343_c1_seq1 comp53301_c2_seq1 comp55001_c2_seq5 comp56251_c1_seq2 comp49811_c0_seq1 comp46717_c0_seq1 comp43369_c1_seq1 comp58135_c1_seq14 comp42413_c0_seq1 comp58152_c0_seq1 comp42831_c2_seq1 comp48247_c0_seq5 comp33200_c0_seq1 comp44372_c0_seq1 comp56886_c0_seq2 comp51207_c0_seq3 comp50704_c1_seq3 comp32697_c0_seq2 comp53624_c0_seq1 comp55023_c0_seq3 comp43465_c1_seq1 comp58268_c2_seq15 comp32481_c0_seq1 comp28767_c0_seq1 comp51684_c0_seq1 comp53348_c0_seq1 comp54668_c0_seq2 comp42159_c0_seq1 comp50677_c0_seq3 comp55516_c1_seq13 comp48099_c0_seq1 comp85029_c0_seq1 comp56155_c10_seq2 comp51797_c1_seq1 comp56629_c0_seq1 comp55238_c10_seq5 comp47835_c0_seq1 comp49865_c2_seq1 comp57456_c3_seq1 comp53728_c2_seq1 comp48071_c0_seq4 comp51461_c0_seq1 comp57077_c0_seq6 comp51606_c0_seq3 comp56727_c0_seq6 comp30578_c0_seq1 comp56396_c0_seq4 comp54823_c0_seq2 comp58102_c7_seq1 comp56787_c0_seq3 comp52111_c2_seq2 comp53855_c0_seq1 comp51649_c0_seq2 comp56993_c1_seq1 comp54740_c0_seq1 comp52125_c0_seq3 comp165806_c0_seq1 comp41220_c0_seq1 comp56335_c0_seq1 comp52246_c0_seq1 comp53185_c0_seq4 comp44956_c0_seq1 comp58159_c0_seq21 comp53022_c1_seq2 comp53147_c2_seq3 comp58013_c2_seq2 comp50703_c3_seq1 comp51156_c0_seq1 comp41662_c1_seq1 comp50165_c0_seq1 comp49330_c1_seq2 comp27856_c0_seq2 comp54427_c1_seq3 comp57031_c1_seq4 comp56129_c3_seq1 comp30632_c0_seq1 comp50319_c1_seq2 comp57121_c0_seq1 comp48214_c0_seq2 comp49827_c1_seq4 comp56752_c0_seq1 comp55053_c0_seq2 comp50567_c1_seq1 comp49944_c5_seq1 comp57583_c0_seq15 comp48502_c0_seq2 comp57248_c0_seq6 comp42877_c0_seq3 comp55740_c1_seq2 comp48686_c0_seq2 comp49794_c0_seq1 comp45590_c0_seq2 comp53527_c2_seq7 comp54760_c0_seq7 comp46156_c0_seq1 comp55450_c0_seq3 comp56920_c0_seq5 comp50972_c0_seq1 comp56745_c0_seq2 comp54304_c5_seq1 comp53642_c0_seq6 comp57056_c1_seq9 comp58009_c2_seq11 comp51026_c0_seq4 comp28964_c0_seq1 comp56734_c1_seq2 comp48720_c1_seq2 comp33195_c0_seq1 comp53346_c0_seq3 comp58104_c2_seq11 comp53650_c3_seq2 comp50431_c0_seq1 comp57654_c3_seq8 comp56654_c3_seq11 comp49275_c0_seq1 comp51872_c1_seq3 comp57401_c2_seq1 comp48781_c0_seq1 comp55016_c0_seq1 comp48559_c0_seq1 comp55503_c6_seq8 comp57272_c0_seq4 comp58269_c2_seq2 comp54576_c0_seq4 comp53638_c0_seq1 comp55110_c1_seq1 comp55775_c0_seq1 comp55656_c0_seq11 comp54950_c2_seq4 comp57058_c0_seq1 comp58152_c1_seq7 comp56579_c3_seq2 comp56213_c0_seq3 comp52232_c1_seq3 comp50206_c0_seq1 comp52221_c0_seq4 comp56894_c0_seq13 comp55564_c0_seq7 comp56786_c1_seq1 comp57013_c1_seq1 comp50280_c0_seq1 comp55344_c1_seq4 comp32838_c0_seq1 comp50137_c0_seq1 comp57702_c3_seq4 comp56777_c2_seq3 comp53732_c3_seq4 comp56586_c2_seq11 comp57483_c0_seq1 comp56357_c4_seq12 comp55172_c2_seq1 comp52421_c0_seq5 comp57891_c0_seq7 comp52279_c0_seq2 comp48474_c0_seq1 comp53764_c4_seq5 comp54082_c0_seq1 comp52970_c0_seq3 comp50070_c0_seq3 comp37113_c1_seq1 comp48073_c1_seq1 comp54170_c0_seq4 comp54243_c1_seq1 comp44260_c0_seq1 comp57636_c0_seq4 comp55213_c0_seq3 comp48895_c0_seq1 comp58222_c2_seq1 comp50000_c1_seq2 comp52583_c0_seq1 comp50664_c0_seq4 comp54661_c2_seq3 comp42764_c0_seq1 comp57458_c2_seq4 comp44119_c0_seq2 comp57460_c0_seq1 comp55583_c0_seq1 comp53457_c0_seq2 comp52484_c0_seq2 comp57378_c0_seq3 comp52923_c0_seq1 comp31800_c1_seq1 comp43715_c0_seq1 comp55505_c3_seq16 comp57047_c0_seq1 comp57424_c0_seq4 comp53101_c0_seq1 comp46176_c0_seq1 comp53220_c0_seq7 comp54268_c4_seq14 comp56215_c2_seq1 comp52173_c0_seq2 comp57341_c5_seq3 comp56631_c1_seq1 comp57530_c7_seq23 comp57177_c0_seq7 comp56055_c2_seq1 comp55708_c0_seq5 comp54692_c0_seq4 comp52149_c0_seq5 comp57799_c1_seq6 comp57853_c1_seq3 comp56809_c2_seq6 comp52431_c0_seq1 comp54256_c1_seq3 comp48454_c1_seq3 comp51610_c0_seq1 comp49635_c0_seq1 comp49063_c0_seq2 comp56062_c5_seq6 comp49467_c4_seq1 comp53543_c1_seq2 comp50379_c1_seq4 comp53109_c0_seq4 comp31093_c0_seq1 comp51368_c0_seq1 comp46960_c0_seq1 comp45789_c0_seq1 comp52935_c0_seq3 comp53248_c0_seq1 comp49828_c0_seq1 comp54353_c0_seq2 comp53516_c0_seq4 comp57639_c1_seq1 comp57243_c1_seq5 comp49810_c0_seq3 comp56099_c0_seq9 comp50409_c0_seq1 comp56227_c1_seq2 comp57172_c2_seq1 comp50763_c2_seq1 comp49437_c0_seq1 comp44009_c0_seq2 comp57242_c2_seq9 comp56230_c0_seq1 comp52866_c1_seq7 comp30726_c0_seq1 comp49718_c0_seq1 comp47985_c0_seq1 comp56101_c1_seq3 comp56575_c0_seq6 comp57458_c2_seq22 comp56228_c2_seq3 comp54983_c0_seq3 comp55186_c4_seq3 comp42545_c0_seq2 comp57209_c0_seq4 comp51974_c1_seq2 comp48844_c0_seq6 comp56716_c6_seq5 comp56537_c0_seq4 comp52809_c0_seq1 comp54998_c0_seq7 comp29470_c0_seq1 comp56922_c1_seq7 comp45853_c2_seq1 comp51911_c0_seq1 comp51099_c0_seq5 comp56751_c0_seq1 comp57217_c0_seq2 comp47734_c0_seq4 comp53401_c1_seq1 comp50397_c0_seq2 comp48914_c0_seq1 comp52663_c0_seq4 comp56726_c2_seq7 comp57743_c5_seq2 comp42685_c0_seq3 comp46049_c0_seq1 comp57861_c0_seq3 comp50773_c3_seq1 comp43714_c0_seq1 comp52079_c1_seq2 comp57644_c0_seq6 comp55932_c0_seq7 comp55875_c1_seq10 comp49080_c1_seq1 comp56588_c4_seq1 comp48290_c0_seq1 comp48665_c0_seq2 comp54156_c0_seq3 comp58244_c0_seq7 comp57557_c6_seq1 comp58134_c0_seq6 comp57665_c1_seq21 comp57208_c3_seq34 comp56680_c1_seq5 comp58231_c1_seq10 comp56361_c2_seq11 comp54477_c1_seq3 comp54633_c1_seq2 comp57124_c1_seq24 comp32164_c0_seq1 comp51463_c0_seq1 comp51773_c1_seq1 comp56881_c0_seq2 comp52031_c3_seq2 comp51793_c1_seq3 comp58125_c1_seq18 comp56330_c0_seq4 comp58252_c3_seq8 comp54897_c0_seq2 comp43559_c0_seq1 comp51300_c0_seq6 comp51293_c2_seq4 comp57744_c0_seq4 comp52925_c0_seq3 comp58386_c0_seq1 comp38141_c0_seq1 comp60345_c0_seq1 comp52568_c4_seq1 comp56761_c2_seq1 comp56012_c1_seq5 comp50840_c2_seq1 comp55414_c5_seq14 comp54013_c0_seq2 comp56672_c0_seq2 comp48286_c0_seq1 comp57663_c0_seq13 comp50231_c0_seq1 comp48967_c3_seq1 comp47824_c2_seq1 comp39051_c0_seq1 comp53490_c1_seq5 comp43443_c0_seq1 comp58117_c1_seq6 comp56365_c0_seq4 comp53631_c0_seq11 comp55021_c0_seq2 comp51023_c0_seq1 comp57370_c3_seq4 comp56445_c0_seq6 comp55611_c0_seq1 comp56128_c1_seq13 comp52766_c0_seq2 comp27766_c0_seq1 comp56924_c4_seq2 comp54585_c0_seq1 comp51507_c0_seq1 comp46586_c0_seq1 comp49617_c0_seq1 comp50340_c0_seq1 comp57330_c2_seq6 comp118377_c0_seq1 comp55440_c0_seq2 comp38387_c0_seq2 comp172818_c0_seq1 comp52498_c0_seq3 comp46993_c0_seq3 comp53794_c0_seq1 comp36938_c0_seq1 comp58304_c0_seq1 comp57967_c0_seq1 comp57951_c0_seq26 comp57964_c0_seq6 comp57169_c1_seq3 comp43135_c0_seq2 comp52145_c0_seq1 comp58113_c4_seq3 comp54273_c0_seq4 comp55920_c1_seq2 comp56639_c1_seq1 comp52412_c2_seq2 comp47066_c0_seq3 comp34445_c0_seq3 comp43689_c0_seq2 comp57147_c0_seq2 comp51047_c2_seq1 comp47175_c0_seq1 comp59229_c0_seq1 comp57539_c2_seq9 comp54230_c0_seq1 comp54401_c8_seq27 comp56081_c1_seq3 comp57818_c1_seq5 comp56578_c0_seq2 comp57756_c3_seq2 comp27814_c0_seq2 comp57324_c1_seq23 comp56808_c0_seq1 comp53982_c4_seq4 comp56670_c0_seq6 comp53327_c0_seq3 comp47261_c0_seq1 comp55080_c4_seq8 comp56944_c0_seq11 comp54643_c0_seq1 comp55102_c2_seq1 comp54826_c0_seq1 comp56358_c0_seq3 comp55097_c2_seq1 comp56953_c2_seq3 comp57403_c1_seq2 comp55573_c3_seq1 comp50463_c0_seq1 comp58031_c2_seq22 comp56795_c0_seq3 comp53259_c1_seq5 comp50085_c0_seq2 comp54438_c3_seq4 comp49205_c2_seq1 comp58288_c0_seq4 comp51728_c0_seq3 comp57419_c0_seq8 comp55081_c0_seq1 comp33310_c0_seq1 comp56354_c1_seq11 comp33242_c1_seq1 comp56524_c2_seq3 comp58040_c4_seq4 comp55130_c1_seq3 comp57534_c1_seq4 comp172261_c0_seq1 comp53966_c0_seq1 comp54849_c3_seq5 comp49770_c8_seq1 comp57784_c1_seq3 comp55566_c0_seq1 comp54357_c0_seq3 comp52512_c3_seq4 comp57297_c1_seq2 comp54121_c0_seq2 comp56228_c5_seq1 comp57488_c3_seq11 comp53896_c0_seq2 comp58121_c0_seq2 comp37825_c0_seq1 comp28118_c1_seq1 comp56345_c6_seq1 comp58151_c3_seq6 comp52687_c1_seq2 comp46264_c0_seq1 comp51142_c0_seq1 comp58301_c1_seq10 comp58286_c1_seq12 comp54374_c0_seq2 comp57907_c11_seq5 comp46875_c0_seq1 comp50217_c0_seq1 comp45293_c0_seq1 comp48046_c0_seq2 comp48106_c0_seq3 comp48275_c0_seq2 comp30783_c0_seq1 comp44228_c0_seq1 comp56567_c1_seq5 comp56721_c0_seq1 comp58098_c2_seq13 comp47698_c1_seq1 comp57782_c0_seq1 comp46940_c0_seq3 comp54853_c2_seq12 comp46578_c0_seq1 comp56072_c0_seq2 comp57903_c0_seq7 comp56684_c0_seq5 comp50566_c0_seq1 comp55520_c3_seq1 comp57478_c0_seq5 comp58096_c3_seq47 comp53441_c0_seq3 comp46003_c0_seq1 comp55434_c3_seq1 comp55783_c0_seq1 comp32239_c0_seq1 comp57849_c2_seq5 comp52044_c2_seq4 comp56319_c0_seq3 comp56884_c2_seq1 comp28997_c0_seq1 comp46430_c0_seq3 comp48578_c0_seq1 comp57693_c0_seq3 comp51652_c0_seq2 comp56514_c0_seq2 comp99526_c0_seq1 comp30905_c0_seq1 comp57175_c3_seq5 comp47339_c1_seq1 comp52467_c0_seq10 comp58233_c8_seq7 comp56658_c0_seq7 comp53535_c0_seq1 comp46273_c0_seq1 comp54188_c1_seq1 comp50789_c1_seq5 comp33251_c0_seq1 comp46280_c0_seq4 comp55409_c0_seq5 comp56883_c0_seq1 comp55915_c1_seq5 comp56228_c4_seq1 comp44976_c1_seq1 comp49295_c0_seq2 comp58579_c0_seq1 comp57898_c3_seq10 comp56019_c1_seq2 comp49034_c0_seq1 comp49716_c1_seq1 comp48135_c0_seq1 comp50503_c1_seq2 comp56530_c1_seq1 comp47596_c0_seq1 comp52972_c0_seq2 comp45644_c0_seq1 comp56178_c0_seq1 comp54402_c2_seq1 comp54311_c0_seq3 comp57849_c3_seq1 comp53401_c1_seq10 comp57481_c0_seq10 comp54908_c1_seq9 comp55065_c0_seq5 comp45287_c1_seq1 comp57040_c3_seq8 comp57960_c0_seq2 comp54485_c5_seq1 comp57803_c0_seq9 comp37231_c0_seq1 comp50386_c1_seq1 comp58087_c8_seq3 comp213835_c0_seq1 comp56594_c7_seq2 comp56391_c2_seq1 comp53114_c3_seq1 comp54965_c0_seq3 comp53958_c0_seq2 comp53538_c3_seq1 comp58078_c0_seq56 comp51064_c0_seq1 comp58213_c0_seq12 comp56546_c0_seq1 comp45796_c1_seq1 comp50166_c1_seq1 comp49012_c0_seq1 comp49839_c0_seq1 comp51383_c6_seq1 comp48705_c0_seq2 comp53274_c2_seq2 comp48762_c0_seq1 comp54743_c0_seq1 comp57147_c0_seq6 comp56126_c0_seq1 comp48452_c0_seq2 comp52076_c0_seq1 comp58023_c1_seq12 comp58255_c1_seq13 comp29401_c0_seq1 comp28797_c0_seq1 comp52218_c0_seq2 comp51375_c0_seq4 comp55489_c0_seq2 comp54193_c11_seq2 comp56466_c0_seq2 comp44800_c0_seq1 comp31054_c0_seq1 comp57511_c0_seq9 comp53945_c0_seq2 comp50189_c1_seq1 comp50504_c0_seq1 comp55527_c0_seq1 comp43781_c0_seq1 comp57532_c3_seq12 comp49484_c0_seq2 comp30718_c0_seq2 comp42554_c0_seq2 comp54438_c1_seq1 comp56693_c1_seq5 comp55718_c1_seq2 comp43769_c0_seq2 comp56377_c2_seq3 comp51667_c4_seq1 comp54549_c4_seq1 comp57049_c0_seq2 comp46372_c3_seq1 comp55041_c0_seq2 comp42236_c0_seq1 comp49684_c0_seq10 comp57102_c2_seq10 comp45834_c2_seq2 comp52843_c0_seq16 comp55080_c2_seq2 comp55061_c0_seq6 comp55860_c0_seq3 comp52199_c0_seq3 comp56606_c0_seq2 comp57122_c0_seq4 comp49588_c0_seq2 comp57837_c0_seq1 comp56641_c0_seq8 comp53431_c2_seq12 comp56622_c1_seq15 comp52765_c0_seq3 comp46284_c0_seq1 comp50848_c0_seq7 comp54721_c0_seq5 comp49174_c1_seq4 comp55897_c0_seq2 comp57749_c0_seq9 comp54222_c0_seq2 comp57769_c2_seq4 comp52038_c2_seq1 comp57313_c0_seq4 comp56006_c3_seq3 comp53680_c0_seq4 comp55383_c2_seq2 comp52524_c0_seq5 comp54500_c1_seq2 comp50722_c4_seq2 comp47185_c0_seq1 comp57240_c1_seq2 comp57517_c0_seq24 comp58042_c1_seq5 comp57407_c1_seq1 comp48987_c0_seq1 comp57674_c1_seq7 comp57307_c2_seq2 comp56225_c0_seq4 comp51782_c2_seq1 comp47011_c0_seq1 comp54830_c8_seq2 comp48247_c0_seq1 comp54550_c0_seq5 comp44811_c0_seq2 comp57871_c0_seq7 comp50746_c0_seq1 comp53470_c0_seq4 comp57002_c2_seq2 comp57468_c4_seq2 comp52650_c0_seq2 comp54103_c0_seq1 comp28697_c0_seq1 comp45871_c0_seq1 comp56309_c1_seq3 comp55924_c0_seq3 comp56083_c0_seq1 comp52131_c1_seq2 comp54234_c0_seq2 comp41893_c0_seq1 comp52526_c0_seq4 comp46566_c0_seq2 comp51250_c0_seq3 comp53326_c0_seq1 comp53152_c0_seq2 comp56356_c0_seq1 comp58170_c1_seq31 comp52316_c2_seq1 comp37344_c0_seq1 comp58168_c3_seq16 comp58106_c0_seq1 comp48056_c1_seq1 comp57900_c0_seq2 comp50825_c0_seq1 comp57603_c18_seq2 comp51637_c1_seq2 comp49860_c0_seq3 comp53357_c0_seq1 comp55972_c2_seq4 comp57803_c4_seq2 comp48296_c0_seq1 comp37228_c0_seq1 comp57784_c3_seq1 comp49124_c0_seq1 comp57485_c0_seq3 comp54696_c1_seq1 comp57837_c1_seq1 comp52028_c0_seq1 comp55221_c2_seq1 comp54785_c1_seq2 comp47227_c1_seq1 comp41999_c0_seq1 comp58362_c0_seq1 comp52410_c1_seq1 comp57618_c1_seq1 comp41911_c1_seq1 comp55052_c2_seq2 comp40317_c0_seq1 comp49260_c0_seq3 comp55926_c0_seq2 comp39026_c0_seq1 comp51508_c2_seq7 comp58254_c0_seq6 comp57431_c4_seq6 comp29187_c0_seq1 comp55629_c1_seq8 comp57272_c2_seq11 comp57445_c0_seq14 comp48050_c0_seq3 comp56197_c4_seq7 comp55399_c0_seq2 comp57664_c3_seq4 comp186859_c0_seq1 comp58084_c1_seq3 comp48520_c1_seq2 comp58265_c0_seq1 comp48817_c0_seq4 comp53753_c6_seq1 comp56881_c0_seq6 comp29949_c1_seq1 comp28747_c0_seq1 comp55596_c1_seq2 comp54700_c3_seq1 comp46166_c1_seq1 comp55639_c0_seq5 comp56370_c0_seq2 comp54942_c1_seq8 comp57740_c6_seq8 comp49042_c3_seq1 comp45438_c3_seq1 comp57846_c0_seq10 comp57923_c0_seq3 comp56872_c2_seq3 comp56326_c8_seq1 comp41385_c0_seq1 comp57360_c0_seq7 comp58167_c1_seq3 comp57796_c0_seq1 comp48320_c0_seq1 comp51608_c0_seq5 comp56865_c0_seq4 comp51935_c0_seq2 comp55184_c5_seq4 comp54494_c2_seq4 comp56336_c0_seq14 comp50327_c0_seq2 comp53302_c1_seq1 comp48893_c0_seq1 comp51776_c0_seq2 comp55392_c0_seq10 comp58208_c0_seq5 comp56813_c3_seq8 comp57528_c11_seq2 comp57669_c0_seq31 comp47714_c0_seq1 comp48789_c0_seq2 comp51010_c0_seq1 comp45489_c0_seq1 comp58239_c2_seq13 comp121404_c0_seq1 comp52033_c0_seq14 comp55967_c0_seq3 comp53159_c1_seq2 comp52202_c0_seq3 comp47864_c0_seq3 comp57830_c2_seq6 comp55432_c1_seq3 comp27782_c0_seq1 comp29625_c0_seq2 comp65390_c0_seq1 comp53629_c0_seq4 comp55057_c2_seq1 comp42523_c0_seq1 comp47773_c0_seq1 comp57615_c1_seq11 comp56389_c1_seq2 comp57152_c4_seq6 comp57538_c0_seq4 comp53829_c0_seq2 comp57340_c0_seq9 comp57645_c3_seq12 comp42758_c0_seq1 comp50451_c0_seq1 comp50605_c2_seq1 comp53893_c0_seq1 comp54562_c0_seq1 comp55564_c0_seq1 comp56448_c1_seq1 comp56613_c2_seq1 comp44647_c0_seq1 comp56648_c1_seq8 comp50507_c0_seq1 comp56073_c1_seq2 comp52244_c0_seq5 comp49050_c0_seq1 comp51370_c0_seq4 comp43973_c0_seq1 comp54919_c0_seq2 comp45639_c0_seq1 comp47105_c0_seq1 comp51445_c1_seq1 comp57372_c1_seq8 comp29340_c0_seq1 comp58023_c1_seq6 comp57753_c3_seq12 comp55900_c8_seq2 comp52793_c3_seq2 comp36324_c0_seq2 comp51577_c0_seq1 comp56368_c0_seq6 comp50733_c0_seq2 comp51432_c0_seq2 comp56276_c0_seq4 comp42450_c0_seq1 comp56229_c1_seq3 comp56143_c0_seq6 comp57349_c0_seq1 comp56087_c0_seq2 comp46427_c0_seq2 comp51274_c0_seq1 comp42622_c0_seq1 comp54213_c0_seq5 comp49663_c0_seq1 comp48390_c2_seq4 comp48156_c0_seq1 comp57532_c3_seq16 comp52629_c0_seq2 comp55898_c0_seq17 comp49933_c0_seq1 comp55629_c1_seq5 comp33166_c0_seq1 comp55627_c0_seq2 comp57374_c0_seq3 comp51683_c1_seq1 comp54193_c7_seq1 comp46654_c1_seq2 comp36041_c2_seq1 comp30053_c0_seq1 comp57254_c9_seq7 comp56960_c0_seq4 comp55103_c0_seq7 comp56233_c6_seq2 comp56904_c0_seq15 comp50171_c0_seq1 comp53018_c1_seq5 comp58072_c1_seq3 comp56622_c5_seq7 comp55082_c1_seq1 comp54675_c0_seq2 comp35779_c0_seq1 comp31218_c1_seq1 comp58213_c0_seq7 comp47491_c0_seq1 comp55943_c0_seq2 comp53899_c0_seq8 comp32899_c1_seq1 comp48799_c0_seq1 comp53136_c0_seq4 comp51551_c0_seq4 comp56120_c0_seq2 comp57647_c3_seq12 comp57337_c0_seq7 comp56809_c4_seq5 comp56223_c0_seq1 comp55870_c0_seq6 comp57434_c4_seq3 comp56096_c4_seq20 comp43665_c0_seq3 comp17575_c0_seq1 comp48770_c0_seq1 comp48983_c0_seq1 comp48293_c1_seq5 comp54453_c0_seq1 comp58146_c4_seq16 comp55881_c1_seq3 comp54128_c0_seq2 comp50214_c2_seq1 comp54160_c2_seq1 comp57465_c0_seq4 comp52447_c1_seq1 comp57464_c0_seq5 comp53373_c0_seq2 comp57788_c0_seq1 comp41388_c0_seq1 comp58307_c0_seq5 comp32054_c0_seq1 comp56907_c3_seq9 comp56164_c0_seq2 comp52347_c2_seq1 comp43811_c1_seq1 comp4763_c0_seq1 comp58002_c0_seq14 comp58034_c0_seq2 comp51281_c0_seq1 comp55549_c4_seq2 comp55530_c1_seq1 comp41720_c0_seq1 comp52643_c4_seq2 comp53173_c0_seq6 comp48690_c0_seq1 comp57588_c8_seq2 comp56650_c1_seq3 comp57552_c2_seq8 comp48722_c0_seq2 comp55439_c0_seq5 comp46699_c0_seq1 comp44806_c0_seq2 comp58184_c0_seq9 comp56306_c1_seq1 comp54817_c9_seq5 comp54688_c0_seq2 comp48542_c1_seq5 comp55931_c0_seq1 comp54746_c0_seq4 comp57888_c4_seq12 comp56491_c1_seq1 comp53699_c1_seq5 comp55907_c1_seq1 comp51397_c0_seq1 comp52777_c1_seq3 comp55923_c0_seq4 comp54811_c1_seq4 comp57225_c8_seq1 comp50425_c0_seq3 comp56438_c3_seq2 comp57568_c3_seq15 comp53431_c2_seq1 comp30373_c0_seq1 comp54086_c0_seq8 comp52380_c0_seq2 comp54774_c0_seq3 comp68016_c0_seq1 comp50830_c0_seq2 comp54876_c0_seq2 comp47965_c0_seq1 comp57896_c2_seq10 comp55819_c0_seq5 comp52523_c0_seq1 comp52770_c0_seq2 comp52749_c0_seq1 comp52597_c0_seq3 comp48593_c1_seq1 comp57073_c5_seq12 comp32283_c0_seq1 comp53750_c1_seq11 comp52610_c0_seq3 comp57159_c1_seq7 comp52975_c1_seq2 comp56987_c9_seq10 comp55110_c1_seq3 comp53392_c0_seq6 comp57835_c0_seq3 comp57139_c0_seq3 comp47736_c1_seq1 comp55084_c0_seq6 comp57445_c0_seq7 comp29362_c0_seq2 comp56010_c0_seq1 comp48102_c0_seq1 comp55370_c0_seq2 comp37263_c0_seq1 comp55531_c0_seq3 comp46104_c0_seq1 comp56532_c3_seq2 comp50577_c4_seq1 comp45658_c0_seq1 comp54485_c0_seq1 comp57866_c0_seq1 comp51201_c1_seq1 comp56941_c0_seq8 comp58011_c1_seq12 comp58158_c3_seq10 comp56554_c4_seq1 comp28852_c0_seq1 comp53302_c0_seq1 comp57898_c3_seq15 comp54782_c1_seq7 comp51436_c0_seq5 comp57476_c2_seq8 comp31491_c0_seq1 comp58199_c0_seq5 comp46795_c0_seq1 comp45801_c1_seq2 comp53002_c0_seq2 comp55133_c0_seq3 comp32299_c0_seq1 comp52653_c0_seq1 comp41444_c0_seq1 comp57073_c1_seq6 comp42925_c1_seq1 comp57474_c0_seq14 comp42818_c2_seq1 comp57079_c2_seq6 comp48536_c0_seq6 comp53939_c1_seq3 comp37115_c0_seq2 comp30976_c0_seq1 comp55290_c0_seq3 comp50895_c0_seq1 comp58262_c4_seq14 comp58029_c1_seq11 comp53260_c0_seq2 comp56145_c10_seq8 comp49715_c0_seq1 comp53779_c2_seq6 comp52553_c2_seq1 comp53393_c0_seq2 comp55958_c3_seq8 comp57109_c0_seq1 comp8504_c0_seq1 comp28232_c0_seq1 comp45482_c0_seq1 comp53688_c0_seq1 comp46048_c0_seq1 comp57813_c5_seq6 comp55936_c0_seq2 comp48890_c0_seq4 comp57524_c0_seq7 comp57933_c0_seq12 comp57547_c1_seq4 comp51745_c0_seq1 comp29190_c0_seq1 comp57608_c1_seq9 comp42137_c0_seq1 comp56209_c0_seq2 comp29676_c0_seq1 comp54567_c0_seq3 comp57441_c1_seq2 comp45296_c0_seq3 comp51436_c1_seq1 comp48865_c0_seq3 comp55621_c1_seq1 comp58247_c1_seq4 comp49954_c2_seq1 comp49684_c0_seq7 comp55971_c0_seq1 comp52286_c1_seq4 comp57520_c0_seq6 comp47479_c0_seq1 comp56938_c4_seq11 comp56874_c4_seq34 comp50855_c0_seq2 comp58104_c2_seq5 comp43815_c0_seq1 comp55733_c0_seq2 comp49109_c0_seq1 comp56196_c2_seq4 comp52649_c0_seq4 comp57138_c4_seq3 comp54800_c1_seq1 comp57663_c0_seq8 comp56810_c1_seq5 comp131442_c0_seq1 comp41045_c0_seq1 comp48038_c0_seq5 comp52068_c1_seq1 comp57950_c0_seq7 comp56326_c5_seq4 comp40887_c0_seq1 comp50675_c2_seq1 comp47921_c0_seq1 comp56389_c1_seq1 comp57641_c1_seq4 comp54117_c0_seq1 comp52318_c0_seq1 comp48292_c0_seq4 comp57579_c1_seq6 comp57034_c0_seq4 comp41507_c0_seq1 comp55097_c1_seq1 comp58086_c1_seq7 comp58262_c4_seq2 comp51851_c0_seq5 comp51618_c0_seq1 comp47791_c2_seq1 comp46931_c0_seq3 comp51004_c0_seq1 comp56976_c0_seq1 comp56215_c2_seq5 comp52012_c5_seq4 comp57508_c0_seq2 comp53385_c0_seq3 comp50916_c0_seq1 comp51447_c0_seq1 comp43760_c0_seq1 comp53310_c0_seq3 comp55184_c16_seq3 comp57143_c0_seq13 comp57201_c0_seq5 comp52474_c1_seq3 comp48885_c2_seq1 comp55020_c1_seq1 comp55900_c8_seq12 comp58245_c1_seq44 comp54669_c0_seq4 comp55084_c0_seq14 comp31683_c0_seq1 comp57499_c6_seq23 comp52257_c0_seq3 comp57856_c15_seq20 comp30715_c0_seq1 comp53812_c1_seq1 comp56068_c0_seq1 comp42584_c4_seq1 comp58048_c3_seq1 comp52083_c0_seq3 comp51804_c1_seq3 comp56292_c0_seq1 comp57041_c0_seq1 comp52884_c1_seq4 comp57838_c0_seq1 comp51426_c0_seq1 comp53866_c0_seq1 comp53881_c1_seq2 comp52792_c0_seq1 comp57398_c0_seq1 comp47887_c0_seq1 comp41257_c0_seq4 comp50736_c0_seq1 comp30724_c0_seq1 comp54038_c0_seq1 comp56534_c3_seq1 comp54341_c0_seq1 comp53258_c0_seq1 comp50770_c0_seq1 comp54296_c2_seq3 comp45054_c0_seq1 comp53218_c0_seq2 comp57634_c3_seq7 comp44182_c1_seq1 comp47444_c0_seq1 comp29257_c0_seq1 comp48801_c0_seq3 comp52802_c0_seq6 comp32663_c0_seq1 comp56535_c0_seq3 comp50335_c0_seq2 comp57543_c0_seq6 comp58113_c3_seq1 comp48833_c0_seq3 comp43266_c0_seq1 comp53562_c4_seq6 comp58218_c0_seq11 comp55047_c0_seq2 comp55033_c1_seq3 comp57712_c4_seq4 comp58187_c3_seq2 comp48796_c2_seq1 comp52426_c0_seq1 comp53466_c1_seq1 comp29770_c1_seq1 comp57709_c0_seq2 comp51409_c1_seq2 comp40322_c1_seq1 comp57179_c2_seq1 comp58144_c3_seq2 comp30464_c0_seq2 comp52648_c0_seq1 comp53010_c0_seq4 comp52961_c0_seq4 comp56992_c2_seq6 comp58259_c1_seq21 comp57975_c2_seq4 comp51589_c0_seq2 comp55116_c0_seq4 comp56378_c3_seq2 comp57055_c4_seq2 comp52570_c0_seq4 comp51121_c0_seq1 comp49549_c0_seq2 comp57098_c4_seq10 comp50705_c1_seq1 comp54913_c0_seq8 comp56217_c0_seq1 comp56708_c0_seq5 comp53614_c0_seq2 comp54803_c0_seq1 comp48430_c0_seq5 comp30577_c0_seq1 comp59021_c0_seq1 comp56435_c1_seq3 comp54007_c0_seq2 comp55088_c1_seq1 comp53436_c0_seq2 comp50389_c0_seq7 comp29063_c1_seq1 comp45662_c1_seq1 comp46340_c0_seq3 comp54225_c2_seq9 comp48014_c1_seq1 comp45050_c1_seq1 comp49303_c0_seq1 comp57306_c1_seq5 comp30532_c1_seq1 comp55709_c0_seq1 comp36882_c0_seq1 comp48558_c0_seq1 comp55454_c1_seq1 comp52353_c1_seq2 comp48399_c0_seq6 comp58229_c0_seq12 comp48995_c0_seq1 comp42712_c0_seq2 comp57907_c11_seq1 comp57524_c1_seq1 comp49161_c0_seq4 comp32575_c0_seq1 comp55504_c0_seq2 comp52480_c2_seq4 comp49781_c1_seq1 comp59224_c0_seq1 comp87085_c0_seq1 comp56852_c2_seq1 comp51599_c2_seq1 comp48510_c2_seq1 comp57704_c3_seq5 comp53881_c2_seq1 comp47808_c1_seq1 comp56684_c0_seq2 comp57619_c0_seq14 comp53027_c0_seq1 comp52437_c0_seq3 comp52470_c0_seq1 comp56299_c2_seq15 comp49712_c0_seq2 comp55647_c0_seq6 comp57073_c5_seq11 comp43861_c3_seq1 comp43207_c2_seq2 comp52981_c0_seq1 comp53435_c3_seq1 comp56849_c4_seq7 comp54650_c1_seq3 comp46627_c0_seq1 comp43478_c0_seq1 comp53309_c0_seq3 comp56037_c0_seq2 comp56015_c0_seq3 comp52035_c1_seq2 comp57579_c1_seq7 comp50506_c0_seq4 comp57951_c0_seq23 comp54123_c0_seq1 comp57810_c0_seq8 comp53881_c1_seq4 comp45312_c1_seq1 comp55629_c1_seq11 comp51699_c1_seq2 comp53622_c0_seq2 comp58072_c1_seq5 comp54557_c0_seq2 comp54527_c2_seq1 comp54763_c2_seq1 comp47395_c0_seq1 comp47637_c0_seq2 comp49316_c0_seq1 comp52075_c0_seq2 comp57483_c0_seq4 comp32075_c0_seq1 comp57349_c0_seq4 comp44667_c0_seq1 comp53132_c0_seq4 comp49197_c0_seq1 comp55774_c4_seq1 comp47847_c0_seq1 comp56134_c0_seq2 comp53646_c0_seq2 comp29067_c0_seq1 comp51106_c1_seq11 comp57056_c1_seq6 comp50104_c1_seq2 comp58001_c4_seq2 comp57659_c1_seq5 comp57878_c0_seq3 comp29827_c0_seq1 comp56136_c0_seq10 comp56336_c0_seq9 comp46155_c0_seq4 comp46296_c2_seq1 comp46087_c0_seq1 comp48470_c0_seq2 comp47195_c2_seq1 comp52777_c0_seq3 comp50378_c1_seq2 comp53580_c1_seq1 comp53386_c0_seq3 comp58137_c2_seq15 comp56692_c3_seq3 comp49979_c1_seq4 comp57849_c2_seq10 comp30024_c0_seq1 comp58295_c0_seq38 comp56661_c1_seq2 comp57239_c2_seq20 comp49997_c0_seq1 comp57458_c2_seq2 comp45800_c1_seq1 comp57118_c0_seq7 comp54914_c1_seq2 comp57725_c0_seq3 comp57716_c2_seq10 comp54944_c2_seq1 comp47431_c1_seq1 comp53150_c0_seq1 comp57973_c1_seq3 comp57357_c2_seq6 comp55775_c3_seq2 comp50217_c1_seq2 comp54872_c1_seq1 comp46124_c0_seq2 comp52446_c0_seq1 comp47743_c0_seq2 comp52580_c0_seq1 comp46834_c0_seq1 comp49429_c0_seq13 comp30634_c0_seq1 comp58087_c0_seq3 comp55656_c3_seq1 comp47962_c0_seq2 comp50725_c1_seq1 comp50805_c0_seq1 comp57605_c2_seq17 comp57425_c1_seq6 comp32412_c0_seq1 comp31497_c0_seq1 comp57729_c2_seq13 comp37382_c0_seq1 comp57406_c0_seq4 comp57448_c2_seq5 comp47333_c0_seq2 comp51594_c1_seq1 comp44520_c0_seq1 comp54057_c0_seq2 comp54040_c0_seq1 comp50640_c0_seq1 comp58043_c0_seq5 comp53779_c2_seq10 comp44983_c0_seq1 comp54680_c0_seq1 comp51333_c0_seq1 comp55744_c8_seq28 comp50004_c1_seq2 comp57822_c2_seq6 comp53167_c0_seq3 comp46770_c0_seq1 comp214509_c0_seq1 comp36898_c1_seq1 comp56567_c1_seq2 comp52286_c1_seq1 comp54033_c2_seq2 comp43305_c0_seq1 comp52091_c0_seq2 comp56092_c0_seq6 comp54703_c2_seq3 comp57847_c0_seq4 comp54497_c1_seq1 comp48904_c0_seq2 comp46414_c0_seq1 comp52269_c0_seq2 comp48942_c0_seq1 comp54223_c0_seq1 comp45731_c1_seq1 comp57641_c1_seq3 comp50669_c0_seq8 comp57132_c0_seq2 comp54274_c0_seq2 comp56892_c0_seq2 comp56351_c1_seq8 comp36543_c1_seq1 comp56207_c1_seq4 comp54549_c1_seq4 comp56414_c0_seq2 comp48630_c0_seq1 comp55510_c0_seq5 comp57907_c1_seq3 comp57836_c1_seq1 comp51212_c1_seq10 comp58205_c9_seq2 comp52888_c0_seq2 comp42685_c0_seq2 comp55659_c0_seq4 comp45760_c0_seq1 comp56855_c1_seq1 comp53187_c0_seq1 comp58226_c0_seq11 comp55120_c0_seq3 comp56968_c0_seq4 comp29217_c1_seq1 comp51814_c0_seq1 comp55203_c1_seq5 comp52089_c1_seq4 comp53965_c0_seq1 comp53982_c4_seq3 comp57369_c1_seq5 comp51130_c1_seq1 comp55110_c3_seq18 comp50394_c0_seq1 comp57806_c0_seq6 comp53006_c0_seq1 comp44623_c0_seq1 comp52811_c0_seq1 comp57398_c0_seq2 comp57871_c0_seq6 comp41081_c1_seq1 comp55974_c0_seq5 comp54928_c0_seq3 comp56659_c2_seq1 comp54792_c0_seq1 comp32030_c0_seq1 comp48903_c1_seq2 comp48680_c0_seq1 comp49418_c0_seq3 comp52258_c2_seq1 comp50431_c0_seq2 comp57844_c3_seq7 comp57570_c0_seq4 comp32724_c0_seq2 comp46186_c0_seq2 comp51121_c0_seq2 comp50849_c1_seq3 comp40616_c0_seq1 comp47801_c0_seq2 comp57950_c3_seq1 comp57895_c0_seq3 comp46111_c1_seq2 comp57792_c0_seq1 comp54612_c3_seq1 comp52756_c0_seq1 comp28254_c1_seq1 comp54361_c0_seq3 comp29625_c0_seq1 comp53636_c0_seq3 comp57679_c1_seq8 comp55900_c8_seq39 comp54262_c2_seq1 comp58287_c0_seq8 comp53061_c4_seq1 comp52494_c0_seq4 comp49014_c1_seq1 comp46866_c0_seq1 comp27942_c0_seq1 comp53431_c2_seq2 comp51189_c3_seq1 comp57728_c2_seq16 comp56696_c2_seq13 comp58284_c6_seq6 comp52435_c0_seq1 comp55424_c0_seq7 comp52737_c0_seq2 comp56396_c0_seq7 comp47265_c0_seq1 comp57848_c0_seq3 comp56746_c6_seq1 comp47153_c0_seq1 comp55861_c2_seq3 comp42245_c1_seq1 comp49371_c1_seq1 comp50536_c1_seq1 comp50640_c1_seq1 comp51597_c1_seq1 comp48789_c0_seq4 comp52692_c2_seq1 comp57604_c1_seq15 comp41412_c0_seq1 comp53703_c2_seq1 comp45894_c0_seq1 comp47736_c0_seq1 comp56938_c4_seq9 comp28622_c0_seq1 comp56607_c0_seq1 comp55128_c6_seq4 comp30938_c0_seq2 comp42849_c0_seq1 comp57153_c1_seq20 comp55883_c2_seq3 comp47467_c0_seq1 comp37458_c0_seq1 comp56737_c5_seq9 comp47261_c0_seq4 comp57084_c0_seq4 comp55461_c2_seq1 comp57302_c0_seq1 comp55569_c0_seq6 comp56332_c0_seq7 comp51663_c1_seq3 comp53730_c1_seq4 comp56399_c1_seq2 comp57635_c0_seq1 comp40665_c0_seq1 comp56868_c3_seq6 comp52941_c1_seq1 comp53958_c5_seq1 comp56372_c1_seq6 comp53375_c0_seq1 comp55015_c0_seq1 comp55623_c9_seq4 comp57341_c4_seq2 comp60071_c0_seq1 comp32907_c0_seq2 comp43374_c2_seq1 comp57332_c1_seq1 comp54008_c0_seq2 comp58064_c0_seq9 comp33004_c0_seq1 comp58168_c3_seq18 comp56422_c2_seq1 comp50197_c2_seq1 comp52521_c0_seq2 comp56173_c0_seq7 comp58087_c4_seq1 comp51851_c0_seq1 comp57951_c0_seq12 comp56079_c4_seq3 comp56700_c9_seq2 comp47982_c1_seq1 comp56511_c0_seq1 comp49628_c0_seq1 comp57688_c0_seq4 comp32632_c0_seq1 comp50024_c0_seq1 comp46485_c0_seq1 comp58216_c0_seq2 comp58094_c0_seq20 comp44611_c2_seq1 comp37734_c0_seq1 comp54303_c0_seq3 comp46679_c0_seq3 comp28582_c0_seq1 comp56516_c2_seq9 comp54889_c3_seq5 comp56845_c3_seq1 comp52263_c0_seq5 comp47268_c0_seq2 comp56401_c0_seq1 comp56814_c1_seq6 comp51735_c0_seq2 comp52415_c1_seq3 comp42357_c6_seq1 comp57725_c0_seq11 comp33875_c1_seq1 comp49546_c2_seq1 comp53235_c0_seq1 comp57768_c0_seq2 comp49251_c0_seq2 comp51584_c1_seq3 comp58059_c0_seq2 comp49596_c1_seq1 comp58183_c2_seq1 comp44141_c1_seq1 comp53720_c0_seq1 comp56594_c5_seq1 comp45890_c0_seq1 comp58134_c0_seq1 comp50757_c1_seq1 comp53391_c0_seq5 comp54143_c0_seq3 comp50471_c1_seq1 comp29416_c0_seq1 comp58949_c0_seq1 comp55112_c1_seq2 comp55196_c0_seq9 comp54029_c0_seq2 comp58159_c0_seq1 comp58021_c3_seq12 comp58223_c3_seq1 comp50330_c0_seq1 comp55426_c2_seq1 comp58267_c1_seq10 comp47720_c0_seq1 comp52606_c1_seq1 comp44440_c0_seq1 comp49088_c0_seq1 comp49306_c0_seq1 comp41912_c0_seq3 comp50368_c0_seq2 comp47353_c0_seq2 comp49053_c0_seq2 comp52309_c0_seq2 comp55133_c0_seq5 comp58250_c1_seq7 comp51026_c0_seq2 comp57618_c0_seq17 comp57628_c2_seq1 comp61613_c0_seq1 comp55623_c2_seq15 comp47084_c0_seq1 comp57175_c0_seq5 comp59490_c0_seq1 comp57502_c6_seq7 comp57882_c0_seq1 comp48508_c0_seq1 comp53886_c0_seq7 comp57825_c2_seq15 comp58258_c0_seq6 comp55103_c0_seq5 comp53631_c0_seq7 comp56688_c5_seq2 comp48066_c0_seq2 comp49259_c0_seq1 comp56862_c2_seq1 comp57237_c7_seq1 comp44848_c1_seq1 comp55961_c0_seq2 comp49519_c0_seq1 comp57322_c0_seq2 comp56907_c3_seq6 comp53442_c1_seq4 comp56606_c0_seq3 comp55995_c10_seq41 comp57840_c0_seq8 comp56819_c0_seq1 comp50400_c1_seq4 comp57775_c0_seq3 comp52544_c0_seq2 comp50747_c0_seq1 comp57164_c0_seq8 comp40702_c1_seq1 comp57894_c0_seq6 comp50559_c1_seq3 comp53955_c2_seq8 comp54927_c4_seq2 comp54406_c6_seq1 comp57708_c2_seq10 comp47856_c0_seq1 comp56327_c2_seq6 comp55653_c0_seq3 comp57785_c0_seq2 comp140679_c0_seq1 comp56348_c2_seq2 comp57896_c5_seq1 comp38959_c0_seq1 comp55858_c0_seq3 comp43294_c0_seq1 comp51399_c0_seq2 comp57468_c0_seq11 comp47057_c1_seq1 comp52243_c1_seq1 comp60016_c0_seq1 comp29482_c0_seq1 comp52031_c2_seq2 comp37757_c0_seq1 comp39633_c0_seq1 comp57699_c1_seq4 comp47058_c0_seq1 comp41479_c4_seq1 comp57317_c3_seq14 comp34167_c0_seq1 comp56129_c4_seq14 comp51337_c3_seq2 comp51104_c0_seq3 comp56874_c4_seq17 comp121586_c0_seq1 comp53189_c0_seq1 comp58240_c1_seq1 comp50294_c0_seq4 comp31429_c0_seq1 comp58028_c2_seq1 comp56126_c1_seq1 comp46693_c1_seq1 comp53152_c0_seq3 comp54288_c0_seq1 comp48953_c0_seq1 comp57189_c2_seq1 comp48325_c2_seq3 comp54125_c0_seq4 comp56151_c0_seq22 comp55972_c2_seq7 comp29726_c0_seq1 comp53567_c1_seq5 comp58065_c1_seq2 comp52602_c0_seq1 comp58259_c1_seq16 comp48327_c0_seq1 comp55732_c1_seq1 comp54305_c4_seq1 comp57041_c0_seq3 comp58163_c3_seq5 comp46467_c0_seq4 comp54740_c0_seq4 comp49807_c1_seq1 comp48794_c1_seq1 comp57066_c0_seq3 comp48836_c2_seq1 comp38974_c4_seq1 comp46149_c0_seq2 comp52843_c0_seq12 comp50440_c0_seq1 comp54573_c0_seq3 comp51500_c0_seq4 comp48614_c1_seq1 comp48344_c0_seq1 comp56363_c0_seq1 comp56904_c0_seq9 comp54815_c2_seq5 comp52740_c0_seq2 comp48913_c0_seq1 comp48906_c1_seq1 comp48114_c0_seq2 comp50554_c0_seq2 comp42975_c0_seq1 comp54339_c0_seq5 comp51598_c0_seq2 comp52337_c1_seq1 comp55181_c0_seq4 comp55168_c3_seq1 comp56574_c0_seq8 comp57361_c0_seq1 comp49057_c0_seq1 comp51378_c1_seq1 comp50401_c1_seq3 comp53606_c0_seq2 comp56618_c0_seq2 comp57467_c3_seq1 comp56552_c2_seq15 comp50244_c0_seq1 comp50584_c0_seq2 comp31290_c0_seq2 comp50251_c0_seq2 comp58108_c1_seq13 comp48721_c0_seq2 comp57255_c0_seq4 comp52349_c2_seq1 comp48787_c1_seq2 comp154828_c0_seq1 comp36073_c1_seq1 comp41847_c0_seq2 comp39276_c0_seq2 comp41281_c0_seq1 comp139495_c0_seq1 comp56580_c0_seq4 comp52006_c0_seq4 comp56665_c1_seq7 comp53832_c0_seq1 comp55109_c0_seq1 comp47368_c0_seq1 comp55994_c0_seq8 comp45063_c1_seq1 comp51031_c1_seq1 comp55231_c0_seq4 comp57212_c2_seq3 comp57394_c1_seq2 comp55687_c0_seq4 comp56315_c0_seq3 comp50579_c0_seq1 comp48389_c0_seq1 comp56950_c0_seq2 comp57291_c4_seq1 comp49932_c0_seq1 comp49232_c0_seq1 comp55524_c0_seq3 comp35360_c2_seq1 comp57494_c7_seq1 comp54434_c0_seq2 comp55314_c0_seq4 comp56031_c1_seq5 comp47398_c4_seq1 comp55158_c0_seq2 comp54849_c0_seq1 comp55317_c0_seq4 comp51401_c0_seq9 comp48924_c0_seq2 comp30287_c0_seq1 comp32675_c0_seq1 comp54849_c3_seq2 comp54409_c2_seq3 comp57844_c3_seq11 comp45668_c0_seq1 comp55473_c0_seq5 comp55555_c8_seq5 comp54022_c0_seq4 comp39196_c0_seq1 comp56374_c0_seq1 comp39786_c0_seq1 comp53400_c1_seq2 comp57807_c7_seq2 comp55791_c0_seq2 comp52896_c0_seq2 comp52438_c0_seq9 comp54203_c0_seq2 comp47090_c1_seq2 comp50622_c1_seq1 comp29003_c0_seq1 comp46543_c0_seq2 comp51263_c0_seq1 comp57694_c7_seq6 comp55538_c1_seq4 comp44906_c0_seq1 comp56073_c0_seq4 comp46588_c0_seq1 comp51636_c1_seq2 comp54817_c9_seq4 comp56084_c3_seq1 comp57824_c1_seq7 comp50417_c1_seq2 comp53267_c6_seq2 comp54930_c0_seq5 comp31531_c0_seq1 comp57950_c0_seq11 comp47098_c0_seq1 comp29697_c0_seq1 comp57109_c2_seq1 comp57986_c3_seq5 comp57753_c2_seq2 comp56681_c0_seq2 comp29846_c0_seq1 comp52125_c0_seq2 comp47347_c0_seq2 comp57711_c0_seq5 comp53846_c1_seq1 comp47832_c0_seq1 comp53934_c0_seq2 comp55505_c3_seq14 comp57322_c0_seq6 comp56515_c3_seq3 comp48250_c0_seq3 comp164650_c0_seq1 comp54405_c0_seq3 comp58256_c1_seq26 comp54518_c0_seq1 comp30601_c0_seq1 comp45983_c0_seq1 comp53102_c1_seq2 comp45766_c2_seq1 comp45842_c0_seq4 comp57448_c1_seq1 comp45237_c0_seq2 comp51413_c2_seq3 comp47079_c0_seq1 comp55365_c0_seq2 comp57543_c0_seq3 comp57800_c0_seq2 comp57178_c0_seq9 comp58133_c3_seq3 comp50195_c1_seq3 comp55141_c0_seq14 comp56907_c5_seq1 comp56709_c1_seq5 comp51301_c0_seq1 comp52672_c0_seq2 comp57657_c2_seq1 comp57499_c6_seq28 comp54468_c1_seq11 comp58206_c6_seq21 comp57506_c0_seq6 comp55662_c0_seq1 comp46327_c0_seq1 comp55609_c0_seq3 comp56503_c0_seq2 comp47344_c2_seq1 comp46993_c0_seq4 comp55977_c1_seq2 comp47434_c0_seq1 comp50013_c0_seq3 comp57089_c0_seq4 comp48293_c1_seq3 comp47421_c2_seq1 comp42074_c0_seq1 comp56730_c1_seq2 comp52902_c0_seq1 comp57335_c0_seq14 comp46351_c0_seq1 comp52635_c0_seq1 comp58167_c0_seq5 comp36660_c0_seq1 comp45736_c0_seq1 comp47024_c1_seq1 comp48454_c1_seq1 comp48385_c2_seq1 comp51857_c1_seq4 comp50246_c0_seq2 comp53742_c1_seq2 comp57998_c1_seq10 comp45966_c1_seq1 comp54837_c2_seq1 comp49856_c0_seq2 comp55506_c0_seq9 comp58086_c1_seq19 comp34135_c0_seq1 comp55593_c1_seq2 comp52139_c0_seq7 comp53524_c0_seq3 comp44280_c0_seq3 comp52033_c0_seq8 comp58048_c4_seq36 comp56942_c0_seq4 comp55643_c0_seq2 comp58126_c1_seq4 comp43665_c0_seq2 comp57662_c0_seq3 comp56856_c2_seq6 comp57837_c1_seq9 comp57525_c0_seq13 comp58226_c0_seq3 comp46157_c0_seq1 comp48797_c0_seq2 comp4336_c0_seq1 comp52675_c0_seq2 comp53515_c0_seq2 comp45806_c0_seq1 comp58059_c7_seq1 comp56345_c0_seq1 comp48921_c0_seq4 comp40500_c0_seq1 comp57645_c1_seq8 comp50351_c0_seq1 comp46218_c0_seq2 comp33431_c0_seq2 comp43977_c1_seq2 comp56989_c0_seq2 comp56486_c1_seq2 comp42742_c1_seq2 comp51728_c0_seq2 comp54192_c0_seq1 comp58223_c6_seq23 comp55414_c5_seq13 comp56075_c1_seq4 comp57325_c2_seq2 comp47286_c0_seq1 comp56858_c3_seq6 comp52437_c0_seq1 comp55436_c3_seq1 comp44869_c0_seq1 comp56754_c0_seq8 comp56218_c3_seq2 comp46038_c0_seq1 comp57001_c3_seq1 comp58037_c4_seq3 comp58152_c1_seq9 comp55847_c0_seq10 comp43117_c0_seq2 comp34966_c0_seq2 comp53985_c0_seq3 comp238687_c0_seq1 comp49070_c0_seq1 comp43281_c0_seq1 comp58081_c1_seq13 comp47717_c3_seq1 comp57658_c6_seq7 comp50831_c0_seq1 comp57904_c3_seq8 comp29063_c0_seq1 comp50662_c0_seq2 comp58144_c3_seq7 comp48706_c0_seq1 comp52784_c0_seq3 comp55319_c0_seq4 comp48358_c1_seq1 comp48228_c0_seq1 comp58050_c3_seq3 comp57831_c2_seq14 comp48263_c0_seq2 comp42110_c0_seq1 comp58196_c0_seq23 comp51999_c0_seq3 comp57936_c5_seq2 comp32911_c1_seq1 comp49999_c3_seq1 comp57931_c0_seq6 comp58040_c4_seq2 comp56864_c0_seq2 comp42706_c1_seq1 comp47557_c0_seq1 comp43913_c1_seq1 comp56726_c2_seq24 comp54273_c0_seq2 comp47048_c0_seq1 comp49823_c0_seq1 comp57660_c0_seq3 comp53669_c1_seq3 comp53001_c0_seq2 comp57774_c1_seq1 comp49069_c0_seq1 comp46789_c0_seq1 comp51891_c0_seq1 comp54900_c0_seq1 comp48289_c1_seq1 comp55374_c0_seq3 comp27822_c0_seq1 comp53092_c1_seq1 comp44999_c0_seq2 comp49087_c0_seq1 comp54548_c0_seq1 comp54539_c0_seq1 comp61225_c0_seq1 comp157016_c0_seq1 comp50872_c2_seq1 comp55689_c1_seq8 comp52396_c2_seq1 comp47840_c0_seq1 comp48300_c0_seq2 comp43806_c2_seq1 comp44866_c1_seq1 comp51689_c0_seq6 comp56346_c0_seq23 comp43512_c0_seq1 comp55277_c0_seq1 comp57831_c2_seq6 comp56197_c1_seq3 comp44836_c0_seq1 comp58141_c1_seq5 comp53222_c0_seq1 comp48615_c1_seq1 comp54869_c0_seq2 comp51752_c1_seq1 comp50839_c0_seq5 comp57326_c5_seq9 comp58163_c4_seq1 comp45937_c0_seq1 comp50259_c0_seq1 comp55343_c1_seq10 comp56815_c3_seq1 comp53919_c0_seq4 comp55081_c0_seq2 comp55599_c0_seq1 comp51711_c0_seq1 comp49703_c0_seq1 comp52354_c0_seq2 comp56042_c0_seq7 comp45015_c2_seq3 comp58238_c1_seq21 comp56595_c0_seq17 comp54599_c0_seq3 comp43183_c0_seq1 comp57966_c0_seq15 comp42430_c0_seq2 comp36598_c1_seq1 comp50181_c1_seq4 comp53538_c6_seq1 comp51525_c0_seq1 comp49134_c1_seq3 comp50705_c0_seq3 comp54469_c0_seq3 comp50582_c1_seq2 comp52113_c2_seq1 comp56827_c0_seq6 comp56601_c0_seq1 comp45952_c0_seq1 comp50209_c0_seq4 comp50754_c1_seq1 comp50411_c0_seq2 comp51125_c0_seq1 comp50122_c0_seq3 comp44295_c0_seq1 comp56838_c0_seq6 comp50621_c0_seq1 comp50179_c0_seq1 comp52311_c0_seq2 comp36694_c0_seq1 comp58872_c0_seq1 comp54357_c0_seq2 comp49989_c0_seq2 comp52407_c0_seq1 comp5139_c0_seq1 comp51897_c0_seq2 comp57583_c0_seq16 comp50403_c0_seq4 comp57280_c1_seq4 comp56121_c0_seq3 comp53971_c0_seq3 comp57171_c0_seq2 comp35184_c0_seq1 comp52004_c3_seq3 comp55504_c5_seq3 comp56882_c0_seq4 comp37372_c1_seq1 comp111959_c0_seq1 comp55954_c0_seq1 comp50354_c0_seq2 comp31746_c0_seq1 comp55606_c3_seq1 comp53668_c0_seq3 comp57418_c0_seq2 comp48803_c2_seq1 comp50786_c0_seq2 comp31303_c0_seq1 comp58286_c1_seq27 comp47301_c0_seq1 comp56264_c0_seq3 comp52327_c2_seq3 comp57729_c2_seq7 comp49042_c1_seq2 comp51488_c0_seq3 comp51154_c0_seq4 comp50828_c0_seq1 comp55737_c0_seq6 comp58098_c2_seq5 comp54184_c0_seq6 comp45696_c0_seq1 comp57013_c1_seq3 comp51296_c0_seq2 comp52984_c0_seq1 comp55416_c1_seq6 comp57828_c2_seq1 comp44112_c0_seq1 comp58133_c5_seq1 comp55644_c0_seq8 comp58103_c2_seq5 comp33239_c0_seq1 comp54894_c0_seq5 comp57109_c0_seq4 comp57665_c1_seq23 comp56534_c1_seq5 comp56698_c2_seq2 comp112430_c0_seq1 comp52717_c1_seq1 comp55900_c1_seq1 comp55899_c0_seq1 comp57544_c6_seq3 comp54086_c0_seq17 comp56268_c0_seq6 comp63297_c0_seq1 comp47200_c1_seq1 comp48097_c1_seq1 comp50441_c2_seq1 comp33757_c0_seq1 comp57474_c3_seq2 comp49448_c0_seq1 comp47753_c0_seq1 comp55799_c0_seq1 comp56810_c1_seq6 comp50877_c2_seq1 comp46195_c1_seq3 comp54788_c0_seq6 comp46737_c1_seq1 comp45602_c0_seq1 comp52182_c0_seq1 comp56886_c0_seq3 comp55260_c1_seq3 comp57764_c1_seq4 comp58253_c1_seq2 comp54889_c1_seq1 comp54079_c1_seq1 comp43853_c3_seq1 comp57388_c2_seq11 comp56059_c0_seq3 comp53034_c0_seq1 comp56049_c0_seq7 comp52524_c0_seq3 comp56941_c1_seq2 comp51489_c0_seq4 comp45799_c0_seq1 comp47477_c0_seq2 comp58034_c0_seq5 comp54130_c0_seq1 comp41793_c0_seq1 comp52954_c0_seq7 comp56603_c3_seq5 comp57637_c0_seq2 comp57538_c0_seq7 comp53519_c0_seq2 comp56341_c3_seq10 comp52956_c0_seq1 comp42145_c2_seq1 comp55500_c0_seq1 comp53380_c0_seq3 comp53527_c2_seq8 comp37278_c1_seq1 comp30385_c0_seq1 comp44499_c0_seq1 comp57913_c0_seq5 comp32884_c0_seq1 comp55201_c0_seq3 comp48125_c0_seq1 comp56670_c0_seq11 comp58111_c4_seq1 comp55966_c0_seq4 comp57416_c0_seq1 comp55101_c0_seq3 comp48216_c0_seq1 comp55674_c0_seq3 comp55875_c1_seq1 comp50204_c0_seq6 comp28018_c0_seq1 comp43710_c1_seq2 comp57998_c1_seq3 comp49830_c0_seq1 comp51753_c1_seq2 comp45119_c0_seq1 comp53705_c2_seq3 comp56641_c0_seq15 comp57764_c7_seq39 comp48941_c0_seq1 comp52566_c0_seq2 comp56297_c1_seq5 comp44666_c4_seq1 comp31526_c0_seq1 comp56260_c0_seq1 comp57347_c0_seq5 comp43640_c0_seq2 comp54886_c1_seq4 comp45590_c0_seq1 comp54451_c1_seq2 comp52553_c2_seq3 comp55492_c4_seq4 comp29305_c0_seq1 comp55762_c4_seq11 comp56192_c4_seq14 comp48174_c0_seq1 comp52493_c3_seq4 comp56233_c6_seq3 comp54866_c0_seq8 comp58128_c2_seq1 comp51351_c0_seq2 comp48171_c0_seq1 comp57252_c1_seq3 comp54140_c0_seq1 comp50313_c0_seq3 comp51817_c0_seq1 comp50304_c1_seq2 comp58068_c1_seq10 comp49092_c0_seq1 comp54263_c1_seq1 comp53678_c2_seq2 comp56287_c3_seq2 comp53403_c0_seq1 comp53310_c0_seq1 comp53874_c0_seq2 comp55503_c6_seq11 comp53682_c0_seq2 comp52504_c0_seq4 comp52421_c0_seq3 comp57178_c0_seq5 comp58021_c3_seq3 comp57935_c1_seq1 comp54120_c2_seq1 comp57203_c0_seq2 comp44534_c0_seq1 comp50077_c0_seq1 comp51097_c0_seq1 comp58262_c4_seq3 comp56470_c1_seq2 comp54862_c0_seq2 comp53475_c0_seq5 comp57254_c0_seq1 comp32086_c1_seq1 comp51527_c2_seq1 comp52094_c1_seq1 comp36218_c1_seq1 comp52534_c0_seq2 comp56671_c0_seq4 comp57803_c2_seq1 comp46609_c0_seq2 comp56952_c6_seq31 comp54919_c0_seq3 comp42894_c0_seq1 comp52443_c0_seq2 comp33257_c1_seq1 comp55978_c1_seq4 comp57336_c0_seq2 comp58281_c4_seq3 comp57654_c3_seq7 comp53915_c1_seq2 comp55626_c1_seq7 comp57128_c0_seq4 comp50762_c1_seq2 comp55896_c1_seq2 comp53053_c0_seq2 comp51016_c0_seq1 comp32511_c0_seq1 comp57384_c2_seq1 comp54453_c2_seq2 comp57116_c4_seq42 comp52576_c0_seq1 comp53037_c0_seq5 comp28678_c0_seq1 comp57807_c6_seq13 comp50347_c1_seq3 comp57229_c1_seq4 comp56860_c3_seq10 comp58241_c0_seq37 comp28919_c0_seq1 comp47722_c1_seq1 comp52334_c0_seq1 comp31111_c1_seq1 comp56446_c0_seq3 comp56043_c0_seq4 comp56680_c1_seq1 comp50740_c0_seq3 comp2444_c0_seq1 comp55238_c9_seq3 comp57854_c0_seq4 comp56775_c5_seq1 comp37038_c0_seq1 comp53800_c0_seq3 comp41417_c0_seq1 comp54417_c0_seq1 comp52217_c1_seq1 comp51334_c4_seq1 comp59358_c0_seq1 comp56920_c5_seq7 comp42342_c0_seq3 comp44358_c0_seq1 comp57422_c0_seq4 comp56379_c1_seq8 comp46412_c0_seq1 comp56131_c1_seq1 comp48820_c0_seq1 comp55156_c2_seq3 comp50819_c2_seq1 comp51621_c2_seq3 comp55512_c1_seq3 comp56987_c9_seq3 comp47096_c4_seq1 comp55894_c0_seq3 comp49417_c2_seq1 comp57645_c1_seq10 comp51918_c0_seq2 comp45497_c0_seq1 comp43348_c0_seq2 comp53335_c0_seq10 comp58048_c4_seq27 comp54598_c3_seq2 comp51503_c0_seq1 comp54459_c1_seq1 comp55010_c1_seq2 comp52477_c1_seq1 comp44010_c2_seq1 comp55394_c0_seq2 comp56823_c1_seq9 comp48825_c2_seq4 comp58092_c2_seq12 comp56195_c0_seq1 comp33384_c0_seq2 comp46625_c0_seq1 comp57070_c1_seq2 comp58108_c1_seq3 comp56910_c0_seq3 comp30147_c0_seq1 comp53765_c0_seq1 comp49228_c1_seq2 comp56934_c0_seq9 comp53529_c2_seq1 comp58566_c0_seq1 comp58020_c0_seq3 comp56540_c0_seq9 comp52393_c2_seq2 comp57337_c0_seq2 comp58089_c4_seq6 comp58221_c0_seq21 comp50931_c1_seq9 comp47070_c1_seq1 comp50393_c0_seq1 comp49008_c1_seq1 comp56193_c6_seq6 comp55735_c0_seq1 comp51246_c0_seq1 comp46342_c0_seq2 comp54167_c1_seq1 comp51300_c0_seq10 comp56054_c6_seq2 comp56331_c4_seq3 comp56422_c1_seq5 comp56110_c4_seq1 comp54058_c0_seq1 comp59348_c0_seq1 comp33106_c0_seq1 comp52568_c10_seq1 comp56871_c0_seq2 comp53423_c1_seq5 comp36981_c0_seq1 comp54745_c1_seq1 comp50208_c0_seq3 comp52434_c0_seq3 comp57856_c15_seq26 comp53659_c1_seq1 comp50204_c0_seq3 comp29864_c0_seq1 comp53071_c0_seq1 comp51410_c0_seq1 comp55947_c0_seq1 comp48082_c0_seq1 comp52852_c0_seq2 comp46368_c0_seq3 comp45746_c0_seq1 comp55055_c0_seq1 comp50282_c0_seq2 comp54190_c6_seq6 comp46319_c0_seq1 comp58296_c0_seq22 comp51232_c0_seq1 comp55172_c1_seq3 comp47687_c0_seq1 comp52735_c0_seq7 comp57869_c0_seq4 comp57843_c1_seq4 comp47728_c0_seq1 comp53565_c2_seq2 comp56804_c1_seq11 comp57953_c3_seq32 comp57407_c1_seq19 comp48654_c0_seq2 comp48998_c0_seq2 comp56393_c0_seq1 comp57038_c0_seq3 comp50421_c0_seq4 comp51098_c0_seq3 comp58169_c0_seq1 comp57288_c2_seq4 comp56192_c3_seq1 comp55436_c1_seq1 comp47739_c0_seq1 comp44703_c0_seq1 comp54987_c0_seq1 comp57584_c0_seq1 comp44945_c1_seq1 comp55412_c0_seq2 comp32646_c0_seq1 comp52767_c0_seq1 comp48183_c0_seq1 comp56227_c1_seq8 comp50794_c1_seq1 comp57503_c1_seq1 comp56499_c0_seq4 comp52184_c0_seq1 comp54064_c1_seq1 comp52704_c2_seq1 comp56073_c1_seq3 comp57153_c1_seq6 comp55036_c0_seq1 comp48058_c0_seq1 comp48319_c0_seq3 comp55424_c0_seq2 comp56179_c0_seq1 comp50648_c0_seq1 comp55503_c6_seq4 comp41970_c0_seq2 comp55388_c1_seq1 comp54010_c0_seq4 comp52086_c0_seq2 comp57035_c0_seq1 comp57473_c0_seq33 comp57111_c1_seq1 comp51599_c1_seq1 comp55119_c2_seq2 comp32657_c0_seq1 comp51099_c0_seq4 comp54428_c0_seq10 comp57467_c0_seq34 comp54195_c0_seq2 comp56663_c0_seq2 comp48277_c0_seq1 comp32575_c1_seq1 comp58120_c1_seq12 comp33001_c0_seq1 comp51212_c1_seq9 comp56812_c0_seq2 comp49768_c0_seq3 comp55526_c5_seq2 comp2857_c0_seq1 comp53710_c1_seq4 comp8764_c0_seq1 comp33065_c0_seq1 comp57997_c1_seq1 comp53627_c2_seq1 comp56319_c0_seq1 comp56653_c1_seq20 comp58066_c1_seq2 comp53241_c0_seq13 comp50255_c1_seq1 comp58038_c1_seq5 comp55207_c2_seq2 comp57730_c6_seq2 comp56897_c1_seq1 comp53681_c3_seq1 comp55133_c0_seq2 comp57165_c1_seq9 comp57058_c0_seq3 comp53221_c0_seq3 comp50653_c0_seq1 comp29278_c0_seq1 comp50417_c1_seq1 comp51650_c0_seq2 comp38856_c0_seq1 comp47850_c0_seq1 comp54605_c1_seq1 comp54549_c1_seq1 comp57868_c1_seq4 comp46684_c0_seq1 comp50789_c1_seq3 comp41335_c0_seq1 comp56136_c0_seq7 comp52380_c0_seq1 comp57079_c2_seq3 comp54175_c0_seq1 comp57702_c1_seq1 comp51253_c1_seq1 comp56793_c0_seq3 comp57425_c1_seq5 comp57078_c0_seq3 comp58140_c7_seq5 comp53760_c1_seq1 comp58246_c1_seq3 comp57530_c7_seq10 comp48044_c0_seq3 comp39130_c1_seq1 comp57694_c7_seq10 comp53071_c2_seq2 comp54203_c2_seq2 comp49574_c1_seq1 comp57813_c0_seq1 comp53184_c1_seq2 comp37570_c0_seq1 comp45800_c2_seq1 comp57835_c0_seq6 comp50550_c0_seq1 comp57404_c0_seq2 comp50022_c1_seq1 comp55238_c6_seq10 comp57084_c0_seq2 comp28533_c0_seq1 comp43211_c0_seq1 comp55946_c0_seq7 comp52127_c0_seq3 comp57782_c0_seq3 comp57566_c10_seq1 comp56737_c5_seq27 comp51286_c1_seq1 comp50108_c0_seq4 comp56256_c0_seq2 comp56726_c2_seq13 comp29219_c0_seq1 comp57810_c0_seq11 comp54782_c1_seq6 comp56751_c2_seq12 comp53532_c0_seq1 comp52276_c3_seq1 comp117314_c0_seq1 comp29269_c0_seq1 comp57899_c2_seq13 comp56650_c1_seq6 comp53478_c0_seq3 comp53251_c0_seq4 comp32596_c0_seq1 comp30662_c0_seq1 comp44074_c3_seq1 comp56516_c2_seq8 comp55656_c1_seq1 comp32839_c0_seq1 comp55964_c0_seq11 comp53976_c0_seq2 comp34386_c0_seq1 comp51311_c0_seq4 comp50075_c0_seq2 comp46882_c0_seq1 comp53033_c0_seq1 comp55132_c0_seq1 comp56826_c1_seq4 comp57109_c0_seq2 comp42127_c0_seq1 comp45629_c0_seq1 comp56656_c0_seq3 comp55890_c0_seq1 comp44682_c1_seq2 comp55383_c2_seq6 comp52835_c1_seq2 comp51185_c1_seq3 comp55473_c0_seq2 comp57341_c5_seq9 comp54052_c0_seq3 comp46488_c0_seq1 comp57107_c0_seq5 comp46939_c1_seq1 comp56227_c1_seq4 comp53011_c0_seq7 comp55908_c1_seq1 comp51386_c0_seq3 comp52291_c0_seq1 comp41501_c0_seq1 comp57780_c6_seq1 comp55822_c3_seq1 comp56777_c0_seq7 comp53917_c0_seq1 comp51828_c2_seq2 comp46928_c1_seq1 comp51770_c1_seq3 comp54196_c0_seq1 comp57462_c0_seq9 comp50787_c1_seq1 comp50408_c2_seq1 comp52889_c0_seq1 comp51334_c0_seq4 comp55549_c4_seq1 comp52252_c0_seq1 comp28125_c0_seq1 comp54139_c4_seq1 comp48914_c0_seq3 comp57214_c0_seq9 comp58161_c0_seq2 comp57125_c0_seq2 comp57697_c0_seq2 comp47748_c3_seq2 comp30938_c0_seq1 comp55981_c0_seq4 comp53712_c1_seq2 comp54394_c1_seq3 comp45619_c0_seq1 comp58239_c2_seq20 comp52979_c2_seq8 comp43899_c1_seq1 comp51033_c3_seq2 comp49542_c0_seq1 comp57073_c1_seq3 comp43963_c1_seq1 comp30396_c0_seq1 comp49910_c0_seq1 comp57682_c2_seq7 comp57142_c0_seq1 comp56952_c6_seq9 comp53942_c6_seq1 comp55536_c0_seq2 comp51159_c1_seq3 comp55810_c0_seq2 comp57069_c2_seq1 comp29163_c0_seq1 comp43614_c0_seq1 comp57624_c2_seq6 comp32472_c0_seq1 comp53915_c0_seq2 comp56262_c0_seq1 comp56332_c0_seq4 comp58154_c2_seq4 comp54006_c0_seq4 comp56445_c0_seq1 comp57236_c1_seq3 comp57259_c0_seq8 comp58100_c1_seq6 comp53084_c0_seq1 comp53427_c0_seq4 comp57517_c4_seq5 comp48704_c0_seq1 comp50031_c0_seq1 comp33431_c0_seq1 comp57205_c0_seq2 comp53130_c1_seq1 comp56435_c1_seq2 comp104508_c0_seq1 comp51091_c0_seq2 comp43081_c0_seq1 comp54399_c0_seq3 comp53604_c0_seq3 comp58133_c5_seq3 comp54738_c0_seq4 comp54689_c3_seq2 comp48980_c0_seq1 comp48207_c0_seq1 comp49463_c0_seq1 comp50516_c1_seq2 comp54424_c0_seq2 comp57272_c2_seq4 comp58001_c3_seq10 comp46804_c0_seq1 comp56887_c0_seq1 comp48796_c2_seq2 comp43925_c0_seq1 comp54724_c2_seq5 comp48805_c5_seq1 comp54078_c0_seq11 comp52680_c0_seq4 comp42906_c0_seq3 comp47180_c0_seq2 comp52686_c0_seq1 comp31513_c0_seq1 comp57747_c1_seq1 comp54841_c0_seq1 comp52455_c0_seq2 comp51031_c1_seq2 comp49483_c1_seq1 comp56529_c5_seq3 comp49155_c1_seq1 comp57859_c0_seq1 comp57645_c3_seq25 comp44123_c2_seq3 comp54303_c0_seq8 comp57041_c0_seq2 comp48929_c1_seq1 comp56388_c2_seq2 comp51207_c1_seq1 comp45261_c1_seq1 comp57671_c4_seq10 comp37759_c0_seq1 comp42589_c1_seq1 comp46068_c0_seq2 comp57755_c3_seq1 comp57390_c1_seq6 comp57232_c1_seq2 comp52261_c0_seq2 comp44676_c0_seq1 comp53575_c0_seq2 comp56260_c0_seq5 comp43455_c1_seq1 comp49692_c0_seq1 comp54033_c2_seq1 comp57638_c3_seq3 comp52300_c0_seq2 comp58274_c2_seq7 comp56590_c1_seq2 comp54378_c2_seq1 comp50487_c0_seq1 comp55998_c5_seq5 comp55184_c10_seq2 comp56184_c2_seq6 comp50862_c1_seq1 comp57680_c0_seq12 comp52994_c0_seq1 comp28884_c0_seq1 comp51212_c1_seq11 comp30957_c0_seq1 comp45953_c1_seq1 comp53229_c0_seq2 comp57230_c0_seq1 comp57115_c0_seq3 comp76337_c0_seq1 comp54555_c0_seq3 comp45803_c0_seq1 comp58233_c8_seq11 comp56818_c0_seq2 comp52989_c3_seq1 comp56107_c0_seq5 comp57499_c6_seq22 comp57508_c0_seq3 comp56854_c1_seq3 comp57145_c3_seq17 comp53779_c2_seq5 comp57523_c0_seq2 comp58263_c1_seq20 comp31726_c0_seq1 comp44383_c0_seq1 comp57864_c1_seq1 comp55084_c0_seq15 comp49654_c0_seq1 comp49014_c0_seq1 comp49870_c0_seq4 comp46501_c0_seq1 comp43727_c1_seq1 comp52011_c0_seq1 comp57554_c1_seq4 comp56424_c0_seq2 comp36832_c1_seq1 comp57791_c0_seq4 comp57188_c1_seq2 comp29156_c0_seq1 comp48629_c1_seq1 comp48411_c2_seq1 comp55641_c0_seq6 comp57756_c2_seq1 comp55699_c0_seq4 comp55243_c0_seq4 comp51052_c0_seq1 comp53397_c0_seq4 comp57086_c1_seq6 comp49678_c0_seq1 comp53527_c2_seq3 comp54700_c3_seq3 comp50517_c1_seq1 comp53605_c0_seq4 comp51329_c2_seq1 comp45194_c1_seq1 comp56895_c1_seq3 comp53688_c0_seq5 comp54362_c0_seq1 comp57539_c1_seq13 comp49072_c0_seq2 comp54443_c0_seq1 comp55592_c0_seq2 comp47406_c0_seq1 comp57907_c11_seq8 comp49504_c0_seq3 comp48865_c0_seq2 comp52012_c5_seq7 comp55358_c0_seq2 comp57739_c5_seq1 comp58074_c5_seq45 comp50404_c0_seq1 comp54545_c0_seq1 comp50131_c1_seq1 comp44459_c0_seq2 comp50094_c0_seq1 comp48220_c1_seq1 comp185550_c0_seq1 comp56665_c2_seq2 comp49535_c1_seq1 comp51057_c0_seq2 comp53495_c0_seq1 comp56091_c0_seq1 comp47504_c0_seq1 comp56145_c10_seq6 comp57823_c1_seq3 comp57776_c3_seq10 comp56941_c0_seq4 comp58223_c6_seq38 comp58296_c0_seq23 comp31856_c0_seq2 comp49078_c1_seq4 comp50563_c0_seq2 comp57963_c2_seq5 comp46014_c0_seq1 comp51611_c1_seq5 comp56555_c0_seq4 comp55217_c0_seq1 comp43498_c0_seq1 comp46177_c0_seq1 comp57984_c1_seq1 comp57513_c1_seq1 comp52081_c0_seq6 comp49151_c0_seq3 comp42759_c0_seq1 comp35114_c0_seq1 comp49725_c0_seq1 comp58565_c0_seq1 comp46224_c0_seq1 comp52716_c0_seq9 comp29429_c0_seq1 comp56309_c1_seq2 comp55982_c0_seq3 comp48621_c0_seq1 comp56889_c0_seq6 comp414890_c0_seq1 comp54619_c1_seq1 comp54234_c0_seq1 comp57394_c1_seq10 comp57210_c2_seq9 comp56898_c0_seq5 comp48783_c0_seq2 comp31291_c0_seq1 comp58274_c2_seq17 comp46761_c0_seq1 comp56448_c0_seq3 comp56481_c2_seq2 comp51127_c0_seq2 comp46001_c0_seq1 comp46919_c1_seq1 comp54326_c0_seq2 comp29024_c0_seq1 comp57905_c1_seq10 comp55938_c0_seq3 comp36675_c0_seq1 comp56374_c0_seq2 comp49106_c0_seq3 comp55892_c0_seq11 comp53107_c0_seq1 comp53645_c0_seq1 comp57402_c3_seq6 comp53873_c0_seq1 comp48767_c0_seq1 comp58042_c1_seq2 comp58108_c1_seq8 comp57324_c1_seq18 comp53077_c0_seq1 comp56387_c1_seq1 comp57389_c0_seq9 comp48122_c0_seq2 comp57564_c2_seq65 comp30526_c0_seq1 comp48671_c0_seq1 comp58158_c2_seq8 comp48779_c4_seq1 comp56668_c0_seq10 comp46887_c0_seq2 comp51459_c1_seq2 comp55083_c0_seq11 comp58131_c2_seq8 comp49728_c0_seq2 comp54337_c0_seq1 comp54512_c0_seq2 comp57445_c0_seq13 comp54449_c1_seq1 comp57082_c0_seq11 comp53482_c0_seq1 comp46893_c0_seq1 comp54659_c0_seq2 comp57587_c2_seq13 comp54382_c0_seq5 comp43339_c0_seq1 comp58011_c1_seq14 comp51089_c1_seq1 comp56764_c1_seq4 comp41494_c0_seq2 comp57023_c1_seq17 comp46762_c0_seq1 comp57566_c15_seq12 comp54020_c0_seq1 comp57241_c2_seq4 comp55238_c6_seq5 comp48430_c0_seq2 comp52329_c1_seq1 comp54438_c1_seq2 comp58087_c4_seq4 comp54309_c0_seq4 comp57431_c4_seq3 comp55598_c1_seq1 comp59550_c0_seq1 comp56959_c0_seq1 comp55439_c0_seq1 comp53950_c0_seq3 comp28546_c0_seq1 comp56096_c4_seq19 comp54333_c0_seq1 comp52778_c0_seq4 comp46728_c2_seq3 comp44666_c3_seq1 comp55678_c0_seq1 comp33227_c0_seq1 comp58224_c0_seq22 comp52708_c0_seq2 comp29420_c0_seq1 comp57293_c0_seq2 comp32413_c0_seq1 comp53074_c0_seq2 comp46304_c0_seq1 comp57568_c3_seq18 comp58354_c0_seq1 comp58268_c2_seq2 comp58185_c2_seq1 comp57639_c1_seq3 comp55196_c0_seq4 comp51217_c0_seq1 comp49233_c0_seq3 comp55157_c0_seq1 comp56237_c0_seq2 comp51943_c0_seq1 comp58237_c0_seq2 comp57109_c5_seq6 comp53572_c1_seq3 comp55321_c0_seq3 comp57038_c0_seq7 comp47570_c1_seq1 comp56277_c0_seq4 comp43331_c0_seq1 comp56628_c1_seq4 comp55167_c0_seq5 comp49671_c0_seq1 comp37829_c0_seq1 comp56188_c1_seq2 comp56940_c0_seq1 comp54724_c2_seq2 comp55084_c0_seq7 comp29518_c0_seq1 comp53499_c2_seq5 comp58278_c0_seq11 comp30953_c0_seq1 comp50007_c0_seq2 comp46231_c0_seq3 comp48624_c0_seq1 comp53091_c0_seq2 comp50146_c0_seq2 comp54427_c1_seq5 comp52411_c2_seq3 comp49905_c1_seq1 comp55230_c1_seq6 comp51962_c1_seq1 comp41963_c3_seq1 comp49036_c0_seq2 comp54830_c2_seq2 comp56709_c1_seq1 comp55299_c0_seq1 comp56804_c1_seq22 comp30308_c0_seq1 comp50144_c0_seq2 comp57239_c2_seq15 comp57031_c1_seq11 comp56634_c0_seq1 comp57643_c9_seq3 comp31639_c0_seq1 comp56099_c0_seq19 comp28791_c0_seq1 comp57525_c0_seq2 comp58023_c1_seq3 comp53403_c0_seq4 comp56889_c2_seq4 comp50746_c0_seq2 comp44473_c1_seq1 comp54586_c3_seq5 comp57857_c1_seq27 comp54501_c0_seq1 comp57172_c1_seq14 comp56588_c1_seq4 comp30366_c0_seq1 comp56784_c2_seq2 comp41824_c0_seq1 comp58441_c0_seq1 comp55614_c0_seq1 comp47824_c0_seq1 comp29552_c1_seq1 comp54213_c0_seq6 comp56951_c0_seq3 comp51455_c0_seq1 comp40420_c0_seq1 comp58065_c1_seq3 comp58057_c2_seq1 comp48875_c0_seq1 comp57698_c1_seq1 comp52951_c0_seq3 comp56762_c0_seq1 comp56436_c0_seq1 comp41753_c0_seq1 comp56748_c2_seq2 comp58216_c0_seq15 comp29577_c0_seq1 comp56022_c3_seq4 comp58273_c0_seq2 comp56391_c3_seq10 comp54385_c1_seq2 comp55400_c0_seq1 comp56378_c4_seq1 comp51970_c2_seq1 comp56359_c0_seq4 comp50831_c0_seq5 comp52919_c0_seq6 comp52473_c0_seq1 comp50429_c0_seq1 comp50560_c0_seq1 comp58255_c1_seq4 comp57254_c9_seq16 comp55385_c0_seq4 comp46035_c0_seq1 comp56192_c4_seq11 comp50744_c0_seq4 comp58171_c0_seq6 comp45893_c0_seq2 comp56558_c0_seq9 comp40518_c0_seq1 comp58016_c3_seq1 comp53190_c0_seq1 comp54018_c0_seq2 comp53680_c0_seq5 comp41176_c0_seq2 comp57743_c3_seq44 comp58240_c1_seq2 comp48949_c0_seq1 comp58207_c0_seq5 comp51722_c2_seq1 comp46531_c1_seq4 comp29851_c0_seq1 comp55585_c3_seq1 comp53941_c4_seq2 comp57680_c0_seq1 comp56690_c0_seq3 comp45972_c0_seq2 comp57956_c0_seq3 comp54494_c2_seq3 comp53517_c0_seq5 comp57827_c1_seq1 comp30016_c0_seq1 comp49661_c0_seq2 comp46173_c0_seq4 comp39263_c1_seq3 comp55477_c1_seq6 comp56376_c0_seq1 comp56777_c2_seq9 comp55703_c2_seq7 comp51242_c1_seq2 comp57433_c0_seq5 comp57306_c3_seq1 comp57588_c11_seq7 comp46164_c0_seq1 comp57876_c0_seq4 comp57842_c6_seq13 comp55194_c0_seq3 comp50815_c1_seq2 comp53543_c1_seq1 comp57011_c3_seq1 comp45022_c0_seq1 comp54505_c0_seq1 comp41515_c0_seq2 comp48424_c1_seq2 comp41420_c0_seq1 comp54444_c1_seq2 comp59285_c0_seq1 comp57153_c1_seq23 comp47026_c0_seq1 comp50610_c0_seq2 comp46702_c0_seq1 comp53539_c1_seq2 comp51434_c0_seq2 comp58304_c1_seq3 comp56954_c2_seq5 comp51392_c0_seq1 comp54558_c1_seq1 comp56536_c0_seq9 comp55111_c0_seq5 comp43741_c0_seq1 comp56515_c3_seq1 comp56212_c0_seq3 comp57717_c0_seq6 comp55774_c3_seq1 comp51541_c2_seq11 comp52925_c0_seq2 comp57926_c8_seq14 comp56835_c2_seq1 comp58063_c0_seq30 comp58245_c1_seq35 comp54966_c1_seq5 comp56297_c1_seq2 comp53130_c2_seq1 comp54917_c0_seq3 comp57614_c0_seq12 comp45052_c0_seq8 comp55199_c1_seq2 comp52259_c0_seq1 comp57588_c4_seq4 comp55585_c0_seq1 comp55156_c1_seq1 comp52906_c2_seq4 comp42968_c0_seq1 comp51971_c2_seq1 comp56529_c7_seq4 comp51290_c0_seq1 comp35416_c1_seq1 comp55725_c1_seq1 comp54998_c0_seq6 comp28446_c0_seq1 comp47397_c0_seq2 comp53624_c1_seq1 comp57214_c0_seq4 comp57672_c5_seq1 comp56803_c1_seq1 comp17615_c0_seq1 comp31908_c0_seq1 comp57850_c0_seq4 comp52551_c0_seq2 comp55162_c0_seq4 comp58039_c0_seq1 comp54139_c2_seq7 comp51817_c1_seq1 comp57989_c0_seq11 comp34540_c0_seq2 comp52153_c1_seq1 comp53012_c0_seq1 comp55776_c2_seq2 comp49753_c0_seq2 comp48226_c1_seq1 comp54088_c2_seq1 comp28537_c0_seq1 comp48017_c0_seq1 comp52962_c0_seq1 comp55361_c0_seq1 comp54939_c0_seq2 comp108869_c0_seq1 comp49941_c1_seq1 comp46252_c0_seq1 comp46006_c1_seq1 comp45881_c1_seq1 comp49144_c0_seq1 comp46338_c0_seq2 comp57838_c0_seq13 comp58235_c0_seq3 comp56111_c1_seq4 comp57807_c6_seq14 comp57680_c0_seq15 comp44084_c0_seq1 comp54000_c0_seq8 comp57297_c1_seq3 comp55644_c0_seq5 comp52830_c0_seq1 comp46132_c0_seq3 comp57612_c0_seq5 comp49677_c0_seq1 comp58062_c4_seq1 comp57987_c9_seq13 comp55367_c0_seq6 comp56923_c0_seq5 comp53951_c0_seq3 comp56211_c0_seq1 comp50711_c0_seq4 comp54867_c1_seq5 comp51196_c0_seq1 comp55643_c0_seq4 comp32301_c0_seq1 comp57308_c1_seq3 comp56481_c2_seq11 comp53818_c1_seq2 comp55547_c0_seq2 comp49605_c0_seq1 comp56192_c4_seq15 comp43994_c1_seq1 comp55830_c4_seq1 comp31307_c0_seq1 comp57020_c0_seq4 comp43280_c0_seq2 comp58040_c4_seq5 comp55136_c0_seq3 comp57380_c1_seq3 comp56698_c1_seq1 comp57861_c0_seq6 comp51689_c0_seq1 comp48609_c0_seq1 comp58167_c0_seq2 comp51789_c0_seq1 comp53753_c1_seq2 comp56716_c2_seq9 comp54405_c0_seq8 comp45244_c4_seq2 comp51828_c2_seq3 comp29465_c0_seq1 comp58206_c6_seq7 comp56247_c0_seq2 comp45787_c0_seq2 comp56145_c10_seq2 comp60827_c0_seq1 comp51715_c1_seq2 comp39865_c0_seq1 comp49972_c0_seq1 comp58920_c0_seq1 comp57390_c1_seq2 comp54901_c0_seq1 comp53557_c0_seq1 comp47094_c0_seq1 comp55403_c1_seq13 comp50528_c0_seq1 comp48780_c0_seq2 comp58196_c0_seq3 comp58240_c1_seq17 comp55057_c0_seq7 comp57385_c2_seq4 comp54683_c0_seq1 comp48626_c1_seq1 comp28300_c0_seq1 comp48618_c1_seq1 comp57346_c3_seq10 comp46544_c0_seq4 comp51085_c0_seq1 comp57388_c2_seq12 comp51593_c0_seq1 comp55327_c2_seq2 comp49368_c0_seq1 comp58182_c2_seq12 comp57696_c2_seq1 comp45767_c1_seq1 comp47399_c0_seq1 comp55950_c0_seq4 comp45699_c1_seq2 comp53381_c1_seq3 comp30141_c0_seq1 comp49885_c2_seq1 comp56132_c1_seq1 comp57667_c2_seq5 comp51822_c0_seq1 comp57387_c0_seq7 comp57638_c2_seq1 comp51065_c0_seq1 comp49127_c1_seq1 comp56254_c0_seq3 comp53100_c1_seq3 comp55589_c4_seq2 comp49684_c0_seq9 comp53629_c0_seq6 comp57438_c3_seq5 comp57892_c1_seq1 comp50586_c0_seq3 comp28745_c0_seq1 comp51808_c0_seq4 comp57789_c0_seq7 comp55972_c2_seq18 comp58092_c1_seq1 comp53875_c0_seq11 comp56044_c0_seq4 comp55130_c1_seq4 comp46205_c0_seq4 comp55767_c0_seq5 comp50652_c1_seq1 comp54520_c1_seq1 comp55685_c1_seq5 comp56428_c4_seq4 comp48698_c3_seq2 comp53125_c0_seq7 comp53562_c2_seq12 comp57330_c2_seq21 comp55309_c0_seq4 comp45856_c0_seq1 comp45459_c1_seq1 comp58042_c1_seq15 comp56081_c1_seq2 comp55171_c1_seq1 comp53964_c0_seq1 comp54351_c1_seq3 comp42926_c0_seq2 comp56856_c2_seq7 comp57610_c5_seq7 comp52262_c0_seq3 comp57481_c0_seq11 comp52263_c0_seq14 comp57495_c4_seq1 comp55211_c0_seq3 comp56931_c0_seq1 comp48622_c0_seq1 comp54350_c0_seq1 comp49619_c0_seq2 comp39150_c1_seq1 comp56116_c0_seq3 comp51763_c1_seq2 comp53324_c0_seq2 comp34992_c0_seq1 comp54195_c0_seq3 comp48726_c0_seq3 comp52397_c2_seq1 comp48921_c0_seq1 comp50789_c0_seq1 comp51893_c1_seq1 comp48369_c0_seq1 comp55291_c0_seq1 comp58156_c0_seq3 comp55005_c1_seq2 comp51372_c0_seq1 comp44701_c0_seq1 comp55736_c1_seq8 comp52587_c0_seq3 comp53946_c0_seq1 comp55329_c0_seq4 comp56742_c0_seq2 comp45834_c1_seq1 comp59433_c0_seq1 comp55771_c0_seq4 comp55788_c1_seq5 comp49466_c0_seq1 comp53145_c3_seq1 comp39374_c1_seq1 comp49629_c0_seq3 comp53326_c0_seq5 comp51152_c0_seq1 comp56672_c1_seq26 comp53625_c0_seq3 comp58102_c11_seq10 comp55389_c1_seq4 comp57986_c2_seq3 comp56810_c4_seq4 comp55652_c0_seq2 comp55063_c6_seq7 comp57643_c3_seq3 comp57446_c0_seq1 comp38415_c1_seq1 comp50609_c3_seq1 comp251406_c0_seq1 comp51207_c0_seq4 comp30848_c0_seq2 comp52592_c1_seq1 comp45828_c0_seq1 comp51921_c0_seq2 comp46248_c0_seq2 comp51362_c0_seq1 comp33341_c0_seq1 comp54584_c0_seq1 comp52512_c3_seq3 comp43175_c1_seq1 comp57273_c1_seq3 comp55585_c1_seq1 comp52323_c1_seq1 comp54694_c1_seq3 comp57301_c1_seq1 comp49566_c0_seq2 comp54848_c0_seq4 comp54903_c1_seq3 comp57750_c1_seq6 comp57802_c0_seq1 comp55072_c0_seq4 comp55900_c8_seq18 comp56458_c0_seq1 comp29300_c0_seq1 comp48907_c0_seq1 comp52488_c0_seq4 comp56293_c0_seq5 comp57122_c0_seq1 comp42160_c0_seq1 comp52111_c2_seq1 comp57355_c1_seq6 comp55104_c1_seq3 comp27881_c0_seq1 comp56920_c5_seq5 comp48995_c2_seq1 comp57351_c0_seq1 comp41468_c0_seq1 comp56493_c1_seq2 comp53893_c0_seq4 comp61252_c0_seq1 comp50054_c0_seq4 comp47805_c0_seq1 comp46292_c0_seq1 comp45945_c0_seq1 comp46114_c1_seq1 comp58076_c4_seq3 comp58258_c0_seq12 comp56020_c2_seq6 comp54597_c0_seq2 comp52665_c0_seq1 comp32259_c0_seq1 comp104147_c0_seq1 comp53101_c0_seq2 comp51726_c0_seq1 comp52309_c2_seq1 comp51385_c0_seq1 comp56182_c0_seq7 comp30536_c0_seq1 comp57317_c3_seq11 comp45518_c0_seq1 comp53406_c1_seq2 comp29108_c0_seq1 comp53777_c0_seq3 comp54458_c2_seq2 comp55783_c0_seq4 comp47892_c0_seq1 comp57003_c0_seq1 comp70776_c0_seq1 comp41259_c0_seq3 comp43258_c0_seq1 comp51045_c0_seq2 comp56388_c1_seq9 comp51588_c0_seq1 comp57837_c1_seq10 comp49155_c0_seq1 comp54925_c0_seq4 comp45089_c0_seq1 comp54870_c0_seq4 comp30977_c0_seq1 comp47272_c1_seq3 comp58137_c2_seq22 comp56718_c1_seq1 comp44697_c0_seq1 comp57206_c0_seq2 comp57334_c1_seq1 comp57989_c0_seq1 comp56413_c0_seq1 comp56622_c2_seq3 comp56005_c0_seq6 comp51853_c0_seq1 comp44080_c0_seq1 comp55689_c1_seq16 comp48669_c0_seq1 comp56131_c0_seq1 comp56942_c0_seq1 comp56527_c0_seq5 comp32636_c0_seq1 comp54887_c6_seq2 comp54039_c0_seq4 comp56970_c0_seq4 comp49357_c0_seq4 comp57430_c1_seq17 comp50931_c1_seq8 comp54104_c0_seq4 comp57274_c4_seq6 comp52476_c0_seq4 comp43870_c0_seq2 comp49847_c0_seq1 comp51852_c1_seq2 comp50005_c0_seq1 comp56152_c0_seq8 comp53854_c4_seq5 comp55631_c0_seq15 comp54909_c1_seq4 comp57589_c0_seq1 comp55772_c2_seq1 comp42555_c0_seq2 comp53232_c0_seq5 comp58250_c1_seq11 comp58098_c2_seq21 comp45451_c0_seq1 comp56211_c1_seq4 comp50232_c0_seq1 comp43742_c0_seq1 comp54197_c0_seq1 comp35817_c0_seq2 comp57807_c7_seq7 comp45868_c1_seq1 comp56856_c2_seq2 comp55268_c1_seq2 comp46841_c2_seq3 comp54391_c4_seq1 comp53038_c0_seq1 comp56116_c0_seq2 comp54475_c0_seq4 comp55670_c5_seq2 comp55385_c0_seq1 comp56563_c3_seq1 comp43844_c0_seq1 comp31415_c0_seq1 comp40095_c0_seq1 comp49374_c0_seq1 comp47277_c0_seq2 comp49979_c2_seq1 comp52891_c2_seq1 comp58134_c0_seq5 comp56151_c0_seq10 comp54091_c1_seq2 comp54288_c4_seq1 comp32289_c0_seq1 comp54065_c0_seq1 comp58178_c0_seq4 comp53945_c0_seq1 comp46295_c0_seq1 comp52735_c0_seq2 comp49257_c0_seq1 comp56583_c2_seq2 comp55184_c10_seq1 comp58010_c0_seq23 comp57946_c6_seq11 comp51805_c0_seq2 comp30358_c0_seq1 comp48832_c0_seq5 comp51854_c0_seq1 comp56597_c0_seq2 comp56296_c0_seq1 comp53867_c0_seq1 comp54053_c0_seq2 comp56641_c0_seq12 comp57422_c0_seq5 comp53426_c0_seq1 comp51269_c0_seq1 comp56789_c0_seq1 comp50896_c0_seq1 comp54354_c0_seq2 comp30146_c0_seq1 comp56660_c1_seq3 comp49978_c0_seq1 comp52582_c6_seq4 comp57956_c0_seq9 comp48192_c0_seq1 comp47090_c1_seq1 comp246704_c0_seq1 comp54616_c0_seq3 comp55805_c2_seq1 comp49531_c0_seq1 comp57725_c0_seq10 comp57063_c1_seq1 comp36409_c0_seq1 comp57431_c6_seq1 comp58000_c0_seq2 comp48334_c1_seq1 comp45813_c1_seq1 comp56013_c1_seq3 comp45379_c1_seq1 comp56054_c6_seq7 comp51241_c0_seq1 comp56947_c0_seq1 comp38496_c0_seq1 comp54267_c0_seq1 comp47687_c0_seq2 comp55475_c0_seq3 comp57213_c2_seq7 comp54321_c0_seq3 comp52977_c0_seq6 comp52348_c0_seq2 comp32381_c0_seq1 comp49576_c0_seq1 comp56341_c3_seq5 comp56961_c3_seq36 comp30272_c0_seq1 comp55373_c0_seq3 comp52425_c0_seq3 comp49146_c0_seq1 comp51611_c1_seq4 comp51506_c0_seq1 comp45418_c1_seq1 comp45002_c0_seq1 comp56454_c0_seq3 comp57492_c3_seq2 comp49354_c0_seq1 comp48611_c0_seq4 comp55239_c0_seq3 comp58123_c0_seq24 comp57752_c0_seq6 comp57511_c0_seq3 comp55964_c0_seq2 comp56223_c2_seq1 comp41903_c3_seq2 comp48571_c0_seq1 comp33436_c0_seq1 comp49514_c0_seq5 comp32899_c0_seq1 comp44463_c0_seq1 comp57530_c1_seq3 comp52401_c0_seq1 comp54606_c0_seq5 comp58127_c0_seq7 comp57894_c0_seq2 comp56679_c2_seq2 comp51001_c0_seq1 comp39681_c1_seq1 comp54358_c2_seq2 comp50406_c0_seq3 comp31171_c0_seq1 comp53898_c0_seq2 comp54002_c0_seq2 comp57518_c10_seq9 comp38478_c0_seq1 comp31027_c0_seq1 comp48269_c0_seq3 comp54359_c2_seq16 comp54711_c1_seq4 comp56584_c3_seq4 comp54697_c3_seq2 comp51093_c0_seq2 comp79641_c0_seq1 comp51026_c0_seq3 comp29227_c0_seq1 comp57603_c3_seq5 comp53885_c2_seq2 comp57256_c0_seq5 comp57844_c3_seq12 comp56700_c10_seq1 comp55228_c0_seq2 comp57499_c6_seq2 comp56199_c0_seq11 comp48880_c1_seq1 comp52482_c0_seq1 comp57424_c0_seq5 comp56933_c0_seq5 comp56518_c2_seq2 comp51283_c0_seq2 comp49311_c0_seq2 comp49709_c0_seq1 comp53304_c0_seq3 comp54139_c5_seq1 comp55973_c1_seq2 comp55478_c2_seq4 comp56082_c0_seq2 comp52897_c1_seq3 comp44642_c0_seq3 comp55636_c0_seq3 comp49869_c0_seq2 comp55267_c0_seq2 comp54391_c1_seq4 comp240278_c0_seq1 comp55320_c2_seq1 comp50156_c0_seq1 comp57506_c0_seq5 comp54736_c0_seq4 comp54238_c0_seq1 comp56338_c2_seq3 comp56001_c0_seq5 comp49006_c0_seq4 comp57432_c0_seq2 comp146923_c0_seq1 comp50437_c0_seq2 comp42791_c0_seq1 comp28363_c0_seq1 comp55344_c1_seq5 comp56488_c0_seq2 comp51500_c0_seq1 comp49594_c1_seq1 comp57930_c0_seq3 comp29147_c0_seq1 comp52305_c0_seq1 comp47742_c5_seq1 comp54521_c0_seq2 comp50441_c2_seq2 comp58281_c4_seq4 comp56357_c4_seq13 comp56357_c4_seq8 comp39323_c0_seq1 comp58118_c1_seq4 comp52823_c0_seq1 comp44893_c1_seq1 comp49065_c0_seq2 comp57708_c2_seq7 comp51407_c2_seq1 comp28823_c1_seq1 comp43700_c2_seq1 comp57605_c2_seq9 comp50886_c1_seq3 comp55555_c6_seq3 comp55590_c1_seq1 comp32613_c0_seq2 comp56919_c0_seq2 comp45446_c0_seq1 comp50747_c1_seq2 comp44826_c1_seq1 comp54936_c1_seq1 comp55324_c0_seq4 comp32626_c0_seq2 comp58009_c2_seq10 comp57654_c3_seq4 comp57810_c0_seq1 comp50932_c2_seq2 comp56395_c12_seq5 comp55167_c0_seq2 comp47255_c0_seq2 comp49040_c0_seq1 comp31344_c0_seq1 comp57946_c0_seq1 comp56706_c1_seq2 comp57659_c1_seq9 comp49848_c0_seq1 comp57925_c1_seq1 comp48027_c0_seq1 comp54593_c0_seq5 comp57278_c4_seq6 comp41630_c1_seq1 comp54367_c1_seq2 comp55878_c2_seq5 comp54982_c1_seq2 comp55714_c0_seq1 comp57462_c0_seq11 comp58023_c1_seq8 comp56725_c2_seq3 comp55256_c4_seq6 comp52888_c2_seq1 comp56706_c1_seq1 comp50118_c0_seq1 comp48626_c0_seq1 comp52080_c1_seq3 comp36924_c0_seq1 comp28514_c0_seq1 comp51797_c0_seq1 comp50041_c1_seq13 comp35456_c2_seq1 comp52589_c0_seq2 comp40399_c0_seq1 comp56054_c2_seq1 comp57793_c0_seq10 comp54127_c0_seq1 comp45066_c0_seq1 comp52681_c0_seq1 comp61202_c0_seq1 comp135095_c0_seq1 comp49608_c1_seq4 comp53713_c0_seq2 comp49943_c0_seq3 comp31561_c0_seq1 comp48625_c1_seq1 comp47398_c3_seq1 comp49603_c3_seq3 comp53281_c0_seq2 comp44170_c1_seq1 comp48622_c0_seq2 comp40893_c0_seq1 comp53689_c0_seq2 comp57839_c7_seq6 comp57502_c3_seq1 comp58237_c1_seq1 comp54735_c0_seq4 comp49607_c1_seq1 comp51729_c0_seq1 comp58286_c1_seq28 comp46171_c0_seq2 comp47955_c2_seq1 comp52055_c0_seq1 comp56354_c1_seq8 comp58006_c3_seq1 comp46074_c0_seq1 comp53792_c2_seq1 comp57723_c1_seq1 comp54993_c0_seq6 comp57768_c0_seq3 comp53939_c1_seq2 comp55413_c1_seq2 comp50939_c0_seq4 comp55389_c1_seq7 comp52054_c0_seq1 comp57199_c0_seq3 comp54365_c1_seq2 comp44033_c0_seq1 comp50040_c0_seq7 comp51264_c0_seq2 comp49073_c0_seq1 comp51280_c0_seq1 comp56357_c4_seq21 comp55022_c0_seq4 comp57813_c1_seq1 comp52408_c2_seq1 comp49747_c0_seq2 comp57664_c3_seq9 comp48105_c0_seq1 comp31777_c0_seq2 comp55111_c0_seq2 comp56060_c1_seq6 comp46276_c0_seq1 comp42555_c1_seq1 comp51329_c3_seq1 comp52321_c0_seq3 comp53431_c2_seq14 comp58228_c0_seq1 comp42060_c1_seq1 comp56996_c0_seq1 comp53461_c0_seq1 comp50819_c4_seq1 comp57489_c0_seq21 comp53304_c0_seq4 comp53888_c0_seq2 comp56993_c0_seq10 comp53544_c0_seq1 comp56559_c0_seq11 comp53275_c1_seq2 comp54359_c2_seq17 comp52439_c0_seq3 comp57658_c2_seq1 comp57806_c7_seq10 comp29716_c0_seq1 comp57370_c3_seq6 comp54134_c0_seq1 comp50669_c0_seq9 comp54158_c0_seq1 comp54475_c0_seq3 comp48116_c0_seq1 comp56867_c5_seq2 comp47702_c0_seq1 comp49929_c0_seq1 comp58221_c0_seq32 comp55172_c3_seq1 comp57803_c0_seq13 comp54799_c1_seq4 comp48872_c1_seq2 comp49659_c0_seq7 comp46603_c0_seq3 comp56699_c0_seq4 comp48776_c2_seq1 comp58063_c0_seq22 comp53745_c0_seq2 comp45821_c0_seq2 comp53554_c3_seq2 comp49294_c1_seq2 comp53057_c1_seq2 comp58017_c1_seq11 comp55503_c6_seq14 comp57716_c2_seq15 comp52772_c0_seq2 comp61270_c0_seq1 comp57659_c5_seq11 comp52317_c0_seq1 comp47333_c1_seq1 comp51990_c2_seq18 comp47457_c0_seq1 comp56735_c0_seq14 comp49619_c2_seq2 comp45474_c0_seq1 comp50973_c0_seq1 comp53385_c0_seq1 comp57423_c0_seq3 comp47514_c1_seq5 comp32611_c0_seq1 comp58122_c0_seq11 comp57949_c0_seq2 comp50850_c0_seq1 comp57296_c0_seq3 comp50758_c4_seq1 comp58140_c6_seq7 comp50401_c1_seq4 comp54255_c1_seq2 comp54714_c3_seq1 comp53336_c2_seq1 comp56074_c0_seq10 comp54665_c0_seq2 comp56199_c0_seq8 comp52260_c2_seq1 comp47683_c0_seq1 comp31403_c0_seq2 comp58010_c0_seq17 comp49973_c0_seq7 comp60735_c0_seq1 comp57042_c0_seq2 comp55868_c0_seq2 comp53917_c1_seq4 comp46512_c0_seq1 comp56979_c5_seq6 comp54591_c0_seq1 comp55735_c0_seq9 comp57051_c6_seq7 comp52152_c0_seq1 comp32286_c0_seq2 comp58177_c8_seq1 comp47499_c1_seq1 comp45764_c1_seq3 comp57259_c0_seq6 comp56584_c3_seq7 comp58074_c1_seq1 comp54421_c0_seq1 comp49632_c0_seq1 comp53032_c0_seq2 comp47398_c0_seq1 comp56625_c1_seq10 comp57818_c5_seq57 comp54960_c2_seq9 comp56562_c2_seq3 comp51545_c0_seq3 comp50866_c0_seq1 comp57007_c3_seq1 comp58042_c1_seq7 comp56430_c1_seq2 comp56149_c0_seq1 comp46000_c0_seq1 comp51874_c0_seq1 comp53137_c1_seq2 comp55755_c0_seq4 comp57680_c1_seq1 comp29993_c0_seq1 comp57927_c2_seq1 comp57285_c0_seq10 comp57696_c3_seq30 comp55732_c1_seq2 comp52500_c2_seq1 comp57316_c1_seq4 comp50783_c0_seq1 comp49168_c0_seq2 comp48504_c0_seq1 comp57257_c2_seq8 comp47926_c1_seq1 comp48338_c1_seq1 comp57563_c1_seq4 comp53204_c0_seq2 comp54387_c1_seq5 comp57494_c7_seq2 comp34868_c0_seq1 comp57382_c2_seq4 comp35351_c1_seq1 comp43035_c0_seq1 comp52464_c0_seq3 comp57241_c2_seq17 comp50353_c3_seq1 comp56703_c3_seq4 comp56054_c6_seq5 comp47299_c1_seq1 comp56449_c4_seq1 comp53768_c3_seq1 comp58275_c4_seq24 comp55080_c2_seq1 comp57848_c0_seq21 comp55882_c5_seq16 comp42162_c0_seq1 comp57807_c7_seq1 comp57386_c2_seq1 comp56809_c4_seq11 comp46770_c0_seq2 comp46612_c0_seq1 comp53552_c0_seq3 comp50721_c0_seq1 comp54389_c0_seq1 comp67449_c0_seq1 comp57014_c0_seq2 comp55538_c1_seq5 comp57111_c1_seq3 comp46148_c0_seq2 comp57612_c0_seq25 comp49442_c0_seq2 comp52529_c0_seq2 comp31107_c0_seq1 comp56110_c4_seq4 comp54178_c0_seq1 comp54947_c2_seq3 comp57999_c1_seq2 comp53220_c0_seq6 comp53047_c1_seq3 comp55083_c0_seq6 comp52211_c0_seq2 comp53538_c0_seq1 comp57589_c0_seq5 comp57713_c0_seq4 comp51311_c0_seq1 comp53579_c1_seq1 comp55527_c0_seq4 comp55359_c0_seq1 comp54218_c1_seq4 comp51220_c0_seq3 comp55531_c2_seq7 comp55719_c0_seq1 comp57215_c2_seq16 comp54719_c0_seq3 comp57921_c0_seq3 comp55195_c4_seq1 comp52908_c0_seq10 comp50854_c0_seq3 comp29662_c0_seq1 comp51882_c0_seq2 comp50792_c0_seq2 comp56985_c0_seq1 comp54708_c1_seq7 comp55268_c2_seq6 comp48987_c0_seq3 comp47791_c1_seq1 comp57681_c0_seq7 comp57462_c0_seq3 comp55467_c4_seq5 comp54236_c0_seq1 comp57474_c0_seq1 comp57530_c6_seq1 comp55524_c0_seq2 comp51953_c0_seq1 comp57432_c0_seq9 comp54443_c0_seq2 comp49108_c1_seq1 comp54383_c1_seq1 comp53684_c2_seq2 comp50428_c1_seq2 comp28994_c0_seq1 comp49698_c0_seq2 comp32634_c0_seq1 comp53653_c0_seq2 comp52570_c0_seq3 comp55328_c0_seq4 comp30809_c0_seq2 comp55105_c0_seq2 comp54581_c0_seq14 comp57603_c10_seq3 comp50990_c1_seq2 comp53470_c0_seq2 comp47100_c0_seq1 comp55547_c0_seq1 comp54347_c4_seq1 comp52355_c0_seq2 comp56140_c1_seq2 comp57674_c1_seq5 comp53105_c0_seq1 comp51865_c0_seq8 comp54291_c1_seq4 comp52488_c0_seq5 comp57797_c1_seq6 comp56524_c2_seq1 comp48566_c0_seq2 comp30479_c0_seq1 comp57524_c0_seq13 comp60638_c0_seq1 comp54544_c0_seq4 comp57803_c4_seq3 comp57504_c2_seq5 comp46082_c0_seq1 comp58204_c0_seq1 comp55501_c1_seq5 comp56625_c1_seq7 comp56377_c2_seq2 comp52920_c0_seq1 comp50385_c0_seq1 comp57232_c1_seq5 comp50333_c0_seq2 comp56816_c0_seq9 comp57579_c1_seq8 comp53525_c0_seq6 comp55757_c0_seq2 comp54533_c2_seq3 comp53691_c0_seq1 comp45807_c2_seq1 comp55090_c0_seq1 comp46136_c0_seq1 comp30799_c0_seq1 comp51716_c0_seq1 comp60015_c0_seq1 comp44512_c0_seq1 comp52884_c1_seq1 comp51183_c1_seq1 comp52932_c0_seq7 comp56225_c1_seq1 comp49753_c0_seq1 comp57564_c2_seq55 comp32956_c0_seq1 comp54303_c0_seq7 comp53731_c0_seq2 comp55828_c0_seq1 comp54698_c1_seq4 comp45658_c0_seq3 comp32043_c0_seq1 comp55200_c0_seq4 comp52858_c0_seq2 comp56390_c3_seq2 comp55840_c1_seq2 comp55379_c0_seq3 comp55617_c0_seq4 comp49990_c0_seq1 comp58167_c1_seq6 comp51664_c0_seq2 comp29843_c0_seq1 comp56081_c0_seq1 comp56429_c0_seq7 comp53132_c0_seq5 comp30464_c0_seq1 comp56717_c0_seq1 comp57713_c0_seq9 comp57566_c4_seq4 comp57940_c1_seq2 comp55244_c0_seq2 comp58185_c2_seq5 comp57566_c8_seq1 comp61608_c0_seq1 comp48263_c0_seq1 comp56206_c0_seq1 comp51667_c3_seq2 comp50516_c0_seq1 comp48884_c0_seq2 comp54503_c0_seq2 comp51275_c0_seq1 comp47333_c0_seq1 comp30732_c0_seq1 comp46421_c0_seq2 comp49392_c0_seq8 comp57335_c0_seq10 comp43776_c0_seq1 comp52039_c0_seq1 comp57838_c0_seq11 comp44723_c0_seq4 comp48635_c0_seq2 comp49207_c0_seq1 comp54605_c2_seq9 comp46009_c0_seq3 comp36611_c0_seq2 comp55152_c0_seq2 comp44313_c2_seq1 comp47600_c0_seq2 comp49454_c0_seq4 comp45412_c0_seq2 comp51559_c0_seq2 comp55183_c0_seq3 comp48483_c0_seq1 comp51336_c0_seq1 comp56061_c1_seq8 comp57260_c0_seq5 comp58108_c1_seq16 comp53650_c5_seq1 comp55496_c3_seq7 comp54942_c1_seq6 comp57593_c0_seq3 comp47440_c0_seq3 comp53656_c1_seq2 comp55635_c2_seq2 comp44554_c0_seq1 comp32728_c0_seq1 comp53391_c0_seq4 comp56829_c1_seq7 comp54845_c0_seq2 comp53153_c0_seq1 comp56540_c0_seq10 comp57862_c4_seq3 comp54197_c0_seq2 comp53761_c3_seq1 comp41697_c0_seq1 comp56707_c0_seq4 comp57867_c1_seq8 comp49020_c0_seq1 comp51764_c3_seq1 comp53786_c2_seq3 comp66366_c0_seq1 comp49110_c2_seq4 comp54944_c13_seq1 comp58196_c0_seq4 comp46990_c1_seq5 comp50389_c0_seq4 comp31758_c0_seq1 comp46110_c0_seq1 comp42584_c4_seq3 comp57956_c0_seq2 comp55539_c0_seq6 comp44760_c2_seq1 comp58247_c0_seq29 comp55853_c0_seq4 comp58045_c0_seq2 comp56978_c0_seq1 comp54734_c0_seq4 comp58876_c0_seq1 comp57401_c0_seq1 comp54119_c1_seq1 comp51529_c1_seq1 comp57239_c2_seq21 comp57818_c5_seq24 comp55141_c0_seq15 comp48542_c1_seq3 comp57830_c4_seq4 comp58245_c1_seq28 comp54184_c0_seq12 comp43806_c0_seq1 comp55637_c0_seq3 comp56340_c1_seq1 comp43365_c0_seq1 comp36409_c1_seq1 comp58291_c0_seq3 comp28369_c0_seq1 comp56403_c0_seq3 comp52898_c0_seq1 comp30205_c0_seq1 comp52187_c0_seq5 comp55856_c1_seq6 comp52406_c0_seq1 comp58287_c0_seq9 comp56227_c1_seq9 comp46146_c0_seq1 comp47094_c0_seq4 comp51249_c0_seq1 comp57807_c4_seq1 comp45811_c0_seq1 comp46499_c0_seq1 comp48789_c0_seq1 comp32004_c0_seq1 comp50117_c3_seq1 comp47786_c2_seq1 comp50821_c1_seq1 comp58203_c5_seq12 comp49174_c1_seq2 comp48868_c0_seq1 comp51675_c1_seq1 comp55521_c0_seq1 comp57640_c1_seq8 comp54800_c0_seq2 comp46312_c0_seq1 comp58229_c0_seq11 comp50458_c0_seq1 comp53283_c0_seq5 comp49310_c0_seq2 comp53161_c0_seq1 comp45111_c0_seq1 comp39769_c1_seq1 comp56249_c0_seq6 comp39106_c0_seq1 comp54498_c0_seq6 comp57984_c0_seq1 comp55782_c1_seq3 comp51157_c1_seq3 comp57849_c2_seq7 comp35458_c1_seq1 comp50789_c0_seq2 comp58098_c2_seq19 comp55077_c0_seq2 comp57152_c4_seq55 comp55373_c0_seq2 comp52645_c0_seq1 comp57907_c10_seq1 comp53786_c3_seq2 comp48821_c1_seq1 comp56476_c1_seq1 comp51299_c0_seq1 comp55839_c0_seq2 comp55489_c0_seq1 comp54976_c0_seq1 comp57965_c0_seq8 comp52731_c0_seq3 comp46294_c0_seq1 comp54841_c8_seq4 comp56079_c4_seq2 comp49523_c0_seq3 comp57397_c9_seq5 comp43012_c2_seq1 comp45332_c1_seq1 comp48059_c0_seq1 comp45882_c0_seq1 comp48392_c1_seq2 comp50370_c0_seq1 comp56694_c1_seq1 comp53779_c2_seq8 comp47819_c1_seq4 comp50670_c0_seq2 comp53202_c4_seq2 comp52702_c0_seq1 comp46062_c0_seq2 comp29607_c1_seq1 comp57530_c1_seq1 comp51488_c0_seq4 comp58060_c0_seq11 comp51219_c0_seq1 comp55629_c1_seq6 comp46618_c0_seq7 comp57729_c2_seq3 comp55784_c0_seq5 comp52077_c4_seq9 comp57826_c6_seq4 comp53232_c0_seq4 comp54549_c1_seq10 comp55867_c3_seq1 comp51051_c0_seq1 comp56266_c0_seq4 comp54342_c0_seq1 comp47306_c0_seq1 comp51898_c0_seq1 comp56053_c0_seq1 comp56537_c0_seq1 comp57544_c6_seq2 comp53802_c0_seq1 comp55643_c0_seq1 comp48620_c0_seq1 comp42930_c0_seq2 comp55510_c0_seq6 comp55384_c0_seq3 comp61175_c0_seq1 comp57228_c0_seq1 comp31957_c0_seq1 comp47415_c0_seq1 comp50928_c0_seq1 comp56867_c2_seq1 comp32473_c0_seq2 comp49269_c1_seq1 comp46607_c0_seq1 comp42333_c0_seq3 comp55731_c1_seq8 comp29443_c1_seq1 comp56688_c5_seq1 comp32808_c0_seq1 comp29931_c0_seq1 comp47418_c0_seq1 comp57026_c0_seq3 comp54525_c3_seq2 comp57774_c2_seq1 comp57539_c2_seq4 comp45063_c0_seq1 comp53400_c1_seq8 comp45923_c1_seq2 comp58278_c0_seq4 comp52633_c1_seq1 comp56395_c12_seq10 comp53980_c0_seq1 comp53707_c0_seq4 comp56955_c2_seq1 comp56515_c4_seq6 comp56238_c1_seq5 comp41818_c0_seq2 comp53645_c1_seq1 comp47426_c0_seq1 comp56662_c0_seq15 comp55743_c1_seq1 comp52095_c1_seq1 comp47539_c1_seq1 comp30207_c0_seq1 comp50305_c0_seq3 comp32719_c0_seq1 comp54944_c8_seq1 comp47432_c1_seq1 comp53488_c0_seq1 comp57629_c3_seq19 comp54159_c1_seq2 comp57816_c1_seq7 comp57927_c0_seq1 comp53381_c0_seq1 comp58030_c0_seq3 comp56993_c0_seq1 comp48864_c0_seq1 comp43710_c1_seq3 comp57347_c0_seq4 comp55458_c0_seq3 comp57659_c5_seq1 comp52309_c1_seq1 comp57416_c1_seq11 comp47126_c0_seq1 comp45665_c0_seq1 comp58207_c0_seq7 comp45478_c1_seq2 comp49832_c0_seq2 comp51407_c2_seq2 comp56285_c0_seq3 comp55656_c0_seq14 comp50408_c3_seq1 comp56671_c0_seq3 comp56109_c2_seq6 comp55170_c0_seq1 comp36848_c0_seq1 comp37309_c0_seq1 comp57422_c0_seq1 comp46735_c0_seq4 comp58011_c1_seq2 comp56347_c0_seq9 comp59526_c0_seq1 comp57869_c0_seq3 comp57833_c0_seq29 comp49530_c1_seq2 comp57468_c0_seq12 comp54713_c0_seq3 comp49477_c0_seq3 comp49659_c2_seq1 comp54698_c1_seq28 comp57763_c0_seq2 comp57658_c3_seq1 comp57340_c0_seq2 comp52093_c2_seq3 comp56938_c4_seq3 comp57487_c5_seq7 comp54907_c1_seq2 comp56631_c1_seq3 comp55338_c0_seq1 comp51946_c1_seq1 comp53175_c3_seq6 comp31752_c0_seq1 comp57716_c2_seq3 comp57022_c12_seq1 comp48644_c0_seq1 comp49046_c0_seq1 comp55385_c0_seq7 comp53159_c1_seq3 comp56590_c1_seq3 comp29803_c0_seq1 comp58231_c1_seq3 comp60270_c0_seq1 comp58288_c0_seq12 comp44902_c0_seq1 comp58372_c0_seq1 comp56868_c2_seq2 comp33430_c0_seq1 comp50941_c0_seq1 comp57072_c0_seq2 comp29737_c0_seq1 comp55552_c0_seq3 comp56622_c1_seq8 comp57891_c0_seq1 comp53370_c0_seq7 comp54054_c1_seq1 comp57368_c9_seq1 comp52532_c0_seq2 comp55624_c0_seq4 comp33258_c0_seq1 comp51917_c0_seq1 comp57554_c1_seq2 comp49656_c0_seq1 comp52273_c0_seq1 comp55339_c2_seq2 comp57659_c4_seq1 comp29508_c0_seq1 comp49654_c0_seq2 comp48415_c0_seq3 comp44711_c0_seq1 comp55858_c0_seq4 comp46759_c1_seq1 comp55500_c0_seq2 comp42033_c0_seq2 comp50958_c1_seq2 comp57318_c0_seq6 comp45135_c1_seq1 comp27743_c0_seq1 comp52465_c1_seq1 comp57900_c0_seq9 comp40733_c0_seq1 comp54427_c1_seq1 comp55356_c0_seq8 comp52630_c1_seq3 comp57182_c0_seq1 comp56156_c0_seq3 comp52936_c1_seq2 comp57517_c4_seq7 comp58258_c0_seq7 comp51327_c0_seq1 comp53448_c5_seq1 comp49270_c0_seq2 comp30260_c0_seq1 comp52110_c1_seq3 comp55993_c5_seq1 comp57368_c3_seq1 comp51950_c0_seq1 comp49599_c0_seq1 comp50373_c0_seq1 comp55732_c2_seq2 comp32270_c0_seq1 comp47863_c0_seq1 comp45453_c2_seq1 comp57758_c2_seq1 comp57680_c0_seq5 comp49611_c2_seq1 comp52262_c0_seq4 comp56260_c0_seq6 comp57051_c12_seq1 comp53068_c0_seq3 comp51111_c0_seq3 comp54593_c0_seq4 comp52249_c1_seq1 comp54337_c0_seq9 comp42253_c0_seq1 comp43865_c0_seq2 comp56696_c3_seq2 comp61911_c0_seq1 comp53999_c4_seq1 comp55441_c0_seq2 comp57854_c0_seq3 comp48923_c0_seq4 comp56940_c1_seq1 comp50590_c0_seq2 comp57190_c0_seq6 comp46441_c0_seq2 comp53327_c0_seq4 comp58828_c0_seq1 comp55344_c1_seq10 comp53874_c0_seq1 comp52308_c0_seq1 comp47514_c1_seq2 comp51185_c1_seq4 comp58276_c2_seq9 comp49252_c0_seq1 comp54273_c0_seq3 comp53293_c0_seq2 comp50779_c0_seq1 comp46231_c0_seq2 comp53110_c2_seq12 comp58053_c2_seq8 comp47449_c0_seq1 comp53895_c0_seq3 comp52094_c0_seq2 comp57980_c0_seq1 comp52443_c0_seq1 comp47215_c0_seq2 comp50438_c0_seq2 comp55380_c0_seq1 comp45122_c0_seq1 comp58210_c0_seq2 comp145936_c0_seq1 comp47376_c1_seq1 comp51881_c2_seq1 comp56939_c0_seq3 comp57639_c1_seq6 comp56515_c4_seq2 comp30143_c0_seq1 comp31900_c0_seq1 comp55992_c0_seq3 comp49996_c0_seq1 comp41775_c1_seq1 comp51235_c0_seq1 comp48035_c0_seq1 comp57539_c2_seq8 comp50195_c1_seq2 comp109639_c0_seq1 comp40579_c0_seq1 comp57550_c0_seq7 comp49364_c0_seq4 comp53796_c0_seq1 comp54015_c0_seq2 comp55806_c1_seq3 comp55791_c0_seq1 comp51889_c1_seq1 comp57927_c3_seq13 comp44282_c0_seq1 comp52982_c0_seq2 comp48064_c0_seq1 comp42004_c0_seq1 comp56127_c2_seq1 comp55266_c3_seq3 comp50088_c1_seq2 comp55280_c3_seq4 comp47891_c0_seq1 comp57776_c3_seq15 comp57852_c0_seq5 comp57147_c0_seq9 comp57410_c1_seq5 comp45956_c4_seq1 comp48687_c1_seq1 comp39276_c0_seq3 comp52347_c1_seq1 comp56096_c4_seq5 comp57130_c2_seq3 comp45564_c1_seq1 comp51070_c0_seq2 comp51794_c0_seq1 comp55739_c0_seq2 comp57301_c0_seq2 comp50499_c1_seq1 comp54337_c0_seq14 comp52422_c0_seq1 comp57445_c0_seq12 comp50840_c1_seq1 comp53363_c0_seq1 comp54394_c4_seq24 comp53117_c1_seq1 comp54034_c0_seq2 comp53715_c0_seq5 comp50892_c0_seq3 comp35000_c0_seq2 comp56360_c0_seq1 comp51949_c1_seq1 comp55187_c0_seq1 comp48210_c0_seq4 comp41122_c0_seq1 comp57254_c9_seq11 comp52178_c1_seq2 comp53529_c2_seq2 comp55603_c3_seq2 comp48952_c0_seq1 comp34909_c0_seq1 comp56075_c4_seq1 comp57977_c0_seq2 comp52979_c2_seq3 comp29264_c0_seq1 comp50180_c2_seq2 comp57382_c1_seq1 comp108904_c0_seq1 comp56048_c0_seq6 comp57283_c0_seq3 comp48479_c2_seq1 comp54340_c0_seq6 comp54598_c3_seq1 comp57351_c3_seq1 comp56665_c2_seq9 comp53666_c0_seq4 comp52527_c0_seq3 comp53110_c2_seq5 comp53254_c2_seq1 comp57355_c1_seq9 comp56004_c1_seq1 comp33741_c0_seq1 comp57825_c2_seq18 comp56805_c0_seq2 comp56820_c0_seq4 comp54516_c2_seq3 comp54534_c0_seq2 comp51285_c0_seq5 comp55742_c0_seq10 comp57786_c1_seq9 comp55736_c1_seq5 comp50722_c3_seq1 comp53333_c0_seq2 comp48412_c0_seq2 comp57022_c7_seq6 comp49911_c0_seq1 comp47314_c4_seq1 comp53033_c0_seq4 comp53955_c1_seq1 comp54594_c1_seq1 comp42877_c0_seq1 comp54698_c0_seq1 comp51599_c0_seq1 comp56667_c0_seq26 comp57329_c3_seq2 comp56610_c2_seq8 comp50349_c1_seq4 comp54834_c0_seq1 comp56583_c2_seq6 comp53550_c0_seq1 comp48582_c0_seq6 comp56295_c0_seq2 comp50838_c0_seq1 comp56750_c1_seq2 comp52508_c0_seq3 comp57619_c0_seq22 comp53493_c0_seq5 comp55003_c0_seq5 comp56405_c0_seq1 comp49827_c1_seq3 comp57102_c2_seq7 comp41572_c0_seq1 comp49135_c0_seq3 comp53731_c1_seq1 comp52954_c0_seq1 comp53864_c0_seq4 comp61329_c0_seq1 comp55416_c1_seq8 comp56233_c3_seq1 comp56324_c0_seq5 comp58047_c0_seq9 comp48783_c0_seq4 comp57857_c1_seq15 comp55991_c2_seq5 comp57811_c1_seq13 comp55261_c0_seq1 comp48825_c2_seq3 comp50754_c0_seq2 comp131895_c0_seq1 comp43892_c0_seq1 comp47274_c0_seq1 comp56516_c2_seq10 comp58189_c0_seq5 comp56813_c3_seq4 comp53399_c0_seq2 comp55062_c0_seq1 comp57566_c15_seq11 comp50561_c1_seq1 comp48233_c1_seq3 comp56137_c0_seq1 comp56487_c4_seq19 comp42197_c1_seq1 comp53957_c0_seq3 comp55385_c0_seq6 comp55648_c1_seq2 comp50228_c0_seq1 comp55530_c1_seq3 comp45048_c0_seq1 comp52491_c0_seq1 comp55078_c0_seq1 comp57070_c1_seq8 comp45511_c0_seq1 comp44202_c0_seq1 comp58087_c8_seq1 comp56306_c4_seq5 comp57341_c5_seq18 comp52966_c0_seq1 comp57001_c3_seq11 comp58152_c0_seq5 comp46280_c0_seq2 comp57777_c0_seq5 comp52586_c0_seq4 comp53894_c0_seq2 comp45813_c0_seq1 comp57614_c0_seq4 comp54692_c0_seq2 comp103782_c0_seq1 comp55575_c1_seq1 comp50966_c2_seq1 comp52183_c0_seq16 comp47725_c0_seq1 comp55254_c0_seq4 comp52223_c2_seq1 comp54528_c0_seq1 comp41159_c0_seq1 comp53173_c0_seq7 comp52119_c0_seq2 comp56859_c1_seq19 comp49429_c0_seq16 comp55345_c0_seq6 comp45979_c0_seq1 comp57022_c7_seq2 comp105219_c0_seq1 comp57096_c0_seq5 comp31845_c0_seq1 comp54440_c0_seq1 comp56775_c2_seq34 comp49063_c0_seq3 comp55233_c0_seq5 comp55491_c3_seq4 comp55846_c0_seq1 comp47567_c0_seq1 comp52389_c0_seq2 comp28698_c0_seq1 comp56987_c3_seq2 comp57115_c0_seq7 comp57661_c2_seq4 comp51413_c2_seq4 comp56907_c5_seq2 comp56599_c2_seq1 comp46155_c0_seq3 comp57910_c0_seq1 comp46578_c0_seq4 comp26119_c0_seq1 comp51875_c0_seq1 comp54161_c0_seq2 comp58206_c6_seq1 comp52929_c0_seq2 comp48097_c0_seq3 comp57893_c6_seq3 comp56189_c0_seq2 comp57903_c0_seq2 comp54391_c1_seq8 comp57847_c0_seq5 comp55646_c0_seq6 comp46684_c2_seq1 comp50765_c1_seq1 comp57810_c0_seq7 comp56192_c1_seq1 comp57152_c4_seq67 comp54039_c0_seq1 comp53823_c2_seq1 comp49107_c0_seq1 comp57748_c3_seq1 comp56070_c0_seq2 comp57850_c1_seq2 comp52762_c0_seq3 comp54891_c2_seq1 comp58490_c0_seq1 comp56117_c1_seq9 comp56809_c5_seq1 comp53089_c0_seq1 comp50647_c0_seq1 comp56829_c1_seq2 comp52833_c0_seq16 comp56221_c0_seq2 comp39659_c0_seq1 comp28494_c0_seq1 comp51163_c0_seq1 comp56141_c2_seq8 comp54837_c0_seq1 comp54079_c3_seq2 comp53427_c0_seq2 comp53379_c1_seq2 comp53778_c0_seq1 comp57780_c4_seq1 comp52662_c0_seq2 comp51888_c1_seq1 comp49258_c1_seq1 comp57838_c0_seq4 comp54424_c0_seq1 comp33149_c0_seq1 comp55337_c1_seq2 comp36313_c0_seq1 comp47201_c0_seq1 comp46940_c0_seq1 comp48225_c0_seq1 comp54563_c0_seq2 comp47780_c1_seq1 comp53241_c0_seq4 comp57839_c0_seq7 comp56267_c0_seq1 comp53640_c0_seq1 comp55328_c1_seq4 comp53522_c1_seq1 comp53484_c0_seq2 comp48489_c0_seq1 comp47803_c1_seq2 comp57624_c2_seq5 comp51210_c0_seq1 comp56314_c3_seq1 comp52680_c0_seq1 comp57194_c1_seq3 comp57428_c0_seq5 comp54904_c0_seq3 comp28726_c0_seq2 comp55185_c1_seq25 comp55809_c1_seq12 comp56286_c3_seq8 comp53914_c3_seq5 comp56477_c1_seq2 comp57979_c0_seq2 comp53952_c0_seq2 comp29867_c0_seq1 comp54918_c0_seq3 comp56625_c0_seq2 comp58016_c5_seq2 comp49462_c0_seq2 comp55153_c2_seq1 comp49423_c0_seq1 comp29312_c0_seq2 comp28048_c0_seq2 comp55176_c0_seq1 comp54364_c0_seq2 comp57604_c1_seq1 comp55252_c0_seq1 comp57680_c0_seq16 comp57869_c0_seq13 comp57071_c0_seq1 comp55810_c0_seq1 comp64346_c0_seq1 comp46097_c1_seq1 comp57649_c3_seq12 comp48851_c2_seq1 comp51556_c1_seq2 comp55360_c0_seq1 comp54135_c1_seq2 comp57338_c1_seq2 comp56653_c1_seq23 comp57462_c0_seq6 comp38262_c0_seq1 comp56572_c0_seq5 comp55084_c0_seq11 comp58252_c3_seq17 comp55139_c1_seq5 comp52919_c0_seq4 comp56381_c0_seq4 comp50426_c2_seq1 comp55995_c10_seq66 comp55964_c0_seq5 comp54871_c0_seq1 comp58188_c0_seq3 comp54198_c0_seq1 comp43403_c0_seq1 comp32876_c1_seq1 comp58233_c8_seq9 comp49703_c0_seq2 comp57084_c0_seq5 comp56159_c0_seq2 comp57530_c3_seq3 comp30521_c0_seq2 comp44641_c0_seq1 comp34248_c0_seq1 comp213591_c0_seq1 comp48408_c0_seq1 comp63505_c0_seq1 comp53214_c0_seq1 comp37759_c1_seq1 comp57756_c3_seq4 comp57102_c2_seq5 comp50402_c0_seq1 comp55370_c1_seq8 comp58199_c0_seq1 comp52797_c0_seq3 comp56170_c4_seq2 comp56981_c0_seq2 comp56259_c3_seq1 comp57466_c3_seq5 comp43976_c0_seq1 comp56940_c1_seq6 comp54281_c1_seq1 comp58178_c0_seq1 comp57202_c3_seq4 comp57944_c0_seq1 comp53915_c0_seq4 comp53048_c0_seq1 comp57818_c1_seq8 comp45192_c1_seq1 comp57254_c1_seq1 comp52141_c1_seq2 comp55599_c0_seq2 comp29166_c0_seq1 comp55959_c0_seq1 comp49206_c0_seq1 comp50041_c1_seq1 comp55888_c0_seq1 comp55908_c1_seq4 comp54695_c1_seq1 comp50033_c0_seq1 comp58292_c0_seq3 comp57810_c1_seq1 comp57390_c1_seq10 comp57822_c2_seq1 comp58038_c1_seq6 comp54807_c0_seq2 comp57365_c0_seq11 comp52955_c0_seq1 comp52150_c1_seq2 comp55122_c0_seq1 comp46095_c0_seq1 comp56541_c0_seq4 comp57349_c2_seq1 comp57466_c5_seq3 comp50384_c1_seq1 comp53202_c4_seq8 comp55881_c1_seq2 comp45727_c0_seq1 comp52941_c1_seq4 comp56268_c0_seq7 comp56127_c1_seq1 comp52678_c0_seq1 comp46268_c2_seq2 comp51357_c0_seq1 comp52421_c0_seq4 comp58139_c1_seq9 comp43584_c1_seq1 comp57931_c0_seq1 comp55891_c0_seq4 comp58063_c0_seq26 comp50680_c0_seq1 comp47410_c0_seq1 comp55996_c0_seq1 comp58086_c1_seq2 comp57082_c2_seq3 comp55181_c0_seq8 comp57890_c0_seq33 comp56924_c4_seq1 comp53364_c0_seq6 comp47221_c0_seq2 comp184225_c0_seq1 comp51644_c1_seq1 comp43279_c0_seq2 comp51699_c1_seq3 comp3295_c0_seq1 comp58096_c7_seq2 comp56694_c0_seq3 comp56199_c0_seq16 comp32357_c0_seq2 comp56056_c0_seq2 comp57019_c0_seq2 comp56378_c4_seq8 comp47261_c0_seq3 comp51523_c0_seq1 comp56879_c0_seq4 comp112295_c0_seq1 comp52475_c0_seq3 comp44863_c0_seq1 comp61780_c0_seq1 comp55762_c4_seq7 comp32630_c0_seq1 comp57696_c3_seq14 comp52423_c0_seq1 comp46584_c0_seq1 comp56968_c0_seq1 comp30151_c0_seq2 comp45473_c0_seq1 comp58269_c0_seq16 comp60771_c0_seq1 comp54052_c0_seq2 comp53225_c0_seq2 comp76334_c0_seq1 comp54339_c2_seq2 comp58045_c0_seq26 comp56273_c0_seq1 comp55087_c1_seq3 comp56603_c3_seq4 comp49600_c0_seq2 comp53553_c0_seq2 comp42955_c0_seq1 comp45830_c0_seq1 comp57411_c0_seq1 comp46048_c0_seq4 comp52909_c3_seq1 comp58257_c0_seq1 comp58255_c1_seq2 comp55470_c0_seq4 comp57150_c0_seq2 comp39537_c1_seq1 comp57153_c1_seq9 comp49404_c1_seq3 comp52804_c0_seq1 comp48632_c3_seq2 comp49194_c0_seq2 comp55755_c1_seq1 comp56967_c0_seq5 comp53619_c0_seq4 comp51575_c0_seq1 comp53828_c0_seq1 comp43325_c0_seq1 comp55718_c1_seq4 comp55131_c0_seq4 comp48159_c0_seq3 comp57578_c1_seq26 comp42049_c1_seq1 comp43870_c0_seq1 comp48265_c0_seq1 comp48748_c0_seq4 comp52406_c0_seq2 comp58226_c0_seq7 comp56856_c2_seq3 comp54884_c2_seq1 comp51601_c0_seq1 comp42709_c0_seq1 comp50397_c0_seq5 comp58267_c1_seq9 comp55009_c0_seq6 comp52885_c0_seq3 comp56083_c0_seq3 comp36886_c0_seq1 comp54191_c0_seq1 comp39065_c0_seq2 comp54306_c15_seq2 comp50903_c0_seq8 comp58175_c1_seq2 comp56748_c2_seq3 comp54234_c0_seq3 comp52022_c3_seq4 comp52299_c0_seq6 comp58132_c0_seq3 comp46812_c0_seq2 comp57804_c2_seq8 comp51517_c0_seq4 comp45914_c2_seq1 comp53733_c0_seq1 comp55816_c0_seq8 comp50115_c0_seq2 comp55168_c15_seq2 comp57239_c2_seq12 comp57635_c1_seq2 comp37945_c0_seq1 comp58060_c0_seq14 comp58265_c2_seq13 comp48711_c1_seq4 comp50338_c1_seq1 comp47059_c0_seq1 comp46105_c0_seq3 comp48397_c0_seq1 comp51269_c0_seq2 comp52364_c0_seq1 comp54769_c0_seq1 comp53639_c2_seq1 comp46197_c0_seq1 comp57569_c1_seq22 comp53188_c0_seq5 comp41757_c0_seq1 comp58255_c1_seq11 comp27899_c0_seq1 comp55894_c0_seq4 comp54864_c2_seq1 comp56544_c0_seq2 comp48467_c0_seq2 comp52076_c0_seq4 comp57001_c4_seq1 comp56722_c1_seq16 comp51002_c0_seq1 comp48430_c0_seq3 comp54193_c11_seq4 comp56297_c1_seq1 comp58300_c0_seq16 comp29412_c0_seq1 comp30803_c0_seq1 comp58119_c0_seq1 comp55353_c1_seq13 comp58133_c3_seq1 comp47796_c0_seq1 comp58246_c1_seq12 comp54338_c0_seq1 comp52806_c0_seq2 comp55546_c0_seq4 comp50793_c3_seq5 comp31996_c0_seq1 comp49428_c0_seq1 comp32931_c0_seq4 comp48940_c0_seq2 comp27772_c0_seq1 comp54500_c2_seq1 comp56881_c0_seq4 comp56398_c1_seq8 comp56538_c0_seq3 comp55749_c7_seq4 comp30705_c0_seq1 comp56929_c4_seq5 comp58137_c2_seq7 comp54136_c0_seq5 comp54753_c0_seq2 comp55118_c0_seq3 comp55238_c7_seq1 comp36445_c0_seq1 comp57902_c0_seq1 comp57260_c0_seq6 comp56527_c0_seq2 comp56604_c1_seq1 comp30048_c0_seq1 comp57098_c4_seq12 comp48106_c0_seq1 comp51096_c0_seq1 comp57986_c0_seq10 comp56462_c1_seq1 comp55412_c0_seq1 comp57963_c2_seq6 comp47257_c0_seq2 comp56763_c2_seq1 comp29092_c0_seq1 comp51492_c0_seq3 comp58244_c0_seq13 comp45884_c0_seq3 comp47904_c0_seq1 comp56439_c0_seq1 comp43321_c0_seq1 comp58171_c0_seq7 comp56860_c3_seq2 comp48936_c0_seq1 comp57023_c1_seq6 comp55512_c1_seq1 comp282065_c0_seq1 comp54507_c0_seq1 comp55967_c0_seq1 comp57082_c0_seq7 comp41142_c0_seq1 comp50848_c0_seq5 comp51401_c0_seq4 comp54222_c0_seq4 comp55589_c3_seq5 comp43919_c1_seq1 comp56354_c1_seq3 comp56601_c0_seq3 comp55025_c0_seq2 comp57953_c3_seq34 comp111979_c0_seq1 comp57579_c2_seq5 comp31075_c0_seq1 comp54987_c0_seq2 comp51373_c0_seq1 comp51093_c0_seq1 comp54495_c0_seq1 comp58235_c0_seq11 comp31597_c0_seq1 comp57907_c12_seq2 comp51901_c0_seq1 comp51886_c0_seq3 comp54337_c0_seq2 comp43981_c0_seq1 comp58213_c0_seq5 comp57056_c1_seq2 comp57388_c2_seq6 comp48532_c0_seq1 comp50932_c2_seq1 comp54757_c0_seq1 comp61106_c0_seq1 comp38856_c2_seq1 comp46041_c0_seq2 comp52445_c0_seq1 comp54187_c0_seq3 comp38974_c0_seq1 comp50924_c0_seq1 comp48496_c0_seq1 comp39823_c1_seq1 comp57159_c1_seq2 comp57728_c2_seq23 comp53787_c0_seq6 comp54236_c0_seq2 comp53087_c1_seq1 comp53335_c0_seq2 comp31021_c0_seq1 comp48056_c0_seq1 comp48769_c1_seq1 comp44015_c0_seq2 comp51840_c0_seq2 comp54205_c1_seq4 comp52465_c1_seq3 comp52472_c0_seq2 comp49750_c1_seq5 comp55500_c0_seq4 comp56910_c2_seq3 comp53703_c4_seq1 comp32442_c0_seq1 comp47232_c1_seq1 comp49460_c0_seq1 comp51403_c1_seq1 comp49864_c1_seq1 comp54181_c1_seq5 comp27767_c0_seq1 comp47568_c0_seq1 comp57070_c1_seq4 comp56481_c2_seq20 comp55179_c0_seq4 comp58127_c0_seq6 comp50079_c0_seq3 comp51800_c1_seq1 comp56828_c0_seq7 comp49918_c1_seq1 comp41341_c0_seq1 comp51378_c1_seq2 comp53226_c0_seq1 comp45757_c0_seq1 comp45612_c0_seq1 comp50822_c0_seq1 comp41881_c0_seq2 comp56128_c0_seq1 comp50138_c0_seq1 comp57926_c8_seq7 comp54711_c1_seq5 comp48602_c0_seq2 comp46917_c1_seq3 comp53783_c2_seq1 comp54611_c0_seq3 comp53526_c0_seq2 comp53629_c0_seq2 comp42261_c0_seq1 comp51228_c2_seq4 comp55099_c0_seq2 comp57336_c0_seq3 comp57603_c3_seq2 comp55248_c1_seq4 comp47236_c0_seq1 comp57028_c0_seq4 comp29738_c0_seq1 comp53489_c1_seq2 comp53934_c0_seq4 comp49533_c0_seq1 comp56912_c0_seq4 comp57651_c1_seq1 comp57740_c6_seq6 comp43983_c0_seq1 comp57680_c0_seq9 comp56144_c0_seq3 comp57728_c2_seq6 comp46867_c0_seq2 comp53724_c0_seq4 comp57488_c2_seq1 comp50438_c0_seq3 comp56496_c0_seq1 comp56481_c2_seq5 comp57568_c3_seq10 comp57239_c2_seq4 comp40046_c1_seq1 comp51166_c2_seq1 comp55155_c0_seq1 comp55194_c0_seq2 comp46180_c0_seq1 comp55022_c0_seq1 comp55247_c0_seq5 comp52893_c0_seq1 comp53754_c0_seq1 comp48150_c0_seq2 comp56875_c0_seq6 comp30930_c0_seq1 comp57499_c6_seq21 comp52202_c0_seq1 comp58104_c2_seq3 comp55792_c0_seq2 comp54866_c0_seq5 comp52801_c0_seq3 comp113338_c0_seq1 comp58174_c1_seq9 comp56104_c3_seq1 comp29386_c0_seq1 comp53534_c4_seq1 comp28657_c0_seq1 comp57629_c3_seq8 comp55442_c0_seq2 comp41684_c1_seq1 comp45088_c0_seq1 comp56422_c1_seq3 comp52253_c0_seq1 comp58030_c0_seq2 comp56700_c7_seq2 comp55126_c0_seq1 comp52986_c2_seq1 comp58163_c4_seq3 comp57645_c3_seq10 comp53719_c1_seq1 comp51559_c0_seq3 comp52386_c0_seq1 comp42140_c0_seq1 comp57153_c1_seq13 comp55108_c0_seq1 comp58031_c2_seq3 comp50001_c1_seq2 comp58168_c3_seq7 comp54788_c0_seq3 comp42756_c1_seq1 comp54183_c1_seq1 comp44720_c0_seq1 comp45217_c1_seq1 comp53200_c0_seq1 comp42068_c0_seq1 comp55973_c1_seq8 comp53668_c0_seq1 comp53581_c0_seq1 comp53227_c1_seq3 comp53854_c0_seq1 comp41558_c0_seq1 comp52439_c0_seq4 comp128534_c0_seq1 comp56588_c4_seq3 comp54190_c6_seq4 comp31289_c0_seq2 comp55176_c1_seq1 comp52698_c0_seq6 comp54297_c1_seq1 comp49977_c2_seq1 comp58151_c1_seq1 comp44232_c0_seq2 comp54032_c0_seq1 comp53931_c0_seq2 comp29386_c1_seq1 comp46544_c0_seq1 comp38454_c0_seq1 comp53398_c4_seq2 comp55024_c0_seq1 comp50977_c1_seq1 comp58293_c2_seq2 comp54359_c2_seq9 comp51618_c0_seq3 comp55220_c0_seq2 comp54515_c2_seq2 comp50681_c0_seq1 comp55993_c6_seq1 comp54782_c1_seq5 comp51604_c0_seq2 comp57466_c2_seq3 comp50041_c1_seq7 comp50017_c0_seq1 comp55207_c3_seq2 comp51621_c2_seq6 comp56944_c0_seq4 comp57701_c2_seq14 comp48730_c0_seq3 comp56610_c6_seq1 comp52420_c0_seq1 comp47789_c1_seq2 comp58262_c4_seq16 comp40554_c0_seq1 comp50360_c0_seq3 comp47633_c0_seq3 comp53781_c0_seq3 comp50615_c1_seq1 comp56237_c0_seq1 comp47984_c0_seq1 comp165317_c0_seq1 comp57096_c3_seq1 comp55839_c0_seq5 comp53688_c0_seq6 comp48131_c0_seq2 comp54970_c1_seq1 comp53722_c2_seq1 comp57570_c0_seq1 comp56302_c0_seq2 comp57380_c1_seq2 comp57819_c0_seq3 comp27824_c1_seq1 comp49559_c0_seq2 comp51252_c4_seq1 comp47253_c0_seq3 comp51573_c3_seq4 comp46009_c1_seq1 comp56574_c0_seq3 comp44880_c0_seq1 comp58238_c1_seq43 comp56634_c6_seq2 comp55386_c0_seq4 comp48775_c0_seq1 comp57764_c1_seq19 comp45882_c0_seq2 comp49555_c0_seq1 comp49331_c0_seq2 comp56846_c3_seq4 comp52519_c0_seq2 comp48104_c0_seq2 comp29486_c0_seq1 comp42946_c0_seq1 comp58230_c2_seq1 comp57139_c0_seq1 comp29344_c0_seq1 comp58116_c1_seq3 comp57079_c2_seq4 comp46309_c0_seq1 comp32529_c0_seq1 comp31986_c0_seq1 comp51866_c0_seq1 comp50315_c0_seq2 comp56659_c6_seq3 comp53423_c0_seq2 comp32248_c0_seq1 comp55353_c1_seq3 comp53711_c0_seq4 comp50072_c0_seq1 comp31602_c0_seq1 comp52213_c0_seq8 comp47313_c0_seq4 comp52444_c1_seq3 comp56737_c4_seq1 comp50551_c0_seq1 comp36154_c1_seq1 comp32445_c0_seq1 comp52513_c1_seq2 comp56831_c0_seq5 comp56941_c0_seq1 comp32858_c0_seq1 comp49167_c0_seq1 comp45845_c0_seq2 comp54773_c0_seq7 comp57500_c2_seq5 comp48243_c0_seq1 comp56809_c4_seq3 comp50298_c0_seq1 comp282429_c0_seq1 comp48915_c3_seq2 comp56804_c2_seq1 comp50877_c2_seq3 comp56909_c0_seq1 comp59015_c0_seq1 comp50811_c1_seq1 comp57526_c0_seq5 comp53845_c0_seq1 comp53013_c1_seq6 comp44676_c1_seq1 comp57579_c2_seq2 comp31481_c0_seq2 comp53478_c0_seq2 comp47233_c1_seq1 comp55072_c0_seq1 comp47615_c2_seq1 comp52196_c2_seq2 comp56227_c1_seq5 comp56817_c2_seq3 comp51034_c0_seq4 comp54817_c9_seq3 comp51360_c1_seq5 comp41540_c0_seq1 comp56354_c1_seq16 comp54261_c6_seq1 comp50026_c0_seq1 comp57899_c2_seq12 comp52381_c0_seq7 comp56529_c3_seq1 comp53155_c0_seq5 comp48996_c0_seq1 comp58006_c1_seq1 comp55957_c0_seq1 comp43667_c0_seq1 comp29678_c0_seq1 comp57400_c1_seq1 comp57717_c0_seq2 comp48965_c0_seq1 comp56737_c5_seq28 comp55923_c0_seq2 comp59008_c0_seq1 comp48291_c1_seq1 comp58345_c0_seq1 comp57889_c0_seq2 comp53732_c3_seq1 comp50097_c3_seq8 comp54785_c1_seq12 comp54811_c1_seq2 comp54627_c2_seq1 comp51131_c3_seq1 comp57604_c0_seq3 comp52654_c0_seq5 comp53364_c0_seq5 comp48667_c0_seq2 comp57316_c1_seq8 comp52708_c0_seq1 comp58222_c0_seq1 comp58052_c0_seq1 comp4856_c1_seq1 comp52738_c0_seq6 comp54772_c3_seq3 comp54635_c0_seq2 comp48117_c1_seq1 comp34907_c0_seq1 comp55643_c0_seq3 comp54980_c0_seq1 comp43078_c0_seq1 comp53916_c1_seq3 comp56817_c0_seq1 comp54450_c0_seq1 comp46767_c0_seq1 comp53149_c0_seq3 comp48198_c0_seq1 comp49148_c1_seq1 comp56618_c0_seq10 comp160064_c0_seq1 comp56850_c0_seq1 comp57324_c1_seq19 comp54106_c0_seq1 comp49743_c2_seq1 comp47068_c0_seq2 comp57122_c0_seq9 comp56718_c4_seq1 comp55716_c0_seq4 comp52223_c1_seq2 comp54233_c0_seq1 comp54119_c1_seq5 comp55314_c0_seq1 comp56920_c2_seq1 comp60720_c0_seq1 comp57577_c1_seq1 comp34329_c0_seq1 comp48695_c1_seq1 comp52368_c0_seq1 comp53367_c2_seq1 comp57917_c0_seq13 comp58047_c0_seq20 comp55340_c0_seq2 comp56007_c0_seq2 comp49031_c0_seq1 comp29315_c0_seq1 comp30057_c0_seq1 comp58914_c0_seq1 comp55676_c0_seq1 comp50264_c0_seq1 comp45662_c1_seq3 comp52716_c0_seq8 comp57313_c0_seq10 comp45650_c0_seq1 comp56249_c0_seq1 comp49835_c0_seq1 comp53336_c2_seq2 comp55831_c7_seq1 comp57665_c2_seq2 comp57124_c1_seq13 comp55516_c1_seq11 comp50189_c0_seq1 comp49339_c2_seq3 comp57776_c2_seq2 comp50703_c2_seq1 comp57681_c1_seq15 comp56264_c0_seq4 comp53692_c0_seq1 comp47239_c0_seq1 comp58225_c0_seq11 comp47536_c0_seq1 comp44942_c3_seq1 comp45755_c0_seq1 comp52787_c0_seq1 comp30981_c0_seq2 comp29034_c0_seq1 comp55565_c0_seq1 comp56802_c1_seq4 comp56068_c0_seq4 comp51083_c0_seq2 comp54567_c0_seq6 comp45354_c0_seq2 comp29184_c0_seq1 comp57157_c0_seq10 comp57716_c2_seq8 comp53083_c0_seq1 comp56312_c1_seq3 comp53635_c0_seq1 comp52143_c0_seq1 comp42342_c0_seq1 comp52474_c1_seq1 comp47247_c0_seq4 comp56444_c5_seq7 comp29570_c0_seq2 comp49333_c0_seq1 comp50517_c1_seq2 comp57867_c1_seq7 comp57520_c0_seq8 comp50423_c1_seq3 comp50677_c0_seq1 comp55268_c1_seq1 comp58247_c1_seq2 comp50096_c0_seq1 comp56724_c0_seq2 comp47571_c0_seq1 comp57434_c4_seq9 comp56299_c3_seq6 comp48669_c0_seq2 comp53132_c0_seq2 comp56340_c0_seq11 comp50274_c0_seq4 comp57376_c0_seq5 comp56175_c0_seq1 comp57221_c0_seq11 comp57335_c0_seq13 comp52913_c2_seq4 comp57807_c2_seq1 comp57755_c2_seq2 comp57992_c0_seq13 comp49654_c0_seq7 comp32237_c0_seq1 comp57875_c0_seq5 comp54657_c0_seq3 comp50402_c0_seq2 comp51788_c0_seq2 comp48903_c0_seq1 comp47144_c0_seq1 comp54894_c0_seq4 comp77222_c0_seq1 comp57641_c1_seq2 comp56531_c0_seq9 comp53379_c1_seq5 comp55491_c3_seq9 comp56045_c0_seq3 comp58367_c0_seq1 comp49583_c0_seq1 comp49453_c0_seq1 comp57523_c0_seq3 comp55238_c6_seq2 comp55787_c0_seq1 comp50002_c0_seq1 comp55835_c0_seq6 comp56535_c0_seq1 comp58233_c8_seq10 comp56800_c1_seq5 comp53786_c3_seq5 comp50937_c0_seq1 comp51043_c0_seq1 comp53632_c0_seq1 comp56091_c1_seq4 comp56705_c1_seq5 comp55837_c0_seq2 comp57419_c1_seq1 comp52818_c1_seq2 comp42906_c0_seq4 comp56970_c0_seq3 comp55503_c2_seq1 comp53022_c2_seq1 comp29283_c0_seq1 comp47643_c0_seq1 comp55306_c2_seq1 comp50585_c0_seq2 comp56857_c2_seq6 comp57739_c6_seq7 comp58184_c0_seq12 comp56787_c0_seq5 comp50886_c1_seq2 comp38106_c0_seq1 comp57131_c0_seq2 comp29343_c0_seq1 comp57116_c4_seq22 comp46580_c0_seq1 comp55327_c1_seq1 comp55836_c0_seq4 comp40132_c0_seq1 comp51263_c0_seq2 comp56670_c0_seq4 comp46291_c0_seq1 comp48440_c0_seq4 comp50725_c1_seq2 comp55610_c0_seq1 comp50514_c0_seq1 comp56987_c2_seq3 comp51665_c1_seq2 comp58265_c2_seq1 comp48961_c0_seq1 comp52276_c0_seq3 comp54430_c0_seq4 comp53194_c0_seq4 comp58202_c2_seq12 comp54576_c0_seq2 comp57286_c0_seq3 comp57555_c3_seq2 comp46887_c0_seq4 comp49196_c1_seq2 comp56399_c2_seq2 comp52871_c0_seq2 comp56141_c2_seq17 comp32535_c0_seq1 comp165058_c0_seq1 comp56508_c1_seq1 comp48406_c2_seq1 comp52428_c0_seq1 comp55060_c2_seq5 comp56288_c1_seq7 comp53873_c0_seq3 comp57366_c1_seq5 comp56115_c1_seq3 comp57283_c0_seq9 comp57260_c0_seq16 comp51142_c2_seq1 comp56018_c2_seq2 comp55391_c0_seq3 comp55568_c0_seq1 comp57776_c1_seq1 comp53623_c3_seq1 comp56735_c0_seq9 comp47160_c0_seq1 comp46440_c0_seq2 comp48071_c0_seq6 comp48924_c0_seq1 comp57179_c0_seq6 comp51266_c0_seq2 comp58086_c1_seq23 comp38053_c0_seq1 comp57422_c0_seq6 comp55809_c1_seq1 comp58200_c2_seq13 comp47713_c1_seq1 comp57700_c0_seq2 comp50989_c0_seq1 comp56667_c0_seq7 comp57949_c0_seq3 comp55814_c0_seq2 comp51307_c0_seq1 comp53886_c0_seq8 comp57618_c0_seq3 comp56195_c0_seq3 comp49746_c0_seq1 comp56714_c3_seq14 comp49348_c1_seq1 comp57424_c0_seq2 comp56497_c1_seq6 comp54918_c0_seq4 comp58123_c0_seq15 comp58001_c3_seq22 comp57051_c12_seq3 comp57389_c0_seq15 comp50265_c0_seq1 comp48251_c0_seq1 comp51988_c0_seq4 comp49713_c0_seq1 comp54002_c0_seq1 comp57245_c0_seq1 comp58115_c0_seq3 comp53175_c3_seq3 comp57635_c1_seq3 comp42092_c1_seq1 comp57539_c1_seq2 comp58267_c1_seq2 comp57315_c0_seq3 comp51453_c0_seq2 comp58057_c2_seq18 comp52605_c0_seq4 comp32563_c0_seq1 comp44014_c1_seq1 comp31663_c0_seq1 comp53493_c0_seq2 comp49807_c0_seq1 comp53476_c0_seq1 comp28536_c0_seq1 comp57754_c2_seq6 comp57548_c0_seq3 comp49361_c2_seq2 comp57834_c2_seq3 comp58276_c2_seq17 comp56151_c0_seq26 comp58152_c0_seq3 comp52498_c0_seq1 comp43197_c0_seq1 comp57764_c1_seq16 comp52982_c0_seq9 comp57461_c0_seq1 comp51802_c1_seq3 comp49814_c0_seq1 comp53259_c1_seq2 comp53949_c0_seq1 comp54527_c1_seq1 comp43381_c0_seq2 comp56820_c0_seq7 comp53379_c1_seq1 comp55649_c0_seq4 comp54712_c0_seq9 comp55816_c0_seq4 comp57863_c4_seq5 comp47354_c1_seq1 comp46317_c0_seq1 comp50048_c0_seq1 comp48740_c0_seq1 comp64526_c0_seq1 comp55191_c0_seq4 comp52549_c0_seq2 comp41378_c1_seq2 comp58087_c5_seq2 comp51928_c0_seq2 comp57086_c1_seq5 comp57705_c6_seq1 comp48573_c1_seq1 comp46513_c0_seq2 comp53942_c1_seq2 comp58231_c1_seq6 comp43865_c0_seq1 comp50258_c0_seq2 comp50199_c0_seq1 comp54085_c1_seq3 comp52433_c0_seq1 comp85274_c0_seq1 comp55847_c0_seq1 comp49586_c0_seq1 comp57637_c0_seq10 comp57096_c1_seq3 comp56027_c0_seq7 comp58463_c0_seq1 comp51295_c0_seq1 comp57406_c0_seq2 comp57005_c0_seq5 comp57072_c0_seq1 comp55703_c1_seq1 comp52693_c0_seq4 comp40682_c0_seq2 comp45000_c1_seq1 comp49287_c0_seq3 comp52664_c1_seq2 comp34502_c0_seq3 comp54227_c0_seq9 comp56173_c0_seq1 comp54472_c0_seq1 comp55368_c0_seq1 comp56399_c2_seq1 comp37905_c0_seq1 comp56987_c3_seq1 comp54378_c4_seq1 comp51544_c0_seq1 comp51706_c0_seq1 comp43412_c0_seq1 comp58274_c3_seq1 comp52554_c0_seq3 comp52125_c0_seq1 comp54538_c0_seq1 comp46647_c0_seq2 comp55239_c0_seq4 comp55719_c0_seq2 comp49895_c0_seq4 comp38713_c0_seq1 comp58199_c0_seq3 comp55495_c8_seq1 comp48443_c0_seq1 comp48217_c1_seq4 comp52103_c0_seq3 comp52044_c3_seq1 comp55557_c0_seq10 comp48952_c0_seq2 comp51958_c2_seq2 comp50325_c0_seq3 comp55037_c0_seq6 comp45939_c0_seq1 comp45419_c0_seq1 comp57637_c0_seq7 comp56235_c3_seq14 comp51556_c0_seq1 comp53056_c0_seq1 comp52951_c0_seq4 comp55984_c1_seq1 comp59951_c0_seq1 comp57742_c0_seq1 comp54458_c2_seq1 comp53769_c0_seq1 comp54398_c0_seq4 comp54069_c0_seq5 comp55682_c0_seq3 comp34112_c0_seq1 comp57180_c1_seq1 comp57366_c1_seq1 comp52081_c0_seq7 comp56813_c3_seq1 comp51238_c0_seq1 comp30864_c0_seq2 comp48969_c0_seq1 comp56701_c0_seq1 comp41151_c0_seq1 comp55603_c3_seq7 comp52387_c0_seq1 comp57982_c2_seq1 comp57942_c0_seq4 comp54528_c0_seq4 comp50722_c6_seq1 comp46091_c0_seq1 comp55952_c2_seq4 comp44717_c1_seq2 comp57311_c0_seq7 comp57588_c4_seq3 comp42968_c1_seq1 comp57771_c0_seq7 comp56345_c4_seq2 comp55444_c0_seq1 comp32643_c0_seq1 comp53472_c0_seq1 comp52147_c1_seq2 comp56518_c2_seq3 comp46353_c1_seq3 comp55569_c0_seq13 comp33098_c0_seq1 comp57263_c2_seq3 comp54082_c0_seq3 comp56325_c0_seq3 comp59264_c0_seq1 comp49511_c0_seq1 comp56977_c1_seq2 comp58163_c3_seq1 comp51221_c0_seq1 comp53120_c0_seq1 comp52347_c2_seq3 comp50472_c0_seq4 comp54534_c1_seq1 comp48255_c0_seq1 comp58008_c2_seq17 comp58684_c0_seq1 comp50306_c0_seq3 comp57430_c1_seq34 comp56361_c2_seq15 comp56488_c0_seq3 comp42490_c0_seq1 comp28506_c0_seq1 comp54304_c5_seq3 comp48269_c0_seq2 comp53664_c0_seq1 comp50260_c0_seq11 comp57598_c0_seq6 comp57935_c1_seq2 comp56914_c2_seq1 comp56986_c0_seq1 comp53527_c2_seq5 comp56626_c4_seq6 comp31954_c0_seq1 comp57166_c0_seq2 comp53395_c0_seq1 comp58213_c0_seq1 comp52523_c0_seq3 comp56988_c0_seq14 comp52864_c1_seq1 comp58237_c5_seq8 comp33794_c0_seq1 comp38589_c0_seq2 comp56788_c0_seq17 comp38148_c1_seq1 comp48511_c0_seq1 comp60219_c0_seq1 comp44712_c1_seq1 comp57996_c5_seq1 comp58196_c0_seq20 comp56938_c8_seq1 comp57816_c1_seq6 comp33937_c0_seq1 comp56223_c2_seq4 comp57398_c0_seq3 comp4780_c1_seq1 comp55953_c0_seq4 comp50013_c0_seq4 comp58031_c2_seq12 comp48394_c0_seq1 comp58253_c1_seq25 comp51389_c0_seq4 comp57621_c1_seq1 comp55480_c2_seq1 comp56891_c0_seq2 comp57461_c1_seq2 comp56840_c0_seq5 comp56346_c0_seq5 comp56578_c0_seq4 comp56378_c4_seq7 comp56780_c1_seq3 comp54466_c2_seq1 comp58080_c0_seq1 comp29342_c0_seq1 comp43861_c3_seq2 comp56322_c0_seq2 comp55883_c2_seq2 comp55670_c5_seq11 comp56659_c2_seq3 comp55902_c2_seq1 comp55995_c2_seq1 comp30574_c0_seq1 comp55650_c1_seq2 comp46651_c0_seq2 comp50029_c0_seq1 comp56749_c0_seq4 comp57419_c0_seq4 comp51540_c2_seq1 comp58287_c3_seq2 comp55392_c0_seq8 comp50762_c0_seq1 comp55866_c1_seq2 comp50559_c1_seq1 comp57996_c0_seq1 comp57369_c2_seq8 comp57824_c1_seq11 comp54685_c1_seq5 comp31593_c0_seq1 comp57969_c1_seq5 comp57153_c1_seq22 comp55190_c0_seq7 comp54539_c0_seq4 comp51636_c1_seq3 comp51185_c1_seq2 comp28313_c0_seq1 comp46951_c0_seq1 comp53020_c0_seq1 comp57961_c1_seq1 comp54155_c1_seq10 comp56396_c0_seq2 comp51164_c1_seq1 comp43461_c2_seq1 comp53716_c0_seq1 comp55510_c0_seq3 comp54958_c0_seq2 comp50777_c0_seq1 comp59243_c0_seq1 comp54957_c1_seq2 comp55604_c2_seq1 comp55488_c1_seq1 comp48903_c1_seq1 comp57191_c0_seq1 comp42903_c0_seq1 comp55856_c2_seq1 comp56737_c5_seq23 comp42904_c0_seq1 comp47502_c0_seq1 comp56898_c0_seq3 comp31925_c0_seq1 comp51680_c0_seq1 comp58096_c3_seq18 comp51192_c0_seq1 comp31229_c0_seq1 comp47698_c0_seq1 comp53452_c1_seq3 comp53401_c1_seq3 comp31558_c0_seq1 comp55452_c0_seq2 comp56666_c0_seq12 comp46183_c1_seq1 comp50083_c1_seq1 comp49837_c0_seq1 comp49893_c0_seq1 comp56481_c2_seq19 comp46592_c0_seq1 comp58176_c1_seq8 comp41705_c0_seq1 comp49957_c0_seq1 comp31511_c2_seq1 comp56940_c1_seq3 comp52659_c0_seq1 comp54277_c0_seq4 comp54779_c1_seq1 comp58150_c0_seq1 comp43475_c0_seq1 comp56598_c0_seq4 comp53887_c0_seq2 comp57953_c3_seq12 comp58030_c0_seq6 comp57865_c0_seq18 comp57137_c1_seq6 comp50140_c0_seq3 comp43802_c0_seq1 comp28823_c0_seq1 comp52096_c0_seq5 comp51777_c0_seq1 comp57951_c0_seq3 comp51463_c0_seq3 comp57760_c3_seq6 comp55739_c1_seq1 comp57395_c2_seq14 comp55070_c1_seq11 comp34328_c0_seq1 comp53783_c0_seq2 comp51984_c0_seq3 comp58025_c0_seq50 comp46401_c0_seq1 comp56773_c0_seq1 comp55538_c1_seq1 comp36702_c2_seq1 comp51300_c0_seq4 comp46041_c1_seq1 comp57573_c1_seq7 comp56754_c0_seq3 comp30435_c0_seq1 comp57129_c0_seq1 comp53953_c0_seq1 comp55547_c0_seq5 comp58283_c4_seq11 comp49447_c1_seq2 comp54667_c0_seq3 comp45342_c0_seq2 comp45610_c0_seq2 comp49132_c0_seq1 comp57261_c0_seq1 comp55628_c0_seq2 comp51681_c0_seq2 comp46847_c4_seq1 comp53891_c0_seq1 comp57623_c1_seq2 comp29663_c0_seq1 comp57038_c2_seq1 comp56877_c0_seq5 comp46537_c3_seq1 comp56856_c2_seq8 comp52301_c1_seq1 comp50960_c0_seq2 comp54927_c3_seq1 comp53475_c0_seq1 comp33182_c0_seq1 comp49227_c0_seq2 comp36938_c1_seq1 comp56107_c0_seq6 comp54080_c0_seq2 comp57295_c1_seq2 comp53721_c1_seq2 comp58154_c2_seq27 comp56631_c1_seq4 comp53263_c0_seq1 comp55615_c1_seq1 comp57099_c0_seq1 comp51825_c0_seq2 comp46205_c0_seq5 comp57916_c1_seq13 comp54841_c7_seq3 comp32927_c1_seq1 comp58138_c2_seq3 comp55445_c1_seq4 comp49846_c0_seq2 comp37529_c0_seq1 comp54908_c1_seq7 comp46892_c2_seq1 comp54087_c0_seq1 comp55479_c12_seq6 comp54722_c0_seq1 comp57120_c0_seq2 comp51153_c0_seq1 comp52074_c0_seq1 comp57185_c0_seq2 comp57369_c1_seq4 comp55646_c0_seq1 comp3034_c0_seq1 comp52601_c0_seq1 comp56052_c0_seq3 comp52209_c0_seq1 comp31946_c1_seq1 comp50952_c1_seq1 comp55878_c1_seq6 comp57004_c1_seq1 comp56737_c5_seq11 comp36597_c0_seq1 comp49433_c0_seq2 comp48784_c1_seq1 comp33857_c0_seq2 comp47656_c0_seq1 comp56345_c2_seq6 comp57224_c0_seq5 comp53109_c0_seq6 comp56874_c4_seq19 comp46128_c0_seq1 comp58176_c1_seq3 comp56158_c0_seq2 comp135740_c0_seq1 comp47870_c0_seq1 comp56175_c0_seq7 comp56726_c2_seq17 comp57807_c6_seq3 comp44379_c0_seq1 comp35993_c0_seq2 comp43000_c0_seq3 comp57159_c3_seq1 comp54167_c2_seq2 comp58051_c0_seq1 comp58001_c3_seq12 comp57878_c0_seq5 comp56075_c2_seq7 comp28593_c0_seq2 comp53443_c0_seq2 comp46185_c0_seq1 comp52196_c0_seq1 comp57667_c2_seq4 comp55495_c4_seq6 comp52987_c0_seq3 comp54445_c0_seq3 comp43415_c0_seq1 comp56935_c0_seq1 comp58304_c0_seq4 comp56092_c0_seq5 comp57285_c0_seq16 comp48487_c3_seq1 comp50576_c1_seq2 comp54608_c1_seq6 comp57766_c0_seq9 comp137511_c0_seq1 comp57188_c1_seq8 comp50563_c0_seq3 comp55975_c0_seq1 comp54309_c0_seq1 comp56736_c1_seq4 comp58016_c5_seq5 comp58140_c1_seq2 comp52291_c0_seq4 comp50384_c2_seq1 comp57539_c2_seq7 comp51541_c2_seq2 comp53241_c0_seq9 comp53904_c1_seq2 comp49720_c0_seq1 comp50453_c0_seq1 comp53743_c0_seq4 comp54466_c0_seq3 comp57430_c1_seq6 comp54748_c2_seq3 comp43683_c0_seq1 comp55862_c0_seq3 comp46323_c0_seq1 comp48238_c1_seq3 comp57973_c1_seq1 comp46076_c1_seq1 comp56434_c1_seq1 comp57725_c0_seq1 comp48728_c2_seq1 comp31630_c0_seq1 comp51342_c0_seq3 comp50454_c0_seq5 comp57638_c3_seq9 comp52244_c0_seq3 comp55726_c0_seq1 comp28774_c0_seq1 comp45761_c0_seq1 comp53795_c0_seq2 comp46127_c0_seq1 comp56509_c0_seq1 comp57988_c4_seq40 comp48916_c1_seq3 comp68632_c0_seq1 comp53740_c0_seq1 comp50754_c0_seq1 comp57649_c3_seq13 comp52048_c0_seq4 comp56433_c1_seq5 comp57077_c0_seq4 comp47115_c1_seq2 comp49219_c0_seq1 comp33785_c0_seq1 comp56752_c1_seq3 comp52164_c0_seq2 comp45806_c0_seq2 comp54580_c0_seq2 comp42685_c0_seq1 comp50898_c0_seq1 comp57995_c6_seq5 comp53937_c0_seq2 comp45734_c2_seq2 comp44298_c0_seq1 comp42594_c0_seq2 comp56876_c4_seq2 comp49638_c0_seq1 comp45106_c3_seq1 comp56641_c0_seq13 comp45015_c2_seq1 comp56931_c0_seq3 comp46831_c1_seq1 comp48041_c1_seq1 comp55987_c0_seq1 comp52813_c0_seq2 comp53272_c0_seq4 comp50103_c2_seq1 comp53291_c0_seq1 comp55495_c10_seq1 comp30684_c0_seq2 comp49641_c0_seq1 comp53700_c3_seq1 comp53577_c0_seq1 comp41685_c0_seq1 comp45761_c0_seq2 comp42739_c0_seq1 comp46534_c1_seq2 comp56858_c3_seq8 comp58945_c0_seq1 comp51554_c2_seq3 comp54851_c0_seq3 comp50057_c0_seq2 comp57844_c0_seq1 comp45078_c0_seq1 comp57817_c1_seq2 comp57137_c1_seq5 comp31303_c1_seq1 comp54871_c4_seq1 comp56437_c0_seq6 comp58061_c0_seq26 comp38102_c0_seq1 comp49685_c0_seq2 comp49410_c0_seq1 comp56124_c0_seq1 comp56341_c3_seq4 comp58118_c1_seq2 comp54461_c1_seq3 comp52142_c5_seq7 comp50327_c0_seq1 comp54012_c0_seq1 comp56881_c0_seq10 comp46992_c0_seq4 comp43237_c0_seq1 comp46382_c0_seq2 comp57711_c0_seq1 comp57467_c0_seq8 comp43036_c0_seq1 comp54682_c0_seq1 comp43215_c1_seq1 comp55614_c2_seq8 comp58050_c3_seq5 comp57748_c1_seq6 comp56708_c0_seq2 comp52708_c0_seq4 comp53311_c2_seq1 comp56878_c8_seq2 comp56444_c3_seq1 comp56922_c2_seq1 comp56175_c0_seq8 comp50175_c0_seq1 comp29915_c0_seq1 comp58119_c0_seq6 comp58216_c0_seq11 comp53021_c0_seq1 comp54874_c0_seq1 comp55513_c2_seq2 comp50697_c0_seq1 comp57385_c2_seq2 comp48143_c0_seq2 comp30216_c0_seq1 comp56608_c1_seq2 comp57906_c1_seq3 comp56272_c0_seq2 comp56536_c0_seq5 comp58249_c1_seq3 comp46132_c0_seq2 comp56848_c0_seq20 comp48150_c0_seq1 comp49720_c0_seq2 comp29909_c0_seq1 comp57434_c4_seq2 comp57531_c0_seq4 comp58287_c0_seq6 comp56987_c3_seq3 comp58182_c2_seq14 comp49264_c0_seq1 comp47873_c0_seq1 comp47998_c0_seq1 comp50856_c0_seq1 comp43012_c0_seq1 comp50695_c0_seq1 comp56485_c1_seq7 comp57520_c0_seq5 comp49233_c0_seq1 comp57160_c1_seq8 comp49030_c0_seq4 comp54406_c8_seq2 comp43034_c0_seq1 comp54732_c0_seq4 comp31328_c0_seq1 comp56709_c1_seq8 comp57631_c0_seq6 comp38311_c0_seq1 comp46081_c0_seq1 comp28533_c1_seq1 comp44347_c0_seq1 comp49802_c1_seq1 comp57837_c1_seq7 comp45951_c0_seq1 comp41906_c0_seq3 comp57897_c0_seq7 comp53340_c0_seq1 comp55491_c3_seq7 comp49670_c1_seq1 comp57672_c5_seq3 comp52297_c0_seq1 comp54725_c1_seq1 comp51508_c2_seq4 comp57461_c3_seq4 comp49187_c0_seq1 comp51267_c0_seq2 comp49567_c3_seq1 comp57784_c3_seq4 comp57398_c0_seq4 comp46118_c0_seq1 comp56068_c0_seq3 comp57431_c1_seq2 comp57096_c0_seq2 comp57932_c0_seq8 comp56869_c0_seq3 comp58062_c4_seq4 comp36878_c0_seq1 comp56306_c4_seq13 comp56638_c1_seq4 comp54974_c0_seq4 comp57346_c0_seq1 comp46567_c0_seq6 comp51370_c0_seq2 comp57933_c2_seq2 comp56766_c1_seq1 comp57851_c2_seq1 comp51896_c1_seq1 comp55372_c0_seq1 comp53659_c0_seq2 comp57468_c0_seq8 comp55443_c0_seq3 comp58152_c1_seq1 comp50870_c3_seq2 comp42321_c0_seq2 comp51100_c0_seq2 comp58205_c9_seq9 comp55376_c0_seq1 comp54149_c0_seq2 comp42288_c0_seq1 comp57445_c0_seq9 comp230668_c0_seq1 comp58031_c2_seq7 comp57257_c2_seq1 comp57861_c0_seq14 comp45013_c0_seq1 comp56724_c1_seq6 comp55101_c0_seq1 comp57868_c1_seq1 comp56305_c0_seq2 comp42033_c0_seq3 comp52189_c0_seq1 comp55815_c2_seq1 comp50655_c2_seq1 comp55741_c2_seq3 comp58183_c2_seq9 comp122363_c0_seq1 comp56303_c0_seq1 comp56218_c2_seq1 comp55297_c0_seq4 comp51982_c0_seq2 comp48422_c0_seq1 comp58289_c3_seq1 comp57630_c0_seq1 comp53037_c0_seq2 comp55049_c0_seq2 comp49451_c0_seq2 comp48890_c0_seq1 comp46084_c0_seq1 comp55765_c1_seq2 comp38420_c0_seq1 comp51555_c0_seq1 comp58168_c3_seq4 comp52942_c0_seq1 comp58001_c4_seq9 comp58001_c3_seq20 comp58040_c4_seq7 comp58491_c0_seq1 comp41604_c0_seq1 comp57384_c2_seq5 comp52906_c0_seq1 comp54405_c0_seq5 comp55818_c3_seq1 comp57438_c3_seq4 comp57934_c1_seq3 comp52972_c0_seq1 comp57391_c0_seq9 comp57626_c8_seq12 comp48437_c0_seq1 comp46260_c0_seq1 comp51144_c0_seq2 comp55028_c0_seq1 comp54316_c2_seq3 comp48794_c3_seq1 comp42648_c0_seq1 comp57073_c5_seq17 comp48605_c0_seq3 comp49590_c0_seq2 comp56726_c2_seq4 comp34185_c0_seq1 comp55910_c1_seq1 comp46993_c0_seq2 comp54685_c1_seq3 comp51133_c0_seq1 comp34526_c0_seq1 comp31237_c0_seq2 comp54572_c1_seq3 comp57649_c3_seq9 comp56212_c0_seq5 comp45842_c0_seq2 comp54896_c1_seq1 comp42285_c0_seq1 comp58021_c3_seq17 comp54599_c0_seq1 comp50723_c0_seq2 comp54525_c3_seq1 comp55798_c1_seq3 comp48167_c0_seq1 comp57759_c5_seq1 comp57544_c0_seq5 comp45791_c1_seq1 comp53908_c0_seq1 comp50806_c0_seq1 comp53329_c1_seq1 comp49139_c2_seq1 comp50853_c0_seq2 comp53133_c1_seq6 comp39033_c0_seq1 comp51808_c0_seq2 comp56056_c0_seq3 comp54614_c0_seq2 comp57033_c1_seq1 comp54539_c0_seq3 comp53605_c0_seq5 comp51583_c0_seq3 comp53285_c2_seq2 comp51015_c1_seq8 comp46761_c1_seq1 comp47667_c0_seq1 comp49625_c0_seq2 comp56346_c0_seq21 comp46735_c0_seq5 comp41342_c0_seq1 comp57048_c1_seq2 comp53431_c2_seq6 comp50422_c0_seq2 comp51097_c2_seq2 comp52585_c0_seq5 comp49498_c0_seq1 comp57882_c1_seq1 comp47303_c0_seq1 comp48429_c0_seq1 comp55735_c0_seq7 comp54942_c1_seq10 comp52047_c0_seq1 comp56395_c12_seq18 comp56595_c0_seq11 comp53829_c0_seq4 comp55168_c3_seq3 comp56625_c1_seq1 comp56135_c0_seq6 comp32203_c0_seq1 comp47642_c0_seq1 comp44500_c1_seq1 comp49199_c1_seq2 comp48728_c1_seq1 comp56742_c0_seq3 comp43862_c1_seq1 comp54686_c0_seq5 comp57070_c1_seq7 comp60234_c0_seq1 comp44156_c2_seq1 comp264454_c0_seq1 comp58099_c0_seq8 comp50506_c0_seq2 comp54142_c0_seq3 comp42862_c0_seq1 comp56576_c1_seq11 comp55420_c0_seq3 comp57879_c0_seq11 comp58053_c2_seq9 comp52868_c1_seq1 comp54384_c0_seq1 comp57306_c2_seq1 comp55312_c0_seq24 comp57129_c5_seq1 comp49982_c1_seq1 comp46647_c0_seq4 comp58044_c1_seq4 comp36030_c0_seq1 comp50438_c0_seq1 comp43712_c0_seq1 comp56494_c0_seq3 comp46036_c1_seq1 comp37751_c1_seq1 comp50544_c0_seq1 comp46139_c0_seq1 comp54268_c4_seq2 comp57629_c3_seq20 comp57318_c0_seq7 comp53217_c0_seq3 comp51298_c2_seq1 comp50238_c0_seq1 comp50956_c0_seq1 comp32630_c0_seq2 comp54304_c5_seq2 comp50301_c0_seq1 comp52985_c0_seq3 comp56344_c0_seq1 comp50851_c0_seq1 comp52936_c1_seq3 comp46093_c1_seq1 comp51306_c0_seq1 comp54994_c0_seq10 comp52656_c3_seq1 comp55145_c0_seq2 comp51452_c0_seq1 comp53392_c0_seq1 comp56156_c0_seq2 comp55578_c1_seq3 comp44717_c0_seq1 comp54843_c3_seq6 comp54031_c0_seq1 comp58290_c0_seq28 comp49303_c1_seq1 comp54942_c1_seq9 comp47115_c1_seq1 comp43862_c2_seq1 comp52768_c0_seq3 comp58100_c1_seq1 comp57883_c0_seq12 comp36340_c0_seq1 comp55415_c1_seq12 comp55227_c1_seq2 comp58124_c1_seq4 comp33182_c0_seq3 comp51655_c0_seq1 comp28704_c0_seq1 comp54525_c0_seq3 comp57758_c2_seq2 comp58170_c1_seq15 comp49994_c0_seq4 comp58261_c4_seq22 comp54016_c0_seq7 comp55270_c0_seq3 comp54021_c1_seq2 comp47637_c1_seq3 comp54958_c0_seq3 comp55889_c0_seq4 comp53610_c0_seq1 comp57478_c0_seq4 comp31401_c0_seq1 comp52757_c2_seq3 comp57605_c2_seq21 comp43634_c1_seq1 comp55682_c0_seq7 comp58202_c2_seq13 comp30965_c0_seq1 comp51573_c2_seq1 comp46699_c1_seq1 comp51579_c1_seq2 comp55260_c1_seq5 comp51040_c0_seq1 comp57393_c1_seq2 comp53387_c1_seq1 comp58227_c1_seq3 comp53206_c0_seq2 comp28067_c0_seq1 comp59999_c0_seq1 comp56044_c0_seq2 comp55587_c0_seq9 comp45150_c0_seq1 comp55423_c1_seq10 comp54863_c0_seq3 comp50490_c0_seq1 comp56639_c0_seq2 comp53145_c4_seq1 comp46077_c0_seq2 comp57397_c9_seq13 comp48786_c1_seq1 comp55212_c0_seq1 comp56626_c4_seq7 comp49482_c0_seq3 comp47169_c1_seq1 comp52069_c1_seq1 comp53747_c0_seq1 comp52324_c1_seq1 comp57407_c1_seq21 comp50524_c0_seq1 comp55343_c1_seq4 comp56883_c0_seq7 comp47134_c0_seq1 comp53941_c2_seq1 comp46563_c1_seq1 comp55356_c0_seq9 comp47411_c3_seq1 comp46527_c0_seq1 comp51400_c1_seq5 comp51958_c2_seq1 comp30300_c0_seq1 comp55479_c12_seq14 comp53490_c3_seq1 comp57594_c4_seq2 comp55639_c0_seq8 comp56111_c1_seq6 comp57232_c0_seq1 comp51325_c1_seq1 comp54171_c1_seq9 comp57806_c0_seq8 comp32470_c0_seq1 comp49593_c1_seq2 comp58166_c1_seq8 comp51987_c0_seq2 comp47581_c2_seq1 comp55895_c0_seq9 comp54262_c0_seq1 comp54127_c0_seq6 comp57675_c3_seq20 comp52157_c0_seq4 comp57263_c7_seq1 comp56683_c1_seq3 comp52493_c3_seq2 comp40478_c1_seq1 comp53215_c0_seq1 comp57458_c2_seq9 comp49846_c0_seq3 comp50122_c0_seq1 comp57369_c2_seq4 comp47773_c0_seq2 comp48607_c1_seq1 comp56271_c0_seq4 comp57247_c0_seq3 comp51013_c1_seq1 comp52313_c1_seq1 comp54346_c1_seq3 comp55412_c0_seq4 comp56083_c0_seq4 comp54843_c2_seq1 comp54632_c0_seq4 comp53098_c0_seq1 comp54634_c0_seq1 comp52508_c0_seq2 comp51363_c0_seq4 comp54660_c3_seq4 comp31733_c0_seq1 comp51749_c0_seq1 comp57930_c0_seq5 comp57811_c1_seq1 comp57357_c0_seq1 comp53229_c0_seq5 comp53591_c0_seq4 comp47910_c0_seq1 comp56984_c0_seq2 comp41880_c2_seq1 comp54589_c0_seq1 comp53650_c4_seq2 comp55831_c18_seq1 comp47613_c1_seq1 comp53518_c0_seq4 comp56961_c3_seq19 comp55476_c0_seq2 comp55268_c1_seq4 comp57241_c2_seq14 comp57257_c1_seq1 comp51798_c0_seq2 comp49664_c0_seq1 comp32556_c0_seq1 comp55713_c1_seq1 comp55031_c0_seq1 comp58181_c0_seq2 comp54855_c0_seq1 comp30140_c0_seq1 comp56497_c1_seq8 comp56152_c0_seq2 comp47807_c0_seq1 comp54257_c0_seq1 comp54697_c0_seq4 comp56578_c2_seq1 comp57837_c1_seq16 comp57680_c0_seq17 comp51221_c2_seq2 comp51216_c1_seq2 comp57581_c0_seq2 comp48554_c0_seq1 comp54267_c0_seq3 comp55763_c0_seq5 comp56585_c1_seq2 comp49995_c0_seq1 comp53739_c0_seq1 comp56296_c0_seq3 comp48616_c0_seq4 comp52932_c0_seq1 comp44095_c0_seq1 comp50157_c0_seq1 comp57583_c0_seq5 comp54628_c0_seq2 comp55341_c2_seq1 comp56106_c4_seq1 comp57188_c1_seq11 comp55503_c6_seq6 comp56652_c0_seq1 comp53914_c3_seq24 comp56750_c1_seq3 comp47590_c0_seq1 comp51327_c1_seq1 comp48978_c0_seq2 comp56860_c2_seq1 comp50948_c0_seq8 comp48770_c0_seq4 comp50975_c0_seq1 comp52622_c0_seq2 comp56688_c3_seq1 comp57485_c0_seq1 comp58275_c4_seq15 comp56192_c4_seq16 comp44301_c2_seq1 comp51228_c2_seq3 comp48313_c3_seq4 comp55078_c1_seq2 comp55492_c4_seq2 comp53224_c2_seq1 comp55747_c1_seq14 comp57579_c2_seq4 comp56341_c3_seq15 comp56915_c1_seq2 comp56845_c0_seq7 comp49411_c1_seq1 comp49011_c1_seq1 comp56191_c0_seq1 comp47054_c0_seq2 comp45834_c0_seq1 comp54401_c8_seq22 comp47424_c0_seq1 comp54771_c0_seq1 comp28293_c0_seq1 comp47393_c0_seq1 comp57831_c5_seq1 comp51086_c0_seq1 comp55195_c4_seq3 comp56043_c0_seq6 comp49532_c0_seq1 comp53878_c3_seq1 comp29826_c0_seq1 comp57327_c2_seq5 comp53945_c0_seq3 comp55363_c1_seq3 comp56630_c2_seq1 comp55894_c0_seq1 comp57454_c6_seq2 comp57634_c3_seq11 comp57197_c1_seq5 comp55244_c2_seq1 comp48009_c3_seq1 comp57369_c1_seq8 comp54109_c0_seq2 comp57537_c0_seq16 comp28773_c0_seq1 comp49155_c4_seq1 comp57140_c5_seq4 comp56755_c0_seq4 comp58200_c2_seq17 comp54352_c0_seq1 comp58040_c3_seq3 comp49873_c2_seq1 comp44098_c0_seq1 comp54458_c7_seq3 comp58037_c4_seq9 comp56109_c2_seq5 comp56934_c0_seq7 comp47222_c0_seq1 comp55689_c1_seq14 comp42877_c0_seq2 comp53839_c0_seq1 comp56470_c1_seq4 comp55685_c0_seq1 comp50720_c0_seq3 comp52622_c0_seq11 comp43525_c0_seq1 comp57387_c0_seq2 comp41860_c0_seq1 comp27703_c0_seq1 comp31243_c0_seq1 comp57194_c1_seq4 comp57588_c11_seq2 comp56798_c1_seq4 comp56685_c0_seq2 comp57298_c4_seq1 comp57445_c0_seq11 comp31902_c0_seq1 comp51278_c0_seq2 comp57601_c0_seq4 comp57236_c1_seq1 comp56404_c0_seq13 comp54812_c0_seq1 comp55817_c1_seq7 comp57210_c2_seq4 comp56719_c0_seq4 comp52789_c0_seq3 comp55999_c0_seq2 comp32891_c1_seq1 comp42469_c0_seq1 comp58049_c0_seq13 comp57817_c1_seq3 comp55823_c0_seq4 comp30354_c0_seq1 comp44413_c0_seq1 comp56714_c3_seq11 comp55640_c7_seq2 comp31122_c0_seq1 comp31583_c0_seq2 comp59375_c0_seq1 comp54350_c0_seq2 comp55723_c2_seq4 comp50499_c3_seq1 comp51140_c0_seq1 comp50710_c0_seq1 comp49158_c0_seq1 comp55204_c1_seq1 comp51549_c0_seq2 comp55947_c1_seq1 comp55576_c2_seq3 comp56543_c0_seq2 comp53935_c0_seq1 comp53445_c0_seq1 comp32634_c0_seq2 comp46688_c0_seq2 comp29445_c0_seq1 comp54866_c0_seq3 comp47455_c4_seq1 comp56048_c0_seq1 comp4735_c0_seq1 comp58176_c1_seq7 comp56075_c0_seq2 comp40935_c1_seq1 comp53049_c1_seq3 comp43961_c0_seq1 comp52972_c0_seq5 comp57585_c0_seq2 comp47363_c0_seq1 comp43724_c0_seq1 comp54574_c0_seq2 comp49881_c0_seq1 comp46626_c0_seq1 comp57296_c0_seq30 comp50230_c0_seq1 comp45388_c0_seq1 comp56444_c5_seq6 comp49035_c0_seq1 comp55985_c3_seq1 comp53791_c0_seq1 comp57849_c2_seq12 comp57475_c1_seq3 comp50982_c0_seq2 comp55878_c5_seq1 comp55409_c0_seq7 comp47800_c0_seq1 comp52912_c1_seq2 comp53787_c1_seq1 comp55065_c0_seq3 comp54062_c0_seq4 comp55398_c2_seq3 comp57776_c0_seq2 comp29168_c0_seq1 comp54010_c0_seq2 comp53177_c0_seq5 comp48243_c0_seq2 comp56570_c0_seq2 comp56557_c0_seq1 comp57073_c5_seq4 comp46914_c0_seq1 comp58288_c0_seq7 comp46372_c0_seq1 comp52760_c0_seq1 comp51834_c0_seq2 comp44454_c1_seq1 comp54207_c0_seq5 comp57808_c2_seq2 comp57900_c3_seq1 comp29452_c0_seq1 comp101468_c0_seq1 comp48599_c1_seq1 comp54029_c0_seq4 comp43914_c1_seq1 comp61438_c0_seq1 comp53783_c0_seq3 comp57525_c0_seq4 comp57857_c1_seq25 comp57077_c0_seq7 comp54058_c1_seq1 comp50275_c0_seq2 comp55655_c0_seq1 comp55548_c1_seq13 comp57894_c0_seq11 comp52990_c0_seq1 comp48661_c0_seq2 comp53473_c0_seq1 comp42632_c0_seq1 comp53730_c1_seq2 comp52375_c2_seq1 comp45953_c1_seq2 comp55744_c8_seq26 comp53931_c0_seq1 comp57909_c0_seq11 comp52492_c0_seq3 comp51187_c1_seq2 comp58103_c3_seq1 comp57689_c2_seq18 comp50785_c0_seq4 comp55710_c0_seq7 comp56462_c1_seq5 comp53490_c1_seq4 comp48080_c0_seq1 comp57766_c0_seq8 comp57695_c1_seq2 comp58105_c1_seq38 comp57997_c3_seq2 comp54909_c1_seq6 comp55314_c0_seq6 comp49191_c0_seq2 comp45352_c0_seq1 comp57927_c3_seq5 comp56255_c7_seq5 comp48844_c0_seq3 comp57264_c2_seq1 comp47393_c1_seq1 comp40219_c0_seq1 comp57968_c0_seq2 comp58024_c0_seq1 comp46426_c0_seq2 comp49390_c1_seq1 comp51667_c3_seq3 comp52810_c0_seq2 comp49494_c0_seq1 comp51678_c0_seq1 comp52030_c1_seq4 comp47679_c0_seq1 comp54842_c0_seq3 comp55873_c0_seq5 comp56719_c0_seq3 comp54717_c0_seq3 comp57897_c0_seq6 comp58819_c0_seq1 comp54756_c0_seq1 comp51939_c0_seq2 comp58232_c1_seq1 comp27800_c0_seq1 comp29217_c0_seq1 comp30941_c0_seq2 comp55833_c0_seq1 comp46166_c2_seq1 comp45977_c0_seq1 comp60847_c0_seq1 comp52623_c0_seq1 comp42405_c0_seq1 comp49020_c0_seq2 comp55181_c0_seq10 comp48974_c0_seq1 comp29247_c0_seq1 comp54526_c0_seq3 comp46831_c3_seq2 comp57743_c5_seq1 comp53482_c0_seq3 comp32526_c0_seq2 comp50179_c3_seq2 comp58182_c2_seq1 comp54817_c10_seq1 comp52628_c0_seq1 comp43853_c1_seq1 comp51653_c0_seq1 comp31643_c0_seq1 comp56659_c2_seq8 comp56079_c4_seq1 comp48111_c0_seq1 comp48441_c0_seq2 comp45385_c0_seq1 comp55330_c0_seq2 comp50538_c0_seq1 comp57792_c0_seq8 comp49077_c0_seq1 comp53383_c1_seq2 comp43594_c0_seq1 comp55506_c3_seq2 comp51788_c0_seq1 comp51844_c1_seq1 comp49333_c0_seq2 comp56037_c0_seq1 comp47872_c0_seq1 comp53905_c0_seq2 comp55242_c1_seq1 comp57332_c2_seq2 comp58198_c4_seq11 comp55690_c0_seq4 comp46024_c0_seq1 comp48914_c0_seq4 comp53036_c0_seq1 comp54111_c2_seq1 comp58440_c0_seq1 comp56260_c0_seq8 comp36121_c2_seq1 comp47464_c0_seq1 comp50426_c0_seq1 comp31563_c0_seq3 comp55995_c10_seq3 comp54153_c0_seq2 comp56284_c1_seq1 comp57473_c0_seq24 comp57546_c3_seq16 comp57743_c4_seq2 comp55461_c2_seq7 comp57123_c0_seq8 comp49011_c0_seq1 comp53149_c0_seq2 comp49308_c0_seq1 comp31218_c0_seq1 comp54298_c0_seq2 comp51679_c4_seq4 comp54721_c0_seq9 comp55392_c0_seq6 comp54710_c0_seq7 comp54573_c2_seq2 comp43624_c1_seq1 comp58161_c0_seq4 comp54863_c0_seq7 comp56324_c2_seq1 comp54342_c0_seq2 comp42560_c0_seq1 comp47429_c0_seq1 comp49608_c1_seq3 comp52627_c0_seq1 comp55251_c1_seq1 comp47685_c0_seq1 comp51029_c0_seq2 comp57839_c7_seq10 comp57748_c2_seq5 comp50919_c0_seq1 comp59211_c0_seq1 comp57499_c6_seq16 comp56834_c3_seq4 comp54509_c2_seq1 comp50087_c0_seq2 comp56141_c2_seq18 comp51967_c0_seq1 comp54831_c0_seq2 comp52090_c0_seq2 comp42653_c0_seq2 comp56742_c0_seq7 comp57907_c11_seq6 comp45399_c0_seq4 comp29292_c0_seq1 comp43507_c0_seq1 comp55979_c0_seq1 comp50215_c0_seq1 comp57152_c4_seq47 comp48683_c1_seq1 comp53671_c1_seq1 comp55230_c1_seq7 comp58128_c3_seq6 comp50307_c0_seq1 comp53526_c0_seq1 comp30814_c0_seq2 comp54796_c0_seq2 comp52368_c0_seq2 comp48140_c0_seq3 comp57425_c1_seq3 comp56877_c0_seq9 comp56036_c0_seq6 comp40833_c0_seq1 comp55502_c0_seq6 comp55373_c0_seq1 comp53703_c1_seq1 comp57221_c0_seq6 comp58183_c1_seq3 comp43633_c0_seq1 comp56178_c3_seq1 comp53565_c3_seq1 comp54154_c1_seq1 comp49275_c1_seq2 comp57865_c0_seq6 comp48443_c0_seq5 comp49174_c1_seq3 comp47313_c0_seq5 comp33615_c1_seq1 comp29489_c0_seq1 comp51742_c0_seq1 comp52800_c1_seq4 comp56252_c0_seq1 comp54025_c0_seq1 comp54592_c0_seq4 comp51725_c0_seq1 comp4116_c0_seq1 comp54232_c0_seq2 comp58160_c1_seq3 comp56005_c2_seq1 comp50120_c0_seq4 comp55663_c9_seq15 comp56758_c5_seq10 comp48982_c0_seq1 comp56726_c2_seq11 comp55186_c4_seq1 comp50447_c0_seq2 comp54304_c3_seq1 comp27895_c0_seq1 comp54676_c0_seq1 comp56964_c3_seq5 comp54770_c0_seq2 comp46492_c0_seq1 comp41539_c1_seq1 comp56258_c0_seq2 comp56230_c14_seq12 comp58194_c0_seq1 comp54668_c0_seq4 comp57676_c0_seq2 comp53132_c2_seq1 comp52879_c1_seq1 comp51413_c1_seq2 comp41877_c0_seq1 comp52331_c0_seq1 comp51459_c1_seq3 comp56436_c0_seq7 comp50352_c0_seq3 comp56944_c0_seq7 comp56096_c2_seq3 comp55120_c0_seq1 comp53802_c0_seq2 comp55976_c1_seq9 comp30127_c0_seq1 comp55946_c0_seq5 comp46661_c0_seq2 comp58257_c0_seq3 comp49489_c2_seq1 comp50052_c0_seq1 comp57679_c1_seq6 comp54158_c0_seq2 comp50030_c0_seq5 comp52097_c0_seq1 comp52360_c0_seq1 comp53955_c2_seq2 comp57803_c0_seq14 comp52609_c0_seq1 comp53406_c1_seq1 comp55825_c0_seq1 comp46571_c0_seq1 comp49109_c0_seq2 comp57410_c1_seq3 comp53925_c0_seq4 comp47546_c0_seq1 comp31093_c0_seq2 comp49296_c1_seq1 comp56883_c0_seq2 comp56804_c1_seq6 comp57639_c1_seq8 comp52287_c0_seq1 comp57801_c0_seq3 comp51576_c1_seq5 comp56695_c0_seq2 comp54438_c0_seq1 comp57539_c1_seq3 comp54398_c0_seq7 comp57450_c1_seq2 comp56697_c0_seq5 comp56434_c2_seq1 comp51156_c0_seq2 comp29757_c0_seq1 comp56725_c2_seq2 comp55923_c0_seq6 comp57468_c0_seq17 comp58016_c3_seq3 comp214123_c0_seq1 comp58243_c0_seq15 comp49569_c0_seq1 comp54673_c0_seq3 comp56234_c0_seq6 comp55437_c0_seq1 comp48502_c0_seq1 comp48199_c1_seq1 comp29472_c0_seq1 comp57896_c5_seq3 comp62208_c0_seq1 comp37760_c0_seq1 comp56275_c0_seq1 comp56622_c5_seq1 comp55852_c0_seq2 comp56460_c0_seq1 comp53127_c1_seq1 comp55555_c1_seq3 comp51190_c0_seq1 comp55791_c1_seq2 comp53082_c0_seq2 comp53933_c0_seq1 comp32778_c0_seq1 comp56562_c1_seq4 comp48236_c5_seq1 comp54475_c0_seq2 comp57026_c0_seq2 comp57994_c0_seq1 comp29623_c0_seq1 comp46640_c1_seq1 comp48593_c0_seq3 comp54447_c1_seq1 comp57845_c0_seq23 comp56924_c2_seq17 comp49238_c0_seq1 comp53281_c0_seq1 comp45729_c0_seq1 comp56595_c0_seq7 comp52668_c4_seq1 comp56340_c0_seq2 comp56859_c1_seq16 comp48650_c2_seq2 comp54570_c0_seq2 comp58018_c0_seq4 comp57124_c3_seq1 comp50914_c0_seq1 comp30208_c0_seq1 comp53113_c0_seq1 comp49037_c0_seq1 comp50523_c0_seq1 comp36631_c1_seq1 comp48354_c0_seq1 comp50293_c0_seq3 comp49819_c1_seq3 comp53403_c0_seq6 comp57369_c2_seq3 comp57430_c1_seq38 comp57372_c1_seq9 comp58585_c0_seq1 comp42240_c0_seq2 comp56065_c3_seq1 comp57152_c4_seq61 comp57232_c1_seq9 comp53974_c0_seq1 comp55980_c1_seq4 comp58147_c2_seq7 comp56489_c0_seq3 comp58297_c0_seq81 comp52835_c1_seq7 comp56243_c2_seq10 comp46638_c0_seq1 comp48017_c2_seq1 comp33883_c0_seq1 comp54357_c0_seq4 comp53973_c0_seq4 comp47907_c0_seq1 comp51279_c0_seq1 comp54193_c6_seq1 comp47744_c2_seq1 comp50642_c0_seq1 comp51787_c0_seq2 comp57895_c2_seq2 comp51597_c0_seq1 comp51223_c0_seq2 comp29953_c0_seq2 comp55050_c0_seq1 comp53085_c0_seq1 comp43196_c1_seq1 comp48486_c2_seq1 comp48463_c0_seq1 comp58017_c1_seq1 comp47655_c0_seq3 comp55298_c0_seq1 comp30033_c0_seq1 comp46127_c0_seq2 comp46820_c0_seq2 comp50904_c0_seq1 comp50605_c2_seq2 comp56374_c0_seq7 comp54323_c1_seq1 comp58021_c3_seq8 comp46714_c0_seq2 comp56541_c0_seq6 comp50360_c0_seq1 comp57098_c3_seq5 comp56031_c1_seq3 comp55784_c0_seq9 comp29255_c0_seq1 comp54600_c0_seq2 comp56670_c0_seq1 comp54409_c2_seq1 comp32435_c0_seq1 comp52905_c0_seq1 comp40446_c0_seq1 comp51892_c1_seq4 comp55892_c0_seq14 comp57291_c4_seq3 comp46886_c1_seq3 comp57324_c1_seq16 comp53697_c2_seq2 comp32224_c0_seq1 comp46628_c0_seq1 comp49903_c0_seq1 comp46604_c0_seq1 comp56540_c0_seq17 comp56875_c0_seq5 comp54157_c10_seq1 comp49750_c1_seq8 comp56069_c1_seq1 comp50031_c0_seq2 comp53680_c0_seq3 comp53194_c0_seq5 comp41541_c0_seq1 comp53711_c0_seq3 comp48410_c1_seq2 comp48022_c0_seq2 comp34087_c1_seq3 comp56478_c0_seq2 comp52529_c0_seq1 comp43556_c0_seq1 comp52042_c0_seq1 comp31641_c0_seq1 comp54651_c0_seq2 comp56602_c0_seq1 comp54205_c1_seq3 comp58081_c1_seq6 comp56998_c3_seq4 comp31178_c0_seq1 comp57694_c7_seq1 comp52081_c0_seq4 comp58130_c0_seq2 comp51925_c1_seq2 comp52089_c1_seq2 comp52911_c1_seq1 comp43465_c0_seq1 comp29533_c0_seq2 comp58216_c0_seq8 comp57751_c0_seq2 comp53187_c0_seq3 comp56202_c0_seq2 comp54178_c1_seq1 comp55860_c0_seq8 comp39332_c0_seq3 comp52823_c0_seq2 comp57632_c6_seq1 comp57476_c2_seq4 comp56578_c2_seq8 comp30108_c0_seq1 comp52079_c1_seq1 comp46659_c0_seq1 comp55087_c0_seq6 comp53888_c0_seq3 comp32251_c1_seq1 comp48435_c1_seq1 comp58082_c1_seq2 comp56857_c2_seq3 comp46202_c0_seq1 comp51872_c1_seq1 comp54619_c1_seq2 comp49885_c1_seq1 comp58139_c1_seq7 comp48248_c0_seq1 comp43531_c0_seq1 comp53490_c4_seq1 comp58299_c3_seq7 comp54417_c0_seq2 comp56900_c0_seq3 comp50316_c0_seq1 comp56104_c3_seq6 comp58138_c2_seq8 comp57046_c1_seq1 comp55129_c1_seq3 comp49985_c0_seq1 comp31846_c0_seq1 comp58272_c2_seq7 comp55473_c0_seq9 comp43225_c0_seq2 comp48953_c0_seq3 comp57604_c0_seq10 comp55886_c7_seq1 comp51818_c0_seq2 comp58253_c1_seq10 comp56665_c1_seq5 comp52682_c0_seq1 comp29610_c0_seq1 comp57407_c5_seq3 comp55465_c1_seq2 comp55192_c9_seq6 comp54075_c0_seq1 comp57277_c0_seq1 comp46778_c0_seq1 comp57216_c0_seq7 comp53838_c0_seq1 comp56249_c0_seq5 comp56929_c1_seq5 comp56599_c0_seq2 comp51195_c1_seq1 comp53770_c0_seq6 comp56172_c1_seq1 comp52521_c0_seq5 comp71581_c0_seq1 comp30738_c0_seq2 comp56675_c0_seq7 comp56665_c1_seq9 comp50144_c0_seq4 comp50054_c0_seq3 comp47783_c2_seq1 comp28901_c0_seq1 comp48993_c0_seq2 comp57525_c0_seq11 comp52926_c0_seq1 comp51646_c0_seq5 comp58292_c0_seq21 comp56481_c2_seq4 comp50198_c0_seq3 comp54785_c1_seq6 comp48928_c0_seq1 comp52681_c0_seq2 comp166080_c0_seq1 comp55454_c1_seq4 comp57800_c0_seq4 comp57672_c1_seq1 comp48871_c0_seq1 comp57431_c4_seq9 comp55900_c3_seq1 comp28809_c0_seq1 comp57791_c0_seq6 comp55183_c0_seq2 comp57687_c1_seq8 comp57722_c0_seq2 comp44376_c0_seq1 comp48000_c0_seq1 comp55214_c0_seq1 comp57630_c0_seq4 comp52031_c3_seq3 comp54195_c0_seq1 comp49768_c0_seq4 comp48130_c0_seq1 comp31740_c0_seq1 comp49699_c0_seq3 comp49045_c0_seq2 comp52216_c0_seq1 comp44022_c0_seq1 comp54339_c2_seq1 comp55747_c1_seq2 comp54345_c3_seq1 comp57461_c3_seq1 comp57831_c1_seq18 comp44771_c0_seq1 comp58142_c1_seq3 comp54734_c0_seq6 comp58235_c0_seq5 comp41694_c0_seq1 comp54120_c6_seq2 comp45947_c1_seq1 comp57241_c2_seq8 comp58283_c4_seq10 comp53232_c0_seq1 comp55644_c0_seq3 comp41530_c0_seq1 comp46335_c0_seq3 comp48460_c0_seq1 comp57687_c1_seq1 comp55670_c0_seq2 comp55140_c0_seq1 comp54279_c0_seq6 comp58158_c3_seq1 comp52745_c0_seq1 comp56987_c9_seq7 comp54138_c1_seq4 comp57680_c0_seq7 comp56047_c3_seq5 comp56987_c6_seq1 comp56654_c2_seq12 comp56975_c0_seq1 comp31932_c0_seq2 comp52663_c0_seq5 comp55754_c2_seq1 comp54111_c1_seq1 comp56589_c1_seq2 comp52242_c0_seq1 comp146245_c0_seq1 comp55924_c0_seq1 comp52849_c0_seq1 comp56361_c2_seq8 comp44858_c2_seq2 comp57538_c0_seq5 comp55616_c0_seq7 comp53664_c0_seq2 comp52899_c0_seq1 comp55850_c0_seq1 comp56981_c0_seq5 comp48351_c0_seq1 comp50160_c0_seq2 comp56328_c0_seq2 comp29296_c0_seq1 comp57442_c1_seq6 comp50543_c0_seq2 comp55359_c1_seq1 comp51801_c1_seq4 comp43980_c0_seq2 comp54464_c0_seq2 comp33532_c0_seq1 comp57234_c0_seq1 comp50610_c0_seq4 comp46121_c0_seq1 comp54027_c0_seq3 comp56383_c0_seq2 comp55817_c1_seq4 comp57210_c2_seq3 comp55798_c1_seq1 comp56028_c1_seq1 comp54892_c0_seq1 comp31378_c0_seq1 comp42949_c0_seq1 comp53491_c0_seq1 comp54286_c0_seq2 comp55153_c3_seq2 comp64894_c0_seq1 comp58141_c1_seq3 comp53556_c1_seq3 comp48248_c1_seq1 comp55585_c1_seq4 comp56060_c1_seq9 comp51752_c1_seq2 comp53856_c0_seq2 comp54630_c1_seq1 comp55107_c0_seq4 comp53354_c1_seq2 comp51889_c2_seq1 comp52438_c0_seq3 comp53054_c0_seq1 comp50942_c2_seq1 comp56395_c1_seq1 comp56716_c7_seq1 comp32437_c0_seq1 comp52770_c0_seq1 comp58188_c3_seq84 comp48117_c2_seq1 comp32166_c0_seq1 comp57356_c0_seq1 comp56048_c0_seq5 comp31650_c0_seq1 comp55401_c0_seq7 comp58198_c4_seq15 comp57830_c5_seq1 comp51208_c0_seq1 comp51920_c0_seq2 comp56559_c0_seq5 comp50560_c1_seq2 comp56251_c0_seq1 comp53417_c1_seq1 comp54387_c0_seq2 comp48487_c0_seq1 comp51003_c0_seq1 comp30446_c0_seq1 comp116513_c0_seq1 comp48756_c0_seq1 comp57753_c3_seq7 comp54746_c0_seq1 comp57977_c0_seq4 comp57614_c0_seq10 comp55815_c2_seq2 comp53139_c1_seq2 comp49446_c0_seq1 comp95836_c0_seq1 comp46061_c2_seq1 comp49334_c3_seq1 comp53547_c0_seq1 comp37991_c0_seq1 comp48530_c0_seq1 comp56674_c1_seq1 comp57117_c0_seq4 comp57500_c2_seq3 comp56817_c4_seq1 comp41479_c3_seq1 comp50582_c1_seq4 comp52357_c1_seq1 comp55809_c1_seq14 comp48834_c2_seq3 comp56721_c0_seq2 comp60991_c0_seq1 comp50176_c0_seq1 comp56021_c3_seq3 comp42962_c0_seq1 comp56146_c0_seq2 comp52973_c2_seq1 comp51513_c0_seq6 comp56688_c2_seq23 comp54046_c0_seq1 comp52841_c0_seq2 comp51497_c0_seq1 comp57030_c2_seq1 comp55270_c0_seq4 comp56575_c3_seq2 comp55116_c0_seq1 comp51986_c1_seq1 comp45804_c1_seq1 comp56096_c0_seq1 comp53752_c1_seq3 comp53776_c1_seq2 comp57090_c2_seq2 comp52833_c0_seq14 comp54361_c0_seq5 comp55514_c1_seq5 comp46115_c1_seq1 comp51957_c0_seq1 comp46070_c0_seq2 comp58086_c1_seq16 comp54376_c0_seq1 comp50064_c4_seq1 comp56245_c1_seq1 comp50785_c0_seq1 comp56780_c0_seq2 comp56307_c2_seq1 comp57084_c0_seq7 comp47108_c0_seq1 comp51063_c2_seq3 comp54702_c0_seq1 comp51154_c0_seq2 comp58486_c0_seq1 comp54119_c0_seq4 comp58073_c2_seq1 comp57725_c1_seq1 comp57145_c3_seq3 comp57136_c1_seq13 comp42851_c1_seq1 comp49156_c0_seq1 comp52279_c1_seq2 comp57454_c6_seq3 comp28134_c0_seq1 comp51763_c1_seq4 comp27701_c0_seq1 comp54027_c2_seq1 comp55677_c2_seq1 comp52423_c0_seq2 comp54848_c0_seq6 comp51487_c0_seq3 comp56948_c0_seq3 comp47677_c1_seq1 comp46264_c0_seq2 comp28723_c0_seq1 comp53011_c0_seq4 comp45892_c0_seq1 comp56813_c3_seq11 comp48030_c0_seq1 comp58229_c0_seq42 comp48916_c0_seq1 comp52863_c0_seq2 comp47328_c0_seq2 comp50932_c1_seq1 comp44806_c0_seq1 comp48622_c0_seq3 comp27998_c0_seq1 comp44825_c2_seq1 comp53778_c0_seq2 comp53957_c0_seq2 comp58248_c0_seq6 comp56380_c0_seq1 comp57372_c1_seq5 comp48916_c1_seq2 comp77772_c0_seq1 comp56132_c1_seq3 comp52160_c0_seq1 comp51990_c2_seq11 comp58256_c1_seq4 comp55389_c1_seq13 comp54495_c0_seq2 comp55348_c0_seq2 comp49614_c1_seq1 comp46616_c1_seq1 comp50516_c2_seq1 comp52412_c2_seq1 comp48477_c1_seq1 comp56812_c0_seq7 comp52243_c0_seq3 comp57202_c3_seq15 comp57190_c0_seq8 comp56109_c2_seq4 comp53286_c2_seq1 comp48827_c0_seq1 comp55232_c0_seq3 comp55403_c1_seq10 comp55571_c2_seq1 comp57554_c2_seq1 comp57946_c4_seq2 comp57043_c2_seq4 comp49367_c0_seq5 comp57881_c3_seq1 comp56720_c1_seq8 comp27857_c0_seq1 comp44515_c0_seq1 comp52819_c0_seq1 comp48817_c0_seq2 comp57803_c3_seq7 comp57320_c1_seq5 comp34156_c0_seq1 comp35848_c0_seq1 comp50740_c1_seq1 comp56888_c1_seq1 comp49225_c1_seq1 comp56564_c1_seq4 comp51212_c2_seq1 comp56539_c0_seq1 comp59544_c0_seq1 comp31248_c1_seq1 comp57601_c0_seq1 comp57454_c2_seq2 comp56394_c1_seq4 comp52787_c0_seq3 comp31420_c0_seq1 comp55030_c2_seq5 comp56129_c4_seq6 comp57714_c0_seq1 comp58131_c2_seq10 comp41542_c0_seq3 comp45948_c0_seq1 comp56447_c2_seq2 comp53576_c0_seq3 comp53562_c2_seq14 comp58240_c1_seq15 comp55168_c10_seq1 comp47033_c0_seq1 comp50669_c0_seq1 comp57610_c5_seq9 comp57712_c4_seq1 comp33838_c0_seq1 comp33313_c0_seq1 comp55859_c8_seq6 comp57896_c2_seq9 comp53307_c0_seq2 comp55066_c0_seq2 comp42028_c2_seq1 comp56378_c4_seq6 comp53375_c2_seq1 comp57511_c0_seq6 comp54793_c1_seq1 comp57202_c3_seq2 comp58035_c0_seq1 comp58295_c0_seq42 comp52431_c0_seq2 comp54894_c0_seq8 comp49735_c0_seq1 comp57764_c1_seq17 comp54337_c1_seq1 comp55141_c0_seq9 comp58096_c9_seq1 comp47855_c0_seq1 comp52336_c0_seq1 comp56260_c0_seq7 comp57853_c1_seq2 comp38262_c1_seq1 comp55289_c0_seq3 comp55229_c0_seq2 comp40351_c0_seq1 comp37546_c1_seq1 comp50249_c0_seq6 comp56660_c1_seq5 comp50561_c1_seq3 comp48947_c1_seq1 comp42137_c0_seq2 comp40887_c3_seq1 comp57976_c0_seq5 comp46631_c0_seq2 comp57935_c1_seq3 comp28280_c0_seq1 comp28699_c0_seq1 comp57194_c1_seq1 comp54817_c11_seq4 comp50531_c0_seq1 comp50386_c1_seq2 comp48832_c0_seq6 comp48697_c0_seq2 comp51310_c0_seq1 comp58200_c2_seq5 comp50693_c2_seq1 comp57577_c6_seq10 comp55454_c1_seq2 comp53503_c0_seq1 comp57539_c1_seq15 comp30006_c0_seq1 comp58205_c1_seq1 comp54801_c0_seq1 comp48096_c0_seq3 comp34257_c0_seq2 comp52593_c0_seq1 comp53700_c1_seq2 comp43032_c2_seq1 comp57039_c3_seq1 comp55983_c0_seq1 comp56670_c0_seq5 comp57081_c0_seq2 comp57708_c2_seq9 comp57603_c3_seq8 comp56095_c5_seq1 comp58295_c0_seq49 comp52024_c0_seq1 comp54414_c1_seq3 comp47953_c1_seq1 comp33700_c0_seq1 comp53851_c0_seq4 comp48452_c0_seq1 comp56055_c0_seq1 comp59249_c0_seq1 comp51555_c0_seq4 comp58081_c1_seq4 comp52983_c0_seq4 comp49844_c0_seq1 comp49659_c0_seq2 comp29044_c0_seq1 comp53231_c0_seq1 comp56961_c2_seq1 comp54303_c0_seq1 comp55603_c3_seq3 comp56722_c1_seq10 comp53567_c0_seq1 comp56578_c2_seq7 comp46353_c1_seq2 comp56008_c0_seq5 comp55293_c1_seq6 comp56331_c4_seq2 comp54256_c1_seq2 comp50445_c0_seq2 comp56288_c1_seq6 comp55544_c0_seq1 comp52987_c0_seq2 comp45870_c1_seq1 comp53829_c0_seq3 comp57958_c0_seq2 comp57739_c1_seq2 comp48987_c0_seq2 comp56918_c0_seq4 comp58237_c5_seq12 comp53527_c2_seq6 comp51363_c0_seq5 comp48095_c0_seq2 comp41781_c0_seq1 comp55681_c0_seq1 comp49598_c0_seq1 comp54601_c1_seq4 comp57037_c0_seq2 comp53710_c1_seq5 comp53186_c0_seq1 comp57925_c3_seq1 comp55052_c2_seq3 comp50886_c1_seq5 comp56471_c1_seq2 comp48386_c1_seq1 comp55056_c0_seq1 comp52488_c0_seq2 comp55051_c1_seq2 comp48141_c0_seq1 comp50242_c1_seq2 comp28410_c1_seq1 comp55365_c0_seq1 comp53095_c0_seq1 comp53335_c0_seq8 comp56497_c1_seq5 comp50258_c0_seq1 comp3166_c0_seq1 comp50880_c0_seq1 comp57397_c9_seq46 comp50801_c0_seq2 comp52954_c0_seq5 comp56580_c0_seq1 comp32182_c1_seq1 comp57509_c3_seq4 comp57509_c5_seq2 comp49407_c0_seq1 comp57602_c0_seq2 comp55881_c1_seq13 comp56005_c0_seq4 comp57858_c1_seq5 comp55951_c4_seq2 comp53301_c3_seq2 comp54908_c1_seq8 comp53893_c0_seq6 comp56657_c1_seq6 comp50220_c0_seq1 comp57811_c1_seq4 comp54763_c3_seq4 comp36491_c4_seq1 comp28450_c0_seq1 comp53202_c4_seq10 comp58253_c1_seq8 comp50027_c0_seq1 comp53043_c2_seq3 comp57371_c0_seq1 comp51296_c0_seq3 comp51905_c0_seq1 comp47037_c0_seq1 comp54613_c1_seq1 comp63224_c0_seq1 comp56189_c0_seq4 comp52334_c0_seq2 comp50896_c1_seq2 comp53689_c0_seq3 comp32509_c0_seq1 comp54476_c0_seq1 comp51628_c0_seq1 comp53229_c0_seq4 comp54193_c3_seq2 comp153623_c0_seq1 comp56762_c3_seq1 comp55739_c0_seq1 comp34611_c0_seq1 comp57615_c1_seq10 comp54610_c0_seq4 comp54280_c1_seq3 comp54447_c0_seq1 comp47686_c4_seq3 comp55816_c0_seq3 comp51867_c0_seq1 comp46534_c0_seq1 comp29231_c0_seq1 comp48488_c0_seq1 comp56447_c9_seq1 comp55430_c2_seq2 comp52117_c0_seq2 comp47984_c3_seq2 comp56814_c1_seq3 comp57636_c0_seq3 comp46927_c0_seq1 comp54097_c0_seq1 comp57623_c1_seq1 comp52872_c6_seq1 comp55560_c3_seq2 comp30546_c0_seq1 comp58136_c0_seq8 comp50000_c1_seq3 comp42051_c0_seq1 comp54380_c0_seq1 comp56236_c0_seq2 comp57592_c0_seq1 comp54830_c2_seq1 comp33211_c0_seq1 comp49868_c0_seq1 comp57577_c16_seq3 comp50139_c1_seq1 comp56712_c0_seq1 comp52848_c0_seq1 comp46973_c0_seq1 comp53885_c2_seq1 comp45303_c0_seq1 comp38648_c0_seq1 comp53147_c2_seq2 comp52855_c1_seq1 comp57620_c3_seq15 comp58258_c0_seq3 comp54021_c1_seq1 comp55324_c0_seq6 comp58130_c0_seq9 comp54964_c4_seq1 comp56121_c0_seq4 comp56370_c0_seq1 comp52180_c0_seq1 comp53626_c0_seq2 comp135578_c0_seq1 comp57464_c0_seq2 comp44114_c0_seq1 comp57740_c2_seq1 comp55741_c2_seq2 comp36760_c1_seq1 comp29028_c0_seq1 comp56128_c2_seq1 comp54660_c3_seq6 comp56470_c1_seq1 comp51090_c0_seq1 comp55797_c0_seq3 comp52843_c0_seq9 comp56717_c0_seq6 comp42966_c2_seq1 comp46076_c1_seq5 comp52755_c0_seq1 comp52728_c1_seq2 comp55898_c0_seq2 comp49256_c3_seq1 comp48948_c0_seq3 comp86860_c0_seq1 comp56218_c1_seq1 comp50482_c2_seq2 comp51748_c0_seq1 comp56641_c0_seq5 comp52051_c0_seq1 comp57779_c0_seq4 comp49993_c0_seq1 comp37475_c0_seq2 comp57147_c0_seq10 comp48542_c0_seq1 comp33120_c0_seq1 comp51540_c4_seq4 comp52111_c2_seq3 comp51590_c0_seq1 comp40887_c1_seq1 comp53847_c1_seq1 comp53494_c1_seq1 comp56285_c0_seq2 comp30130_c0_seq1 comp53367_c1_seq1 comp47510_c0_seq1 comp37679_c0_seq1 comp48520_c1_seq1 comp54022_c0_seq1 comp55932_c0_seq1 comp35806_c0_seq1 comp41886_c0_seq1 comp45842_c0_seq3 comp50308_c0_seq2 comp43887_c0_seq1 comp56223_c2_seq3 comp57458_c2_seq18 comp50164_c0_seq2 comp56419_c0_seq1 comp31919_c0_seq3 comp49939_c0_seq1 comp46513_c0_seq1 comp47734_c0_seq5 comp48234_c0_seq2 comp54041_c1_seq5 comp54206_c0_seq2 comp48498_c0_seq1 comp57547_c1_seq1 comp56920_c11_seq3 comp56671_c0_seq2 comp57222_c3_seq9 comp57913_c0_seq7 comp55604_c3_seq3 comp57422_c0_seq2 comp54711_c1_seq7 comp56735_c0_seq5 comp48759_c1_seq1 comp56284_c0_seq7 comp49662_c0_seq1 comp45799_c0_seq2 comp54715_c0_seq1 comp58202_c2_seq4 comp53463_c1_seq7 comp57822_c2_seq12 comp55022_c0_seq3 comp33088_c0_seq1 comp57644_c0_seq7 comp43135_c0_seq1 comp57775_c0_seq5 comp50667_c0_seq1 comp44830_c0_seq1 comp54045_c1_seq1 comp52173_c0_seq1 comp53380_c0_seq1 comp40624_c0_seq1 comp53748_c0_seq11 comp54887_c6_seq4 comp57971_c4_seq24 comp57489_c0_seq11 comp56957_c4_seq9 comp53810_c0_seq2 comp55886_c1_seq1 comp49947_c1_seq1 comp44970_c2_seq2 comp55640_c5_seq4 comp52035_c1_seq4 comp55756_c0_seq2 comp47076_c0_seq1 comp56964_c3_seq8 comp57134_c1_seq2 comp47726_c0_seq1 comp56274_c2_seq2 comp55538_c1_seq7 comp44309_c0_seq1 comp57698_c0_seq3 comp45923_c1_seq1 comp51642_c0_seq1 comp57996_c5_seq15 comp46675_c1_seq1 comp55710_c0_seq4 comp46023_c0_seq1 comp54475_c0_seq1 comp54785_c1_seq11 comp56099_c0_seq17 comp57524_c0_seq8 comp54850_c0_seq5 comp58259_c1_seq5 comp56944_c0_seq12 comp58812_c0_seq1 comp31312_c0_seq1 comp47684_c0_seq2 comp56629_c4_seq1 comp54768_c2_seq1 comp58268_c2_seq8 comp53061_c5_seq1 comp33262_c0_seq1 comp49906_c1_seq2 comp42168_c0_seq2 comp53614_c0_seq1 comp55375_c2_seq1 comp51475_c0_seq1 comp55313_c0_seq1 comp43907_c0_seq1 comp57190_c0_seq1 comp54643_c1_seq1 comp46203_c0_seq1 comp50497_c0_seq1 comp58244_c0_seq10 comp56911_c3_seq1 comp57695_c1_seq5 comp49757_c0_seq1 comp42969_c0_seq2 comp52659_c1_seq1 comp49126_c0_seq2 comp57464_c0_seq6 comp54643_c0_seq3 comp53917_c1_seq2 comp56293_c0_seq4 comp48945_c0_seq1 comp53723_c0_seq2 comp46657_c0_seq2 comp27686_c0_seq1 comp378722_c0_seq1 comp46255_c0_seq1 comp56769_c1_seq1 comp57801_c0_seq6 comp48326_c0_seq1 comp52467_c0_seq14 comp50310_c1_seq1 comp55421_c0_seq4 comp50906_c2_seq3 comp56638_c0_seq1 comp120995_c0_seq1 comp53762_c1_seq1 comp50376_c0_seq1 comp58063_c2_seq1 comp52258_c2_seq2 comp53892_c0_seq1 comp46321_c0_seq1 comp58293_c2_seq6 comp56155_c1_seq5 comp93617_c0_seq1 comp57950_c0_seq5 comp51039_c1_seq1 comp45907_c0_seq1 comp51831_c0_seq1 comp56910_c2_seq2 comp53636_c0_seq1 comp57500_c2_seq14 comp42663_c0_seq1 comp55858_c0_seq1 comp36249_c0_seq1 comp53241_c0_seq11 comp30267_c0_seq1 comp29840_c0_seq1 comp55742_c0_seq3 comp57260_c0_seq2 comp54705_c0_seq2 comp56675_c0_seq8 comp55851_c0_seq1 comp56774_c0_seq1 comp58009_c6_seq2 comp42139_c0_seq3 comp57895_c0_seq1 comp50127_c2_seq2 comp46383_c0_seq5 comp49440_c0_seq1 comp51421_c0_seq1 comp56885_c0_seq4 comp52594_c2_seq2 comp55831_c2_seq4 comp55186_c4_seq5 comp37399_c1_seq1 comp48534_c0_seq2 comp50360_c0_seq2 comp52048_c0_seq2 comp50943_c0_seq3 comp43862_c3_seq1 comp55281_c0_seq1 comp55442_c0_seq1 comp52608_c0_seq2 comp51387_c0_seq2 comp50333_c0_seq1 comp47939_c2_seq4 comp47213_c0_seq1 comp51104_c0_seq4 comp44751_c0_seq2 comp46149_c0_seq1 comp56672_c0_seq1 comp56283_c1_seq2 comp56951_c1_seq1 comp56268_c0_seq4 comp43606_c4_seq1 comp31290_c0_seq1 comp53779_c2_seq7 comp48563_c0_seq1 comp57781_c0_seq1 comp57489_c0_seq4 comp48662_c0_seq2 comp52638_c1_seq3 comp43486_c0_seq1 comp55774_c4_seq3 comp56800_c1_seq4 comp57648_c0_seq1 comp49185_c0_seq2 comp58011_c1_seq10 comp57882_c1_seq2 comp56255_c8_seq2 comp57671_c11_seq1 comp46682_c2_seq1 comp56922_c1_seq1 comp57168_c0_seq2 comp51397_c0_seq2 comp50303_c0_seq2 comp56716_c2_seq2 comp57986_c3_seq2 comp56703_c3_seq6 comp56430_c1_seq4 comp57455_c2_seq1 comp52794_c2_seq1 comp40002_c0_seq1 comp55893_c0_seq1 comp54498_c0_seq7 comp47559_c1_seq1 comp48148_c1_seq1 comp50891_c0_seq2 comp52610_c0_seq2 comp46266_c0_seq1 comp55882_c5_seq14 comp46952_c0_seq1 comp29470_c0_seq2 comp54000_c0_seq1 comp57572_c1_seq2 comp55782_c1_seq4 comp32946_c0_seq1 comp43813_c1_seq1 comp41403_c0_seq1 comp54806_c0_seq2 comp57122_c2_seq3 comp57861_c0_seq11 comp51733_c0_seq3 comp48656_c0_seq12 comp56429_c0_seq13 comp58240_c15_seq3 comp51485_c0_seq1 comp53102_c1_seq7 comp52257_c0_seq2 comp31727_c0_seq1 comp53512_c0_seq2 comp51637_c0_seq1 comp53441_c0_seq2 comp54099_c0_seq6 comp57473_c0_seq11 comp47231_c1_seq1 comp52499_c0_seq3 comp52400_c1_seq1 comp52865_c0_seq1 comp55111_c1_seq1 comp57766_c0_seq3 comp48010_c0_seq2 comp55216_c1_seq3 comp56554_c4_seq10 comp57545_c1_seq13 comp58121_c0_seq3 comp56598_c0_seq2 comp53392_c0_seq5 comp46130_c0_seq1 comp32761_c0_seq1 comp29318_c0_seq1 comp57654_c0_seq3 comp54186_c1_seq1 comp48189_c0_seq2 comp56255_c7_seq1 comp56724_c0_seq1 comp48103_c0_seq2 comp57731_c0_seq1 comp57427_c1_seq1 comp47993_c0_seq2 comp58084_c0_seq19 comp53078_c1_seq4 comp55591_c1_seq1 comp49405_c0_seq1 comp54383_c1_seq3 comp57765_c4_seq1 comp57091_c9_seq3 comp55846_c0_seq9 comp54135_c1_seq1 comp56175_c0_seq2 comp57572_c1_seq9 comp52286_c1_seq3 comp55947_c1_seq6 comp28377_c0_seq1 comp57152_c6_seq1 comp32900_c0_seq1 comp43578_c0_seq1 comp57488_c5_seq3 comp51230_c1_seq6 comp57520_c0_seq7 comp57967_c2_seq8 comp55711_c0_seq12 comp55494_c2_seq1 comp53386_c0_seq2 comp56784_c1_seq1 comp55408_c0_seq3 comp44228_c0_seq2 comp48153_c1_seq1 comp47320_c1_seq1 comp48663_c3_seq1 comp55144_c0_seq5 comp53349_c1_seq3 comp58220_c0_seq9 comp55704_c1_seq16 comp56198_c1_seq3 comp38410_c0_seq1 comp31660_c1_seq1 comp45362_c0_seq1 comp49543_c0_seq1 comp54278_c0_seq4 comp46502_c0_seq1 comp57419_c0_seq2 comp55738_c0_seq5 comp58138_c2_seq15 comp58123_c0_seq2 comp55999_c1_seq1 comp57567_c0_seq3 comp55921_c0_seq1 comp52072_c1_seq3 comp53430_c0_seq3 comp57089_c0_seq1 comp57604_c0_seq7 comp56097_c5_seq1 comp54064_c0_seq1 comp29479_c0_seq1 comp55256_c4_seq1 comp57753_c1_seq2 comp56856_c1_seq1 comp46179_c1_seq1 comp56282_c0_seq1 comp57189_c1_seq2 comp53527_c2_seq1 comp54648_c0_seq1 comp51334_c0_seq2 comp57351_c3_seq15 comp54979_c0_seq3 comp40977_c0_seq1 comp54078_c0_seq13 comp47705_c0_seq1 comp58111_c2_seq2 comp52567_c0_seq3 comp54415_c0_seq4 comp49418_c0_seq5 comp44434_c0_seq1 comp53455_c0_seq2 comp44671_c4_seq2 comp54271_c0_seq2 comp44906_c0_seq2 comp54786_c0_seq2 comp52562_c0_seq1 comp57487_c2_seq1 comp46156_c1_seq2 comp58382_c0_seq1 comp57021_c0_seq1 comp52389_c0_seq5 comp55867_c3_seq11 comp47287_c0_seq1 comp52245_c0_seq1 comp32785_c0_seq1 comp48329_c0_seq1 comp56900_c0_seq2 comp50377_c0_seq1 comp57632_c1_seq10 comp42049_c0_seq1 comp35893_c0_seq1 comp51608_c0_seq4 comp41142_c0_seq2 comp54823_c0_seq1 comp40016_c0_seq1 comp29443_c0_seq1 comp51401_c0_seq7 comp55282_c0_seq7 comp57254_c9_seq18 comp55161_c0_seq1 comp42748_c4_seq1 comp51550_c0_seq2 comp49061_c0_seq2 comp287398_c0_seq1 comp61121_c0_seq1 comp55464_c1_seq3 comp53620_c1_seq1 comp51123_c0_seq1 comp55579_c3_seq10 comp51520_c1_seq1 comp50756_c0_seq3 comp54143_c0_seq6 comp42689_c0_seq1 comp51614_c1_seq1 comp54703_c2_seq2 comp58257_c0_seq16 comp49624_c0_seq2 comp49505_c0_seq3 comp56654_c2_seq3 comp57367_c0_seq2 comp49940_c0_seq1 comp51124_c0_seq2 comp44118_c0_seq1 comp48154_c0_seq1 comp43689_c0_seq4 comp51649_c0_seq1 comp55943_c0_seq1 comp58033_c0_seq7 comp29167_c0_seq1 comp49800_c0_seq2 comp50577_c1_seq1 comp41580_c0_seq1 comp49537_c1_seq2 comp48941_c0_seq2 comp52076_c0_seq3 comp50089_c0_seq1 comp29060_c0_seq1 comp54162_c0_seq1 comp45729_c0_seq3 comp56657_c1_seq2 comp56841_c0_seq1 comp55607_c0_seq1 comp55504_c0_seq8 comp55819_c0_seq2 comp58224_c0_seq20 comp53047_c1_seq2 comp55640_c5_seq20 comp52453_c2_seq4 comp52640_c0_seq1 comp48682_c0_seq1 comp58234_c0_seq10 comp53243_c0_seq1 comp54509_c2_seq11 comp55359_c0_seq2 comp57975_c2_seq24 comp54079_c4_seq1 comp58273_c0_seq4 comp58194_c0_seq14 comp50792_c0_seq1 comp56603_c3_seq10 comp57410_c1_seq8 comp49537_c0_seq1 comp55640_c5_seq19 comp58177_c1_seq1 comp52212_c0_seq1 comp54740_c0_seq2 comp58429_c0_seq1 comp47232_c5_seq1 comp46021_c0_seq2 comp34570_c0_seq2 comp57726_c0_seq2 comp49758_c0_seq2 comp48182_c1_seq1 comp49363_c0_seq3 comp46888_c0_seq2 comp54626_c0_seq3 comp49706_c0_seq1 comp52299_c0_seq7 comp49596_c0_seq1 comp48219_c3_seq1 comp52384_c1_seq2 comp52739_c0_seq2 comp55240_c0_seq1 comp53359_c0_seq2 comp30702_c0_seq4 comp54945_c0_seq3 comp49845_c1_seq1 comp57539_c1_seq8 comp44665_c0_seq1 comp52037_c0_seq2 comp54526_c0_seq4 comp56797_c2_seq5 comp56075_c2_seq18 comp46680_c0_seq1 comp44041_c0_seq1 comp52798_c0_seq2 comp56586_c4_seq1 comp54864_c4_seq2 comp57038_c0_seq9 comp50092_c0_seq1 comp41107_c0_seq2 comp32921_c0_seq1 comp57031_c1_seq6 comp53171_c0_seq1 comp50276_c0_seq2 comp42935_c0_seq1 comp51167_c0_seq1 comp46308_c0_seq1 comp45997_c0_seq1 comp48646_c2_seq2 comp51806_c0_seq2 comp49914_c1_seq1 comp58131_c2_seq6 comp54193_c11_seq3 comp58191_c1_seq7 comp53773_c0_seq11 comp56879_c0_seq8 comp57198_c4_seq11 comp56324_c0_seq7 comp54367_c1_seq3 comp56051_c0_seq3 comp36543_c0_seq1 comp55829_c1_seq8 comp57012_c0_seq6 comp51352_c0_seq3 comp50275_c0_seq1 comp56192_c5_seq1 comp46672_c1_seq1 comp50978_c0_seq1 comp57856_c15_seq23 comp52753_c0_seq1 comp57395_c2_seq9 comp45837_c0_seq2 comp51705_c0_seq1 comp46666_c0_seq1 comp48265_c0_seq2 comp48038_c0_seq6 comp57659_c5_seq9 comp57305_c0_seq2 comp51567_c1_seq1 comp54401_c8_seq21 comp54787_c0_seq1 comp51990_c2_seq8 comp57237_c7_seq7 comp57450_c1_seq5 comp50965_c0_seq2 comp58047_c0_seq8 comp57701_c2_seq4 comp49106_c0_seq1 comp50500_c2_seq1 comp53346_c0_seq4 comp47646_c0_seq1 comp46608_c0_seq1 comp52202_c0_seq2 comp45246_c0_seq1 comp54335_c0_seq6 comp36403_c0_seq1 comp55323_c0_seq1 comp50429_c0_seq3 comp56747_c0_seq3 comp56149_c0_seq2 comp53370_c0_seq5 comp55670_c5_seq19 comp55886_c9_seq9 comp49320_c0_seq1 comp46135_c0_seq1 comp57028_c0_seq5 comp53886_c0_seq1 comp56085_c0_seq4 comp56460_c0_seq3 comp55009_c0_seq2 comp53893_c0_seq2 comp52262_c0_seq6 comp57787_c0_seq4 comp4681_c0_seq1 comp52383_c0_seq5 comp57357_c2_seq9 comp46593_c0_seq1 comp54018_c0_seq4 comp43041_c0_seq1 comp49739_c0_seq2 comp56916_c0_seq2 comp57232_c1_seq7 comp53562_c2_seq4 comp50548_c2_seq1 comp56845_c9_seq3 comp55516_c1_seq10 comp57638_c5_seq6 comp55264_c5_seq4 comp51882_c0_seq3 comp57618_c0_seq14 comp53332_c0_seq4 comp56809_c4_seq10 comp47251_c1_seq1 comp58299_c3_seq12 comp48234_c0_seq1 comp51225_c1_seq2 comp56441_c1_seq10 comp53428_c0_seq1 comp54825_c0_seq1 comp51879_c0_seq1 comp56641_c3_seq17 comp54151_c2_seq6 comp48806_c0_seq1 comp53560_c2_seq1 comp53387_c0_seq1 comp30956_c0_seq1 comp54435_c1_seq1 comp55953_c0_seq2 comp32338_c0_seq1 comp32236_c0_seq1 comp56380_c0_seq5 comp33162_c0_seq1 comp43862_c4_seq1 comp46941_c0_seq1 comp45801_c1_seq3 comp46529_c0_seq1 comp48582_c0_seq4 comp41933_c0_seq1 comp45885_c0_seq2 comp56519_c0_seq5 comp48206_c0_seq1 comp58246_c1_seq6 comp58180_c3_seq5 comp55993_c5_seq6 comp49936_c0_seq1 comp45709_c0_seq1 comp56550_c3_seq1 comp52568_c7_seq1 comp47000_c0_seq2 comp58184_c0_seq6 comp48497_c0_seq1 comp58213_c0_seq8 comp55497_c0_seq1 comp51150_c0_seq7 comp55141_c0_seq7 comp52521_c0_seq10 comp52521_c0_seq8 comp45829_c0_seq1 comp36491_c1_seq1 comp52080_c1_seq1 comp53959_c0_seq4 comp45775_c0_seq2 comp58072_c1_seq2 comp58058_c0_seq3 comp41032_c0_seq1 comp55196_c0_seq7 comp48140_c0_seq2 comp57351_c3_seq7 comp53159_c1_seq1 comp33779_c0_seq1 comp56272_c0_seq6 comp56304_c2_seq3 comp48399_c0_seq1 comp55174_c1_seq1 comp56997_c1_seq4 comp56863_c0_seq1 comp57290_c1_seq2 comp58166_c1_seq13 comp55269_c0_seq1 comp30733_c0_seq1 comp28596_c0_seq1 comp41672_c0_seq2 comp48708_c0_seq2 comp56580_c0_seq2 comp50043_c0_seq1 comp57516_c1_seq4 comp43946_c0_seq1 comp56444_c2_seq1 comp52632_c1_seq1 comp42729_c0_seq1 comp54003_c0_seq1 comp58086_c1_seq17 comp43138_c1_seq1 comp56494_c0_seq6 comp52968_c0_seq7 comp57172_c1_seq16 comp51939_c0_seq1 comp50602_c0_seq1 comp54796_c0_seq3 comp54670_c2_seq4 comp54738_c0_seq1 comp58217_c0_seq38 comp31745_c1_seq1 comp53318_c0_seq1 comp54770_c0_seq1 comp42991_c0_seq1 comp46240_c0_seq2 comp49467_c7_seq2 comp51679_c4_seq6 comp52539_c0_seq1 comp55986_c3_seq2 comp56996_c0_seq5 comp51448_c1_seq6 comp46873_c0_seq1 comp56951_c0_seq1 comp41827_c0_seq1 comp46311_c0_seq2 comp55345_c0_seq7 comp55533_c0_seq10 comp41912_c0_seq2 comp39436_c0_seq1 comp53541_c0_seq1 comp48815_c0_seq1 comp51358_c0_seq2 comp41855_c0_seq2 comp51471_c0_seq1 comp57352_c1_seq2 comp57857_c1_seq2 comp57994_c0_seq54 comp50304_c1_seq1 comp57961_c1_seq9 comp50821_c3_seq2 comp58243_c0_seq16 comp58210_c0_seq12 comp49994_c0_seq5 comp51108_c2_seq1 comp29626_c0_seq1 comp56332_c0_seq8 comp55193_c2_seq2 comp30887_c0_seq1 comp53175_c3_seq1 comp58237_c5_seq6 comp57701_c2_seq20 comp47801_c0_seq3 comp51762_c0_seq2 comp54815_c2_seq3 comp34500_c0_seq1 comp52794_c0_seq1 comp52134_c1_seq1 comp57575_c2_seq2 comp56610_c2_seq6 comp50368_c0_seq1 comp49027_c0_seq1 comp45382_c2_seq1 comp49981_c0_seq1 comp56940_c0_seq2 comp51389_c0_seq3 comp51691_c0_seq1 comp56385_c0_seq9 comp58257_c0_seq8 comp52780_c3_seq1 comp54924_c0_seq1 comp48066_c0_seq3 comp52791_c0_seq1 comp49349_c0_seq3 comp49698_c0_seq1 comp116100_c0_seq1 comp56828_c0_seq2 comp62527_c0_seq1 comp49392_c0_seq3 comp52866_c1_seq3 comp49786_c2_seq2 comp51851_c0_seq2 comp48204_c1_seq5 comp54921_c0_seq3 comp31302_c0_seq1 comp30162_c0_seq1 comp52606_c1_seq2 comp53529_c1_seq1 comp44822_c0_seq2 comp57434_c5_seq1 comp57776_c3_seq1 comp43138_c0_seq1 comp52323_c2_seq1 comp57845_c0_seq30 comp52508_c0_seq1 comp53848_c0_seq1 comp62416_c0_seq1 comp55283_c0_seq3 comp58059_c0_seq3 comp55073_c1_seq3 comp55042_c1_seq5 comp43064_c0_seq1 comp57761_c0_seq4 comp49093_c0_seq1 comp55649_c0_seq6 comp57483_c0_seq5 comp55985_c1_seq1 comp51818_c0_seq5 comp56128_c1_seq1 comp55637_c0_seq1 comp53838_c0_seq5 comp57440_c0_seq6 comp47093_c2_seq1 comp57946_c9_seq1 comp53220_c0_seq2 comp51406_c1_seq1 comp56659_c2_seq2 comp55236_c2_seq7 comp57199_c1_seq1 comp45496_c0_seq1 comp56576_c1_seq10 comp55610_c0_seq3 comp49051_c0_seq1 comp51941_c0_seq1 comp51115_c0_seq2 comp53316_c0_seq1 comp55968_c0_seq2 comp47556_c2_seq1 comp57340_c0_seq4 comp57636_c0_seq8 comp54124_c0_seq1 comp54724_c0_seq1 comp53806_c0_seq4 comp56675_c0_seq1 comp58254_c0_seq25 comp45342_c0_seq3 comp58155_c3_seq24 comp57320_c3_seq3 comp59518_c0_seq1 comp45252_c0_seq1 comp46111_c1_seq1 comp54623_c1_seq2 comp55961_c0_seq1 comp53803_c2_seq1 comp55345_c0_seq3 comp50296_c0_seq1 comp52951_c0_seq6 comp53189_c0_seq2 comp51418_c2_seq2 comp41763_c1_seq1 comp54494_c0_seq3 comp54786_c0_seq6 comp54217_c0_seq1 comp65384_c0_seq1 comp49923_c0_seq1 comp47470_c0_seq3 comp45598_c0_seq1 comp52626_c0_seq1 comp44908_c1_seq1 comp49786_c2_seq3 comp30541_c0_seq1 comp39891_c0_seq1 comp51530_c0_seq1 comp36377_c0_seq2 comp50086_c1_seq1 comp43069_c0_seq1 comp53881_c0_seq1 comp54005_c0_seq1 comp56896_c0_seq1 comp49963_c1_seq1 comp49779_c0_seq1 comp53896_c2_seq1 comp51166_c0_seq2 comp47092_c2_seq1 comp58104_c2_seq8 comp51366_c0_seq1 comp43595_c0_seq1 comp41793_c0_seq2 comp52126_c0_seq3 comp31007_c0_seq1 comp57143_c0_seq7 comp57669_c0_seq38 comp49649_c0_seq2 comp57805_c1_seq16 comp52430_c0_seq1 comp54453_c1_seq1 comp52210_c1_seq1 comp48651_c0_seq1 comp49859_c1_seq1 comp57752_c0_seq7 comp57323_c1_seq6 comp49769_c0_seq1 comp56346_c0_seq18 comp48973_c0_seq1 comp53217_c0_seq1 comp55904_c0_seq1 comp32678_c0_seq2 comp56665_c1_seq6 comp65213_c0_seq1 comp57124_c4_seq1 comp57156_c2_seq1 comp45836_c0_seq1 comp27867_c0_seq1 comp53045_c0_seq1 comp53993_c0_seq2 comp54208_c0_seq1 comp52248_c0_seq2 comp53363_c0_seq3 comp54797_c0_seq2 comp57011_c0_seq2 comp51387_c0_seq1 comp57136_c1_seq9 comp42313_c0_seq1 comp52688_c0_seq2 comp28551_c0_seq1 comp45030_c0_seq1 comp54723_c0_seq3 comp55998_c0_seq4 comp51924_c3_seq5 comp50228_c0_seq2 comp31091_c0_seq1 comp46380_c0_seq1 comp41482_c1_seq1 comp58084_c0_seq12 comp49310_c0_seq3 comp55436_c5_seq3 comp53518_c0_seq3 comp45754_c0_seq1 comp44426_c1_seq1 comp56422_c3_seq1 comp55043_c1_seq3 comp51823_c1_seq1 comp39399_c1_seq1 comp52136_c1_seq2 comp37144_c0_seq1 comp57935_c1_seq4 comp48953_c0_seq2 comp54193_c3_seq4 comp57031_c1_seq8 comp52735_c0_seq13 comp51063_c2_seq2 comp54619_c1_seq5 comp50663_c0_seq1 comp57770_c1_seq1 comp48856_c3_seq1 comp56829_c0_seq1 comp55618_c0_seq2 comp57889_c0_seq4 comp52149_c0_seq10 comp34091_c0_seq1 comp57900_c4_seq8 comp56912_c0_seq1 comp53818_c1_seq1 comp56141_c2_seq5 comp51078_c0_seq3 comp55416_c1_seq1 comp53721_c1_seq14 comp46976_c0_seq1 comp58001_c4_seq1 comp52650_c0_seq1 comp57451_c0_seq1 comp55133_c0_seq11 comp46599_c0_seq1 comp53493_c0_seq7 comp58259_c1_seq17 comp58187_c3_seq8 comp55600_c3_seq4 comp57909_c0_seq2 comp53282_c0_seq1 comp54491_c0_seq4 comp51923_c0_seq6 comp46825_c0_seq2 comp56599_c0_seq3 comp55992_c0_seq1 comp51812_c0_seq1 comp55617_c0_seq11 comp53439_c3_seq1 comp58269_c0_seq8 comp47121_c0_seq1 comp46906_c1_seq1 comp49861_c0_seq2 comp54120_c3_seq2 comp29014_c0_seq1 comp56571_c0_seq25 comp53400_c1_seq3 comp58213_c0_seq10 comp53207_c0_seq2 comp51883_c0_seq4 comp53409_c0_seq2 comp58082_c1_seq5 comp46748_c0_seq1 comp45264_c2_seq1 comp57900_c4_seq1 comp29624_c0_seq1 comp48844_c0_seq11 comp56048_c0_seq4 comp52343_c0_seq2 comp50937_c2_seq1 comp58230_c0_seq3 comp47014_c0_seq1 comp53603_c2_seq6 comp49574_c0_seq2 comp55432_c0_seq1 comp52764_c0_seq3 comp56961_c3_seq20 comp56727_c0_seq2 comp57071_c1_seq24 comp58131_c2_seq4 comp82605_c0_seq1 comp45891_c0_seq4 comp50257_c0_seq1 comp53773_c0_seq15 comp46539_c0_seq1 comp53714_c0_seq1 comp55027_c2_seq4 comp56945_c1_seq2 comp42729_c4_seq1 comp55119_c0_seq1 comp29128_c0_seq1 comp31250_c0_seq1 comp51627_c0_seq1 comp51453_c0_seq1 comp55037_c0_seq1 comp56288_c1_seq9 comp52516_c0_seq6 comp58003_c0_seq3 comp60797_c0_seq1 comp52651_c0_seq1 comp37969_c0_seq1 comp58081_c1_seq1 comp57509_c3_seq13 comp46058_c0_seq1 comp56962_c1_seq2 comp53487_c1_seq2 comp56235_c1_seq2 comp48096_c0_seq1 comp56357_c4_seq5 comp54394_c4_seq18 comp54740_c0_seq5 comp57471_c2_seq6 comp52979_c2_seq4 comp30583_c0_seq1 comp53141_c0_seq1 comp50577_c4_seq4 comp56374_c0_seq6 comp27741_c1_seq1 comp54773_c0_seq2 comp49513_c0_seq1 comp51907_c0_seq1 comp57587_c2_seq11 comp55506_c0_seq1 comp55749_c7_seq3 comp54139_c7_seq1 comp53475_c0_seq2 comp58150_c0_seq12 comp58234_c0_seq13 comp82718_c0_seq1 comp56031_c1_seq4 comp58202_c2_seq11 comp55809_c1_seq10 comp163490_c0_seq1 comp47258_c0_seq3 comp35717_c1_seq1 comp28200_c0_seq1 comp54619_c0_seq1 comp183449_c0_seq1 comp56286_c1_seq2 comp63262_c0_seq1 comp57294_c2_seq2 comp57102_c2_seq2 comp51999_c0_seq1 comp44434_c0_seq5 comp43800_c0_seq2 comp53291_c0_seq2 comp55270_c0_seq8 comp55810_c0_seq4 comp52077_c4_seq1 comp55449_c0_seq4 comp50576_c1_seq1 comp53582_c0_seq2 comp51772_c0_seq1 comp57960_c0_seq6 comp52800_c0_seq3 comp49426_c0_seq4 comp56354_c1_seq25 comp55595_c0_seq1 comp57979_c0_seq4 comp57430_c1_seq10 comp53388_c0_seq2 comp55963_c1_seq1 comp56208_c2_seq3 comp54410_c0_seq1 comp29677_c0_seq1 comp54096_c0_seq2 comp57446_c0_seq7 comp45853_c1_seq1 comp54332_c1_seq3 comp62714_c0_seq1 comp56653_c1_seq32 comp57642_c1_seq3 comp46027_c0_seq2 comp54029_c0_seq5 comp57822_c2_seq5 comp57357_c2_seq10 comp28887_c0_seq1 comp58029_c4_seq1 comp53219_c0_seq2 comp56735_c0_seq8 comp53646_c0_seq3 comp49220_c0_seq1 comp43198_c0_seq1 comp63618_c0_seq1 comp52110_c0_seq1 comp57599_c0_seq29 comp57898_c3_seq14 comp57506_c0_seq1 comp58067_c0_seq4 comp54696_c1_seq2 comp58076_c6_seq1 comp52418_c0_seq1 comp50454_c0_seq3 comp29390_c0_seq1 comp50986_c0_seq2 comp53241_c0_seq2 comp50175_c0_seq7 comp57675_c3_seq42 comp63380_c0_seq1 comp53368_c1_seq1 comp51370_c0_seq3 comp50960_c0_seq6 comp36505_c0_seq1 comp49701_c1_seq1 comp51686_c0_seq1 comp46293_c0_seq1 comp31778_c0_seq1 comp29415_c0_seq2 comp48661_c0_seq1 comp57098_c4_seq6 comp50799_c0_seq2 comp45735_c2_seq1 comp51588_c1_seq1 comp45361_c1_seq1 comp53167_c0_seq2 comp52580_c0_seq2 comp57932_c0_seq9 comp54839_c0_seq2 comp53900_c0_seq2 comp52981_c0_seq2 comp54680_c0_seq2 comp57858_c1_seq4 comp32685_c0_seq1 comp56735_c1_seq1 comp56883_c0_seq5 comp52492_c1_seq1 comp42910_c0_seq2 comp57158_c0_seq5 comp594818_c0_seq1 comp28976_c1_seq1 comp47135_c0_seq2 comp42150_c1_seq1 comp56151_c0_seq27 comp43578_c0_seq2 comp55275_c2_seq1 comp52756_c0_seq2 comp57093_c1_seq1 comp55629_c1_seq10 comp52359_c0_seq1 comp53580_c1_seq2 comp48484_c0_seq1 comp50784_c0_seq3 comp32741_c1_seq1 comp56950_c0_seq1 comp54830_c2_seq12 comp55475_c1_seq1 comp48470_c0_seq1 comp53627_c3_seq1 comp49781_c0_seq1 comp56099_c0_seq4 comp53512_c0_seq1 comp53506_c1_seq1 comp56964_c3_seq9 comp57306_c1_seq8 comp57985_c5_seq2 comp47680_c3_seq1 comp48658_c1_seq3 comp53749_c1_seq1 comp57803_c3_seq6 comp56075_c5_seq4 comp42694_c1_seq1 comp57096_c0_seq3 comp53305_c0_seq3 comp50378_c1_seq3 comp59832_c0_seq1 comp57644_c0_seq2 comp58300_c0_seq4 comp57511_c0_seq4 comp48542_c1_seq2 comp54201_c0_seq1 comp55698_c0_seq8 comp43164_c0_seq1 comp48578_c1_seq2 comp29722_c1_seq1 comp46357_c0_seq1 comp41479_c0_seq1 comp43455_c3_seq1 comp43501_c0_seq2 comp54807_c0_seq4 comp57861_c0_seq7 comp55887_c0_seq4 comp58098_c8_seq1 comp44203_c0_seq1 comp47295_c3_seq1 comp53112_c1_seq2 comp50444_c2_seq3 comp51919_c0_seq4 comp47993_c0_seq1 comp56395_c4_seq1 comp57991_c2_seq8 comp54722_c0_seq3 comp56739_c0_seq2 comp54391_c1_seq12 comp114060_c0_seq1 comp52941_c1_seq2 comp56490_c0_seq15 comp57115_c0_seq9 comp56641_c3_seq10 comp32647_c0_seq1 comp53402_c0_seq3 comp50756_c0_seq4 comp55411_c1_seq1 comp44506_c0_seq2 comp48844_c0_seq2 comp58083_c4_seq3 comp56952_c6_seq29 comp43579_c0_seq1 comp50415_c0_seq1 comp51012_c0_seq1 comp29795_c0_seq1 comp54274_c0_seq3 comp46202_c1_seq1 comp28005_c0_seq1 comp54401_c8_seq2 comp40519_c0_seq1 comp47561_c0_seq1 comp44520_c0_seq2 comp30453_c0_seq1 comp51145_c0_seq1 comp54549_c1_seq5 comp49904_c2_seq6 comp56467_c0_seq1 comp45761_c0_seq3 comp28842_c0_seq1 comp49409_c0_seq4 comp58238_c1_seq40 comp57445_c0_seq8 comp50140_c0_seq1 comp30960_c0_seq1 comp55345_c3_seq1 comp32202_c0_seq2 comp52564_c0_seq8 comp52096_c0_seq7 comp57188_c1_seq6 comp56968_c0_seq3 comp57664_c4_seq1 comp56540_c1_seq1 comp57046_c2_seq1 comp50841_c1_seq3 comp47785_c0_seq1 comp57533_c1_seq3 comp48052_c0_seq1 comp52768_c0_seq2 comp60148_c0_seq1 comp140031_c0_seq1 comp53807_c0_seq2 comp55231_c0_seq3 comp57881_c3_seq3 comp48743_c3_seq1 comp57084_c0_seq3 comp56737_c5_seq8 comp52601_c0_seq3 comp49691_c0_seq1 comp58041_c0_seq12 comp56700_c9_seq3 comp44115_c0_seq1 comp55818_c3_seq7 comp53595_c0_seq6 comp50546_c2_seq1 comp54586_c7_seq3 comp54241_c0_seq1 comp56487_c4_seq1 comp55998_c4_seq2 comp57373_c0_seq2 comp45800_c0_seq1 comp57419_c0_seq6 comp50839_c0_seq2 comp56868_c3_seq5 comp42517_c0_seq2 comp54469_c0_seq4 comp54509_c2_seq8 comp53051_c1_seq5 comp52086_c0_seq3 comp57051_c12_seq4 comp52330_c0_seq1 comp57309_c0_seq4 comp57178_c0_seq10 comp57171_c0_seq1 comp57030_c2_seq11 comp56173_c0_seq6 comp50395_c1_seq1 comp51541_c2_seq5 comp57682_c1_seq1 comp28834_c0_seq2 comp53948_c0_seq2 comp42088_c0_seq1 comp56780_c1_seq1 comp51106_c1_seq10 comp54957_c1_seq1 comp34747_c1_seq1 comp45015_c2_seq2 comp50414_c0_seq1 comp54958_c0_seq4 comp57675_c3_seq14 comp53194_c0_seq3 comp46515_c0_seq1 comp54872_c2_seq1 comp56212_c0_seq4 comp50421_c0_seq2 comp41525_c0_seq1 comp55219_c0_seq1 comp56302_c0_seq1 comp56047_c0_seq3 comp55866_c1_seq4 comp55146_c0_seq1 comp57516_c1_seq8 comp9492_c0_seq1 comp45183_c0_seq2 comp57937_c1_seq6 comp42389_c1_seq1 comp58221_c0_seq39 comp32158_c0_seq3 comp4856_c0_seq1 comp52089_c1_seq3 comp57629_c1_seq1 comp52835_c1_seq6 comp52807_c0_seq2 comp56906_c0_seq6 comp57807_c6_seq5 comp55091_c0_seq6 comp54863_c0_seq2 comp58270_c3_seq36 comp51576_c1_seq4 comp44704_c0_seq1 comp51639_c0_seq1 comp43387_c1_seq1 comp46246_c1_seq2 comp31636_c0_seq1 comp49516_c0_seq1 comp49134_c1_seq2 comp30290_c0_seq1 comp56641_c0_seq14 comp53858_c0_seq2 comp57626_c14_seq1 comp57721_c3_seq4 comp57270_c1_seq3 comp42783_c0_seq1 comp50055_c0_seq2 comp31551_c0_seq1 comp57664_c3_seq10 comp36071_c0_seq1 comp44313_c1_seq1 comp52557_c0_seq1 comp49341_c1_seq1 comp50890_c0_seq1 comp50672_c0_seq1 comp51618_c0_seq9 comp50852_c0_seq1 comp57755_c2_seq4 comp49889_c1_seq1 comp57547_c2_seq2 comp50455_c1_seq1 comp55255_c0_seq3 comp54497_c0_seq1 comp57651_c1_seq2 comp58128_c2_seq2 comp51987_c0_seq1 comp54704_c0_seq3 comp53018_c1_seq1 comp54866_c0_seq7 comp58080_c2_seq1 comp48429_c0_seq2 comp55762_c4_seq10 comp54590_c0_seq2 comp46195_c1_seq2 comp55422_c0_seq1 comp53901_c1_seq2 comp32661_c0_seq1 comp53116_c0_seq3 comp56059_c0_seq6 comp51972_c0_seq1 comp51304_c1_seq1 comp53695_c1_seq2 comp50506_c0_seq3 comp39927_c0_seq1 comp57560_c0_seq1 comp51990_c0_seq1 comp54398_c0_seq8 comp53724_c0_seq6 comp56140_c0_seq3 comp52494_c0_seq3 comp56559_c0_seq7 comp64828_c0_seq1 comp57028_c0_seq3 comp51179_c0_seq1 comp44761_c1_seq2 comp46189_c0_seq1 comp58215_c2_seq6 comp30098_c0_seq1 comp50839_c0_seq6 comp50039_c0_seq1 comp53380_c0_seq2 comp57576_c0_seq1 comp49636_c0_seq2 comp43416_c0_seq1 comp46956_c1_seq1 comp52603_c0_seq2 comp56825_c1_seq4 comp57728_c2_seq1 comp52244_c0_seq1 comp56735_c0_seq12 comp46844_c0_seq1 comp56689_c2_seq3 comp47879_c0_seq1 comp58145_c0_seq17 comp53335_c0_seq11 comp55400_c0_seq3 comp52665_c1_seq1 comp57704_c1_seq2 comp56748_c2_seq1 comp58201_c0_seq52 comp54867_c0_seq1 comp56496_c0_seq3 comp49966_c0_seq2 comp57783_c0_seq8 comp52804_c0_seq3 comp57544_c1_seq1 comp56785_c5_seq4 comp56909_c1_seq2 comp52044_c2_seq1 comp54698_c1_seq19 comp56043_c0_seq3 comp54849_c2_seq1 comp56449_c6_seq1 comp58141_c0_seq1 comp51753_c1_seq1 comp29054_c0_seq1 comp55267_c0_seq6 comp53842_c1_seq6 comp55946_c0_seq4 comp54367_c1_seq4 comp41013_c0_seq1 comp51103_c2_seq1 comp50614_c0_seq2 comp54927_c4_seq3 comp54736_c0_seq1 comp55328_c0_seq2 comp52657_c0_seq1 comp41487_c0_seq4 comp54153_c0_seq3 comp54726_c0_seq2 comp51071_c0_seq2 comp58063_c0_seq3 comp46981_c1_seq2 comp53222_c0_seq2 comp57982_c3_seq4 comp54814_c0_seq1 comp46405_c1_seq1 comp57273_c1_seq6 comp57518_c10_seq4 comp48246_c1_seq1 comp54687_c1_seq2 comp54846_c0_seq2 comp56648_c1_seq4 comp40050_c0_seq1 comp50477_c0_seq1 comp57457_c2_seq9 comp57580_c0_seq4 comp56470_c1_seq3 comp55878_c0_seq1 comp50435_c0_seq3 comp27717_c0_seq1 comp57488_c3_seq14 comp55026_c0_seq6 comp54325_c0_seq1 comp52022_c3_seq2 comp56284_c0_seq3 comp50069_c0_seq2 comp53210_c0_seq3 comp58448_c0_seq1 comp50953_c0_seq1 comp55640_c5_seq25 comp55066_c0_seq10 comp57796_c0_seq5 comp55674_c0_seq2 comp57795_c0_seq5 comp50109_c0_seq1 comp56367_c0_seq2 comp57528_c8_seq1 comp46200_c0_seq2 comp46036_c1_seq2 comp58265_c2_seq11 comp56211_c1_seq6 comp57524_c0_seq1 comp53306_c0_seq1 comp57231_c2_seq1 comp51832_c0_seq1 comp56404_c0_seq12 comp54109_c0_seq1 comp53882_c0_seq2 comp56054_c6_seq3 comp41541_c0_seq2 comp53249_c1_seq1 comp57997_c4_seq34 comp55707_c1_seq2 comp49218_c1_seq4 comp52878_c0_seq4 comp56934_c0_seq8 comp29339_c0_seq1 comp56283_c1_seq6 comp49139_c0_seq2 comp53259_c1_seq8 comp29533_c0_seq1 comp49862_c2_seq1 comp52062_c0_seq3 comp50204_c0_seq4 comp47476_c0_seq1 comp58036_c1_seq11 comp52524_c0_seq9 comp57566_c15_seq15 comp56942_c0_seq5 comp54976_c4_seq4 comp47398_c2_seq1 comp55469_c0_seq3 comp56607_c0_seq4 comp57247_c0_seq2 comp47266_c0_seq1 comp48894_c0_seq1 comp49523_c0_seq1 comp53423_c1_seq6 comp54947_c2_seq5 comp55736_c1_seq11 comp58943_c0_seq1 comp56083_c0_seq5 comp57572_c1_seq11 comp56139_c0_seq5 comp56641_c0_seq4 comp57578_c1_seq35 comp56244_c1_seq2 comp54550_c0_seq1 comp36436_c0_seq1 comp56696_c2_seq1 comp51232_c0_seq2 comp57427_c1_seq4 comp53650_c4_seq1 comp56884_c1_seq3 comp48441_c1_seq1 comp51495_c0_seq2 comp54417_c0_seq3 comp48599_c2_seq1 comp54971_c0_seq1 comp56227_c1_seq7 comp56803_c0_seq1 comp55940_c0_seq1 comp56759_c2_seq5 comp55589_c3_seq3 comp52285_c0_seq2 comp29275_c0_seq1 comp46894_c1_seq1 comp57524_c0_seq11 comp29410_c0_seq1 comp53047_c1_seq1 comp32090_c0_seq1 comp56670_c0_seq2 comp62235_c0_seq1 comp53779_c2_seq15 comp55275_c0_seq1 comp58262_c1_seq1 comp55859_c7_seq1 comp49532_c0_seq2 comp48400_c0_seq1 comp56342_c0_seq1 comp58171_c0_seq1 comp51892_c1_seq3 comp49401_c0_seq1 comp52674_c0_seq1 comp54619_c1_seq15 comp48476_c1_seq1 comp52742_c0_seq2 comp52590_c0_seq1 comp56341_c3_seq9 comp52582_c6_seq6 comp56798_c1_seq1 comp58154_c2_seq11 comp58038_c1_seq10 comp58237_c5_seq15 comp53964_c1_seq3 comp56499_c0_seq3 comp57588_c7_seq1 comp57919_c0_seq1 comp55223_c0_seq2 comp56617_c5_seq4 comp56422_c1_seq6 comp56182_c0_seq12 comp57023_c1_seq26 comp44796_c1_seq1 comp46774_c1_seq1 comp51050_c0_seq1 comp53393_c1_seq1 comp48551_c4_seq1 comp57255_c0_seq3 comp49768_c0_seq2 comp58001_c3_seq9 comp52567_c1_seq2 comp52641_c0_seq1 comp52850_c0_seq4 comp57397_c9_seq45 comp52890_c1_seq1 comp56442_c1_seq1 comp46493_c0_seq1 comp55398_c2_seq4 comp54490_c0_seq4 comp68671_c0_seq1 comp50896_c1_seq3 comp55640_c5_seq15 comp47298_c0_seq1 comp33552_c0_seq1 comp50286_c0_seq1 comp54136_c0_seq2 comp33042_c0_seq1 comp58040_c4_seq1 comp56610_c3_seq5 comp52977_c0_seq3 comp50858_c0_seq1 comp50726_c0_seq3 comp46346_c0_seq3 comp51098_c0_seq2 comp57804_c2_seq6 comp30258_c0_seq1 comp46609_c0_seq1 comp55465_c1_seq1 comp29577_c1_seq1 comp56120_c0_seq5 comp48750_c1_seq1 comp45262_c2_seq1 comp46873_c1_seq1 comp54371_c0_seq1 comp2628_c0_seq1 comp53518_c0_seq1 comp54387_c1_seq7 comp52860_c0_seq6 comp45648_c2_seq1 comp40259_c0_seq1 comp51300_c0_seq11 comp57912_c0_seq6 comp46105_c0_seq4 comp50249_c0_seq3 comp57288_c2_seq5 comp58122_c0_seq8 comp54181_c1_seq3 comp48886_c0_seq4 comp48697_c0_seq3 comp57494_c7_seq4 comp57808_c0_seq1 comp53424_c2_seq5 comp57122_c0_seq8 comp57617_c7_seq6 comp53196_c0_seq1 comp57280_c2_seq2 comp57665_c1_seq2 comp28833_c0_seq2 comp54857_c0_seq1 comp55312_c0_seq18 comp46439_c0_seq1 comp53407_c0_seq1 comp56008_c0_seq3 comp56701_c0_seq3 comp30480_c0_seq1 comp32931_c0_seq3 comp51007_c1_seq1 comp46537_c2_seq1 comp54238_c0_seq2 comp48362_c1_seq1 comp49030_c0_seq1 comp55952_c2_seq2 comp52086_c0_seq1 comp58301_c1_seq8 comp56764_c1_seq1 comp56427_c1_seq1 comp49004_c0_seq1 comp47533_c0_seq1 comp55950_c0_seq3 comp56861_c1_seq1 comp54500_c4_seq1 comp56812_c0_seq5 comp55786_c0_seq4 comp53769_c5_seq1 comp51371_c0_seq2 comp57565_c0_seq2 comp58274_c2_seq9 comp57663_c0_seq5 comp58071_c3_seq1 comp57660_c0_seq2 comp57704_c3_seq8 comp57110_c1_seq4 comp53935_c1_seq3 comp50255_c1_seq2 comp49928_c0_seq3 comp95871_c0_seq1 comp53537_c1_seq2 comp57306_c1_seq1 comp29371_c0_seq1 comp32155_c0_seq1 comp51133_c0_seq2 comp48319_c0_seq2 comp57705_c4_seq1 comp29541_c0_seq1 comp57509_c3_seq2 comp55647_c0_seq3 comp51575_c0_seq3 comp55969_c0_seq1 comp47247_c0_seq2 comp55900_c8_seq16 comp52784_c0_seq2 comp57466_c7_seq2 comp48375_c0_seq3 comp29647_c0_seq2 comp56554_c1_seq1 comp55625_c0_seq1 comp44447_c1_seq1 comp57099_c0_seq4 comp58239_c2_seq17 comp51339_c0_seq2 comp55649_c0_seq7 comp51959_c0_seq1 comp54231_c0_seq2 comp56140_c2_seq1 comp47523_c1_seq1 comp57594_c4_seq4 comp56371_c0_seq1 comp56531_c0_seq20 comp50378_c1_seq5 comp31248_c1_seq2 comp56236_c0_seq1 comp58377_c0_seq1 comp33258_c1_seq1 comp31043_c0_seq1 comp54745_c1_seq2 comp55788_c0_seq3 comp56332_c0_seq1 comp57639_c12_seq3 comp56072_c0_seq1 comp47454_c0_seq1 comp54993_c0_seq1 comp57145_c3_seq6 comp57848_c0_seq19 comp49494_c0_seq2 comp58231_c1_seq13 comp57831_c2_seq13 comp56783_c2_seq2 comp56042_c0_seq14 comp57424_c3_seq1 comp54817_c9_seq1 comp57658_c6_seq6 comp53525_c0_seq5 comp57528_c4_seq2 comp57994_c0_seq28 comp45169_c1_seq1 comp48185_c2_seq1 comp48572_c0_seq1 comp57588_c9_seq1 comp30502_c0_seq1 comp48144_c0_seq1 comp55175_c0_seq2 comp35160_c0_seq1 comp42888_c0_seq2 comp38556_c0_seq1 comp50390_c0_seq1 comp49367_c0_seq1 comp2454_c0_seq1 comp57507_c0_seq43 comp54304_c0_seq1 comp50938_c0_seq3 comp30114_c0_seq1 comp56438_c3_seq6 comp52103_c0_seq5 comp55894_c0_seq2 comp45952_c0_seq2 comp47816_c0_seq1 comp58061_c0_seq21 comp50882_c2_seq1 comp51561_c0_seq2 comp58190_c2_seq7 comp54915_c0_seq2 comp49388_c0_seq1 comp48091_c1_seq2 comp54579_c0_seq1 comp53930_c0_seq1 comp54908_c2_seq1 comp52789_c1_seq1 comp54047_c0_seq2 comp43934_c0_seq1 comp51360_c1_seq7 comp51490_c0_seq2 comp53049_c1_seq4 comp53905_c0_seq1 comp56832_c7_seq6 comp33901_c0_seq1 comp48006_c0_seq1 comp52282_c0_seq2 comp42379_c0_seq1 comp47509_c0_seq1 comp48948_c0_seq1 comp54471_c0_seq5 comp50860_c1_seq1 comp46892_c0_seq1 comp50966_c0_seq5 comp139486_c0_seq1 comp53915_c1_seq1 comp56925_c1_seq1 comp45625_c0_seq1 comp53540_c2_seq1 comp56383_c0_seq1 comp57083_c1_seq5 comp56601_c0_seq2 comp48950_c0_seq2 comp57634_c5_seq5 comp55953_c0_seq10 comp56197_c1_seq4 comp56385_c0_seq5 comp54892_c0_seq5 comp55343_c1_seq11 comp56301_c0_seq2 comp57604_c0_seq8 comp52577_c0_seq1 comp38306_c0_seq1 comp51654_c0_seq2 comp46395_c0_seq1 comp32618_c0_seq1 comp49687_c0_seq1 comp58041_c0_seq3 comp57363_c1_seq1 comp51303_c1_seq2 comp54703_c2_seq6 comp45743_c1_seq1 comp43724_c0_seq2 comp47758_c0_seq1 comp55569_c0_seq7 comp49140_c0_seq1 comp55784_c0_seq3 comp50740_c0_seq4 comp50407_c1_seq1 comp31422_c0_seq1 comp51200_c0_seq1 comp57922_c2_seq1 comp56196_c2_seq6 comp37350_c1_seq2 comp53537_c1_seq1 comp55552_c0_seq10 comp53132_c0_seq10 comp56953_c2_seq6 comp50745_c0_seq2 comp47734_c0_seq3 comp53659_c0_seq1 comp53001_c0_seq3 comp57626_c8_seq4 comp58071_c2_seq6 comp49936_c0_seq2 comp54790_c3_seq1 comp54677_c0_seq1 comp57323_c1_seq2 comp53491_c0_seq4 comp49854_c0_seq2 comp53925_c0_seq1 comp50900_c0_seq1 comp50120_c1_seq1 comp58285_c1_seq3 comp57629_c3_seq13 comp57711_c0_seq4 comp53955_c3_seq2 comp57753_c3_seq3 comp42693_c0_seq1 comp45385_c0_seq2 comp55582_c1_seq5 comp57863_c5_seq1 comp57935_c1_seq7 comp49964_c0_seq2 comp58202_c2_seq6 comp56357_c4_seq4 comp56531_c0_seq19 comp56678_c0_seq1 comp57984_c3_seq8 comp56431_c1_seq4 comp58041_c0_seq7 comp55381_c1_seq1 comp56835_c0_seq2 comp52815_c0_seq2 comp55807_c1_seq4 comp53958_c4_seq2 comp54537_c0_seq2 comp46376_c2_seq1 comp56060_c2_seq1 comp55008_c0_seq3 comp55622_c0_seq1 comp50352_c0_seq4 comp58147_c2_seq1 comp31941_c0_seq1 comp57207_c0_seq9 comp44519_c0_seq2 comp58982_c0_seq1 comp55039_c0_seq1 comp47953_c2_seq1 comp47441_c0_seq2 comp51635_c1_seq1 comp58180_c3_seq11 comp50420_c0_seq3 comp55122_c1_seq6 comp46144_c0_seq1 comp28505_c0_seq1 comp44015_c0_seq1 comp42806_c1_seq1 comp43801_c1_seq1 comp55850_c1_seq2 comp58108_c1_seq9 comp56922_c2_seq7 comp57500_c2_seq7 comp55944_c0_seq8 comp57357_c0_seq4 comp56961_c3_seq17 comp45914_c0_seq4 comp52519_c0_seq4 comp54287_c2_seq1 comp57830_c4_seq6 comp31308_c0_seq1 comp49399_c2_seq1 comp53227_c1_seq1 comp54266_c0_seq10 comp55790_c3_seq5 comp57924_c0_seq1 comp58286_c1_seq26 comp31443_c0_seq1 comp44181_c0_seq1 comp53202_c4_seq4 comp51215_c0_seq2 comp58183_c1_seq2 comp54750_c4_seq1 comp57999_c1_seq9 comp53805_c0_seq1 comp52412_c3_seq2 comp56994_c1_seq1 comp57612_c0_seq1 comp51897_c0_seq3 comp51668_c0_seq2 comp31986_c0_seq2 comp50957_c1_seq1 comp54757_c0_seq2 comp57544_c6_seq9 comp28826_c0_seq1 comp57253_c0_seq5 comp57036_c0_seq5 comp48443_c0_seq4 comp48726_c0_seq1 comp55739_c4_seq1 comp51154_c0_seq3 comp50009_c0_seq3 comp44999_c0_seq5 comp47237_c1_seq1 comp52182_c1_seq1 comp54670_c2_seq2 comp53899_c0_seq2 comp57443_c11_seq4 comp399139_c0_seq1 comp53868_c1_seq1 comp49759_c1_seq3 comp31563_c0_seq2 comp57031_c1_seq5 comp50012_c1_seq1 comp52300_c0_seq1 comp50939_c0_seq3 comp55550_c0_seq2 comp57114_c0_seq1 comp56654_c3_seq5 comp36565_c1_seq1 comp57829_c0_seq83 comp58420_c0_seq1 comp47071_c0_seq4 comp43774_c0_seq1 comp60810_c0_seq1 comp58826_c0_seq1 comp42674_c0_seq1 comp51585_c0_seq1 comp55637_c0_seq5 comp54885_c0_seq2 comp43932_c0_seq1 comp57502_c6_seq8 comp56166_c1_seq3 comp57210_c2_seq8 comp54977_c0_seq3 comp49465_c0_seq1 comp45701_c0_seq1 comp56813_c3_seq6 comp52715_c0_seq1 comp54319_c0_seq1 comp50340_c2_seq2 comp51902_c0_seq1 comp50474_c0_seq4 comp57573_c0_seq1 comp56268_c0_seq1 comp55678_c0_seq2 comp58296_c0_seq24 comp49075_c0_seq1 comp47842_c0_seq1 comp51137_c1_seq1 comp57810_c1_seq2 comp28964_c1_seq1 comp50021_c0_seq2 comp53657_c0_seq4 comp53618_c2_seq2 comp56690_c0_seq4 comp50377_c1_seq1 comp57487_c5_seq5 comp52176_c0_seq1 comp57618_c0_seq1 comp53567_c1_seq3 comp57486_c4_seq1 comp38389_c1_seq1 comp32806_c0_seq1 comp56121_c0_seq5 comp58168_c3_seq1 comp56797_c2_seq6 comp56987_c0_seq2 comp4780_c0_seq1 comp53941_c1_seq1 comp48997_c2_seq1 comp47878_c0_seq6 comp58098_c2_seq22 comp27732_c0_seq1 comp56058_c1_seq1 comp51143_c1_seq1 comp56578_c0_seq5 comp57061_c0_seq3 comp57672_c1_seq2 comp57730_c2_seq4 comp51521_c3_seq1 comp53194_c0_seq2 comp56202_c0_seq1 comp57614_c0_seq5 comp49770_c5_seq1 comp52548_c2_seq1 comp51552_c0_seq2 comp43454_c0_seq1 comp53750_c1_seq6 comp51273_c0_seq1 comp54336_c3_seq3 comp56138_c0_seq1 comp57007_c4_seq1 comp54120_c5_seq1 comp49924_c1_seq1 comp54371_c0_seq2 comp55439_c0_seq2 comp57494_c7_seq5 comp56159_c0_seq4 comp56722_c1_seq11 comp55048_c0_seq4 comp54830_c7_seq2 comp51135_c0_seq1 comp56741_c0_seq2 comp50669_c0_seq2 comp51863_c3_seq3 comp47616_c0_seq1 comp58193_c1_seq1 comp48358_c0_seq1 comp54691_c0_seq2 comp54139_c0_seq4 comp50105_c0_seq1 comp38216_c0_seq1 comp53448_c1_seq4 comp4194_c0_seq1 comp29668_c0_seq1 comp41706_c1_seq1 comp53434_c0_seq1 comp55507_c0_seq1 comp51957_c5_seq2 comp57123_c0_seq9 comp57927_c3_seq15 comp56374_c0_seq3 comp44420_c0_seq1 comp57178_c0_seq1 comp57826_c6_seq11 comp50027_c1_seq1 comp31184_c0_seq1 comp49135_c0_seq1 comp44906_c0_seq3 comp57975_c2_seq12 comp52882_c2_seq4 comp54430_c0_seq2 comp56381_c0_seq5 comp57704_c3_seq2 comp55560_c3_seq1 comp45183_c0_seq1 comp53533_c1_seq6 comp53613_c1_seq2 comp52943_c1_seq2 comp56217_c0_seq4 comp53135_c2_seq5 comp53894_c0_seq3 comp42362_c0_seq1 comp57444_c0_seq16 comp42949_c1_seq1 comp54407_c0_seq3 comp56113_c0_seq2 comp56656_c4_seq2 comp54094_c0_seq1 comp39608_c0_seq1 comp52982_c0_seq5 comp56636_c2_seq1 comp58257_c0_seq17 comp49657_c1_seq1 comp53412_c5_seq2 comp51893_c1_seq3 comp57825_c2_seq11 comp31596_c0_seq1 comp57649_c3_seq6 comp53538_c7_seq2 comp56221_c0_seq1 comp54373_c2_seq1 comp54789_c1_seq1 comp50834_c0_seq1 comp48347_c5_seq1 comp48195_c0_seq2 comp55387_c0_seq2 comp52968_c0_seq8 comp55436_c5_seq4 comp39257_c0_seq1 comp32476_c0_seq3 comp49737_c0_seq3 comp32455_c0_seq1 comp59592_c0_seq1 comp54326_c0_seq3 comp53591_c0_seq3 comp56095_c3_seq1 comp49309_c1_seq1 comp58059_c0_seq1 comp55574_c0_seq4 comp53410_c0_seq8 comp57031_c1_seq14 comp42048_c0_seq1 comp51744_c0_seq1 comp32613_c0_seq1 comp29239_c0_seq1 comp51553_c0_seq3 comp57424_c0_seq7 comp30239_c0_seq1 comp30740_c0_seq1 comp54378_c10_seq1 comp52454_c1_seq3 comp53631_c0_seq3 comp47103_c1_seq1 comp48283_c0_seq1 comp54889_c0_seq1 comp51955_c0_seq1 comp56624_c0_seq3 comp51618_c0_seq8 comp55585_c1_seq3 comp50915_c0_seq1 comp50730_c0_seq1 comp50239_c0_seq1 comp55375_c2_seq3 comp55430_c2_seq11 comp46888_c0_seq1 comp42123_c0_seq1 comp30882_c0_seq1 comp57740_c2_seq13 comp57127_c0_seq4 comp57639_c1_seq4 comp51302_c0_seq2 comp50700_c3_seq1 comp55167_c0_seq4 comp57270_c3_seq1 comp56060_c1_seq3 comp58253_c1_seq30 comp54881_c0_seq4 comp50588_c0_seq1 comp32792_c0_seq1 comp73659_c0_seq1 comp45888_c1_seq1 comp57937_c1_seq7 comp54378_c4_seq17 comp48795_c0_seq1 comp56441_c1_seq4 comp56811_c0_seq1 comp55144_c0_seq2 comp52372_c1_seq1 comp55351_c5_seq3 comp52600_c0_seq2 comp55266_c3_seq1 comp55137_c2_seq9 comp54229_c0_seq2 comp53233_c0_seq1 comp31125_c0_seq1 comp50164_c0_seq1 comp57985_c3_seq11 comp58272_c2_seq6 comp57927_c1_seq1 comp43196_c0_seq1 comp56158_c0_seq4 comp56744_c1_seq2 comp54203_c2_seq3 comp48962_c0_seq1 comp44571_c0_seq1 comp58223_c6_seq15 comp56536_c0_seq1 comp53793_c0_seq1 comp31524_c0_seq1 comp55832_c0_seq2 comp46524_c0_seq2 comp28282_c0_seq1 comp53583_c1_seq3 comp55083_c0_seq7 comp58255_c1_seq1 comp58228_c1_seq1 comp51076_c0_seq1 comp50660_c0_seq2 comp54194_c0_seq4 comp46589_c0_seq1 comp52367_c0_seq3 comp49493_c1_seq1 comp57571_c0_seq2 comp57605_c2_seq8 comp50336_c0_seq5 comp42243_c0_seq2 comp55037_c0_seq8 comp58215_c2_seq1 comp49390_c0_seq2 comp47518_c0_seq2 comp44254_c0_seq1 comp56049_c0_seq6 comp56664_c0_seq2 comp56816_c0_seq3 comp53051_c0_seq1 comp56436_c0_seq2 comp53603_c0_seq1 comp55029_c1_seq1 comp56630_c1_seq3 comp52252_c0_seq4 comp120597_c0_seq1 comp57705_c1_seq2 comp53595_c0_seq3 comp57116_c4_seq24 comp50751_c0_seq1 comp32005_c0_seq1 comp54699_c0_seq2 comp55385_c0_seq3 comp52837_c0_seq1 comp57073_c1_seq16 comp58173_c0_seq1 comp55600_c3_seq5 comp52518_c0_seq1 comp47575_c0_seq1 comp51117_c1_seq3 comp50700_c0_seq1 comp55973_c0_seq1 comp48541_c0_seq3 comp57591_c2_seq1 comp52532_c0_seq4 comp52411_c2_seq2 comp57015_c0_seq1 comp57190_c0_seq7 comp53891_c0_seq3 comp54099_c0_seq3 comp52125_c0_seq6 comp47910_c0_seq5 comp56698_c0_seq4 comp53760_c1_seq2 comp50805_c1_seq1 comp49820_c0_seq1 comp50922_c0_seq1 comp46611_c0_seq1 comp55712_c0_seq1 comp48387_c1_seq1 comp35477_c1_seq1 comp52638_c1_seq2 comp46173_c0_seq3 comp57665_c1_seq16 comp56940_c1_seq5 comp53187_c0_seq5 comp60200_c0_seq1 comp58504_c0_seq1 comp57317_c3_seq22 comp57433_c0_seq4 comp55787_c2_seq4 comp31843_c0_seq1 comp51608_c0_seq3 comp56632_c2_seq9 comp51532_c0_seq5 comp54632_c0_seq12 comp52036_c0_seq1 comp54549_c1_seq2 comp55857_c5_seq11 comp51952_c0_seq1 comp48269_c0_seq4 comp54193_c6_seq5 comp55827_c2_seq3 comp55080_c1_seq1 comp57597_c0_seq7 comp48317_c0_seq1 comp51882_c0_seq8 comp52670_c0_seq1 comp56659_c2_seq5 comp52183_c0_seq1 comp50652_c1_seq4 comp53358_c0_seq1 comp45169_c0_seq1 comp50361_c0_seq1 comp56724_c1_seq3 comp50962_c0_seq1 comp54052_c0_seq4 comp55491_c3_seq15 comp39660_c0_seq1 comp57985_c3_seq2 comp55818_c4_seq1 comp54250_c2_seq2 comp57001_c3_seq9 comp49971_c1_seq1 comp57084_c0_seq1 comp55042_c1_seq8 comp58198_c4_seq16 comp49668_c2_seq1 comp55227_c0_seq1 comp58026_c0_seq3 comp44295_c0_seq2 comp51474_c0_seq1 comp52061_c0_seq1 comp58045_c0_seq24 comp56230_c14_seq3 comp51770_c1_seq2 comp54139_c4_seq2 comp52628_c0_seq4 comp46860_c0_seq1 comp44576_c0_seq1 comp57223_c0_seq5 comp55403_c1_seq11 comp46391_c0_seq1 comp29987_c0_seq1 comp58121_c0_seq6 comp43451_c0_seq1 comp51334_c0_seq1 comp58253_c1_seq23 comp53452_c1_seq5 comp57394_c1_seq6 comp44619_c0_seq1 comp58291_c0_seq5 comp57673_c1_seq1 comp51097_c2_seq6 comp58292_c0_seq12 comp56778_c4_seq2 comp50413_c0_seq1 comp51333_c0_seq2 comp50113_c0_seq2 comp55944_c0_seq2 comp52139_c0_seq3 comp54543_c1_seq1 comp56672_c1_seq12 comp56291_c1_seq1 comp38366_c0_seq1 comp51801_c1_seq5 comp55908_c1_seq2 comp50473_c0_seq1 comp56193_c5_seq1 comp54514_c0_seq1 comp54227_c0_seq1 comp54545_c0_seq2 comp55883_c2_seq9 comp50945_c0_seq1 comp52779_c0_seq1 comp55317_c0_seq2 comp44682_c1_seq1 comp50540_c4_seq3 comp52845_c3_seq2 comp55641_c0_seq1 comp50289_c0_seq2 comp57023_c1_seq9 comp33168_c0_seq1 comp54953_c1_seq1 comp53170_c2_seq2 comp57489_c0_seq10 comp29579_c0_seq1 comp51929_c0_seq4 comp53716_c0_seq4 comp56902_c0_seq1 comp50619_c0_seq1 comp54850_c0_seq4 comp59645_c0_seq1 comp46985_c2_seq1 comp43415_c0_seq3 comp57268_c0_seq1 comp50356_c1_seq8 comp56870_c0_seq4 comp44434_c0_seq2 comp55947_c1_seq5 comp46174_c1_seq1 comp55331_c1_seq6 comp56576_c1_seq5 comp42993_c1_seq1 comp54261_c0_seq1 comp57649_c4_seq1 comp32627_c0_seq1 comp53560_c1_seq1 comp52140_c0_seq1 comp52553_c2_seq8 comp55391_c0_seq1 comp47516_c1_seq2 comp50216_c0_seq1 comp41907_c0_seq2 comp57483_c0_seq8 comp50120_c0_seq1 comp55596_c0_seq2 comp57078_c0_seq5 comp48374_c0_seq1 comp49828_c0_seq2 comp56845_c0_seq4 comp52396_c0_seq1 comp53686_c0_seq3 comp55822_c4_seq2 comp41527_c0_seq1 comp57610_c5_seq1 comp45837_c0_seq1 comp56074_c0_seq4 comp56062_c2_seq3 comp51541_c2_seq4 comp59135_c0_seq1 comp56957_c4_seq4 comp54924_c0_seq4 comp57372_c1_seq1 comp52844_c0_seq2 comp45473_c0_seq3 comp56688_c2_seq24 comp45721_c0_seq1 comp57363_c1_seq8 comp47946_c1_seq1 comp54401_c8_seq6 comp56988_c0_seq11 comp42507_c0_seq1 comp55778_c1_seq1 comp55810_c0_seq3 comp30515_c1_seq1 comp49425_c0_seq2 comp55847_c0_seq7 comp32457_c0_seq1 comp50349_c1_seq3 comp31900_c0_seq2 comp55224_c0_seq2 comp57049_c2_seq1 comp57259_c0_seq7 comp55775_c3_seq6 comp55548_c1_seq2 comp52172_c0_seq2 comp28077_c0_seq1 comp51423_c1_seq4 comp54082_c0_seq4 comp56597_c0_seq1 comp52961_c0_seq3 comp55203_c1_seq1 comp52771_c0_seq6 comp32739_c0_seq1 comp58009_c2_seq22 comp57693_c0_seq4 comp55536_c0_seq1 comp54686_c0_seq10 comp140292_c0_seq1 comp52200_c4_seq1 comp58197_c7_seq13 comp49870_c0_seq3 comp54841_c7_seq1 comp54139_c0_seq1 comp56829_c1_seq15 comp56572_c9_seq2 comp49126_c0_seq1 comp57450_c1_seq4 comp49857_c0_seq2 comp51178_c0_seq1 comp56177_c9_seq1 comp48034_c0_seq1 comp57585_c0_seq4 comp56177_c2_seq1 comp59418_c0_seq1 comp49535_c1_seq2 comp52288_c0_seq1 comp48583_c0_seq1 comp52776_c0_seq1 comp47870_c0_seq3 comp46682_c0_seq1 comp51816_c2_seq4 comp27809_c0_seq1 comp55147_c0_seq5 comp50668_c1_seq1 comp46236_c1_seq2 comp52187_c0_seq7 comp53229_c0_seq1 comp53915_c0_seq3 comp49239_c0_seq1 comp51736_c0_seq2 comp52398_c0_seq9 comp53821_c2_seq3 comp44263_c0_seq1 comp56230_c14_seq19 comp117000_c0_seq1 comp56800_c1_seq1 comp53286_c2_seq2 comp52421_c0_seq6 comp58222_c2_seq10 comp29645_c0_seq1 comp58138_c2_seq17 comp54144_c0_seq1 comp29410_c1_seq1 comp50046_c0_seq1 comp58001_c3_seq11 comp50534_c0_seq1 comp54950_c2_seq3 comp43689_c0_seq3 comp55292_c0_seq1 comp56654_c2_seq8 comp52346_c0_seq2 comp58723_c0_seq1 comp57993_c0_seq2 comp54261_c4_seq1 comp53137_c1_seq1 comp31107_c0_seq3 comp53343_c1_seq2 comp49800_c0_seq1 comp57110_c1_seq3 comp55541_c1_seq8 comp56357_c4_seq19 comp31712_c0_seq1 comp55353_c1_seq11 comp57160_c1_seq5 comp43833_c0_seq1 comp54547_c2_seq1 comp52044_c4_seq1 comp56312_c1_seq5 comp56309_c1_seq12 comp46105_c0_seq1 comp52458_c1_seq1 comp50903_c0_seq2 comp54406_c0_seq1 comp55121_c4_seq1 comp37113_c0_seq1 comp76899_c0_seq1 comp54104_c0_seq1 comp57387_c0_seq4 comp48280_c0_seq1 comp51578_c0_seq1 comp49566_c0_seq3 comp54224_c3_seq2 comp52859_c0_seq5 comp56054_c6_seq8 comp52698_c0_seq3 comp50407_c2_seq1 comp57778_c1_seq1 comp52953_c0_seq3 comp57838_c0_seq10 comp43259_c0_seq1 comp32911_c0_seq1 comp55301_c0_seq1 comp58269_c1_seq1 comp44075_c0_seq1 comp32931_c0_seq2 comp44973_c0_seq1 comp33615_c0_seq1 comp57124_c0_seq1 comp56685_c0_seq4 comp54278_c0_seq1 comp55253_c4_seq3 comp56911_c3_seq7 comp3731_c0_seq1 comp49619_c0_seq1 comp55289_c0_seq2 comp33237_c0_seq2 comp57402_c2_seq1 comp57038_c1_seq1 comp47782_c0_seq1 comp57665_c1_seq1 comp55889_c1_seq1 comp57617_c7_seq9 comp54155_c1_seq5 comp57197_c1_seq1 comp58190_c2_seq4 comp32576_c0_seq1 comp52646_c0_seq1 comp57626_c15_seq1 comp35335_c0_seq1 comp49890_c1_seq1 comp38235_c0_seq2 comp54060_c0_seq1 comp54792_c0_seq2 comp50349_c1_seq7 comp56874_c4_seq27 comp55980_c1_seq6 comp57674_c1_seq3 comp57195_c2_seq3 comp36503_c0_seq1 comp52488_c0_seq3 comp47222_c0_seq3 comp54925_c0_seq7 comp56578_c2_seq4 comp48703_c3_seq3 comp56935_c0_seq3 comp55695_c0_seq4 comp45884_c0_seq1 comp48074_c0_seq2 comp54441_c0_seq1 comp57260_c0_seq8 comp51081_c0_seq1 comp52453_c0_seq1 comp55257_c0_seq2 comp54484_c0_seq1 comp52051_c1_seq1 comp57066_c0_seq4 comp49684_c0_seq3 comp47821_c0_seq6 comp54219_c2_seq1 comp48348_c0_seq2 comp54149_c0_seq1 comp45821_c0_seq1 comp54706_c1_seq8 comp57624_c2_seq10 comp49858_c0_seq2 comp50710_c1_seq1 comp52820_c1_seq2 comp56492_c0_seq1 comp54395_c0_seq2 comp54316_c0_seq1 comp57058_c0_seq15 comp48797_c0_seq1 comp51352_c0_seq4 comp46705_c1_seq1 comp42555_c0_seq1 comp57012_c0_seq5 comp51705_c2_seq1 comp55792_c0_seq4 comp56982_c0_seq5 comp56346_c0_seq13 comp49342_c0_seq1 comp28255_c0_seq1 comp49151_c0_seq2 comp57638_c2_seq2 comp57851_c5_seq4 comp54084_c1_seq1 comp55291_c0_seq3 comp40289_c1_seq1 comp37915_c0_seq1 comp30109_c0_seq1 comp58261_c4_seq41 comp53192_c1_seq1 comp52394_c0_seq4 comp57653_c0_seq2 comp57929_c1_seq5 comp54565_c0_seq1 comp57327_c2_seq7 comp56254_c0_seq4 comp57271_c0_seq3 comp52584_c0_seq4 comp49154_c0_seq2 comp50900_c0_seq2 comp55875_c1_seq5 comp58177_c5_seq15 comp35807_c0_seq1 comp46622_c0_seq1 comp32876_c0_seq2 comp50005_c0_seq2 comp52793_c3_seq1 comp51338_c0_seq6 comp51539_c0_seq1 comp45999_c0_seq1 comp53172_c0_seq2 comp49090_c0_seq1 comp58209_c0_seq13 comp50222_c1_seq1 comp32066_c0_seq1 comp87527_c0_seq1 comp52833_c0_seq6 comp50091_c0_seq1 comp55297_c0_seq3 comp53606_c0_seq4 comp154481_c0_seq1 comp53208_c0_seq1 comp57640_c1_seq7 comp56629_c1_seq2 comp53626_c0_seq5 comp48020_c3_seq1 comp57248_c0_seq2 comp53748_c0_seq12 comp47910_c0_seq4 comp57499_c2_seq1 comp56081_c0_seq2 comp56855_c1_seq2 comp47771_c0_seq1 comp54520_c1_seq2 comp41681_c0_seq1 comp57133_c0_seq12 comp54199_c1_seq1 comp54378_c2_seq3 comp50815_c1_seq5 comp37181_c0_seq1 comp39185_c0_seq1 comp56634_c5_seq7 comp52481_c0_seq1 comp54152_c1_seq1 comp46286_c0_seq1 comp57699_c1_seq12 comp55815_c1_seq1 comp56255_c13_seq2 comp57550_c0_seq5 comp49354_c1_seq1 comp48366_c0_seq1 comp54406_c6_seq4 comp47756_c0_seq1 comp52320_c0_seq3 comp46424_c0_seq3 comp51201_c0_seq3 comp49314_c0_seq1 comp51411_c0_seq1 comp46956_c1_seq2 comp29608_c0_seq1 comp57787_c2_seq7 comp57615_c1_seq3 comp53202_c4_seq11 comp29882_c0_seq1 comp44970_c2_seq1 comp55973_c1_seq1 comp52514_c0_seq4 comp56640_c0_seq2 comp57124_c4_seq2 comp57760_c2_seq1 comp55022_c0_seq2 comp49662_c0_seq2 comp50397_c0_seq3 comp50067_c2_seq1 comp57148_c6_seq3 comp56481_c2_seq8 comp59818_c0_seq1 comp49146_c0_seq2 comp46321_c0_seq2 comp49750_c1_seq3 comp52214_c1_seq4 comp58299_c3_seq11 comp48944_c0_seq2 comp56060_c1_seq8 comp51193_c0_seq1 comp53110_c2_seq19 comp57925_c1_seq8 comp57487_c5_seq21 comp52187_c0_seq3 comp52128_c0_seq3 comp35458_c1_seq2 comp45870_c1_seq4 comp42810_c1_seq1 comp55153_c3_seq1 comp50580_c0_seq1 comp57155_c1_seq1 comp52281_c0_seq2 comp42577_c0_seq3 comp54711_c1_seq13 comp30658_c1_seq1 comp31862_c0_seq2 comp45873_c0_seq2 comp58011_c4_seq1 comp56493_c1_seq1 comp32310_c0_seq1 comp51113_c0_seq1 comp58053_c2_seq5 comp52801_c0_seq2 comp54529_c0_seq2 comp56594_c7_seq6 comp54063_c0_seq4 comp57209_c1_seq1 comp49685_c0_seq1 comp55244_c0_seq1 comp170488_c0_seq1 comp51865_c0_seq5 comp55604_c4_seq1 comp58259_c1_seq6 comp48620_c0_seq2 comp57484_c0_seq3 comp56809_c4_seq2 comp55742_c0_seq9 comp43177_c0_seq1 comp57477_c0_seq6 comp50344_c4_seq1 comp47385_c0_seq1 comp54705_c0_seq1 comp58145_c0_seq14 comp56462_c1_seq9 comp55878_c2_seq3 comp43696_c0_seq2 comp50917_c0_seq1 comp58247_c0_seq6 comp51199_c2_seq1 comp57232_c1_seq11 comp57659_c1_seq8 comp53403_c0_seq9 comp54359_c2_seq7 comp57988_c4_seq20 comp54394_c4_seq2 comp56828_c0_seq5 comp31358_c0_seq1 comp50127_c2_seq1 comp56211_c1_seq1 comp58063_c0_seq33 comp42567_c0_seq1 comp46112_c0_seq1 comp50287_c3_seq2 comp57679_c2_seq1 comp53129_c3_seq1 comp53560_c2_seq2 comp57620_c3_seq22 comp58889_c0_seq1 comp55873_c0_seq8 comp54754_c0_seq1 comp53090_c0_seq2 comp56519_c0_seq6 comp57728_c2_seq8 comp29860_c0_seq1 comp45870_c0_seq1 comp54686_c0_seq8 comp54359_c2_seq11 comp50437_c0_seq3 comp56447_c6_seq1 comp55576_c2_seq7 comp51400_c1_seq2 comp49335_c0_seq1 comp50816_c0_seq1 comp57584_c0_seq3 comp53914_c1_seq1 comp51274_c0_seq2 comp50668_c0_seq1 comp58080_c2_seq4 comp49922_c1_seq2 comp52373_c0_seq1 comp48800_c1_seq1 comp38974_c3_seq1 comp50839_c0_seq9 comp54546_c1_seq1 comp56255_c7_seq4 comp51334_c0_seq3 comp48605_c0_seq2 comp57338_c1_seq6 comp54481_c0_seq2 comp41392_c0_seq1 comp54452_c0_seq1 comp28682_c0_seq2 comp52562_c0_seq2 comp28386_c0_seq1 comp40866_c0_seq1 comp53408_c1_seq2 comp57577_c3_seq2 comp55583_c1_seq2 comp57929_c1_seq10 comp47319_c1_seq1 comp60914_c0_seq1 comp51920_c0_seq1 comp53431_c2_seq15 comp57695_c1_seq6 comp54079_c0_seq4 comp53862_c1_seq2 comp53856_c0_seq1 comp58196_c0_seq2 comp56626_c4_seq1 comp56877_c0_seq13 comp44826_c1_seq2 comp54643_c0_seq2 comp2998_c0_seq1 comp51299_c2_seq2 comp56293_c0_seq3 comp55072_c0_seq3 comp57615_c1_seq8 comp49976_c0_seq4 comp49699_c1_seq1 comp57002_c1_seq2 comp56944_c0_seq3 comp47068_c0_seq4 comp57125_c0_seq1 comp56674_c1_seq4 comp50596_c0_seq2 comp56698_c0_seq10 comp52858_c0_seq1 comp44971_c0_seq1 comp43119_c0_seq2 comp56289_c0_seq2 comp55963_c1_seq2 comp57326_c5_seq11 comp46216_c4_seq1 comp43887_c0_seq2 comp51434_c0_seq1 comp58102_c1_seq4 comp51603_c0_seq3 comp57629_c3_seq10 comp57134_c1_seq3 comp53035_c0_seq4 comp51386_c0_seq2 comp53183_c2_seq1 comp53950_c0_seq5 comp57604_c0_seq5 comp55546_c0_seq2 comp56789_c1_seq3 comp49869_c0_seq1 comp57885_c1_seq63 comp50405_c0_seq3 comp56441_c1_seq11 comp54159_c1_seq4 comp56566_c1_seq9 comp58151_c0_seq7 comp56106_c2_seq3 comp48733_c1_seq1 comp56301_c0_seq1 comp45052_c0_seq9 comp52931_c0_seq5 comp53770_c0_seq2 comp53775_c2_seq1 comp54801_c0_seq4 comp58111_c3_seq50 comp56625_c1_seq12 comp57722_c0_seq5 comp56728_c7_seq5 comp31932_c0_seq1 comp53753_c1_seq3 comp57351_c3_seq8 comp54077_c0_seq4 comp53566_c0_seq2 comp46758_c0_seq2 comp42145_c3_seq1 comp33107_c0_seq1 comp56585_c1_seq4 comp49526_c0_seq1 comp53953_c0_seq5 comp49683_c1_seq1 comp56324_c0_seq3 comp57138_c4_seq2 comp58209_c0_seq2 comp55383_c2_seq5 comp57500_c2_seq2 comp53009_c0_seq1 comp54383_c0_seq1 comp53736_c0_seq1 comp46119_c0_seq1 comp46702_c0_seq2 comp49578_c1_seq1 comp58149_c1_seq7 comp46405_c1_seq4 comp55168_c4_seq1 comp54867_c1_seq2 comp52631_c0_seq7 comp57699_c1_seq9 comp57766_c1_seq1 comp46692_c0_seq1 comp55091_c0_seq3 comp58084_c0_seq8 comp49364_c0_seq1 comp47118_c0_seq1 comp53194_c1_seq1 comp53648_c1_seq1 comp47693_c0_seq1 comp33482_c0_seq2 comp49759_c1_seq2 comp57564_c2_seq42 comp47692_c0_seq1 comp56106_c4_seq3 comp57124_c1_seq15 comp50336_c0_seq1 comp50907_c0_seq2 comp46873_c2_seq1 comp45804_c0_seq2 comp48846_c0_seq1 comp50274_c0_seq2 comp52702_c0_seq3 comp49411_c2_seq1 comp57534_c1_seq1 comp54375_c0_seq4 comp56476_c1_seq7 comp57020_c0_seq5 comp43549_c0_seq1 comp47253_c0_seq1 comp56560_c0_seq1 comp58045_c0_seq21 comp53951_c0_seq2 comp51288_c0_seq1 comp57545_c1_seq14 comp48278_c0_seq2 comp53803_c1_seq4 comp49482_c0_seq1 comp49188_c0_seq1 comp58249_c1_seq2 comp55744_c12_seq5 comp47957_c0_seq1 comp50038_c0_seq2 comp56091_c1_seq2 comp58256_c1_seq22 comp55738_c0_seq6 comp56384_c0_seq5 comp29433_c0_seq1 comp51347_c1_seq1 comp32275_c0_seq1 comp56564_c1_seq6 comp29019_c0_seq1 comp59760_c0_seq1 comp43468_c1_seq1 comp45328_c0_seq1 comp31909_c0_seq1 comp51443_c0_seq2 comp53018_c1_seq4 comp51800_c0_seq1 comp57254_c7_seq2 comp56140_c2_seq2 comp49892_c4_seq4 comp55976_c1_seq4 comp56139_c0_seq2 comp50419_c0_seq1 comp56800_c1_seq3 comp58247_c0_seq26 comp55356_c0_seq1 comp50998_c1_seq3 comp56424_c0_seq1 comp57238_c0_seq8 comp58358_c0_seq1 comp55515_c0_seq2 comp46130_c1_seq1 comp52575_c0_seq1 comp53903_c4_seq1 comp37206_c0_seq1 comp42133_c1_seq3 comp49913_c0_seq2 comp45362_c0_seq2 comp52037_c0_seq1 comp51873_c0_seq1 comp57536_c2_seq3 comp37446_c0_seq1 comp37919_c1_seq1 comp50986_c0_seq1 comp56086_c1_seq8 comp51805_c0_seq3 comp228679_c0_seq1 comp56542_c1_seq4 comp58095_c0_seq3 comp49453_c0_seq3 comp47144_c0_seq3 comp114142_c0_seq1 comp57753_c3_seq16 comp51801_c1_seq7 comp56182_c0_seq10 comp53381_c1_seq4 comp55831_c2_seq5 comp47613_c0_seq1 comp51551_c1_seq1 comp56536_c0_seq6 comp51518_c1_seq2 comp46222_c2_seq1 comp47155_c0_seq1 comp56618_c0_seq15 comp49771_c0_seq3 comp51667_c3_seq5 comp51109_c0_seq3 comp53698_c0_seq2 comp50108_c0_seq1 comp33024_c1_seq1 comp57745_c0_seq2 comp55781_c0_seq1 comp53019_c0_seq1 comp50053_c0_seq1 comp54724_c2_seq4 comp58083_c3_seq1 comp55084_c0_seq9 comp45805_c0_seq1 comp55859_c8_seq1 comp47751_c1_seq1 comp56245_c1_seq4 comp52564_c0_seq2 comp60028_c0_seq1 comp52567_c0_seq4 comp52262_c0_seq2 comp53653_c0_seq1 comp56328_c0_seq1 comp49773_c0_seq1 comp31662_c0_seq1 comp46433_c0_seq1 comp58103_c2_seq12 comp30362_c0_seq1 comp56953_c2_seq10 comp49460_c0_seq3 comp56767_c0_seq5 comp43288_c0_seq1 comp56032_c11_seq13 comp50988_c2_seq1 comp55707_c1_seq10 comp45688_c0_seq4 comp54909_c1_seq5 comp56447_c2_seq3 comp46228_c0_seq2 comp28861_c0_seq1 comp58300_c0_seq14 comp45126_c0_seq1 comp52746_c0_seq2 comp53725_c0_seq5 comp55575_c2_seq7 comp55327_c2_seq3 comp50347_c3_seq1 comp53731_c2_seq1 comp54932_c1_seq1 comp48725_c0_seq2 comp46243_c0_seq1 comp54605_c2_seq3 comp42691_c0_seq1 comp55117_c0_seq1 comp51983_c0_seq1 comp56304_c8_seq1 comp53182_c0_seq4 comp57647_c2_seq1 comp51901_c0_seq2 comp57826_c6_seq15 comp37817_c0_seq1 comp46373_c0_seq1 comp55114_c0_seq1 comp55529_c0_seq10 comp58244_c0_seq9 comp49629_c0_seq2 comp56794_c0_seq3 comp28564_c1_seq1 comp48793_c0_seq1 comp55406_c0_seq1 comp55057_c0_seq1 comp54383_c1_seq2 comp56812_c0_seq8 comp31104_c0_seq1 comp51728_c0_seq1 comp53042_c0_seq2 comp56225_c1_seq3 comp58204_c0_seq3 comp48399_c0_seq5 comp46569_c0_seq1 comp57837_c1_seq11 comp57157_c0_seq2 comp30096_c0_seq1 comp44509_c0_seq4 comp57365_c0_seq9 comp55771_c0_seq5 comp45340_c0_seq1 comp47095_c0_seq1 comp48775_c0_seq3 comp53692_c0_seq3 comp46245_c0_seq1 comp56709_c1_seq3 comp39918_c1_seq1 comp51951_c3_seq1 comp41799_c0_seq1 comp54772_c3_seq7 comp41394_c0_seq1 comp31573_c0_seq1 comp42833_c2_seq1 comp56456_c0_seq1 comp57389_c0_seq3 comp57116_c4_seq3 comp53480_c1_seq2 comp57526_c0_seq1 comp49274_c0_seq2 comp56612_c3_seq3 comp52460_c2_seq8 comp56497_c0_seq1 comp49799_c0_seq2 comp31229_c2_seq1 comp54520_c1_seq4 comp53574_c0_seq3 comp54510_c0_seq2 comp56014_c0_seq2 comp57880_c2_seq7 comp60262_c0_seq1 comp49223_c0_seq1 comp56691_c1_seq3 comp50644_c1_seq1 comp57443_c11_seq3 comp54531_c0_seq2 comp90686_c0_seq1 comp46578_c0_seq3 comp56035_c0_seq2 comp52025_c1_seq1 comp49503_c0_seq1 comp48929_c3_seq1 comp54261_c1_seq3 comp53957_c1_seq1 comp56395_c3_seq3 comp49879_c2_seq1 comp57491_c0_seq1 comp47231_c0_seq1 comp52789_c0_seq1 comp53565_c1_seq2 comp55230_c1_seq2 comp46444_c0_seq2 comp48718_c0_seq1 comp33054_c0_seq1 comp50754_c0_seq3 comp55840_c1_seq1 comp41487_c0_seq1 comp57779_c0_seq9 comp53455_c0_seq4 comp57232_c1_seq6 comp53089_c0_seq2 comp52835_c0_seq1 comp55873_c0_seq7 comp58072_c1_seq23 comp57848_c0_seq18 comp47570_c0_seq1 comp44051_c1_seq1 comp32045_c0_seq1 comp52077_c4_seq8 comp57734_c0_seq17 comp53770_c0_seq3 comp53853_c1_seq1 comp55183_c0_seq4 comp52521_c0_seq9 comp51761_c0_seq2 comp53435_c5_seq2 comp54571_c1_seq1 comp45942_c0_seq1 comp55411_c1_seq10 comp56429_c0_seq6 comp33018_c1_seq1 comp57098_c4_seq3 comp55902_c1_seq1 comp53964_c3_seq1 comp55238_c6_seq12 comp56704_c0_seq1 comp55646_c0_seq5 comp57716_c2_seq4 comp56876_c6_seq4 comp34657_c0_seq1 comp31006_c0_seq1 comp51654_c0_seq1 comp55338_c0_seq8 comp49053_c0_seq1 comp65795_c0_seq1 comp57351_c3_seq13 comp57427_c1_seq3 comp54931_c0_seq4 comp45927_c0_seq1 comp58256_c1_seq30 comp56397_c0_seq33 comp52474_c1_seq5 comp57444_c0_seq7 comp57022_c7_seq1 comp55976_c1_seq7 comp52103_c0_seq6 comp55531_c2_seq6 comp52542_c0_seq1 comp58192_c0_seq14 comp42819_c0_seq1 comp57394_c1_seq9 comp56544_c0_seq1 comp57587_c0_seq1 comp54573_c2_seq4 comp56726_c2_seq18 comp51150_c0_seq10 comp55693_c0_seq1 comp51876_c4_seq1 comp43448_c0_seq1 comp56477_c1_seq3 comp51112_c0_seq1 comp55046_c0_seq2 comp58206_c6_seq4 comp34338_c0_seq1 comp47681_c0_seq1 comp55080_c4_seq6 comp49183_c0_seq1 comp45181_c1_seq1 comp56116_c1_seq1 comp47719_c1_seq2 comp57108_c3_seq1 comp56992_c2_seq3 comp44677_c0_seq1 comp56746_c9_seq15 comp45812_c0_seq1 comp57260_c0_seq4 comp52314_c0_seq2 comp56636_c2_seq2 comp56570_c0_seq4 comp48661_c0_seq3 comp45271_c0_seq1 comp49702_c3_seq1 comp56107_c0_seq1 comp47150_c0_seq1 comp57605_c2_seq10 comp55392_c0_seq4 comp53378_c0_seq3 comp44290_c1_seq1 comp57067_c0_seq1 comp48859_c0_seq1 comp55350_c0_seq2 comp50932_c3_seq3 comp54473_c0_seq1 comp55184_c16_seq1 comp57568_c3_seq3 comp48189_c0_seq1 comp54119_c1_seq2 comp46349_c0_seq1 comp56386_c0_seq13 comp53983_c1_seq6 comp84854_c0_seq1 comp57673_c4_seq28 comp48100_c2_seq2 comp191142_c0_seq1 comp28502_c0_seq1 comp54844_c0_seq1 comp49806_c0_seq1 comp57222_c3_seq1 comp53159_c0_seq1 comp36682_c0_seq1 comp57764_c2_seq1 comp58062_c2_seq3 comp56797_c2_seq3 comp56707_c0_seq3 comp52738_c0_seq3 comp58009_c4_seq1 comp57965_c0_seq11 comp54508_c0_seq3 comp56000_c2_seq9 comp55491_c3_seq10 comp57198_c4_seq8 comp57716_c2_seq14 comp58137_c2_seq18 comp51560_c0_seq1 comp56259_c3_seq6 comp53124_c0_seq2 comp54401_c8_seq12 comp36167_c1_seq1 comp50957_c1_seq2 comp51124_c1_seq1 comp49674_c0_seq6 comp55841_c0_seq2 comp50938_c0_seq4 comp51205_c2_seq1 comp51653_c0_seq2 comp46997_c0_seq1 comp57345_c2_seq4 comp52021_c0_seq2 comp59169_c0_seq1 comp31759_c0_seq1 comp56414_c0_seq6 comp55853_c0_seq3 comp58700_c0_seq1 comp55234_c0_seq3 comp58208_c0_seq6 comp55040_c0_seq1 comp58188_c3_seq33 comp54268_c3_seq1 comp45473_c0_seq8 comp53202_c4_seq1 comp50952_c0_seq1 comp53553_c0_seq1 comp59394_c0_seq1 comp55151_c0_seq7 comp51583_c0_seq4 comp57696_c3_seq22 comp212411_c0_seq1 comp50576_c1_seq3 comp30214_c0_seq1 comp52790_c0_seq1 comp51454_c1_seq1 comp53221_c0_seq7 comp57957_c0_seq1 comp56804_c1_seq1 comp280571_c0_seq1 comp54269_c0_seq2 comp57604_c0_seq9 comp58048_c4_seq4 comp45778_c0_seq2 comp57976_c0_seq2 comp56799_c0_seq12 comp48684_c0_seq1 comp56930_c0_seq1 comp50498_c0_seq1 comp43989_c0_seq2 comp57345_c2_seq8 comp43292_c0_seq1 comp58108_c1_seq10 comp56768_c2_seq9 comp58136_c0_seq6 comp51910_c0_seq1 comp56964_c3_seq3 comp28627_c0_seq1 comp55627_c0_seq5 comp52439_c0_seq2 comp29699_c0_seq1 comp49575_c1_seq1 comp36814_c1_seq1 comp50500_c0_seq1 comp58263_c1_seq12 comp55409_c0_seq8 comp53890_c1_seq5 comp48486_c0_seq1 comp54287_c2_seq2 comp49382_c0_seq1 comp57640_c1_seq9 comp49469_c0_seq1 comp55515_c0_seq4 comp51474_c0_seq2 comp57207_c0_seq12 comp56322_c7_seq3 comp55634_c3_seq4 comp29449_c0_seq1 comp57661_c2_seq1 comp49819_c1_seq4 comp57102_c2_seq4 comp57074_c0_seq4 comp57359_c6_seq4 comp53269_c1_seq2 comp54406_c6_seq2 comp57834_c2_seq14 comp48011_c0_seq1 comp57683_c0_seq2 comp56228_c0_seq1 comp57532_c1_seq3 comp55575_c2_seq10 comp57778_c1_seq3 comp57332_c0_seq7 comp56443_c0_seq1 comp57317_c3_seq25 comp48986_c0_seq1 comp46153_c1_seq1 comp55882_c5_seq12 comp57487_c5_seq23 comp30129_c0_seq1 comp54892_c0_seq4 comp55107_c0_seq1 comp53426_c4_seq6 comp49671_c1_seq1 comp50568_c0_seq4 comp56112_c1_seq2 comp52104_c0_seq1 comp51542_c0_seq1 comp54956_c0_seq1 comp57198_c2_seq2 comp53097_c1_seq3 comp57428_c0_seq2 comp51132_c0_seq1 comp56836_c0_seq8 comp56085_c0_seq3 comp57946_c3_seq1 comp57577_c6_seq7 comp57578_c1_seq14 comp370831_c0_seq1 comp57100_c0_seq2 comp44271_c0_seq2 comp56053_c0_seq2 comp55766_c0_seq1 comp45132_c0_seq2 comp54949_c1_seq4 comp35773_c0_seq1 comp56390_c3_seq3 comp54070_c1_seq4 comp49126_c0_seq4 comp48763_c1_seq4 comp53352_c0_seq1 comp42907_c0_seq2 comp51209_c0_seq2 comp57635_c1_seq6 comp53990_c1_seq1 comp58298_c0_seq2 comp56630_c1_seq17 comp57666_c1_seq14 comp56670_c0_seq3 comp57929_c1_seq14 comp28979_c0_seq1 comp57304_c0_seq2 comp39206_c1_seq1 comp52709_c0_seq1 comp49238_c3_seq1 comp55709_c1_seq1 comp53646_c0_seq1 comp57444_c0_seq18 comp44609_c1_seq1 comp53895_c0_seq2 comp58186_c0_seq2 comp56594_c1_seq22 comp52244_c0_seq2 comp46785_c1_seq1 comp57939_c1_seq1 comp51882_c0_seq4 comp50056_c0_seq2 comp54623_c1_seq4 comp56458_c1_seq1 comp57846_c0_seq9 comp56716_c2_seq1 comp57772_c2_seq4 comp56436_c0_seq5 comp56304_c2_seq1 comp57805_c1_seq10 comp48713_c0_seq3 comp56479_c0_seq1 comp55275_c1_seq2 comp55724_c0_seq1 comp54468_c1_seq7 comp39537_c0_seq1 comp48334_c1_seq4 comp57682_c2_seq3 comp50473_c1_seq4 comp56284_c0_seq2 comp46498_c2_seq1 comp55501_c1_seq1 comp58088_c4_seq6 comp62010_c0_seq1 comp34728_c0_seq1 comp57031_c1_seq15 comp55154_c1_seq1 comp47747_c1_seq1 comp58143_c0_seq17 comp57430_c1_seq33 comp55743_c2_seq2 comp56243_c2_seq14 comp51448_c1_seq5 comp57923_c0_seq6 comp39355_c0_seq1 comp53681_c3_seq2 comp54516_c4_seq1 comp58127_c0_seq2 comp49546_c1_seq3 comp48709_c0_seq2 comp45994_c0_seq3 comp45019_c0_seq2 comp57173_c0_seq6 comp44434_c0_seq3 comp31880_c0_seq1 comp43054_c0_seq2 comp53918_c3_seq2 comp53448_c1_seq1 comp37260_c0_seq1 comp28321_c0_seq1 comp53974_c0_seq3 comp47348_c0_seq1 comp29095_c0_seq1 comp48098_c1_seq1 comp57143_c0_seq1 comp47415_c0_seq2 comp56600_c0_seq6 comp32060_c0_seq1 comp54278_c0_seq2 comp54765_c0_seq1 comp57499_c6_seq7 comp41927_c0_seq1 comp48923_c0_seq3 comp53285_c2_seq7 comp47539_c2_seq1 comp43279_c0_seq1 comp55190_c0_seq4 comp54763_c4_seq1 comp29914_c0_seq1 comp58277_c0_seq55 comp56121_c0_seq2 comp30708_c0_seq2 comp57362_c1_seq1 comp53779_c2_seq9 comp53499_c2_seq1 comp57669_c0_seq26 comp58087_c4_seq6 comp58158_c2_seq16 comp55461_c1_seq1 comp55655_c3_seq1 comp54788_c0_seq1 comp55409_c0_seq1 comp51126_c0_seq2 comp51985_c0_seq1 comp57997_c4_seq25 comp58278_c0_seq1 comp50450_c0_seq5 comp58294_c2_seq6 comp54213_c0_seq2 comp54711_c1_seq14 comp43091_c0_seq1 comp52831_c0_seq1 comp55363_c1_seq6 comp48551_c2_seq1 comp31095_c0_seq1 comp46807_c0_seq3 comp58102_c11_seq2 comp28951_c0_seq1 comp52410_c0_seq1 comp52064_c0_seq1 comp57230_c1_seq5 comp56792_c0_seq3 comp57351_c3_seq5 comp56622_c3_seq2 comp56685_c0_seq5 comp53655_c0_seq2 comp72715_c0_seq1 comp53377_c0_seq3 comp55835_c0_seq2 comp57964_c0_seq10 comp56055_c1_seq1 comp51613_c0_seq1 comp58280_c0_seq3 comp39463_c0_seq1 comp58765_c0_seq1 comp41624_c0_seq1 comp57099_c0_seq8 comp57658_c6_seq4 comp53413_c0_seq1 comp52518_c0_seq6 comp57468_c0_seq15 comp55948_c2_seq3 comp51919_c3_seq2 comp46409_c1_seq1 comp56288_c1_seq10 comp57805_c1_seq4 comp51992_c0_seq1 comp53370_c0_seq6 comp54806_c0_seq4 comp55505_c3_seq11 comp55437_c0_seq3 comp53018_c1_seq2 comp56634_c5_seq2 comp49604_c0_seq1 comp157656_c0_seq1 comp56060_c1_seq7 comp56688_c2_seq8 comp54523_c0_seq2 comp49351_c0_seq1 comp33064_c0_seq3 comp52216_c1_seq1 comp58231_c1_seq4 comp36673_c0_seq1 comp56192_c4_seq7 comp49979_c1_seq8 comp58255_c1_seq8 comp54224_c3_seq3 comp54731_c1_seq3 comp43504_c0_seq1 comp61624_c0_seq1 comp58699_c0_seq1 comp52668_c4_seq3 comp49394_c0_seq2 comp41952_c0_seq1 comp52438_c0_seq1 comp55958_c3_seq11 comp52554_c0_seq2 comp58239_c1_seq2 comp44164_c0_seq1 comp39510_c1_seq1 comp56665_c2_seq6 comp54491_c0_seq2 comp332527_c0_seq1 comp53198_c0_seq1 comp51275_c0_seq2 comp28656_c0_seq1 comp54672_c1_seq1 comp45967_c1_seq1 comp57285_c0_seq6 comp54947_c2_seq2 comp42385_c1_seq1 comp36847_c0_seq1 comp29258_c0_seq1 comp45314_c1_seq1 comp57022_c7_seq5 comp55822_c1_seq6 comp53463_c1_seq5 comp49327_c0_seq1 comp53750_c1_seq9 comp46022_c0_seq1 comp52130_c1_seq1 comp54486_c1_seq2 comp45421_c0_seq2 comp51323_c0_seq1 comp51440_c1_seq7 comp56104_c3_seq4 comp53143_c0_seq1 comp49973_c0_seq8 comp57111_c1_seq4 comp56145_c6_seq2 comp46939_c0_seq1 comp49325_c0_seq1 comp56378_c5_seq1 comp56749_c0_seq2 comp57542_c1_seq1 comp53061_c0_seq1 comp43027_c0_seq1 comp49157_c0_seq4 comp55070_c1_seq2 comp54425_c0_seq1 comp52638_c1_seq1 comp57071_c1_seq19 comp57179_c0_seq2 comp52274_c1_seq1 comp57776_c3_seq14 comp58252_c3_seq4 comp53823_c1_seq2 comp57459_c0_seq2 comp47737_c1_seq1 comp54390_c1_seq1 comp53060_c0_seq1 comp51444_c0_seq3 comp41600_c1_seq1 comp37996_c0_seq1 comp49060_c0_seq1 comp56799_c0_seq4 comp56091_c0_seq5 comp46646_c2_seq1 comp53538_c0_seq2 comp30750_c0_seq1 comp57671_c2_seq1 comp56032_c11_seq6 comp57257_c4_seq4 comp53140_c0_seq1 comp49212_c0_seq1 comp54532_c0_seq1 comp48260_c0_seq1 comp50276_c0_seq1 comp57804_c2_seq3 comp58231_c1_seq14 comp47343_c0_seq1 comp56832_c0_seq4 comp42255_c3_seq1 comp55797_c0_seq2 comp48728_c4_seq1 comp47572_c0_seq1 comp48910_c1_seq1 comp54023_c0_seq3 comp57718_c1_seq4 comp48478_c0_seq1 comp43244_c0_seq1 comp57897_c0_seq2 comp58113_c5_seq3 comp48210_c0_seq1 comp56620_c3_seq4 comp53737_c0_seq1 comp50685_c0_seq1 comp56115_c2_seq1 comp55480_c0_seq2 comp56848_c0_seq42 comp45753_c0_seq1 comp53544_c1_seq5 comp56298_c4_seq2 comp57788_c1_seq8 comp31111_c0_seq1 comp56582_c1_seq1 comp58122_c0_seq14 comp57378_c5_seq1 comp49666_c1_seq2 comp47989_c0_seq1 comp56395_c5_seq1 comp48011_c0_seq2 comp57145_c3_seq20 comp43529_c0_seq1 comp50059_c0_seq1 comp47927_c3_seq2 comp53864_c0_seq5 comp51847_c2_seq1 comp55335_c0_seq1 comp56521_c4_seq7 comp58198_c4_seq13 comp55205_c1_seq1 comp53715_c0_seq6 comp57975_c2_seq26 comp52235_c0_seq1 comp32749_c0_seq1 comp35870_c0_seq1 comp52420_c1_seq2 comp52461_c0_seq6 comp56075_c2_seq20 comp48924_c0_seq3 comp58289_c2_seq39 comp48051_c0_seq2 comp58265_c2_seq15 comp57909_c0_seq8 comp53243_c0_seq3 comp57786_c1_seq1 comp32510_c0_seq1 comp49217_c0_seq1 comp54882_c0_seq1 comp50974_c1_seq5 comp55640_c5_seq22 comp53833_c0_seq4 comp48956_c0_seq2 comp49764_c0_seq1 comp57042_c0_seq1 comp56221_c0_seq4 comp44035_c0_seq1 comp58240_c15_seq2 comp57410_c1_seq6 comp58248_c0_seq8 comp54760_c0_seq1 comp58222_c2_seq13 comp207008_c0_seq1 comp34468_c0_seq1 comp58001_c3_seq8 comp52468_c0_seq1 comp56346_c0_seq16 comp56591_c0_seq3 comp50097_c3_seq4 comp58297_c0_seq25 comp33068_c0_seq1 comp38447_c0_seq1 comp49015_c0_seq2 comp57867_c1_seq12 comp57236_c1_seq8 comp51220_c0_seq2 comp46890_c0_seq1 comp56016_c0_seq1 comp51024_c1_seq2 comp58141_c1_seq10 comp50454_c0_seq2 comp55179_c0_seq2 comp56878_c9_seq6 comp57261_c0_seq5 comp55345_c0_seq5 comp56215_c2_seq8 comp57579_c1_seq2 comp47819_c3_seq1 comp52101_c1_seq1 comp53766_c0_seq2 comp57170_c1_seq7 comp56378_c3_seq6 comp52909_c2_seq1 comp54561_c0_seq1 comp31793_c0_seq1 comp55739_c5_seq1 comp31786_c0_seq1 comp57739_c8_seq1 comp54760_c0_seq11 comp50342_c0_seq1 comp52395_c1_seq1 comp52945_c0_seq1 comp53647_c0_seq1 comp55312_c0_seq22 comp56279_c1_seq1 comp55787_c1_seq1 comp57136_c1_seq8 comp55587_c0_seq4 comp47103_c1_seq2 comp43263_c0_seq1 comp55430_c1_seq2 comp55615_c0_seq1 comp57945_c0_seq5 comp55496_c3_seq2 comp48696_c0_seq1 comp30570_c0_seq1 comp57272_c2_seq9 comp55491_c3_seq14 comp49010_c0_seq2 comp53786_c3_seq6 comp53343_c1_seq3 comp50742_c0_seq1 comp42302_c0_seq1 comp55585_c2_seq2 comp53830_c0_seq1 comp58026_c0_seq2 comp53554_c3_seq1 comp54974_c0_seq2 comp55530_c1_seq4 comp39957_c1_seq1 comp52394_c0_seq1 comp48825_c2_seq5 comp53957_c0_seq4 comp174712_c0_seq1 comp53984_c1_seq10 comp56103_c0_seq1 comp56720_c1_seq11 comp58060_c0_seq1 comp57207_c0_seq6 comp54209_c0_seq4 comp55083_c0_seq9 comp58042_c1_seq10 comp58248_c0_seq5 comp52120_c0_seq3 comp52780_c3_seq4 comp36461_c0_seq2 comp43800_c0_seq1 comp57776_c3_seq2 comp49600_c1_seq1 comp52607_c0_seq1 comp57639_c7_seq1 comp57797_c1_seq1 comp56788_c0_seq26 comp54074_c0_seq1 comp54692_c0_seq3 comp57777_c0_seq6 comp55084_c0_seq10 comp45911_c0_seq2 comp57454_c2_seq1 comp57401_c7_seq2 comp56384_c0_seq8 comp51652_c0_seq3 comp46525_c0_seq1 comp57407_c1_seq5 comp54439_c0_seq3 comp56367_c2_seq1 comp53727_c0_seq1 comp56381_c0_seq3 comp54830_c2_seq13 comp28253_c0_seq1 comp48951_c2_seq1 comp46176_c0_seq2 comp51970_c1_seq5 comp29464_c0_seq1 comp55692_c1_seq1 comp46280_c0_seq3 comp56217_c0_seq2 comp55385_c0_seq5 comp56670_c0_seq12 comp51554_c2_seq1 comp46042_c0_seq1 comp57640_c1_seq10 comp58175_c1_seq5 comp48837_c0_seq2 comp52039_c0_seq2 comp57327_c2_seq2 comp55167_c0_seq1 comp53886_c0_seq3 comp48241_c0_seq1 comp47883_c0_seq1 comp52667_c0_seq2 comp18196_c0_seq1 comp50246_c0_seq3 comp56569_c0_seq1 comp48407_c0_seq1 comp56767_c0_seq17 comp57051_c6_seq6 comp56564_c0_seq9 comp55033_c1_seq7 comp53241_c0_seq3 comp58194_c0_seq17 comp55900_c8_seq28 comp54282_c0_seq1 comp57409_c2_seq1 comp52044_c0_seq1 comp57464_c0_seq1 comp55543_c0_seq2 comp41376_c0_seq1 comp55788_c1_seq1 comp50098_c0_seq1 comp57910_c0_seq2 comp57080_c0_seq5 comp46009_c0_seq4 comp45314_c0_seq1 comp45782_c2_seq1 comp53272_c0_seq2 comp53607_c0_seq8 comp54932_c1_seq2 comp57407_c1_seq9 comp51435_c0_seq1 comp56561_c0_seq2 comp56071_c0_seq4 comp57609_c0_seq3 comp55322_c0_seq3 comp55090_c0_seq2 comp58062_c2_seq2 comp56070_c0_seq3 comp52896_c0_seq1 comp58184_c0_seq4 comp50679_c0_seq1 comp43254_c0_seq1 comp49454_c0_seq1 comp54120_c2_seq2 comp53153_c0_seq2 comp49459_c0_seq2 comp53593_c0_seq1 comp57312_c3_seq8 comp53979_c0_seq3 comp48594_c0_seq1 comp56545_c0_seq3 comp49460_c0_seq2 comp60276_c0_seq1 comp56178_c1_seq2 comp56554_c3_seq1 comp52642_c0_seq2 comp52568_c9_seq1 comp54966_c1_seq1 comp51478_c0_seq1 comp55628_c0_seq1 comp56981_c0_seq3 comp57544_c0_seq10 comp53743_c0_seq1 comp55872_c0_seq4 comp50583_c0_seq2 comp54577_c0_seq2 comp50912_c0_seq1 comp51970_c0_seq1 comp55881_c1_seq7 comp52237_c1_seq3 comp49296_c0_seq1 comp50711_c0_seq8 comp45963_c0_seq2 comp50049_c0_seq1 comp56941_c0_seq5 comp56346_c0_seq9 comp53405_c1_seq2 comp56399_c2_seq6 comp53135_c2_seq4 comp53899_c0_seq4 comp34278_c1_seq1 comp57368_c7_seq11 comp51340_c1_seq4 comp56831_c0_seq3 comp46335_c0_seq1 comp29002_c0_seq1 comp57134_c1_seq6 comp58099_c0_seq4 comp53748_c0_seq5 comp43676_c0_seq1 comp58253_c1_seq7 comp52032_c0_seq1 comp46175_c0_seq1 comp44386_c0_seq1 comp51008_c1_seq1 comp28748_c0_seq1 comp45847_c0_seq1 comp56077_c0_seq5 comp50529_c0_seq2 comp56380_c0_seq10 comp50312_c0_seq1 comp45273_c0_seq1 comp55644_c0_seq1 comp50966_c0_seq2 comp51521_c0_seq2 comp58304_c0_seq2 comp56096_c4_seq11 comp48317_c1_seq1 comp55164_c0_seq1 comp56829_c1_seq1 comp42340_c0_seq1 comp55248_c1_seq6 comp55998_c4_seq1 comp51829_c0_seq2 comp55868_c0_seq9 comp50127_c1_seq1 comp49502_c0_seq1 comp47932_c0_seq4 comp57539_c0_seq1 comp56987_c10_seq2 comp57951_c0_seq5 comp57000_c7_seq1 comp51398_c0_seq1 comp57839_c7_seq5 comp51857_c1_seq1 comp54216_c0_seq1 comp58178_c0_seq8 comp57982_c1_seq10 comp49125_c0_seq1 comp53844_c0_seq2 comp52564_c0_seq11 comp51952_c0_seq2 comp51266_c0_seq1 comp44485_c1_seq1 comp53943_c0_seq5 comp53918_c1_seq1 comp56361_c2_seq7 comp51233_c5_seq2 comp57629_c1_seq2 comp56922_c1_seq6 comp57473_c0_seq26 comp57165_c1_seq30 comp54193_c11_seq12 comp45755_c0_seq2 comp55024_c0_seq5 comp57530_c0_seq1 comp42033_c0_seq1 comp50636_c1_seq2 comp58142_c1_seq2 comp52565_c1_seq1 comp31541_c0_seq1 comp50581_c2_seq1 comp57193_c1_seq9 comp56164_c0_seq1 comp54335_c0_seq3 comp56632_c2_seq10 comp55941_c1_seq1 comp54614_c0_seq4 comp57490_c0_seq1 comp56452_c3_seq1 comp47547_c0_seq1 comp49570_c0_seq2 comp56430_c1_seq7 comp28500_c0_seq1 comp55543_c0_seq10 comp52372_c4_seq1 comp57365_c0_seq12 comp57644_c0_seq1 comp51149_c0_seq1 comp31528_c0_seq1 comp56705_c1_seq4 comp49367_c0_seq4 comp53050_c0_seq1 comp44428_c0_seq1 comp53722_c1_seq2 comp52361_c0_seq4 comp43466_c0_seq1 comp29328_c0_seq1 comp53584_c0_seq1 comp56843_c0_seq3 comp56003_c0_seq2 comp48940_c0_seq4 comp58635_c0_seq1 comp56378_c4_seq9 comp50632_c0_seq3 comp48120_c0_seq1 comp51739_c1_seq1 comp49282_c0_seq1 comp56940_c1_seq7 comp48710_c0_seq1 comp52933_c1_seq3 comp51298_c1_seq1 comp55278_c2_seq4 comp43491_c0_seq1 comp55491_c3_seq5 comp30352_c0_seq1 comp53168_c0_seq3 comp56429_c0_seq20 comp57716_c2_seq2 comp54686_c0_seq3 comp55122_c1_seq1 comp55589_c3_seq2 comp52968_c0_seq4 comp53815_c1_seq1 comp57120_c0_seq6 comp41608_c0_seq1 comp49139_c0_seq3 comp33076_c0_seq1 comp51800_c0_seq2 comp55931_c0_seq3 comp54598_c2_seq1 comp46511_c0_seq1 comp55191_c0_seq7 comp57850_c3_seq8 comp46188_c1_seq1 comp49294_c1_seq1 comp52059_c0_seq1 comp50166_c0_seq1 comp48451_c0_seq1 comp55620_c0_seq8 comp57023_c1_seq20 comp46696_c0_seq1 comp45546_c1_seq1 comp49479_c0_seq2 comp46774_c4_seq2 comp54179_c0_seq1 comp52943_c1_seq1 comp42419_c0_seq1 comp57492_c3_seq6 comp58213_c0_seq3 comp47253_c0_seq4 comp42322_c0_seq2 comp56081_c0_seq5 comp54817_c15_seq1 comp54130_c0_seq2 comp46038_c0_seq2 comp29388_c0_seq1 comp49846_c0_seq1 comp42761_c0_seq2 comp58011_c1_seq1 comp42390_c0_seq1 comp54830_c7_seq1 comp43178_c0_seq1 comp38396_c0_seq1 comp56904_c0_seq12 comp54265_c1_seq3 comp57147_c2_seq1 comp52935_c0_seq6 comp55857_c10_seq3 comp52001_c0_seq1 comp57740_c6_seq12 comp44122_c1_seq1 comp50652_c1_seq5 comp48413_c1_seq4 comp58068_c4_seq1 comp71169_c0_seq1 comp47738_c0_seq1 comp49457_c0_seq2 comp49906_c1_seq4 comp52162_c1_seq2 comp50308_c2_seq1 comp49451_c0_seq1 comp57793_c0_seq1 comp52217_c0_seq1 comp58066_c1_seq5 comp52299_c0_seq2 comp48308_c0_seq1 comp35250_c1_seq1 comp46679_c0_seq2 comp53849_c0_seq3 comp34620_c0_seq2 comp52057_c0_seq1 comp50843_c0_seq1 comp56625_c1_seq13 comp56408_c2_seq2 comp57710_c1_seq6 comp44646_c0_seq1 comp48497_c0_seq2 comp57263_c7_seq3 comp52156_c0_seq2 comp50570_c3_seq1 comp53961_c0_seq1 comp55357_c0_seq2 comp56571_c0_seq21 comp34812_c0_seq1 comp55677_c3_seq2 comp57496_c1_seq1 comp29417_c0_seq1 comp55600_c3_seq3 comp49192_c1_seq4 comp58225_c0_seq2 comp47805_c0_seq5 comp56967_c0_seq2 comp31403_c0_seq1 comp56678_c0_seq8 comp57199_c0_seq1 comp46973_c0_seq3 comp50216_c0_seq3 comp55687_c1_seq3 comp43343_c0_seq1 comp46382_c0_seq3 comp50247_c0_seq1 comp50798_c0_seq4 comp85017_c0_seq1 comp58040_c3_seq6 comp55859_c6_seq3 comp53306_c0_seq3 comp40593_c0_seq1 comp55895_c0_seq11 comp57636_c0_seq9 comp56033_c0_seq1 comp55573_c2_seq1 comp48605_c0_seq5 comp31816_c0_seq1 comp54170_c0_seq2 comp56610_c2_seq7 comp55942_c0_seq1 comp57340_c0_seq5 comp51737_c0_seq1 comp47093_c3_seq1 comp46117_c0_seq1 comp58195_c0_seq4 comp56023_c0_seq1 comp57793_c0_seq14 comp55466_c1_seq7 comp54268_c6_seq4 comp56113_c0_seq4 comp57883_c0_seq10 comp49481_c1_seq1 comp55302_c0_seq10 comp56547_c0_seq8 comp53774_c0_seq1 comp42172_c1_seq1 comp54263_c0_seq1 comp49031_c0_seq2 comp44060_c0_seq1 comp51274_c0_seq5 comp53592_c0_seq1 comp57024_c0_seq7 comp41796_c0_seq1 comp54610_c1_seq1 comp57572_c0_seq1 comp54467_c0_seq1 comp42680_c0_seq1 comp56965_c1_seq1 comp47499_c0_seq1 comp55188_c3_seq8 comp54460_c2_seq1 comp50016_c2_seq1 comp57467_c0_seq12 comp56934_c0_seq4 comp48056_c0_seq2 comp54941_c0_seq4 comp54102_c0_seq2 comp57471_c2_seq7 comp43899_c0_seq1 comp57837_c1_seq14 comp57721_c4_seq1 comp56096_c4_seq18 comp51937_c1_seq1 comp55923_c0_seq11 comp49678_c1_seq4 comp55939_c2_seq1 comp54288_c1_seq1 comp57434_c4_seq7 comp33026_c0_seq1 comp63451_c0_seq1 comp50208_c1_seq1 comp42251_c0_seq1 comp55640_c5_seq28 comp53643_c0_seq1 comp48616_c0_seq2 comp38736_c0_seq1 comp44232_c0_seq1 comp54607_c1_seq4 comp57298_c4_seq3 comp56517_c0_seq2 comp49873_c2_seq3 comp65435_c0_seq1 comp28564_c0_seq1 comp43945_c0_seq1 comp47235_c2_seq1 comp57531_c0_seq2 comp52320_c0_seq7 comp57989_c0_seq5 comp52859_c0_seq2 comp56991_c1_seq4 comp55181_c0_seq9 comp48364_c0_seq1 comp52289_c0_seq1 comp31834_c0_seq1 comp55266_c3_seq2 comp48709_c0_seq4 comp49716_c3_seq2 comp58063_c0_seq17 comp57613_c1_seq2 comp54708_c1_seq8 comp28281_c0_seq1 comp55817_c3_seq2 comp52168_c0_seq1 comp49487_c0_seq1 comp57641_c1_seq5 comp51634_c0_seq1 comp55571_c1_seq1 comp56938_c4_seq1 comp58153_c0_seq26 comp55527_c0_seq5 comp58093_c1_seq2 comp52561_c1_seq1 comp54966_c0_seq3 comp57320_c1_seq10 comp52954_c0_seq2 comp48429_c0_seq4 comp56572_c0_seq8 comp57636_c0_seq11 comp55812_c1_seq1 comp41536_c0_seq1 comp55732_c0_seq1 comp56266_c0_seq5 comp58223_c6_seq49 comp55853_c0_seq1 comp56005_c0_seq2 comp52638_c0_seq1 comp41728_c0_seq1 comp46738_c0_seq1 comp55238_c10_seq8 comp55449_c0_seq3 comp46817_c1_seq2 comp47981_c0_seq1 comp55479_c1_seq1 comp37915_c0_seq2 comp42800_c0_seq1 comp55523_c6_seq4 comp46263_c0_seq1 comp55458_c0_seq2 comp56848_c0_seq29 comp57432_c0_seq10 comp54948_c0_seq1 comp54944_c14_seq1 comp47726_c0_seq3 comp57629_c3_seq9 comp58184_c0_seq10 comp50265_c1_seq4 comp56813_c3_seq5 comp53191_c0_seq1 comp52012_c5_seq8 comp55556_c0_seq8 comp43227_c0_seq1 comp53505_c0_seq1 comp53493_c0_seq8 comp48054_c5_seq1 comp55847_c0_seq5 comp48313_c3_seq2 comp50474_c0_seq2 comp55144_c0_seq3 comp57929_c4_seq1 comp46105_c0_seq2 comp54432_c0_seq1 comp56604_c2_seq1 comp48600_c0_seq2 comp34219_c1_seq1 comp50273_c1_seq2 comp57856_c8_seq1 comp47469_c0_seq1 comp51486_c0_seq1 comp57837_c1_seq5 comp52846_c0_seq1 comp54168_c0_seq2 comp57573_c1_seq3 comp46397_c0_seq2 comp56169_c0_seq1 comp50946_c2_seq1 comp57431_c4_seq2 comp53578_c0_seq1 comp56152_c0_seq4 comp42346_c0_seq1 comp175119_c0_seq1 comp52240_c1_seq1 comp56750_c1_seq1 comp55742_c0_seq6 comp51806_c0_seq1 comp52488_c0_seq8 comp58426_c0_seq1 comp47862_c0_seq1 comp57900_c0_seq6 comp48067_c0_seq1 comp57554_c1_seq3 comp58292_c0_seq26 comp29512_c0_seq1 comp51837_c1_seq2 comp48416_c0_seq1 comp57906_c1_seq5 comp58154_c2_seq23 comp57072_c0_seq3 comp57316_c1_seq11 comp48224_c0_seq2 comp45719_c2_seq1 comp58139_c1_seq5 comp55070_c1_seq12 comp51300_c0_seq5 comp56510_c0_seq1 comp54833_c0_seq1 comp32694_c1_seq1 comp55731_c1_seq3 comp38636_c0_seq1 comp51940_c2_seq2 comp30900_c0_seq1 comp55062_c0_seq4 comp57069_c2_seq2 comp53392_c0_seq3 comp57699_c1_seq1 comp46321_c0_seq3 comp52571_c0_seq3 comp54193_c14_seq1 comp57665_c1_seq22 comp53551_c1_seq4 comp57309_c0_seq7 comp57878_c0_seq8 comp48613_c0_seq1 comp57951_c0_seq9 comp50253_c0_seq2 comp52722_c1_seq1 comp56128_c1_seq5 comp52556_c0_seq1 comp38147_c0_seq1 comp55939_c0_seq1 comp51724_c0_seq1 comp49594_c2_seq1 comp55886_c6_seq1 comp58152_c1_seq4 comp48290_c0_seq4 comp50274_c0_seq3 comp48908_c1_seq6 comp43627_c0_seq1 comp50576_c2_seq1 comp53109_c0_seq1 comp28209_c0_seq1 comp29560_c0_seq1 comp57908_c0_seq6 comp58116_c1_seq2 comp57679_c1_seq4 comp51155_c0_seq1 comp51089_c0_seq1 comp55343_c1_seq6 comp51757_c0_seq1 comp51793_c0_seq1 comp57209_c0_seq3 comp55821_c0_seq2 comp49742_c0_seq3 comp46020_c0_seq1 comp29323_c0_seq1 comp52900_c0_seq2 comp57974_c0_seq3 comp56691_c0_seq2 comp56346_c0_seq15 comp57018_c1_seq1 comp57175_c0_seq2 comp55878_c0_seq3 comp51722_c1_seq1 comp57792_c0_seq5 comp57140_c5_seq2 comp57834_c2_seq11 comp53900_c0_seq1 comp56255_c7_seq3 comp57506_c0_seq2 comp55689_c1_seq3 comp53405_c1_seq1 comp54550_c0_seq2 comp55932_c0_seq8 comp55831_c2_seq1 comp53426_c4_seq2 comp51209_c0_seq1 comp53227_c1_seq2 comp51284_c0_seq1 comp54082_c0_seq2 comp42290_c0_seq2 comp56809_c2_seq9 comp42623_c0_seq1 comp42633_c0_seq1 comp53009_c0_seq2 comp55526_c4_seq1 comp47068_c0_seq3 comp47148_c0_seq1 comp56607_c0_seq5 comp53716_c0_seq2 comp48577_c2_seq1 comp50092_c2_seq1 comp51467_c0_seq4 comp55168_c4_seq2 comp46554_c0_seq1 comp50446_c0_seq1 comp30092_c0_seq1 comp41399_c0_seq1 comp52356_c0_seq1 comp56617_c3_seq1 comp57409_c1_seq8 comp46578_c0_seq2 comp52190_c0_seq4 comp53424_c2_seq3 comp42171_c0_seq1 comp45206_c0_seq1 comp52265_c0_seq1 comp43689_c0_seq1 comp57708_c1_seq1 comp48453_c0_seq1 comp54508_c0_seq4 comp58144_c0_seq2 comp45729_c0_seq2 comp50353_c2_seq1 comp49100_c0_seq1 comp46255_c0_seq3 comp58294_c2_seq11 comp51494_c0_seq1 comp57803_c0_seq8 comp56133_c0_seq1 comp49985_c1_seq1 comp57248_c0_seq7 comp52071_c2_seq1 comp58064_c0_seq13 comp55710_c0_seq2 comp52041_c2_seq2 comp48829_c0_seq1 comp50097_c3_seq9 comp57412_c3_seq1 comp52485_c0_seq1 comp52507_c0_seq1 comp52801_c0_seq1 comp55866_c1_seq1 comp56047_c2_seq1 comp51299_c1_seq1 comp53294_c1_seq4 comp53055_c1_seq1 comp55856_c2_seq2 comp56283_c1_seq4 comp54252_c0_seq1 comp54128_c0_seq1 comp31799_c0_seq1 comp53458_c0_seq1 comp42340_c0_seq2 comp50344_c2_seq1 comp37555_c0_seq1 comp57023_c1_seq10 comp56659_c6_seq4 comp27916_c0_seq1 comp56441_c1_seq2 comp46489_c0_seq1 comp56141_c2_seq12 comp52747_c1_seq2 comp56395_c5_seq5 comp56648_c1_seq7 comp52979_c2_seq1 comp50345_c0_seq1 comp56526_c0_seq1 comp49363_c0_seq1 comp56876_c6_seq1 comp52831_c2_seq1 comp43136_c0_seq2 comp53863_c1_seq11 comp56487_c4_seq8 comp55137_c2_seq29 comp54954_c1_seq1 comp55683_c0_seq1 comp50104_c0_seq1 comp43106_c0_seq1 comp47803_c2_seq1 comp56944_c0_seq2 comp57568_c3_seq5 comp57154_c0_seq1 comp56884_c1_seq5 comp54136_c0_seq3 comp51758_c0_seq9 comp47698_c3_seq1 comp49512_c0_seq2 comp57465_c0_seq3 comp58001_c3_seq17 comp54849_c3_seq6 comp46154_c0_seq1 comp57073_c4_seq1 comp42433_c0_seq1 comp41436_c0_seq1 comp54533_c2_seq1 comp32549_c0_seq1 comp56669_c0_seq1 comp32147_c0_seq1 comp57880_c2_seq2 comp52408_c0_seq2 comp57579_c1_seq3 comp56772_c0_seq1 comp53639_c1_seq5 comp48089_c1_seq2 comp29698_c0_seq1 comp54313_c0_seq1 comp28610_c0_seq1 comp53218_c0_seq3 comp57488_c3_seq1 comp49433_c0_seq1 comp50492_c1_seq1 comp55896_c1_seq5 comp57080_c0_seq7 comp54267_c1_seq1 comp55408_c0_seq1 comp57051_c10_seq1 comp49959_c0_seq1 comp52524_c0_seq6 comp52069_c1_seq3 comp56361_c2_seq14 comp51469_c0_seq2 comp56375_c0_seq3 comp55516_c1_seq12 comp57478_c0_seq6 comp52883_c1_seq1 comp56621_c2_seq2 comp56336_c0_seq13 comp51556_c1_seq1 comp57071_c1_seq21 comp49242_c0_seq1 comp54731_c1_seq5 comp53904_c1_seq1 comp57077_c0_seq5 comp55427_c0_seq1 comp28348_c0_seq1 comp46273_c1_seq2 comp50234_c0_seq1 comp54890_c1_seq1 comp44531_c0_seq2 comp54223_c1_seq1 comp47705_c0_seq3 comp54635_c0_seq3 comp49550_c0_seq1 comp55479_c12_seq12 comp49167_c2_seq1 comp49174_c1_seq1 comp31178_c1_seq1 comp57543_c0_seq5 comp56524_c2_seq2 comp54483_c0_seq3 comp58288_c0_seq5 comp56911_c3_seq3 comp58016_c5_seq4 comp52686_c0_seq6 comp57064_c0_seq1 comp43894_c0_seq1 comp58219_c6_seq16 comp57776_c3_seq5 comp53139_c0_seq1 comp53696_c0_seq2 comp56649_c0_seq2 comp55640_c7_seq1 comp28664_c0_seq1 comp42620_c0_seq1 comp58235_c2_seq1 comp39722_c0_seq1 comp47999_c0_seq2 comp44704_c0_seq3 comp55091_c0_seq4 comp53763_c1_seq1 comp51496_c0_seq1 comp29225_c0_seq1 comp56019_c1_seq1 comp57616_c0_seq2 comp40827_c0_seq1 comp49667_c0_seq1 comp46932_c0_seq1 comp57965_c0_seq1 comp56567_c1_seq6 comp53814_c0_seq1 comp52569_c0_seq1 comp49515_c0_seq3 comp49376_c1_seq1 comp54703_c2_seq7 comp54418_c4_seq5 comp52358_c0_seq1 comp46863_c0_seq2 comp57549_c6_seq3 comp48652_c0_seq1 comp57807_c12_seq1 comp56549_c3_seq1 comp53232_c1_seq1 comp48847_c0_seq1 comp54080_c0_seq1 comp58009_c2_seq9 comp59546_c0_seq1 comp57520_c0_seq9 comp56890_c1_seq1 comp55966_c0_seq1 comp142880_c0_seq1 comp58021_c3_seq7 comp53310_c0_seq6 comp57226_c1_seq2 comp55529_c0_seq11 comp48539_c0_seq1 comp55576_c2_seq1 comp47867_c0_seq1 comp57729_c2_seq17 comp29893_c0_seq1 comp53269_c0_seq1 comp42159_c0_seq2 comp58049_c0_seq11 comp48711_c1_seq3 comp42496_c0_seq1 comp48090_c0_seq1 comp51412_c1_seq1 comp52306_c0_seq6 comp43977_c1_seq4 comp55187_c2_seq1 comp42145_c0_seq1 comp58240_c15_seq1 comp55491_c3_seq8 comp52072_c1_seq1 comp57029_c0_seq1 comp47659_c0_seq1 comp53485_c0_seq1 comp55515_c0_seq1 comp51856_c0_seq1 comp55425_c0_seq1 comp57012_c0_seq8 comp52592_c2_seq1 comp48197_c0_seq1 comp51856_c1_seq7 comp58233_c8_seq4 comp46931_c0_seq2 comp48329_c0_seq2 comp44946_c0_seq1 comp57830_c4_seq9 comp53349_c1_seq5 comp55189_c2_seq3 comp49320_c0_seq2 comp55617_c0_seq18 comp56117_c1_seq16 comp50704_c1_seq2 comp38062_c0_seq1 comp49587_c0_seq1 comp54234_c0_seq4 comp57939_c1_seq2 comp57179_c0_seq3 comp56051_c0_seq1 comp56904_c0_seq5 comp56873_c0_seq11 comp58006_c5_seq2 comp58145_c0_seq54 comp57280_c1_seq11 comp53391_c1_seq1 comp81809_c0_seq1 comp51719_c0_seq1 comp57765_c2_seq9 comp93759_c0_seq1 comp58047_c0_seq19 comp45201_c0_seq1 comp53658_c1_seq2 comp32796_c0_seq1 comp55926_c2_seq3 comp74641_c0_seq1 comp46036_c1_seq3 comp55579_c2_seq1 comp55531_c0_seq5 comp55992_c0_seq2 comp56980_c1_seq1 comp30738_c0_seq1 comp56467_c1_seq1 comp55465_c1_seq4 comp46946_c0_seq1 comp39582_c0_seq1 comp57091_c9_seq1 comp55886_c9_seq4 comp50755_c1_seq1 comp56346_c0_seq12 comp52655_c0_seq1 comp55084_c0_seq2 comp56849_c4_seq4 comp57783_c0_seq7 comp57473_c0_seq8 comp31211_c0_seq1 comp53996_c1_seq1 comp29606_c1_seq1 comp57052_c1_seq5 comp42224_c0_seq1 comp35533_c0_seq1 comp55124_c0_seq2 comp55716_c0_seq1 comp46291_c0_seq7 comp52463_c0_seq1 comp50877_c1_seq3 comp49805_c4_seq1 comp46672_c0_seq1 comp57429_c6_seq4 comp57532_c0_seq2 comp57324_c1_seq24 comp56731_c1_seq3 comp57140_c5_seq5 comp51472_c0_seq1 comp35267_c0_seq2 comp57909_c0_seq5 comp58158_c2_seq1 comp49575_c0_seq1 comp57755_c2_seq3 comp47471_c0_seq2 comp58140_c7_seq2 comp56988_c0_seq2 comp57203_c3_seq1 comp53192_c1_seq2 comp48688_c0_seq2 comp54963_c3_seq1 comp57547_c2_seq1 comp57335_c0_seq9 comp53252_c0_seq4 comp56374_c0_seq5 comp57549_c12_seq1 comp50938_c0_seq5 comp55902_c1_seq4 comp58236_c0_seq37 comp33237_c0_seq1 comp49330_c1_seq1 comp53607_c0_seq14 comp57653_c0_seq3 comp51038_c0_seq1 comp37980_c2_seq1 comp43794_c0_seq1 comp57999_c1_seq6 comp47333_c2_seq1 comp55589_c3_seq6 comp57237_c7_seq5 comp31411_c0_seq1 comp46337_c0_seq1 comp57031_c1_seq3 comp56219_c0_seq6 comp53800_c1_seq2 comp49157_c0_seq3 comp55437_c0_seq4 comp41462_c0_seq1 comp44900_c0_seq2 comp57687_c1_seq12 comp58013_c2_seq3 comp51326_c0_seq1 comp57807_c7_seq6 comp59417_c0_seq1 comp58145_c0_seq9 comp27015_c0_seq1 comp49393_c0_seq3 comp28442_c0_seq1 comp51311_c0_seq5 comp53456_c0_seq3 comp56575_c0_seq3 comp33482_c0_seq1 comp40180_c0_seq1 comp41555_c0_seq2 comp54060_c0_seq2 comp52944_c0_seq1 comp58130_c0_seq14 comp49826_c0_seq1 comp29805_c0_seq1 comp57862_c2_seq5 comp49794_c0_seq2 comp57306_c0_seq6 comp54024_c1_seq1 comp58115_c0_seq4 comp55661_c0_seq2 comp48133_c0_seq1 comp57029_c0_seq4 comp55160_c4_seq2 comp57975_c2_seq18 comp55133_c0_seq15 comp48742_c0_seq2 comp41779_c0_seq2 comp55860_c0_seq2 comp53884_c1_seq1 comp57124_c4_seq3 comp56111_c0_seq1 comp54064_c1_seq4 comp52453_c2_seq6 comp53928_c0_seq2 comp55418_c0_seq3 comp48626_c2_seq1 comp57830_c4_seq2 comp54118_c0_seq1 comp53972_c0_seq2 comp30903_c0_seq1 comp33029_c0_seq1 comp56457_c0_seq1 comp57471_c2_seq3 comp47700_c0_seq1 comp44077_c0_seq1 comp55878_c2_seq2 comp55789_c0_seq2 comp51482_c2_seq3 comp56012_c1_seq2 comp53065_c1_seq1 comp48951_c1_seq1 comp52110_c1_seq2 comp48090_c1_seq1 comp49877_c0_seq1 comp55504_c0_seq6 comp56723_c2_seq2 comp53094_c0_seq1 comp55102_c1_seq2 comp54942_c1_seq2 comp50472_c0_seq3 comp32647_c0_seq2 comp58213_c0_seq6 comp49958_c0_seq1 comp48960_c1_seq2 comp124763_c0_seq1 comp30674_c0_seq1 comp57986_c0_seq7 comp48060_c1_seq1 comp57487_c5_seq20 comp33150_c0_seq1 comp46800_c0_seq4 comp48703_c0_seq1 comp56478_c0_seq1 comp46737_c1_seq2 comp53293_c0_seq1 comp58252_c3_seq10 comp27969_c0_seq1 comp53395_c0_seq2 comp48221_c1_seq2 comp53759_c0_seq7 comp29426_c0_seq1 comp52402_c0_seq2 comp52856_c0_seq1 comp28803_c0_seq1 comp49081_c0_seq2 comp55563_c0_seq1 comp51782_c1_seq1 comp58198_c4_seq10 comp53779_c4_seq4 comp57952_c5_seq7 comp55863_c0_seq6 comp53611_c0_seq1 comp56060_c0_seq7 comp50700_c2_seq1 comp57063_c2_seq5 comp56794_c0_seq2 comp57351_c3_seq9 comp60115_c0_seq1 comp32932_c0_seq1 comp56415_c0_seq1 comp42245_c2_seq1 comp55164_c0_seq5 comp81910_c0_seq1 comp48748_c0_seq1 comp56436_c0_seq9 comp56939_c0_seq1 comp57290_c1_seq9 comp50088_c2_seq2 comp58218_c0_seq10 comp30224_c1_seq1 comp49836_c0_seq1 comp57098_c3_seq4 comp43021_c2_seq1 comp56654_c3_seq10 comp56977_c1_seq1 comp56357_c5_seq2 comp42581_c0_seq1 comp56936_c0_seq2 comp56428_c4_seq7 comp57898_c3_seq9 comp57944_c1_seq4 comp56145_c8_seq4 comp55792_c0_seq5 comp55702_c1_seq1 comp49063_c0_seq1 comp53360_c2_seq1 comp52519_c0_seq1 comp56886_c0_seq5 comp57263_c2_seq2 comp30619_c0_seq1 comp54674_c1_seq1 comp43658_c1_seq1 comp57059_c2_seq8 comp45841_c0_seq1 comp58259_c1_seq3 comp51990_c2_seq1 comp46056_c0_seq1 comp57269_c0_seq3 comp47715_c0_seq1 comp49139_c0_seq4 comp46819_c0_seq1 comp56603_c2_seq2 comp51572_c1_seq2 comp55929_c3_seq1 comp54586_c3_seq9 comp56220_c0_seq1 comp53848_c0_seq3 comp50669_c0_seq4 comp41385_c0_seq2 comp50325_c0_seq2 comp55248_c1_seq12 comp55471_c1_seq1 comp32394_c0_seq1 comp58237_c5_seq9 comp54157_c7_seq13 comp49707_c0_seq1 comp57324_c1_seq6 comp44080_c1_seq1 comp30650_c0_seq1 comp56962_c3_seq4 comp47503_c0_seq1 comp53690_c4_seq11 comp52988_c0_seq2 comp51489_c0_seq1 comp56357_c4_seq16 comp58285_c1_seq16 comp36377_c0_seq1 comp52808_c4_seq2 comp55639_c0_seq10 comp57659_c5_seq10 comp57610_c6_seq1 comp51130_c1_seq5 comp56090_c1_seq4 comp36730_c0_seq1 comp54337_c0_seq15 comp54932_c1_seq6 comp46240_c0_seq1 comp56265_c0_seq11 comp48223_c0_seq1 comp56601_c0_seq4 comp28010_c0_seq1 comp45512_c0_seq1 comp56327_c2_seq2 comp35201_c0_seq1 comp50691_c1_seq1 comp55928_c7_seq3 comp52465_c3_seq3 comp54137_c1_seq1 comp52620_c0_seq1 comp49176_c1_seq1 comp42361_c0_seq1 comp42197_c0_seq1 comp53346_c0_seq2 comp31768_c0_seq1 comp50675_c1_seq1 comp46812_c0_seq1 comp96961_c0_seq1 comp52784_c0_seq4 comp58047_c0_seq1 comp53629_c0_seq3 comp57040_c3_seq7 comp53721_c1_seq11 comp55295_c0_seq4 comp43696_c0_seq3 comp54621_c0_seq1 comp51633_c0_seq1 comp52427_c0_seq3 comp51757_c1_seq1 comp57474_c0_seq13 comp51756_c0_seq2 comp57887_c1_seq1 comp55417_c2_seq1 comp58284_c6_seq9 comp52339_c1_seq1 comp57717_c0_seq1 comp53755_c0_seq1 comp55870_c0_seq5 comp51686_c3_seq1 comp46572_c0_seq1 comp48033_c4_seq1 comp57557_c6_seq2 comp53279_c1_seq1 comp47995_c0_seq2 comp49083_c0_seq3 comp52254_c0_seq1 comp30447_c0_seq1 comp55629_c1_seq2 comp56163_c0_seq1 comp50044_c1_seq1 comp55139_c1_seq1 comp57824_c1_seq10 comp40682_c0_seq1 comp55866_c1_seq5 comp51229_c1_seq1 comp56710_c2_seq5 comp55209_c0_seq4 comp52777_c1_seq2 comp53861_c0_seq2 comp39424_c0_seq1 comp53993_c0_seq5 comp51674_c0_seq1 comp58176_c1_seq18 comp53305_c0_seq2 comp52898_c2_seq1 comp57977_c0_seq6 comp57116_c4_seq36 comp58522_c0_seq1 comp57933_c0_seq11 comp50996_c1_seq5 comp56123_c1_seq3 comp46436_c0_seq1 comp57370_c3_seq3 comp57112_c0_seq5 comp56505_c0_seq1 comp56197_c4_seq6 comp58084_c1_seq6 comp55793_c1_seq1 comp46524_c0_seq1 comp56840_c0_seq6 comp50355_c0_seq2 comp52195_c1_seq1 comp58209_c0_seq8 comp53606_c0_seq3 comp53384_c0_seq2 comp48445_c0_seq1 comp57634_c5_seq8 comp50423_c1_seq4 comp57612_c0_seq19 comp57818_c1_seq2 comp50346_c0_seq3 comp45796_c0_seq1 comp57712_c4_seq3 comp49784_c3_seq1 comp53592_c0_seq2 comp47057_c0_seq1 comp32169_c0_seq1 comp54332_c1_seq2 comp47623_c1_seq1 comp56877_c0_seq11 comp57124_c2_seq1 comp56484_c0_seq1 comp56378_c4_seq4 comp58259_c1_seq11 comp52043_c0_seq1 comp58239_c2_seq12 comp53237_c0_seq2 comp58010_c0_seq19 comp36036_c0_seq1 comp56030_c0_seq5 comp48890_c0_seq3 comp51463_c0_seq2 comp30699_c0_seq1 comp57838_c0_seq8 comp50408_c5_seq1 comp57685_c0_seq2 comp57183_c2_seq2 comp51157_c1_seq2 comp52239_c0_seq1 comp58168_c3_seq13 comp49901_c0_seq1 comp56718_c3_seq1 comp54661_c2_seq2 comp57097_c3_seq4 comp54412_c0_seq1 comp52605_c0_seq2 comp58117_c1_seq5 comp57390_c1_seq11 comp42001_c1_seq1 comp91835_c0_seq1 comp58103_c2_seq9 comp55895_c0_seq1 comp37120_c0_seq2 comp57764_c1_seq18 comp57136_c1_seq10 comp56877_c0_seq6 comp57535_c0_seq3 comp57188_c1_seq7 comp49346_c1_seq2 comp57395_c2_seq13 comp56075_c2_seq1 comp49132_c0_seq2 comp50883_c0_seq1 comp56495_c0_seq3 comp54726_c1_seq2 comp57600_c0_seq3 comp51131_c0_seq4 comp56955_c1_seq1 comp58091_c0_seq2 comp58108_c1_seq12 comp52343_c0_seq1 comp55032_c0_seq5 comp54254_c0_seq1 comp51117_c1_seq2 comp50250_c0_seq1 comp54585_c0_seq3 comp30492_c0_seq1 comp55862_c0_seq2 comp51893_c1_seq2 comp51956_c0_seq2 comp57473_c0_seq9 comp50754_c0_seq7 comp57665_c5_seq1 comp55809_c1_seq11 comp56534_c1_seq9 comp56998_c3_seq2 comp31038_c0_seq1 comp57740_c6_seq5 comp57834_c2_seq9 comp54394_c6_seq2 comp53590_c0_seq1 comp44079_c0_seq1 comp48606_c0_seq2 comp55006_c0_seq1 comp54864_c5_seq3 comp53994_c1_seq1 comp56481_c2_seq18 comp57839_c0_seq3 comp48630_c0_seq2 comp31318_c0_seq1 comp55764_c2_seq3 comp55339_c0_seq1 comp46790_c1_seq2 comp55596_c0_seq1 comp55502_c0_seq1 comp57577_c6_seq11 comp57077_c0_seq11 comp41830_c0_seq1 comp57587_c2_seq4 comp57013_c1_seq7 comp53711_c0_seq1 comp56018_c1_seq1 comp54258_c0_seq1 comp33245_c0_seq1 comp54753_c0_seq1 comp57585_c0_seq5 comp53686_c0_seq2 comp53286_c2_seq3 comp57026_c0_seq7 comp57734_c0_seq15 comp48392_c0_seq1 comp28371_c0_seq1 comp46141_c0_seq1 comp49239_c0_seq2 comp52242_c1_seq2 comp56062_c2_seq4 comp48292_c0_seq3 comp54830_c2_seq10 comp52826_c0_seq3 comp47607_c0_seq1 comp57313_c0_seq3 comp29835_c0_seq1 comp51751_c0_seq2 comp51106_c1_seq6 comp56265_c0_seq6 comp142345_c0_seq1 comp48296_c0_seq2 comp47651_c1_seq1 comp57351_c4_seq1 comp41265_c1_seq1 comp57082_c0_seq8 comp45244_c4_seq3 comp56386_c0_seq2 comp49571_c1_seq2 comp44043_c0_seq1 comp47247_c0_seq5 comp56209_c0_seq1 comp55621_c1_seq2 comp47833_c0_seq2 comp57461_c3_seq6 comp52615_c0_seq6 comp57434_c2_seq1 comp52342_c0_seq2 comp57867_c1_seq1 comp51019_c0_seq1 comp53554_c1_seq2 comp32754_c0_seq1 comp49293_c0_seq4 comp54253_c0_seq1 comp56975_c0_seq3 comp43317_c0_seq1 comp57605_c2_seq20 comp46617_c0_seq1 comp57333_c0_seq2 comp50630_c0_seq1 comp57727_c0_seq3 comp50727_c0_seq1 comp47568_c0_seq2 comp52553_c2_seq9 comp53922_c0_seq3 comp57696_c0_seq5 comp50412_c0_seq2 comp57001_c3_seq13 comp51083_c0_seq3 comp51591_c3_seq2 comp47462_c0_seq2 comp58283_c4_seq12 comp56737_c5_seq10 comp30912_c0_seq1 comp46030_c6_seq3 comp55803_c2_seq1 comp57377_c1_seq1 comp51679_c4_seq1 comp51618_c0_seq2 comp55828_c0_seq3 comp55514_c1_seq3 comp32713_c0_seq2 comp57779_c0_seq10 comp58206_c6_seq11 comp58285_c1_seq21 comp31162_c0_seq1 comp55270_c0_seq2 comp57709_c0_seq3 comp50869_c0_seq1 comp56176_c3_seq1 comp43356_c0_seq1 comp49133_c0_seq1 comp46619_c0_seq1 comp56576_c1_seq9 comp57502_c6_seq4 comp56290_c1_seq5 comp55256_c4_seq8 comp56948_c0_seq5 comp57900_c0_seq5 comp58166_c1_seq16 comp31475_c1_seq1 comp57636_c0_seq12 comp49581_c0_seq1 comp31197_c1_seq1 comp54006_c0_seq6 comp57233_c0_seq2 comp55288_c1_seq13 comp48482_c0_seq1 comp53241_c0_seq1 comp47044_c2_seq1 comp50870_c3_seq4 comp57441_c1_seq3 comp57095_c1_seq2 comp49845_c0_seq1 comp57566_c1_seq2 comp42135_c1_seq1 comp54138_c1_seq1 comp55698_c0_seq3 comp29431_c0_seq1 comp55250_c1_seq2 comp54682_c0_seq3 comp51978_c0_seq2 comp48381_c0_seq1 comp53983_c1_seq4 comp56628_c1_seq5 comp58093_c5_seq2 comp49909_c0_seq2 comp51356_c0_seq3 comp57681_c0_seq6 comp55302_c0_seq5 comp36299_c0_seq1 comp53081_c0_seq2 comp43674_c1_seq1 comp53559_c1_seq1 comp49694_c0_seq1 comp42285_c1_seq1 comp31683_c0_seq2 comp50418_c1_seq1 comp45052_c0_seq3 comp56531_c0_seq10 comp55210_c0_seq4 comp56447_c0_seq1 comp28978_c1_seq1 comp51476_c0_seq5 comp46375_c0_seq2 comp58124_c1_seq2 comp52486_c0_seq3 comp58130_c0_seq18 comp55384_c0_seq1 comp57193_c0_seq1 comp58092_c2_seq27 comp53766_c0_seq3 comp56768_c1_seq1 comp58156_c0_seq6 comp57725_c0_seq15 comp58059_c0_seq5 comp50911_c0_seq1 comp50777_c1_seq1 comp57446_c0_seq8 comp57551_c1_seq3 comp43227_c0_seq2 comp32632_c0_seq2 comp58144_c3_seq3 comp32158_c0_seq1 comp46050_c0_seq1 comp51453_c0_seq3 comp54850_c0_seq2 comp56842_c0_seq1 comp39065_c0_seq1 comp46941_c1_seq1 comp53212_c1_seq2 comp56278_c0_seq5 comp51736_c0_seq5 comp53504_c0_seq2 comp48250_c0_seq1 comp47732_c1_seq1 comp56449_c0_seq1 comp43697_c0_seq1 comp57988_c4_seq6 comp47577_c0_seq2 comp52394_c0_seq2 comp56859_c1_seq1 comp58061_c0_seq24 comp57102_c2_seq11 comp56622_c1_seq18 comp55882_c5_seq1 comp57664_c3_seq5 comp57285_c0_seq13 comp55052_c2_seq1 comp51802_c1_seq2 comp58081_c1_seq2 comp32682_c0_seq1 comp55606_c2_seq2 comp55985_c2_seq1 comp52040_c0_seq1 comp58150_c0_seq11 comp55383_c2_seq3 comp42060_c1_seq2 comp56825_c1_seq2 comp57039_c1_seq1 comp48711_c1_seq1 comp56244_c1_seq5 comp46340_c0_seq2 comp52836_c0_seq1 comp30789_c0_seq5 comp54283_c0_seq1 comp51094_c0_seq1 comp41575_c0_seq1 comp53580_c0_seq1 comp28230_c0_seq1 comp54434_c0_seq1 comp9818_c0_seq1 comp45688_c0_seq2 comp32790_c0_seq1 comp56817_c3_seq1 comp57468_c0_seq3 comp47080_c1_seq2 comp55121_c4_seq4 comp46726_c0_seq1 comp29879_c0_seq2 comp57962_c1_seq3 comp43415_c0_seq2 comp54889_c4_seq1 comp29520_c0_seq1 comp54946_c0_seq4 comp47023_c0_seq1 comp43127_c1_seq1 comp55381_c0_seq2 comp51823_c0_seq1 comp29597_c0_seq2 comp30725_c0_seq1 comp49244_c0_seq3 comp55356_c0_seq7 comp57426_c1_seq1 comp56595_c0_seq13 comp49827_c1_seq1 comp55900_c2_seq1 comp210507_c0_seq1 comp46882_c1_seq2 comp50808_c3_seq1 comp56929_c4_seq6 comp53369_c0_seq2 comp7711_c0_seq1 comp56106_c4_seq10 comp57784_c3_seq2 comp55118_c0_seq2 comp57822_c2_seq17 comp31150_c1_seq1 comp58050_c1_seq11 comp42399_c0_seq1 comp56121_c0_seq6 comp55073_c1_seq2 comp27954_c0_seq1 comp54598_c4_seq1 comp57496_c1_seq2 comp48692_c2_seq1 comp58103_c1_seq1 comp54944_c14_seq2 comp49488_c0_seq2 comp53955_c1_seq4 comp55495_c4_seq8 comp56235_c3_seq6 comp58169_c0_seq6 comp107190_c0_seq1 comp55428_c0_seq1 comp49129_c1_seq1 comp55949_c0_seq3 comp40740_c1_seq1 comp48851_c0_seq1 comp57617_c6_seq1 comp52498_c0_seq2 comp55556_c0_seq2 comp37687_c0_seq1 comp55516_c1_seq7 comp48689_c0_seq2 comp54619_c1_seq12 comp47273_c1_seq1 comp55398_c2_seq2 comp54927_c0_seq1 comp57360_c0_seq6 comp55816_c0_seq5 comp53443_c0_seq1 comp54612_c3_seq2 comp45852_c0_seq1 comp28651_c0_seq1 comp49075_c0_seq2 comp50249_c0_seq8 comp46163_c0_seq1 comp50474_c0_seq5 comp57458_c2_seq7 comp32174_c0_seq1 comp42240_c0_seq1 comp57407_c1_seq6 comp56785_c5_seq1 comp56113_c0_seq3 comp55924_c0_seq4 comp56341_c3_seq13 comp51143_c1_seq2 comp27887_c0_seq1 comp55378_c0_seq10 comp53964_c1_seq2 comp55900_c8_seq40 comp57058_c0_seq13 comp58260_c5_seq38 comp54214_c0_seq4 comp58045_c0_seq7 comp58106_c0_seq2 comp51036_c1_seq1 comp39161_c1_seq1 comp57681_c1_seq22 comp55014_c0_seq2 comp54117_c0_seq2 comp56741_c0_seq1 comp43174_c0_seq1 comp50283_c0_seq1 comp28637_c0_seq1 comp57122_c0_seq3 comp51010_c0_seq2 comp63732_c0_seq1 comp56563_c3_seq6 comp57340_c0_seq6 comp44060_c1_seq1 comp48124_c0_seq1 comp58202_c2_seq15 comp53842_c1_seq2 comp46215_c0_seq1 comp48211_c0_seq1 comp57904_c0_seq1 comp53942_c0_seq1 comp55075_c0_seq2 comp53175_c2_seq1 comp42721_c0_seq1 comp57153_c1_seq14 comp56049_c0_seq3 comp43374_c0_seq1 comp56252_c0_seq6 comp55617_c0_seq1 comp48541_c0_seq2 comp54144_c0_seq4 comp57564_c2_seq60 comp31227_c0_seq1 comp53527_c2_seq4 comp57068_c0_seq3 comp52391_c0_seq4 comp47211_c1_seq2 comp58102_c11_seq7 comp55762_c2_seq1 comp50748_c0_seq3 comp52585_c0_seq4 comp56616_c0_seq1 comp45513_c0_seq1 comp46917_c1_seq2 comp47075_c0_seq1 comp57274_c3_seq1 comp57742_c0_seq2 comp50762_c1_seq1 comp28833_c0_seq1 comp54942_c1_seq7 comp53293_c0_seq4 comp55353_c1_seq9 comp47790_c2_seq1 comp53373_c0_seq3 comp47589_c0_seq2 comp46216_c2_seq1 comp57445_c0_seq1 comp47881_c1_seq1 comp50255_c0_seq1 comp52488_c0_seq9 comp51773_c0_seq1 comp43347_c0_seq2 comp57002_c2_seq1 comp55948_c2_seq4 comp56019_c1_seq4 comp56594_c6_seq1 comp51998_c2_seq1 comp50758_c5_seq1 comp29478_c0_seq1 comp56920_c11_seq1 comp49089_c4_seq2 comp55213_c0_seq2 comp54841_c8_seq10 comp82770_c0_seq1 comp58036_c1_seq6 comp52831_c0_seq7 comp280790_c0_seq1 comp32476_c0_seq2 comp52793_c3_seq3 comp46010_c0_seq1 comp57803_c4_seq1 comp52595_c0_seq1 comp50378_c1_seq8 comp56906_c0_seq4 comp61013_c0_seq1 comp55153_c1_seq1 comp54241_c0_seq2 comp57739_c8_seq4 comp55934_c3_seq2 comp54353_c0_seq1 comp53169_c0_seq2 comp54387_c1_seq12 comp57139_c1_seq4 comp51919_c0_seq2 comp50301_c0_seq4 comp51198_c0_seq3 comp48390_c2_seq3 comp58009_c2_seq19 comp58202_c2_seq1 comp54746_c0_seq3 comp55034_c1_seq6 comp53631_c0_seq4 comp57632_c1_seq4 comp48020_c1_seq1 comp56960_c0_seq5 comp55918_c0_seq2 comp55813_c0_seq6 comp52417_c0_seq3 comp52872_c9_seq1 comp45831_c0_seq1 comp29481_c0_seq1 comp48212_c0_seq1 comp48752_c1_seq2 comp55603_c3_seq6 comp52479_c0_seq2 comp56814_c1_seq5 comp48749_c0_seq3 comp80988_c0_seq1 comp54021_c1_seq3 comp49294_c1_seq4 comp49650_c0_seq1 comp58307_c0_seq6 comp57706_c2_seq1 comp50277_c1_seq1 comp47780_c0_seq1 comp55939_c2_seq2 comp54431_c0_seq1 comp52148_c0_seq4 comp56670_c0_seq15 comp58167_c1_seq4 comp53335_c0_seq6 comp50656_c0_seq1 comp56354_c1_seq4 comp58137_c2_seq8 comp50437_c0_seq1 comp48506_c1_seq1 comp54811_c1_seq3 comp52579_c0_seq2 comp44518_c0_seq3 comp57972_c0_seq5 comp57620_c3_seq3 comp30342_c0_seq1 comp54955_c0_seq3 comp54227_c0_seq8 comp56004_c1_seq5 comp57713_c0_seq8 comp52849_c1_seq1 comp54560_c3_seq15 comp29246_c0_seq1 comp53429_c0_seq3 comp47973_c0_seq1 comp52892_c0_seq2 comp45120_c0_seq1 comp57564_c2_seq14 comp46076_c1_seq3 comp51183_c1_seq3 comp58274_c5_seq1 comp54030_c0_seq1 comp57210_c2_seq10 comp58089_c4_seq2 comp56705_c1_seq1 comp56321_c0_seq11 comp56346_c0_seq11 comp52524_c0_seq11 comp44752_c0_seq1 comp52814_c0_seq1 comp48709_c0_seq1 comp57051_c12_seq6 comp57583_c0_seq4 comp59866_c0_seq1 comp46405_c1_seq3 comp36481_c0_seq1 comp28404_c0_seq1 comp39236_c0_seq1 comp29192_c0_seq1 comp50951_c0_seq1 comp56331_c5_seq3 comp51212_c1_seq8 comp46567_c0_seq3 comp51371_c0_seq4 comp51894_c0_seq2 comp60392_c0_seq1 comp54422_c1_seq6 comp56600_c0_seq5 comp50631_c1_seq1 comp47233_c0_seq1 comp50254_c1_seq2 comp33194_c0_seq1 comp57741_c0_seq7 comp27784_c0_seq1 comp44259_c0_seq3 comp56680_c1_seq2 comp28153_c0_seq1 comp56761_c0_seq1 comp44134_c0_seq1 comp57086_c1_seq10 comp57393_c1_seq4 comp54846_c0_seq4 comp51200_c0_seq3 comp58048_c4_seq7 comp49192_c1_seq1 comp53125_c0_seq3 comp54686_c0_seq6 comp45096_c0_seq1 comp56327_c2_seq5 comp55520_c8_seq3 comp41995_c0_seq1 comp53903_c5_seq1 comp53238_c0_seq2 comp57424_c0_seq1 comp51785_c0_seq1 comp53471_c0_seq1 comp57907_c12_seq6 comp52821_c1_seq2 comp50466_c0_seq2 comp53250_c1_seq2 comp39944_c0_seq1 comp54785_c1_seq26 comp57137_c1_seq1 comp57380_c1_seq6 comp58151_c0_seq6 comp54268_c4_seq3 comp57633_c0_seq2 comp56564_c1_seq1 comp52068_c0_seq3 comp31216_c0_seq2 comp49082_c0_seq1 comp55606_c4_seq2 comp48183_c0_seq2 comp55925_c1_seq4 comp58197_c7_seq15 comp53812_c0_seq2 comp50400_c1_seq1 comp57547_c2_seq4 comp50388_c0_seq1 comp50010_c0_seq1 comp51475_c0_seq4 comp52869_c0_seq1 comp55134_c0_seq4 comp48121_c0_seq1 comp49788_c0_seq1 comp50171_c0_seq3 comp53918_c3_seq3 comp34387_c0_seq1 comp54732_c0_seq2 comp39032_c1_seq1 comp55496_c0_seq1 comp54877_c0_seq10 comp57594_c4_seq1 comp54610_c0_seq1 comp32097_c0_seq1 comp56234_c0_seq5 comp44513_c0_seq1 comp58084_c1_seq1 comp55763_c0_seq1 comp47137_c2_seq1 comp44116_c0_seq2 comp53940_c0_seq2 comp52366_c2_seq3 comp50380_c0_seq1 comp31200_c0_seq1 comp45083_c0_seq1 comp53224_c2_seq2 comp56271_c0_seq1 comp55210_c0_seq1 comp50871_c0_seq1 comp56483_c0_seq1 comp41973_c3_seq1 comp38878_c0_seq1 comp55555_c1_seq4 comp52494_c0_seq5 comp53631_c0_seq8 comp49534_c0_seq4 comp54780_c2_seq1 comp57314_c0_seq1 comp41375_c0_seq1 comp51776_c0_seq1 comp55061_c0_seq3 comp48308_c0_seq2 comp54115_c0_seq2 comp55162_c0_seq2 comp57950_c3_seq2 comp46289_c0_seq1 comp52831_c0_seq2 comp53242_c0_seq1 comp43601_c0_seq1 comp55623_c2_seq14 comp56695_c0_seq1 comp47605_c0_seq1 comp54087_c0_seq2 comp46077_c0_seq1 comp57091_c7_seq2 comp46092_c0_seq2 comp56145_c7_seq1 comp56622_c3_seq3 comp50297_c0_seq2 comp53809_c0_seq2 comp29536_c0_seq1 comp55149_c0_seq2 comp53808_c0_seq1 comp42088_c4_seq1 comp53322_c0_seq1 comp29603_c0_seq1 comp57230_c1_seq4 comp48879_c0_seq1 comp56869_c0_seq2 comp56284_c0_seq8 comp57764_c1_seq7 comp52448_c0_seq1 comp51044_c1_seq1 comp57505_c0_seq1 comp57407_c1_seq20 comp57653_c0_seq5 comp56323_c0_seq4 comp51248_c0_seq1 comp56117_c1_seq11 comp49770_c9_seq1 comp31301_c0_seq1 comp50209_c0_seq3 comp57680_c2_seq1 comp56574_c0_seq7 comp52274_c1_seq2 comp50630_c0_seq4 comp44270_c0_seq1 comp55168_c3_seq2 comp55181_c0_seq3 comp58194_c0_seq4 comp36157_c0_seq1 comp52702_c0_seq2 comp56798_c1_seq3 comp57257_c2_seq9 comp57930_c0_seq4 comp48826_c1_seq1 comp32606_c0_seq1 comp55670_c0_seq6 comp58109_c1_seq6 comp51276_c0_seq2 comp51247_c0_seq4 comp41146_c0_seq1 comp55742_c0_seq14 comp56106_c4_seq2 comp50860_c0_seq1 comp57431_c4_seq8 comp51897_c0_seq1 comp56326_c7_seq3 comp53436_c0_seq3 comp57551_c1_seq1 comp33046_c0_seq1 comp42601_c0_seq1 comp55492_c4_seq3 comp55063_c6_seq3 comp56024_c1_seq2 comp49596_c2_seq1 comp57632_c1_seq3 comp56610_c3_seq7 comp33239_c1_seq1 comp47910_c0_seq2 comp51363_c0_seq1 comp50884_c0_seq2 comp56385_c0_seq11 comp57377_c2_seq2 comp58253_c1_seq15 comp57634_c3_seq10 comp54843_c2_seq2 comp53382_c0_seq1 comp56395_c12_seq24 comp54296_c2_seq4 comp57988_c2_seq1 comp57587_c2_seq2 comp48843_c0_seq1 comp58145_c0_seq1 comp45914_c0_seq7 comp54969_c0_seq3 comp46368_c0_seq2 comp54884_c1_seq1 comp46089_c0_seq2 comp48878_c0_seq1 comp43990_c0_seq1 comp48991_c0_seq1 comp54619_c1_seq13 comp57105_c8_seq4 comp58269_c0_seq1 comp48406_c0_seq1 comp57749_c0_seq4 comp57581_c0_seq1 comp58011_c1_seq15 comp46106_c0_seq2 comp57379_c0_seq11 comp55184_c7_seq1 comp57377_c2_seq3 comp57997_c4_seq2 comp57363_c1_seq26 comp56629_c1_seq1 comp58259_c1_seq20 comp49770_c4_seq1 comp32476_c0_seq1 comp58153_c0_seq4 comp56138_c0_seq2 comp58081_c1_seq9 comp58300_c0_seq9 comp49436_c4_seq1 comp31792_c0_seq3 comp58102_c6_seq2 comp56993_c1_seq4 comp54864_c5_seq1 comp55550_c0_seq3 comp55424_c0_seq1 comp57260_c0_seq13 comp49318_c0_seq1 comp57550_c0_seq3 comp56831_c0_seq1 comp48270_c3_seq1 comp55551_c0_seq1 comp58255_c1_seq15 comp51117_c1_seq1 comp43026_c0_seq1 comp55689_c1_seq13 comp43808_c2_seq1 comp51827_c0_seq2 comp50601_c0_seq1 comp48918_c1_seq1 comp49386_c0_seq1 comp49595_c0_seq1 comp50793_c3_seq1 comp55744_c8_seq7 comp53657_c0_seq1 comp46070_c0_seq3 comp54748_c0_seq2 comp57122_c3_seq1 comp52383_c0_seq3 comp51025_c1_seq4 comp51338_c0_seq5 comp55522_c0_seq2 comp54268_c9_seq1 comp53133_c1_seq8 comp47357_c0_seq3 comp57721_c0_seq2 comp58269_c0_seq21 comp46129_c0_seq1 comp43553_c1_seq1 comp53397_c0_seq3 comp53565_c2_seq1 comp48118_c0_seq1 comp57013_c1_seq6 comp55195_c4_seq2 comp57612_c0_seq12 comp58062_c6_seq1 comp50208_c3_seq2 comp55412_c0_seq5 comp58113_c2_seq8 comp53945_c0_seq4 comp56304_c4_seq4 comp54828_c0_seq1 comp50825_c0_seq2 comp55363_c1_seq2 comp58151_c3_seq2 comp29245_c0_seq1 comp51518_c1_seq1 comp57850_c3_seq1 comp46465_c2_seq1 comp55462_c0_seq1 comp47040_c0_seq1 comp53400_c0_seq1 comp52130_c1_seq2 comp56654_c3_seq8 comp52293_c7_seq1 comp54711_c1_seq15 comp51320_c0_seq1 comp53614_c0_seq4 comp58285_c1_seq7 comp55168_c6_seq1 comp52698_c0_seq4 comp48036_c0_seq2 comp54086_c0_seq16 comp51378_c3_seq1 comp54650_c1_seq2 comp50837_c0_seq3 comp51512_c0_seq2 comp4674_c0_seq1 comp57617_c5_seq1 comp56191_c0_seq2 comp55715_c0_seq1 comp54586_c2_seq3 comp58190_c2_seq5 comp55236_c7_seq11 comp29566_c0_seq1 comp54084_c4_seq12 comp43553_c0_seq1 comp46382_c0_seq1 comp54613_c2_seq2 comp45244_c4_seq1 comp53770_c0_seq4 comp57475_c1_seq5 comp55693_c0_seq2 comp54758_c1_seq2 comp54832_c0_seq5 comp27708_c0_seq1 comp56432_c0_seq1 comp47581_c3_seq1 comp57280_c1_seq8 comp49979_c1_seq5 comp51715_c1_seq1 comp57685_c0_seq3 comp57716_c2_seq11 comp55017_c1_seq2 comp57446_c0_seq9 comp52826_c0_seq4 comp54644_c0_seq1 comp56864_c0_seq3 comp44769_c0_seq1 comp33580_c0_seq1 comp54747_c0_seq1 comp53343_c1_seq1 comp52897_c1_seq5 comp50933_c0_seq1 comp58268_c2_seq14 comp56437_c0_seq3 comp54093_c5_seq1 comp56879_c0_seq10 comp56218_c3_seq3 comp57301_c1_seq2 comp57490_c2_seq2 comp57770_c1_seq3 comp54608_c1_seq9 comp43136_c0_seq1 comp29195_c0_seq1 comp53401_c1_seq11 comp57272_c2_seq1 comp53686_c0_seq1 comp57971_c4_seq2 comp51110_c1_seq1 comp52486_c0_seq4 comp58093_c5_seq7 comp55730_c0_seq1 comp44869_c0_seq2 comp46682_c1_seq1 comp56876_c4_seq1 comp57327_c2_seq4 comp56361_c2_seq10 comp58041_c0_seq10 comp56192_c4_seq18 comp48228_c0_seq2 comp57000_c7_seq2 comp56852_c2_seq2 comp48538_c0_seq1 comp54851_c0_seq2 comp41257_c0_seq1 comp52803_c0_seq1 comp49530_c0_seq1 comp58118_c1_seq1 comp53878_c3_seq2 comp49082_c4_seq1 comp44072_c0_seq2 comp54062_c0_seq1 comp52347_c2_seq5 comp32038_c0_seq1 comp57634_c5_seq13 comp56858_c3_seq7 comp56326_c2_seq1 comp55552_c0_seq5 comp42062_c1_seq1 comp55721_c0_seq1 comp53430_c0_seq4 comp50200_c0_seq4 comp50443_c1_seq1 comp56746_c9_seq16 comp52548_c0_seq1 comp58016_c5_seq10 comp43687_c0_seq1 comp48770_c0_seq3 comp53924_c2_seq1 comp53239_c0_seq1 comp35594_c1_seq1 comp54076_c0_seq1 comp46688_c0_seq3 comp51657_c0_seq4 comp56753_c2_seq2 comp58239_c2_seq9 comp54056_c0_seq1 comp48801_c0_seq4 comp56987_c2_seq1 comp46870_c0_seq2 comp55533_c0_seq8 comp53156_c0_seq1 comp58042_c1_seq11 comp55485_c0_seq1 comp43463_c0_seq1 comp48050_c0_seq2 comp44879_c0_seq3 comp52738_c0_seq2 comp57654_c0_seq4 comp46205_c0_seq2 comp57387_c0_seq5 comp42577_c0_seq2 comp52440_c0_seq4 comp55543_c0_seq1 comp46116_c0_seq1 comp55350_c0_seq1 comp56653_c1_seq12 comp57153_c1_seq3 comp49816_c0_seq1 comp56151_c0_seq18 comp48269_c0_seq1 comp46728_c7_seq1 comp55520_c0_seq1 comp56810_c1_seq3 comp53374_c0_seq1 comp46540_c0_seq1 comp58248_c0_seq2 comp57696_c5_seq2 comp51106_c1_seq1 comp52388_c1_seq3 comp57051_c16_seq1 comp56818_c0_seq1 comp51144_c0_seq1 comp52012_c5_seq2 comp54655_c1_seq1 comp53935_c1_seq1 comp52553_c2_seq7 comp56536_c0_seq4 comp54931_c0_seq1 comp52099_c2_seq1 comp49391_c0_seq1 comp57836_c1_seq2 comp49679_c3_seq1 comp57384_c7_seq1 comp54455_c0_seq3 comp208828_c0_seq1 comp48236_c0_seq2 comp51833_c0_seq1 comp33047_c1_seq1 comp56486_c1_seq3 comp47861_c0_seq1 comp56438_c3_seq4 comp58380_c0_seq1 comp57704_c3_seq6 comp52372_c1_seq3 comp56636_c2_seq3 comp47560_c0_seq2 comp31321_c0_seq1 comp50422_c0_seq1 comp56104_c3_seq5 comp58218_c0_seq3 comp54830_c2_seq19 comp52834_c0_seq1 comp51030_c0_seq1 comp56530_c1_seq2 comp30684_c0_seq1 comp35812_c0_seq2 comp36745_c0_seq1 comp58273_c0_seq7 comp47962_c0_seq1 comp30074_c0_seq1 comp56047_c3_seq1 comp58252_c3_seq20 comp54520_c1_seq3 comp55765_c1_seq1 comp48890_c0_seq2 comp54183_c1_seq4 comp52438_c0_seq17 comp57727_c0_seq9 comp57443_c11_seq6 comp56534_c1_seq4 comp58004_c0_seq5 comp57022_c12_seq4 comp31507_c0_seq1 comp46661_c0_seq1 comp52592_c1_seq2 comp49860_c0_seq2 comp56184_c2_seq1 comp52386_c1_seq1 comp54277_c0_seq3 comp53739_c1_seq1 comp54478_c0_seq1 comp63663_c0_seq1 comp30883_c0_seq1 comp46402_c0_seq1 comp48466_c0_seq1 comp33456_c0_seq1 comp28400_c0_seq1 comp57254_c9_seq6 comp75628_c0_seq1 comp56111_c1_seq5 comp57871_c0_seq9 comp49519_c1_seq1 comp56890_c0_seq1 comp55886_c4_seq1 comp57679_c1_seq9 comp55187_c1_seq4 comp57290_c1_seq13 comp29921_c0_seq1 comp56632_c2_seq11 comp54932_c1_seq3 comp57480_c0_seq3 comp45610_c1_seq1 comp49199_c2_seq1 comp53376_c0_seq2 comp48114_c0_seq1 comp53744_c0_seq2 comp56877_c0_seq14 comp57165_c1_seq32 comp55811_c0_seq3 comp54712_c0_seq1 comp49403_c0_seq3 comp45752_c1_seq1 comp48528_c0_seq3 comp57593_c0_seq4 comp57191_c0_seq2 comp56000_c2_seq13 comp49961_c3_seq1 comp53953_c0_seq11 comp44497_c0_seq2 comp46928_c1_seq4 comp54689_c0_seq1 comp49388_c0_seq3 comp57737_c0_seq2 comp43910_c0_seq2 comp48443_c0_seq2 comp56884_c3_seq1 comp53362_c1_seq3 comp57280_c1_seq3 comp56669_c0_seq3 comp56716_c6_seq1 comp32099_c0_seq1 comp46340_c0_seq1 comp49480_c0_seq1 comp56979_c5_seq13 comp47741_c0_seq2 comp57907_c3_seq1 comp48605_c0_seq4 comp53491_c0_seq7 comp57175_c0_seq4 comp57198_c2_seq3 comp42753_c0_seq1 comp51312_c0_seq1 comp51097_c2_seq5 comp57743_c4_seq3 comp43744_c0_seq1 comp54945_c0_seq4 comp53545_c0_seq2 comp53748_c0_seq14 comp52493_c3_seq1 comp37734_c0_seq2 comp43654_c0_seq1 comp54592_c0_seq1 comp56954_c2_seq2 comp56835_c0_seq4 comp50148_c0_seq1 comp30120_c0_seq1 comp93203_c0_seq1 comp45542_c0_seq1 comp54767_c0_seq3 comp56361_c2_seq5 comp57879_c0_seq12 comp48386_c2_seq1 comp57147_c0_seq3 comp53860_c0_seq1 comp56956_c0_seq2 comp50247_c0_seq4 comp56091_c0_seq6 comp51796_c0_seq1 comp45818_c0_seq1 comp56100_c0_seq1 comp49823_c0_seq4 comp33140_c0_seq1 comp51957_c4_seq1 comp57381_c0_seq4 comp54727_c0_seq5 comp58267_c1_seq27 comp45748_c0_seq1 comp56425_c0_seq1 comp51261_c0_seq1 comp53534_c1_seq1 comp52842_c0_seq1 comp55264_c7_seq3 comp54486_c2_seq1 comp55798_c1_seq2 comp50912_c0_seq2 comp57689_c1_seq2 comp58029_c1_seq5 comp52514_c0_seq5 comp57984_c3_seq6 comp55950_c0_seq5 comp57629_c3_seq12 comp49928_c0_seq1 comp56952_c6_seq10 comp44182_c0_seq1 comp57673_c1_seq3 comp58480_c0_seq1 comp45052_c0_seq7 comp54540_c1_seq1 comp58267_c1_seq14 comp55184_c10_seq5 comp56442_c3_seq10 comp56106_c2_seq2 comp30753_c0_seq1 comp56788_c0_seq18 comp42080_c0_seq1 comp50703_c1_seq1 comp48836_c1_seq1 comp45791_c2_seq1 comp55302_c0_seq6 comp55831_c2_seq3 comp57507_c0_seq16 comp52466_c0_seq1 comp32059_c0_seq1 comp56857_c2_seq5 comp42223_c0_seq4 comp57643_c1_seq1 comp51429_c0_seq2 comp48559_c1_seq1 comp57787_c2_seq6 comp51173_c0_seq1 comp55421_c0_seq3 comp51721_c0_seq1 comp53179_c0_seq1 comp58160_c1_seq6 comp54673_c0_seq2 comp48155_c1_seq1 comp29781_c0_seq1 comp56223_c2_seq7 comp55141_c0_seq8 comp46046_c0_seq6 comp57807_c7_seq10 comp52245_c0_seq2 comp57620_c3_seq4 comp47518_c3_seq1 comp53722_c1_seq1 comp55623_c9_seq3 comp56192_c4_seq8 comp58016_c3_seq4 comp57845_c0_seq12 comp55986_c3_seq6 comp57468_c0_seq16 comp57808_c1_seq13 comp48400_c1_seq1 comp54285_c1_seq1 comp52788_c0_seq1 comp29811_c0_seq1 comp55235_c4_seq5 comp31526_c0_seq2 comp48134_c0_seq1 comp58255_c1_seq7 comp48181_c0_seq1 comp44400_c0_seq2 comp50244_c3_seq2 comp53353_c0_seq7 comp48967_c3_seq2 comp47565_c0_seq1 comp54090_c0_seq2 comp56988_c0_seq9 comp55861_c2_seq6 comp52299_c0_seq3 comp45825_c0_seq1 comp58087_c1_seq2 comp57522_c1_seq1 comp49977_c3_seq1 comp53605_c0_seq2 comp53971_c0_seq2 comp45816_c1_seq1 comp51636_c1_seq1 comp56616_c0_seq5 comp29461_c0_seq1 comp56565_c0_seq1 comp54889_c3_seq4 comp48782_c0_seq1 comp50992_c0_seq2 comp55278_c1_seq2 comp56395_c5_seq2 comp56227_c1_seq1 comp52125_c0_seq5 comp52780_c5_seq4 comp46296_c1_seq1 comp51489_c0_seq3 comp53925_c0_seq3 comp54977_c0_seq1 comp36760_c0_seq1 comp50133_c3_seq2 comp53631_c0_seq1 comp56403_c0_seq8 comp57430_c1_seq50 comp50379_c1_seq2 comp49598_c2_seq1 comp56304_c2_seq5 comp55185_c0_seq1 comp46407_c1_seq1 comp52872_c3_seq1 comp52517_c0_seq2 comp57092_c1_seq6 comp56956_c0_seq6 comp53800_c0_seq4 comp54193_c6_seq2 comp38027_c0_seq1 comp49748_c0_seq2 comp56822_c0_seq4 comp48828_c0_seq3 comp52354_c0_seq3 comp48755_c0_seq2 comp28263_c0_seq1 comp58008_c2_seq2 comp30524_c0_seq1 comp50893_c0_seq1 comp58153_c0_seq16 comp48950_c0_seq3 comp56113_c0_seq5 comp56447_c3_seq3 comp54021_c1_seq4 comp55295_c0_seq2 comp55648_c1_seq3 comp39782_c1_seq1 comp57229_c3_seq1 comp46372_c1_seq1 comp55997_c0_seq2 comp54394_c4_seq5 comp47139_c0_seq2 comp54069_c0_seq1 comp54357_c0_seq5 comp53955_c2_seq5 comp51710_c0_seq4 comp44921_c0_seq2 comp52405_c0_seq1 comp53759_c0_seq3 comp45420_c0_seq1 comp57487_c5_seq26 comp49082_c2_seq1 comp42031_c0_seq1 comp50585_c0_seq3 comp42159_c0_seq3 comp44395_c1_seq1 comp44642_c0_seq2 comp54849_c3_seq4 comp48757_c0_seq1 comp46585_c0_seq1 comp55283_c0_seq1 comp57900_c4_seq3 comp52471_c0_seq1 comp47307_c0_seq2 comp291525_c0_seq1 comp54841_c8_seq8 comp53351_c0_seq1 comp53291_c0_seq3 comp55873_c1_seq1 comp57532_c3_seq5 comp57680_c5_seq1 comp56572_c9_seq8 comp57891_c0_seq9 comp55260_c1_seq4 comp57199_c0_seq2 comp50704_c1_seq1 comp49055_c1_seq1 comp46254_c0_seq2 comp34620_c0_seq1 comp52534_c0_seq1 comp58182_c2_seq7 comp53849_c0_seq5 comp55859_c1_seq1 comp50092_c2_seq3 comp58082_c1_seq3 comp51287_c0_seq2 comp52316_c1_seq1 comp52600_c0_seq1 comp38193_c0_seq1 comp56905_c0_seq6 comp31895_c0_seq1 comp36036_c1_seq1 comp57016_c0_seq1 comp48587_c2_seq1 comp54261_c8_seq1 comp50745_c0_seq1 comp53024_c0_seq3 comp49112_c1_seq1 comp56023_c2_seq32 comp55177_c0_seq1 comp47227_c0_seq1 comp56588_c1_seq2 comp58005_c0_seq14 comp55732_c1_seq4 comp50743_c0_seq1 comp53101_c0_seq3 comp55735_c0_seq2 comp56568_c0_seq4 comp53244_c0_seq2 comp46693_c2_seq1 comp33360_c0_seq1 comp54619_c1_seq3 comp57605_c2_seq6 comp56025_c0_seq2 comp51859_c0_seq1 comp52568_c0_seq1 comp49903_c0_seq2 comp47935_c0_seq2 comp57575_c2_seq4 comp48071_c0_seq2 comp57471_c2_seq8 comp52946_c1_seq1 comp56919_c1_seq1 comp47655_c0_seq2 comp49648_c1_seq1 comp61667_c0_seq1 comp56925_c0_seq2 comp56685_c0_seq1 comp49023_c2_seq2 comp49669_c0_seq1 comp58063_c1_seq1 comp83958_c0_seq1 comp47653_c0_seq1 comp58302_c0_seq1 comp55749_c7_seq1 comp56622_c5_seq2 comp32423_c0_seq1 comp48716_c0_seq2 comp55354_c1_seq1 comp44156_c0_seq1 comp55280_c0_seq1 comp66674_c0_seq1 comp48756_c1_seq1 comp46345_c0_seq2 comp57591_c2_seq11 comp58108_c1_seq2 comp53460_c0_seq1 comp58284_c6_seq1 comp53564_c0_seq1 comp57771_c0_seq3 comp56179_c4_seq1 comp57399_c2_seq2 comp58194_c0_seq11 comp57853_c1_seq1 comp47097_c0_seq1 comp32435_c0_seq2 comp54701_c0_seq2 comp30482_c0_seq1 comp43258_c0_seq4 comp46483_c0_seq1 comp56069_c1_seq2 comp57965_c0_seq14 comp55195_c4_seq10 comp52487_c1_seq1 comp54880_c0_seq6 comp50848_c0_seq2 comp57746_c1_seq9 comp51384_c0_seq5 comp55570_c1_seq2 comp56611_c1_seq1 comp54706_c1_seq3 comp51871_c0_seq2 comp52647_c1_seq3 comp55865_c1_seq5 comp54549_c1_seq3 comp53717_c0_seq1 comp56667_c0_seq3 comp55633_c2_seq2 comp56244_c1_seq12 comp52312_c0_seq1 comp120206_c0_seq1 comp41420_c2_seq1 comp54246_c0_seq1 comp49719_c0_seq2 comp55744_c5_seq2 comp57767_c0_seq1 comp50054_c0_seq2 comp55334_c0_seq1 comp57208_c3_seq7 comp57973_c1_seq4 comp3716_c0_seq1 comp51847_c2_seq3 comp57846_c0_seq4 comp52873_c1_seq2 comp57791_c0_seq5 comp56395_c5_seq7 comp53798_c2_seq2 comp55277_c2_seq3 comp58246_c0_seq7 comp55386_c0_seq3 comp29831_c0_seq1 comp55753_c2_seq2 comp56664_c1_seq1 comp55763_c0_seq4 comp55087_c0_seq7 comp51818_c0_seq3 comp49960_c0_seq2 comp43402_c0_seq1 comp46211_c0_seq1 comp29730_c0_seq1 comp49987_c0_seq1 comp57470_c0_seq1 comp51257_c0_seq1 comp35563_c0_seq1 comp57382_c2_seq10 comp57320_c1_seq11 comp45875_c2_seq1 comp46324_c0_seq1 comp56519_c0_seq1 comp29285_c0_seq1 comp53187_c0_seq4 comp57863_c4_seq9 comp56889_c2_seq3 comp51165_c0_seq2 comp56599_c0_seq1 comp36274_c0_seq1 comp57372_c1_seq2 comp51973_c0_seq1 comp31938_c0_seq1 comp52270_c0_seq1 comp48342_c0_seq1 comp58150_c0_seq18 comp42341_c0_seq1 comp48856_c2_seq1 comp57997_c3_seq3 comp47786_c1_seq1 comp50421_c0_seq3 comp40663_c0_seq1 comp53388_c1_seq1 comp56190_c0_seq3 comp55067_c0_seq2 comp53616_c0_seq1 comp54305_c2_seq2 comp48321_c0_seq1 comp42954_c0_seq1 comp57858_c1_seq2 comp31633_c0_seq1 comp56230_c14_seq13 comp58017_c1_seq14 comp58292_c0_seq14 comp169403_c0_seq1 comp54350_c0_seq3 comp50665_c0_seq1 comp52564_c0_seq6 comp58270_c3_seq10 comp56734_c3_seq1 comp54077_c0_seq3 comp56600_c1_seq1 comp55629_c1_seq12 comp55087_c1_seq6 comp49136_c0_seq3 comp57997_c4_seq11 comp57265_c0_seq2 comp57214_c0_seq1 comp32753_c0_seq1 comp55993_c3_seq1 comp51291_c0_seq5 comp56316_c0_seq2 comp58062_c7_seq7 comp50114_c1_seq2 comp58074_c5_seq11 comp56922_c2_seq10 comp55312_c0_seq3 comp57316_c1_seq12 comp55979_c0_seq2 comp57363_c1_seq19 comp57224_c0_seq1 comp54979_c0_seq1 comp52087_c0_seq1 comp47465_c1_seq1 comp51052_c0_seq2 comp48727_c0_seq2 comp57492_c3_seq1 comp52091_c0_seq1 comp56604_c3_seq4 comp53067_c0_seq1 comp167461_c0_seq1 comp55080_c4_seq3 comp53983_c1_seq2 comp47906_c0_seq1 comp57335_c0_seq2 comp45714_c1_seq1 comp42852_c0_seq1 comp57615_c1_seq6 comp56610_c9_seq1 comp55063_c6_seq8 comp53797_c1_seq1 comp56867_c3_seq1 comp55175_c0_seq6 comp55665_c2_seq7 comp56429_c0_seq8 comp56878_c9_seq7 comp53654_c0_seq2 comp57669_c0_seq5 comp55312_c0_seq21 comp57006_c0_seq4 comp58262_c1_seq2 comp44893_c0_seq1 comp49439_c0_seq1 comp58097_c2_seq7 comp45237_c0_seq1 comp52818_c1_seq3 comp49339_c2_seq2 comp57136_c1_seq7 comp60452_c0_seq1 comp49798_c1_seq1 comp55947_c1_seq2 comp42227_c0_seq1 comp57533_c1_seq1 comp45273_c0_seq4 comp55915_c1_seq6 comp29742_c0_seq1 comp56543_c0_seq1 comp53347_c1_seq2 comp52521_c0_seq4 comp56727_c0_seq8 comp145707_c0_seq1 comp31813_c0_seq1 comp48080_c0_seq2 comp58105_c1_seq39 comp40905_c1_seq1 comp58198_c4_seq4 comp53848_c0_seq6 comp54304_c4_seq1 comp58294_c2_seq12 comp51966_c0_seq1 comp28948_c0_seq1 comp43992_c0_seq1 comp54776_c0_seq1 comp33117_c0_seq1 comp46848_c2_seq1 comp48981_c0_seq1 comp50663_c2_seq1 comp56299_c6_seq1 comp57645_c1_seq9 comp56176_c3_seq4 comp31276_c0_seq1 comp123311_c0_seq1 comp52094_c0_seq5 comp56373_c0_seq1 comp52660_c0_seq1 comp54608_c1_seq2 comp271_c0_seq1 comp30018_c0
[truncated: 1,675,639 more chars]
